# Membrane‐Permeant, Bioactivatable Coumarin Derivatives for In‐Cell Labelling

**DOI:** 10.1002/cbic.202100699

**Published:** 2022-02-23

**Authors:** Madeleine Schultz, Rainer Müller, Yulia Ermakova, Jan‐Erik Hoffmann, Carsten Schultz

**Affiliations:** ^1^ Cell Biology & Biophysics Unit European Molecular Biology Laboratory Meyerhofstr. 1 Heidelberg Germany; ^2^ Dept. of Chemical Physiology and Biochemistry Oregon Health & Science University Portland, OR USA

**Keywords:** cellular location, click chemistry, fluorogenic dyes, live-cell imaging, membrane permeable dyes

## Abstract

The delivery of small molecule fluorophores with minimal compartmentalization is currently one of the most critical technical problems in intracellular labelling. Here we introduce sulfonated and phosphonated coumarin dyes, demonstrate rapid cell entry via a prodrug approach, and show a lack of interaction with membranes, organelles, or other compartments. The dyes show no specific localization and are evenly distributed in the cells. Our fluorogenic, clickable phosphonate derivatives successfully tagged model targets in intact cells and the increase in brightness upon click reaction was around 60‐fold.

## Graphical abstract

**A pro‐drug approach** delivered sulfonated and phosphonated fluorescent coumarin dyes to live cells without labeling internal membranes. The new dyes exhibited homogeneous cell distribution. In addition, the use of azide groups followed by click chemistry provided fluorogenicity.

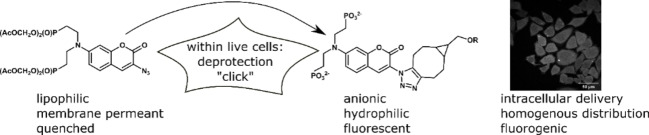

## Introduction

Many small molecule fluorescent dyes are used to label proteins, lipids and nucleotides *in vitro*.^[1]^ The photostability and quantum yields of such dyes are frequently much better than those of fluorescent proteins that are commonly used to tag proteins in cells.^[2]^ However, small molecule dyes often have less utility in experiments within live cells because popular commercial fluorophores such as the Alexa dye series are sulfonated and hence cell impermeant. Other reported small molecule dyes bearing sulfonate and alkyl phosphate groups have been shown to have higher fluorescence quantum yields compared to their neutral analogues due to lack of aggregation, but are also limited in their applicability to live cell studies because of their lack of cell penetration.[^3^, ^4^, ^5^] Conversely, non‐sulfonated dyes such as Cy5 or tetramethylrhodamine (TAMRA) derivatives enter cells readily but accumulate in cell membranes and/or organelles. In fact, there are very few dyes that exhibit all of the desired features required for intracellular delivery and homogenous cell distribution. The most elegant approaches rely on fluorogenic dyes that become fluorescent upon cleavage of a protecting group,^[6]^ or once a labelling reaction is successful.[^7^, ^8^, ^9^, ^10^, ^11^, ^12^, ^13^] Environmentally sensitive dyes have also been effective, such as the silicorhodamines (SiR)[^14^, ^15^] and PYP‐labelling dyes,[^16^, ^17^] which fluoresce more when bound to proteins than when residing in lipid bilayers or the cytosol. An alternative approach is the application of a membrane‐permeating peptide, which carried even highly sulfonated dyes into lysosomes in live cells.^[18]^

In this work, we focused on coumarin dyes, partly because they are small, not prone to π‐stacking, and have reasonable photostabilities and quantum yields. In addition, coumarins have been shown to be useful for performing photo‐chemical reactions in which they are removed from a target molecule.^[19]^ The use of coumarins as photolabile cages for biologically active species is well‐established.[^20^, ^21^] However, standard coumarin dyes such as 7‐diethylamino‐4‐methylcoumarin (**Cou1**) are lipophilic and stain internal membranes readily when applied to cells. Thus, it was of interest to overcome this limitation and ensure an even cytosolic distribution of small coumarin dyes.

We designed coumarins bearing sulfonate and phosphonate groups and applied a prodrug approach to deliver these charged dyes over the plasma membrane. At the same time, to allow applications of the new dyes, we chose to add functional groups for intracellular click reactions using the stable and easily‐handled azide group for lipid or protein labelling.

**Scheme 1:**
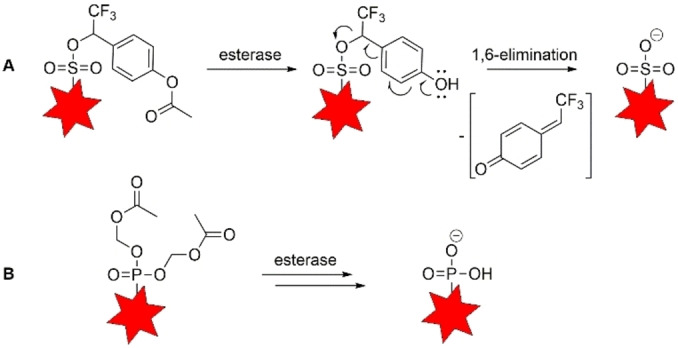
Structure of sulfonate trifluoromethylbenzyl ester (A) and phosphonate acetoxymethyl (AM) ester (B) protecting groups and the mechanisms of prodrug cleavage by esterases (A after Ref. [22], B after Ref. [23]).

The masking of sulfonates and phosphonates relies on the introduction of readily cleavable ester groups which, upon hydrolysis by endogenous esterases, release the charged compound of interest (Scheme 1). Such an approach is well established for phosphonates using acyloxymethyl (AM) esters^[23]^ (Scheme 1B) but has been little used for sulfonates. This is because sulfonate esters are strong alkylating reagents and usually do not have the stability necessary for cell experiments. However, when a combination of electron‐withdrawing groups was used by the Miller group (Scheme 1A), the resulting sulfonate esters were stable and applicable to transfer sulfonated molecules into cells.[^22^, ^24^] We adopted this technique that permitted us to compare cell delivery of sulfonated and phosphonated coumarin dyes.

## Results and Discussion

### Synthesis

**Scheme 2:**
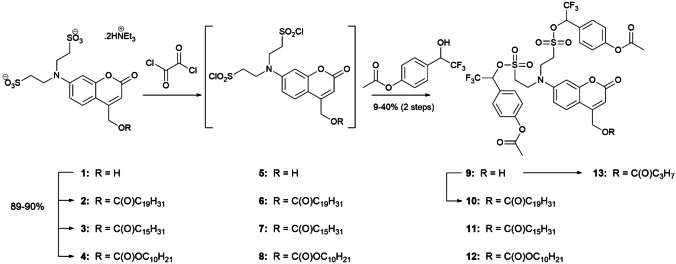
Synthesis of protected sulfonated and lipidated coumarin compounds. Lipid chains: C_19_H_31_ arachidonic acid; C_15_H_13_ palmitic acid; C_10_H_21_ decanoic acid; C_3_H_7_ butanoic acid.

Synthesis of the bioactivatable sulfonated coumarins took advantage of our previously reported route to the sulfonated hydroxymethylene coumarin (compound **1**).^[25]^ The goal was to prepare lipidated as well as 4‐hydroxymethylene derivatives. Starting from 7‐bis(sulfonylethyl)‐amino‐4‐hydroxymethylene coumarin (**1**), we initially synthesized lipidated esters **2** and **3** as well as carbonate **4** in high yields (Scheme 2). These compounds were then chlorinated using oxalyl chloride and further reacted with minimal purification of the intermediate sulfonylchlorides (**6**–**8**) to give the fully protected sulfonate esters **10** and **11**, and carbonate **12** in reasonable yields. The carbonate was useful for cell location experiments because the fatty acid esters may be hydrolyzed in cells. To prepare the non‐lipidated, protected 4‐hydroxymethyl coumarin (**9**), we chlorinated **1** directly to chlorosulfonate **5**, followed by reaction with the trifluoromethyl‐4‐acetoxybenzyl alcohol.^[22]^ This sequence provided the desired 4‐hydroxymethylene bissulfonyl ester, albeit in low yields, probably due to competitive chlorination of the alcohol. The alcohol **9** was esterified in a lower yielding route to arachidonic ester **10** and butyrate **13**.

**Scheme 3:**
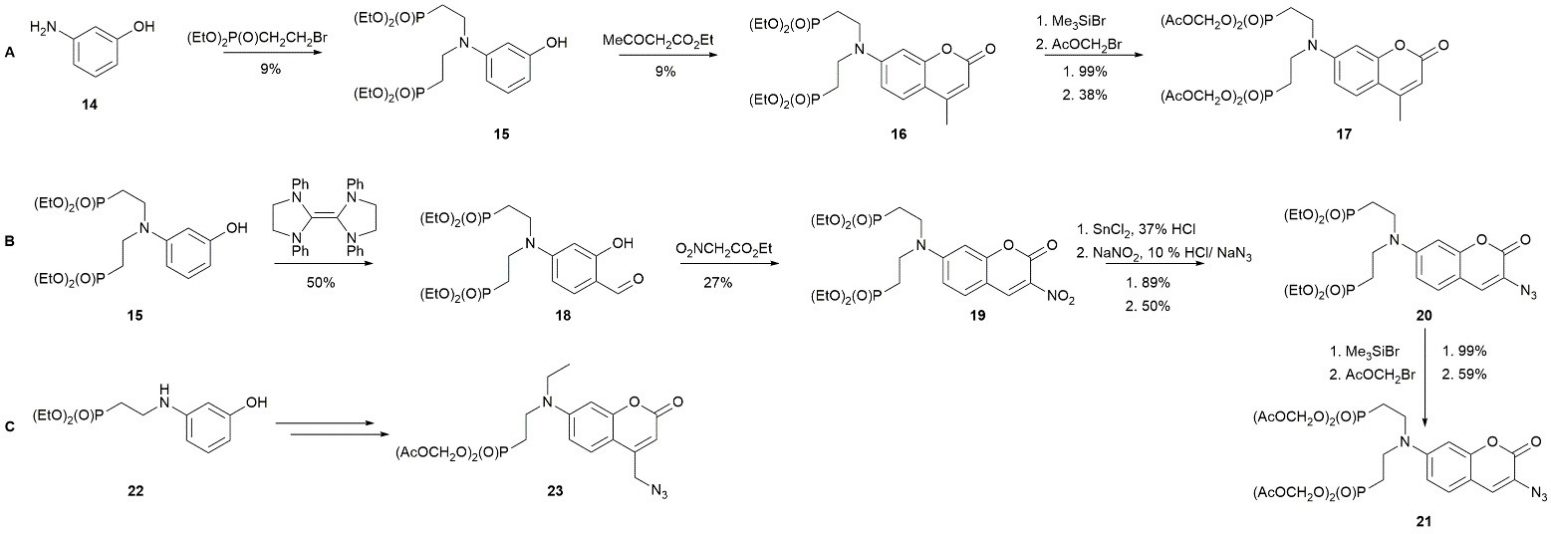
Synthesis of AM ester protected phosphonate compounds **17** (path A), azide **21** (path B) and methylene azide **23** (path C).

The synthesis of the protected phosphonates began with alkylating 3‐aminophenol **14** to intermediate **15** before performing a Pechmann condensation to give coumarin **16** (Scheme 3A). The ethyl groups on the phosphonate were then removed by treatment with TMS−Br followed by alkylation with AM−Br to produce the AM ester‐protected methyl coumarin compound **17**. Alternatively, aldehyde **18** was converted to the AM ester protected azido coumarin **21** via the nitrated coumarin intermediate **19** and azide **20** (Scheme 3B).

In the synthesis of intermediate **15**, a by‐product was obtained with a single phosphonated ethyl group (intermediate **22**). This was further converted to the coumarin methylene azide **23** (Scheme 3C). Comparison of compounds **21** and **23** provided information on the effects of four vs two AM esters and a conjugated vs a 4‐methylene linked azide group on the coumarin.

Upon esterase cleavage, the resulting sulfonated compounds carry one negative charge per sulfonate group. Phosphonate groups usually have between one and two negative charges at physiological pH. For a compound with two phosphonate groups, we estimate an average total charge of −2.4 in PBS, pH 7.4. Thus, both the sulfonated and phosphonated dyes prepared in this work are expected to have negative charges of at least 2 once cleaved by esterases, making them hydrophilic and preventing accumulation in any specific parts of the cell. Cleavage of the sulfonate and AM ester groups by esterase was confirmed *in vitro* by incubating compounds **10**, **12**, **17** and **23** with porcine liver esterase in PBS at room temperature for 15 minutes. After removing the enzyme by methanol precipitation, mass spectrometry of the supernatant allowed the detection of the free sulfonates and phosphonates formed by ester cleavage (data in SI1). Masses were observed corresponding to the sequential loss of each of the protecting groups; the compound becomes anionic and therefore hydrophilic as soon as the first ester group is lost. These experiments indicated the most likely metabolites to be expected in cell experiments.

### Photophysical properties

Several reports have described increased fluorescence for anionic dyes in water compared with neutral analogues, presumably due to reduction in quenching through aggregation or dimerization.[^3^, ^4^, ^26^] In cells, the deprotection and increase in anionic charge will likely lead to a modest increase in fluorescence of our dyes, which is however not distinguishable from dye entry. *In vitro*, the protected compounds have limited solubility in water (or phosphate buffer), making measurements unreliable. Therefore, the photophysical measurements were conducted in ethanol, which has been used extensively in other reports and in which both the esters and the free sulfonates and phosphonates are soluble and stable. Fluorescence spectra of the water‐soluble compounds **17**, **21**, **23**, **27**, **33** and **36** were also measured in a suspension of non‐adherent HL60 cells in PBS (ca. 50000 cells/mL), which should mimic the experimental environment on the microscope stage. These data are included in SI2.

**Scheme 4:**
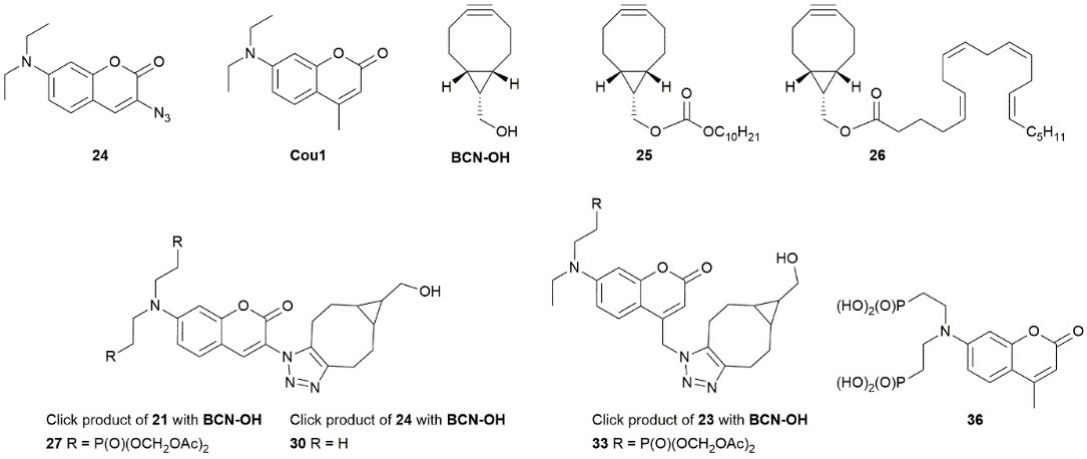
Structures of diethylamino coumarin compounds, BCN click partners, click reaction products and free phosphonate **36**.

Given its structural analogy to the commercially available coumarin azide **24** (Scheme 4),^[7]^ we anticipated that AM ester protected coumarin azides **21** and **23** would be fluorogenic and would become significantly more fluorescent following a click reaction with an appropriate substrate. As control compound, we chose the analogous coumarin 1 (**Cou1**, Scheme 4) for which the photophysical data have been reported.^[26]^ In order to take advantage of copper‐free click reaction conditions that are rapid and suitable for live cells, we chose bicycle[6.1.0]nonyne (BCN) as the click partner. Three compounds with different properties were used in the *in vitro* and live cell experiments (Scheme 4): commercial **BCN‐OH**, BCN‐decyl carbonate **25** and BCN‐arachidonate **26**.

To verify that the click reaction occurs cleanly, samples of the coumarin azides **21**, **23** and commercial **24** were combined with equimolar amounts of **BCN‐OH**, **25** and **26**, respectively in DMSO and diluted with acetonitrile before analysis by UPLC‐MS. In all nine cases, a single product corresponding to the expected triazole with intact AM esters was observed. The structures of some **BCN‐OH** products are shown in Scheme 4 and UPLC‐MS data are provided in SI3.

In order to characterize all coumarin derivatives under identical conditions, fluorescence measurements were conducted using an excitation wavelength of 380 nm. Table 1 summarizes the fluorescence properties of the compounds in ethanol and the spectra are included in SI2. Relative quantum yields were calculated based on the reported value of 0.73 for **Cou1** (Scheme 4) in ethanol.^[27]^ Note that a broad, low intensity emission from **21** and **24** was used to calculate the fold change after click to form **27** and **30**, respectively. An independently synthesized sample of free phosphonate **36**, corresponding to the product formed upon AM ester deprotection of methylcoumarin **17**, has been included and shows red shifted absorption and emission maxima but no difference in quantum yield compared to the protected analogue.

**Table 1:** Photophysical properties of **9**, **10**, **11**, **12**, **17**, **36**, **21**, **24**, **27** and **30** measured in ethanol. Excitation wavelength: 380 nm. Quantum yields were calculated based on the reported value for **Cou1** of 0.73.

Compound	λ_max_ [nm]	ϵ [×10^−4^]	λ_em_ [nm]	Stokes’ shift [nm]	ϕ_fl_	Change in ϕ after click
**9**	355	1.6	448	93	0.72	N/A
**10**	357	0.32	446	89	0.82	N/A
**11**	356	3.5	445	89	0.97	N/A
**12**	356	1.5	448	92	0.50	N/A
**17**	357	2.8	429	72	0.88	N/A
**36**	365	2.8	446	81	0.87	N/A
**21**	378	2.3	457	79	0.01	N/A
**27**	387	2.3	456	69	0.85	60‐fold
**24**	397	3.5	474	77	0.02	N/A
**30**	405	3.2	469	64	0.18	7‐fold

As shown in Table 1, reaction of either of the azido compounds **21** and **24** with an excess of **BCN‐OH** led to a major fluorescence increase in the formed compounds **27** and **30**, respectively. Note that the coumarin azide **24** has been used as a fluorogenic substrate for over a decade.^[7]^ Here, we measured the change in fluorescence quantum yield as a result of the click reaction. Using freshly prepared samples, a seven‐fold increase in fluorescence intensity upon reaction with **BCN‐OH** was observed reproducibly. Interestingly, the increase in fluorescence is much higher for the bisphosphonate coumarin **27** (Table 1). The higher starting quantum yield of **24** and the higher λ_max_ of **30** at 405 nm are likely responsible for the lower fold change compared to **21** forming **27**. The monoethylamino coumarin azide **23** and its click product **33** had similar absorption and emission maxima, but substantially lower fold change compared to the doubly protected pair **21**/**27**. We suspect that this reflects the impact of the additional methylene unit separating the azide from the coumarin fluorophore in those compounds, leading to less effective quenching of **23** and therefore lower fluorescence increase after the click reaction. The data and spectra for compounds **23** and **33** are provided in SI2 along with details of the fluorimetry experiments.

Generally, the observed *in vitro* increases in fluorescence are of the same order of magnitude as previously reported for azides when coupled to form triazoles,^[28]^ but smaller than those reported for tetrazines coupled with TCO.^[12]^ In our experience, a change in fluorescence brightness of 3‐fold is sufficient for use in confocal laser scanning microscopy by choosing settings in which the starting compound is relatively dark and becomes visible only after the click reaction.

### Localization and click reactions in live cells

**Figure 1:**
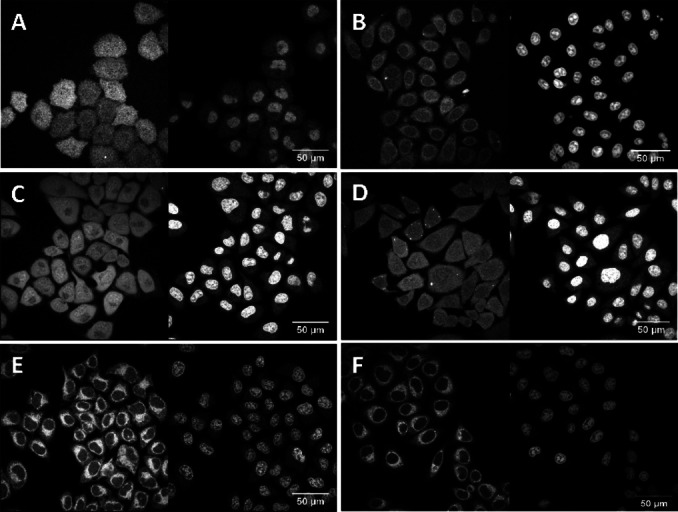
Localization: coumarin compounds in live HeLa cells 20 minutes after addition. A: **9**; B: **10**; C: **17**; D: **21**; E: **Cou1**; F: **24**. Extracellular dye concentration was 10 μM excited with a 405 nm laser and detected at 410–500 nm (left panels); the gain was increased from 500 for A, B, C and E to 900 for D and F to detect these weakly fluorescent compounds. Nuclear stain: DRAQ5 10 μM (right panels). Note that the brightness of the DRAQ5 stain changes over time and has no significance in these experiments. The figures shown are representative for at least six experiments for each compound, with identical outcomes.

The localization of the sulfonate and phosphonate‐containing coumarins formed from incubation of compounds **9**, **10**, **17** and **21** in live HeLa cells was compared to the uncharged compounds **Cou1** and **24** using confocal microscopy (Figure 1).

After 20 minutes incubation, protected sulfonated (**9**, **10**) and phosphonated (**17**, **21**) compounds had entered cells and were quite evenly distributed in the entire cell (Figure 1A–D), indicating complete cell entry and enzymatic cleavage of the protecting groups.[^22^, ^23^] Their localization contrasted starkly with those of the uncharged diethylamino coumarin analogues **Cou1** and **24** (Figure 1E and F), which stained internal membranes and were excluded from the nucleus. The images in Figure 1A, B show that compared to **9**, lipidated **10** stained intracellular membranes and to some extent the nucleus, presumably due to the attached lipid (additional images and data in Figure SI4A, B) while full membrane staining was observed for carbonate **12** (SI4C). This indicated that ester **10** was partially hydrolyzed and released soluble dye (Figure 1B), while the stable carbonate **12** remained membrane‐bound. Compound **17** was brighter in the nuclei than in other regions of the cell (Figure 1C), particularly excluding a perinuclear area that is slightly dimmer (additional image and data in SI4D). The protected monoethylamino coumarin azide **23** localized similarly (SI5A). The azides **21** (Figure 1D) and **24** (Figure 1F) were not visible using standard microscope settings applied for the other compounds, and required a substantial increase of the gain to be observed.

**Figure 2:**
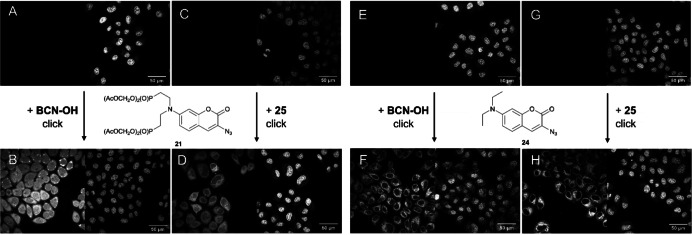
Fluorogenicity and change in localization: Pre‐loading of **21** or **24** followed by addition of BCN‐OH or **25** (10 μM) to live HeLa cells and incubation for 20 min. A: **21**; B: **21**+BCN‐OH; C: **21**; D: **21**+**25**; E: **24**; F: **24**+BCN‐OH; G: **24**; F: **24**+**25**. The extracellular dye concentration was 10 μM (ex. 405 nm, emission 410–500 nm, left panel). Nuclear stain: DRAQ5 10 μM (right panels). All images were collected with identical microscope settings (gain=650). The total dye brightness was similar in B, D and F, and twice as large in H (SI6). Note that the brightness of the DRAQ5 stain changes over time and has no significance in these experiments. The figures shown are representative for at least six experiments for each compound, with identical outcomes.

Consistent with the requirement to increase the gain in order to observe any signal (Figure 1), and as expected based on the fluorimetry data (Table 1), coumarin azides **21** and **24** were essentially non‐fluorescent in cells. Upon addition of **BCN‐OH** or the lipophilic BCN‐decyl carbonate **25** to cells that had been preloaded with **21** or **24**, the fluorescence increased 7–18‐fold, corresponding to the loss of quenching by the azide group when it reacted in a click reaction. The final localization after 20 min is shown in Figure 2 and an increase of brightness is observed in all four cases (data in SI6).

As anticipated, reaction of coumarin azide **21** with **BCN‐OH** in live cells led to both dequenching of the coumarin and esterase deprotection of the AM esters, providing a charged, fluorescent product. This resulted in bright dye distribution in all regions of the cell (Figure 2B). In addition to the increase in brightness, a noticeable difference in localization was observed for the lipid‐clicked product of coumarin azide **21** with BCN‐decyl carbonate **25** (Figure 2D) compared to the initial localization of **21** (Figure 1D). This is best observed by the lower nuclear fluorescence in 2D compared with 1D. In contrast, **21** and its click product with **BCN‐OH** (Figure 2B) were both distributed homogenously throughout the cells, indicating the desired lack of interaction with cell compartments. By comparison, coumarin azide **24** (Figure 1F) showed no change in localization after the click reaction with either **BCN‐OH** or lipidated **25** (Figures 2F, H) because it was already excluded from the nucleus due to its high lipophilicity.

Interestingly, we obtained similar results when the compounds were added in the opposite order, that is first the BCN‐containing lipid **25** followed by azido coumarins **21** or **24** (SI7). The lipidated BCN **25** is expected to bind to membranes upon addition to cells, so the observation of brightness within the nucleus after subsequent addition of **21** indicates that the anionic charge of the deprotected phosphonate is sufficient to alter the lipophilicity of the click product. Lipophilic **24** is excluded from the nucleus when clicked to **25**, regardless of the order of addition.

Analogous experiments were performed first loading monophosphonated coumarin azide **23** onto cells, followed by adding **BCN‐OH** or **25**. This yielded similar localization to that observed with bis(phosphonated) **21** (SI5B, C). In addition, a second lipidated BCN derivative **26** with an ester‐linked arachidonic acid was tested as click partner with **21**, **23**, and **24**. In spite of the highly lipophilic arachidonic acid chain, the final localization of **26** with phosphonated **21** and **23** showed homogenous labelling (SI5D, E), again indicating substantial hydrolysis of the ester. The product of compound **24** with **26** remained bound to internal membranes (SI5F).

These results can be compared with our previous report of a click reaction between coumarin azide **24** and a cyclooctyne‐bearing lipid, in which the click reaction with cyclooctyne was significantly slower (3 h) than that here reported between BCN and azide **24** (20 min).^[29]^ In addition, in our previous work the use of **24** led to membrane staining, which we have now circumvented through use of protected, charged groups in **21** and **23**.

Pre‐incubation of the AM esters **17** (leading to formation of **36**) and **21** with porcine liver esterase prior to addition to live cells led to extracellular localization of the dyes (SI8). The dyes remained in the medium, as was also found for a pure sample of the charged dye **36** added to cells independently. This, along with the mass spectrometry results described above, confirms that ester cleavage reveals the charged phosphonate groups.

A planned application of these dyes is for site‐specific labelling of proteins, whereby fluorogenic azides would become fluorescent upon a click reaction of the azide with a click partner within a protein. This was demonstrated as proof of concept in an *in vitro* experiment as follows. Using Amber stop codon methodology, we prepared GFP^Y39TAG→BCN^, consisting of GFP tagged with an unnatural amino acid containing a BCN group.^[30]^ This purified protein was reacted with coumarin azides **21**, **23** and **24**, respectively. Both SDS‐PAGE and mass spectrometry showed that the click reaction had occurred as designed: a fluorescent band was observed only in the presence of both click partners, and the mass spectrum of the protein increased exactly by the mass of the corresponding dye, while negative controls did not react (SI9).

## Conclusion

We have established robust synthetic routes to protected sulfonate and phosphonate coumarins. These compounds are membrane‐permeant and are deprotected by endogenous esterases to reveal water soluble sulfonate and phosphonate groups. Importantly, these new prodrugs for the first time provide coumarin dyes that evenly distribute in the aqueous cell compartment and lack interaction with membranes. In addition, the prodrug approach has been combined with two chemical reactivities well established for coumarins: fluorogenicity via azide derivatives and click reactions of azides to label a protein site specifically.

## Experimental Section

### Cell culture

HeLa Kyoto cells were grown in DMEM with 4.5 g/L glucose and 10 % FBS and split 1 : 10 every 2–3 days using trypsin. 24 hours before each experiment, 10000–20000 cells were seeded into each well of an 8 well chambered 170 μm coverglass (iBiDi, Munich, Germany) in complete medium.

### Microscopy

Live‐cell imaging of HeLa cells was performed on a Zeiss LSM780 confocal microscope at 37 °C. Growth medium was exchanged for HEPES‐buffered live cell imaging solution (ThermoFisher Scientific, catalog #A14291DJ) 1–2 hours before the experiment, and the nuclear marker DRAQ5 was added 30 minutes before imaging. The coumarin derivatives were excited with 405 nm laser light and emission was monitored between 410 and 500 nm. DRAQ5 was excited with 633 nm laser light and detected between 650–700 nm.

## Conflict of interest

The authors declare no conflict of interest.

1

## Supporting information

As a service to our authors and readers, this journal provides supporting information supplied by the authors. Such materials are peer reviewed and may be re‐organized for online delivery, but are not copy‐edited or typeset. Technical support issues arising from supporting information (other than missing files) should be addressed to the authors.

Supporting Information

## Data Availability

The data that support the findings of this study are available in the supplementary material of this article.
