# Supplementary material for: Membrane‐Permeant, Bioactivatable Coumarin Derivatives for In‐Cell Labelling
Source: Chembiochem. 2022 Feb 23;23(6):e202100699. doi: 10.1002/cbic.202100699 (PMC9305936; doi:10.1002/cbic.202100699)
Supplement: Supplementary file 1 — Supporting Information [file CBIC-23-0-s001.pdf]

ChemBioChem

Supporting Information

Membrane-Permeant, Bioactivatable Coumarin Derivatives for In-Cell Labelling

Madeleine Schultz[+], Rainer Müller[+], Yulia Ermakova, Jan-Erik Hoffmann, and Carsten Schultz*

Table of Contents

SI1: Low resolution mass spectra of ester protected coumarins after incubation with porcine liver esterase........ 2

SI2: Photophysical properties and normalised absorption and emission spectra........ 5

SI3: UPLC traces and mass spectra of *in vitro* click reaction products........ 7

SI4: Localization of 9, 10, 12 and 17 in live cells........ 12

SI5: Localization of 23 and click reactions of 23 in live cells; click reactions of 21 and 24 with 26........ 14

SI6: Fluorescence levels after click reaction in cells........ 14

SI7: Microscope images of BCN click partner followed by coumarin azide added to live cells........ 15

SI8: Microscope images of compounds 17 and 21 after incubation with esterase........ 16

SI9: *In vitro* tagging with purified protein SDS Page and mass spectra of click products........ 17

Sources, materials and equipment........ 21

Experimental details........ 22

NMR spectra........ 47

SI1: Low resolution mass spectra of ester protected coumarins after incubation with porcine liver esterase

The data shows the expected metabolites after removal of bioactivatable protecting groups.

Cleavage of the sulfonate and AM ester groups, respectively, by esterase was confirmed *in vitro* by incubating compounds **10, 12, 17** and **23** (5 µL of 10 mM stock) with commercial porcine liver esterase (10 µL) at room temperature for 15 minutes. Protein was precipitated with methanol (200 µL) and the mixture was centrifuged for 5 minutes at 15000 rpm. Low resolution mass spectra were measured of the supernatants on a Waters Micromass ZQ mass spectrometer. Masses were observed corresponding to the loss of each of the protecting groups; it is to be expected that a mixture of loss of one, two or more protecting groups is observed. Note that a mass corresponding to the intact compound + 122 was observed in each case; this impurity resulted from use of the esterase.

Compound	Calculated mass with protecting groups intact	Cleavage of sulfonate protecting group	Observed (positive ion mode)
**10**	1125.3	909.3 (loss on one sulfonate)	910.9
		693.3 (loss on both sulfonates)	694.6

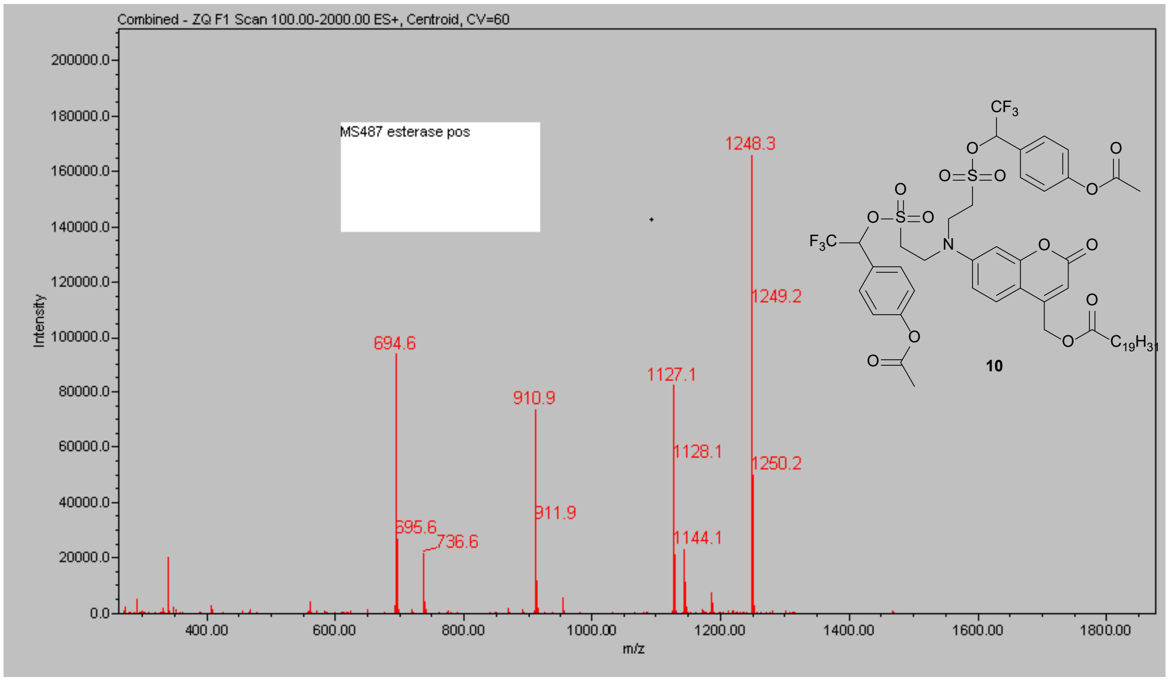

Compound	Calculated mass with protecting groups intact	Cleavage of sulfonate protecting group	Observed (positive ion mode)
**12**	1025.3	809.2 (loss on one sulfonate)	808.6
		593.2 (loss on both sulfonates)	592.4

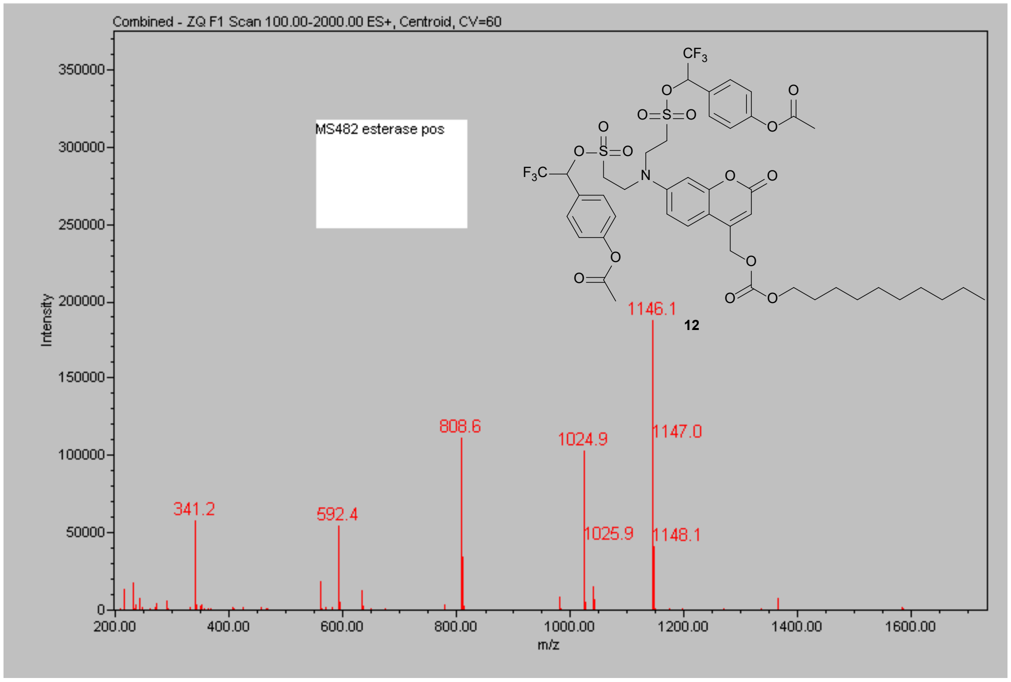

Compound	Calculated mass with protecting groups intact	Cleavage of AM esters	Observed (positive ion mode)
**17**	679.1	607.1 (loss of one AM ester)	608.3
		535.1 (loss of two AM esters)	536.2
		463.1 (loss of three AM esters)	464.2
		391.1 (loss of four AM esters)	392.1

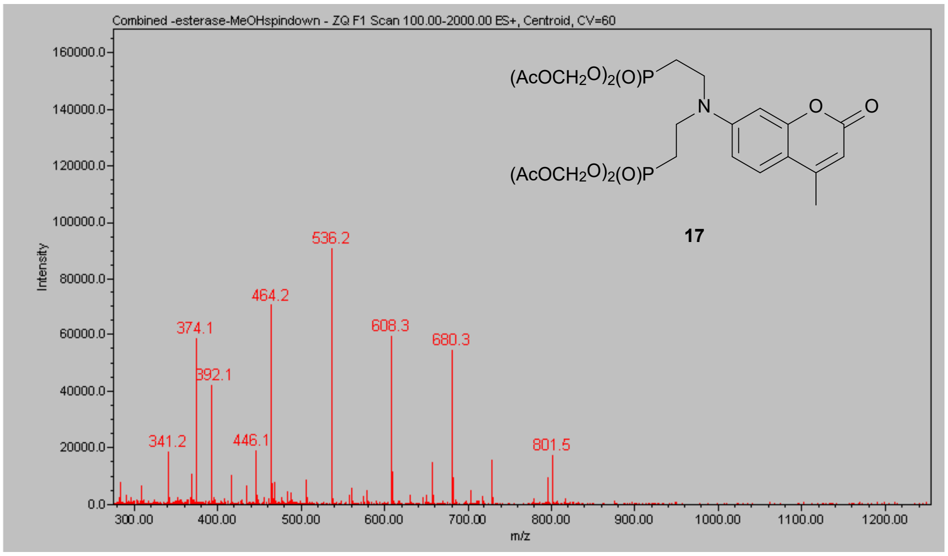

Compound	Calculated mass with protecting groups intact	- AM ester	Observed (negative ion mode)
**23**	496.1	351.1 (loss of both AM esters)	351.1

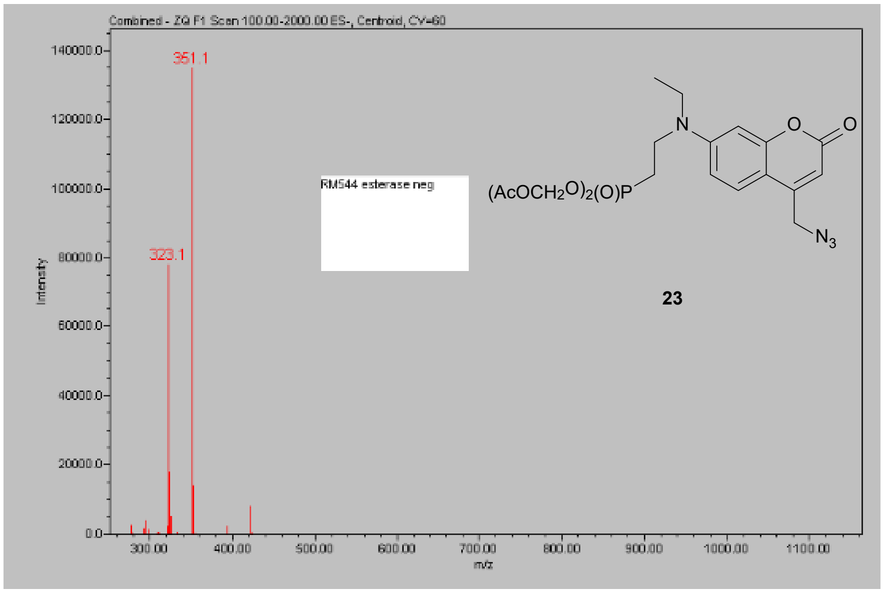

SI2: Photophysical properties and normalised absorption and emission spectra

Photophysical measurements in ethanol were carried out as follows: 1.0 μL of a 10 mM stock solution of the compound in DMSO was diluted to 1.0 mL with ethanol and transferred to a cuvette. The UV-visible spectrum was measured from 300 – 600 nm. 1.0 μL of this solution was then transferred to a fluorimeter cuvette and diluted 1000-fold with ethanol. The fluorescence was measured on a Jasco FP8500 from 390 – 700 nm with an excitation wavelength of 380 nm.

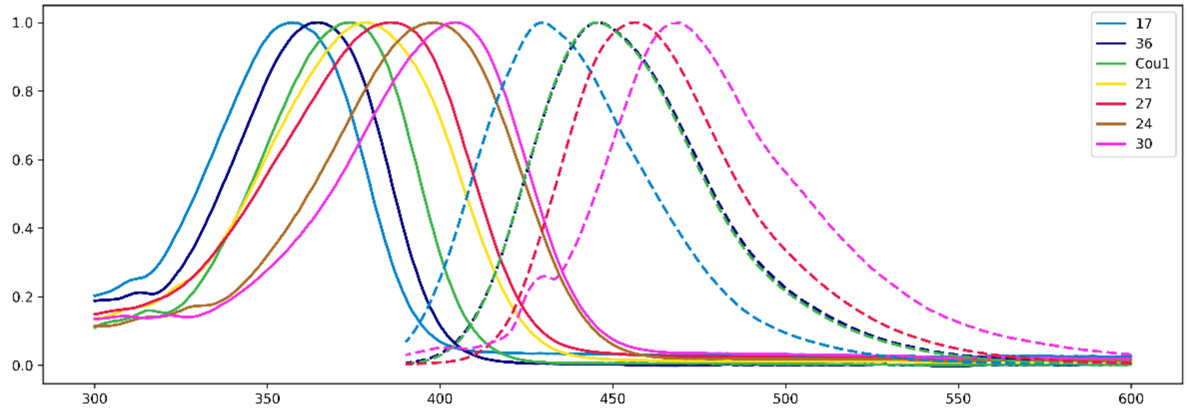

The measurements and spectra for compounds **23** and **33** were also made in ethanol, for comparison with the lipophilic compounds.

compound	λ_{abs} (nm)	ε (x 10^{-4})	λ_{em} (nm)	Stokes' shift (nm)	ϕ_{fl}	Fold change in ϕ_{fl} after click
**23**	372	3.3	461	89	0.13	
**33**	374	2.5	463	88	0.64	5

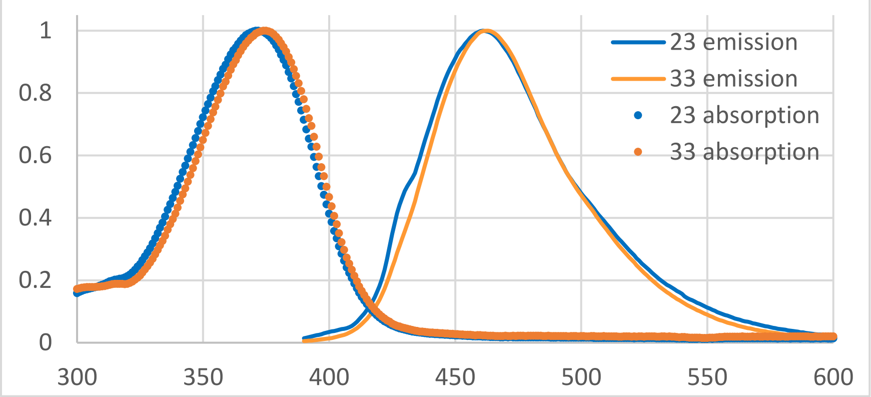

Normalised absorption (solid line) and emission (dotted line) spectra of **23** and **33** in ethanol.

Photophysical measurements of the water-soluble compounds were also made in a suspension of non-adherent HL60 cells in PBS (ca. 50000 cells/mL), which should mimic the environment on the microscope. Compounds **27**, **30** and **33** were prepared in situ by mixing 1.0 μL each of 10 mM DMSO stock solutions **21**, **24** or **23**, respectively with the same amount of a stock solution of **BCN-OH**. After 15 minutes, the mixture was diluted 1000-fold with ethanol before measuring the absorption spectrum, and a further 1000-fold in order to measure the emission spectrum (380 nm excitation wavelength).

Relative and absolute quantum yields were calculated as reported previously.[1]

compound	λ_{abs} (nm)	ε (x 10^{-4})	λ_{em} (nm)	Stokes' shift (nm)	ϕ_{fl}	change in ϕ_{fl} after click
**17**	365	2.6	444	79	0.88	N/A
**21**	382	2.8	456	74	N/A	N/A
**27**	393	2.6	466	73	0.18	7-fold
**23**	382	4.3	478	96	0.04	N/A
**33**	384	4.3	479	95	0.16	4-fold
**36**	380	2.9	465	85	0.12	N/A

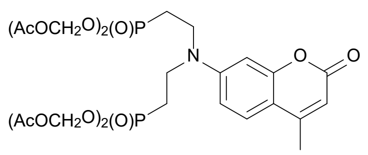

17

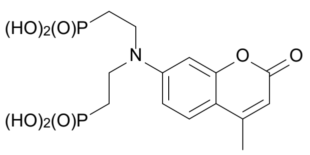

36

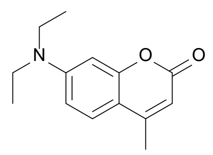

Cou1

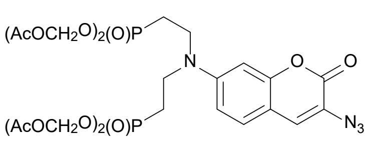

21

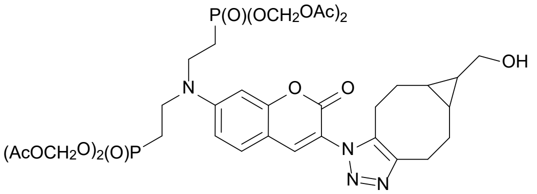

27: Click product of **21** with **BCN-OH**

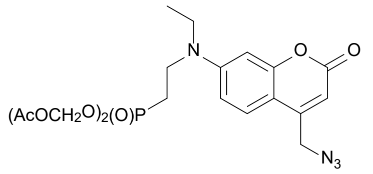

23

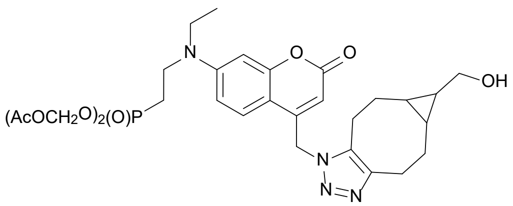

33: Click product of **23** with **BCN-OH**

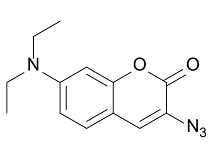

24

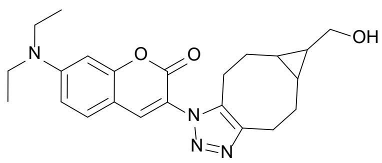

30: Click product of **24** with **BCN-OH**

SI3: UPLC traces and mass spectra of *in vitro* click reaction products

Aliquots of the click partners (1 µL of 10 mM of each compound in DMSO) were incubated at room temperature for 15 minutes. The product was diluted with methanol (500 µL) and subjected to UPLC-MS.

Azide	BCN click partner	Expected click product mass $[M+H]^+$	Expected click product mass $[M+Na]^+$	Observed mass
**21**	**BCN-OH**	857.2	879.2	857.2
**21**	**25**	1042.0	1063.4	1063.6
**21**	**26**	1143.5	1165.5	1143.4
**24**	**BCN-OH**	409.2	431.2	409.4
**24**	**25**	593.8	615.4	593.4
**24**	**26**	695.4	717.5	695.4
**23**	**BCN-OH**	647.2	669.2	647.2
**23**	**25**	831.4	853.4	853.9
**23**	**26**	933.5	955.5	933.4

21 + BCN-OH

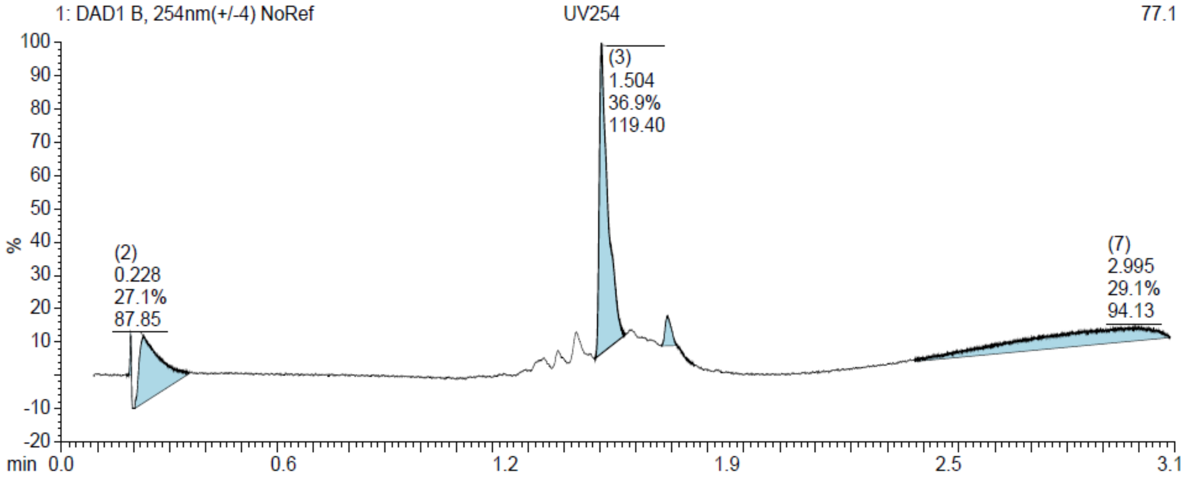

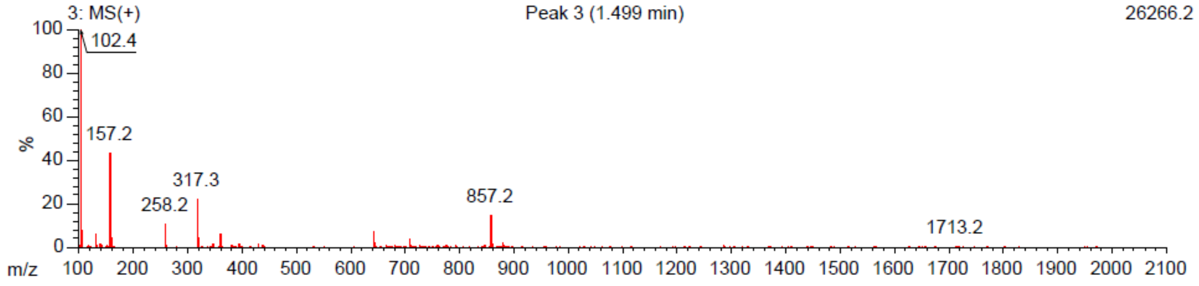

21+ 25

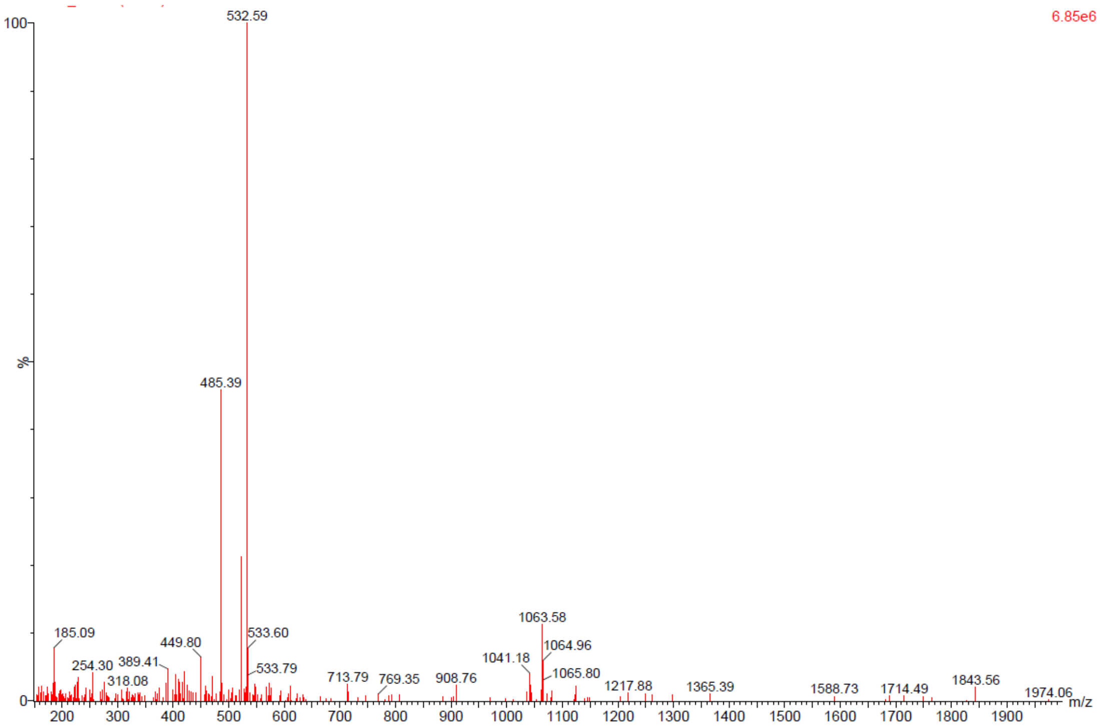

532.59
6.85e6
100
%
0
485.39
185.09
254.30
318.08
389.41
449.80
533.60
533.79
713.79
769.35
908.76
1041.18
1063.58
1064.96
1065.80
1217.88
1365.39
1588.73
1714.49
1843.56
1974.06
m/z
200
300
400
500
600
700
800
900
1000
1100
1200
1300
1400
1500
1600
1700
1800
1900

21 + 26

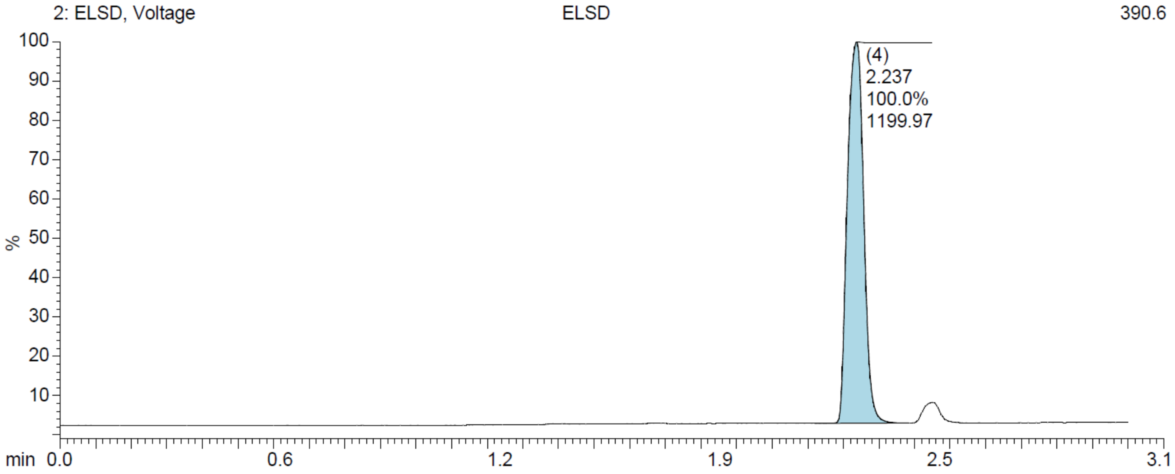

2: ELSD, Voltage
ELSD
390.6
(4)
2.237
100.0%
1199.97
%
min
0.0
0.6
1.2
1.9
2.5
3.1

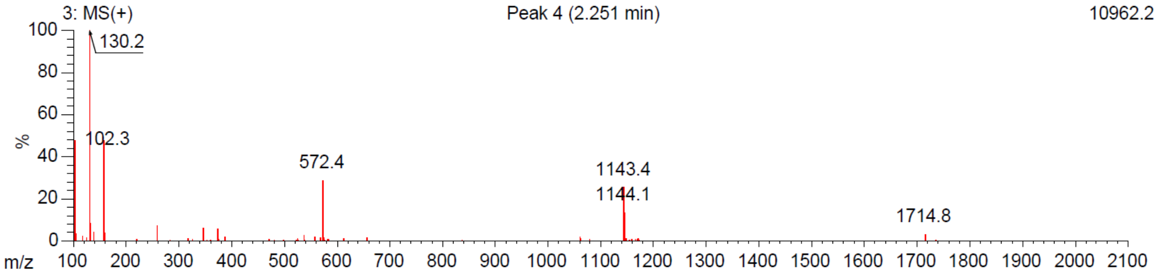

3: MS(+)
Peak 4 (2.251 min)
10962.2
130.2
102.3
572.4
1143.4
1144.1
1714.8
%
m/z

24 + BCN-OH

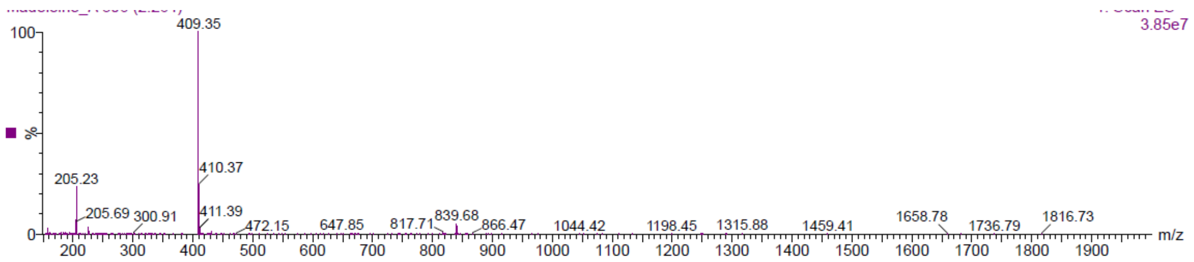

24 + 25

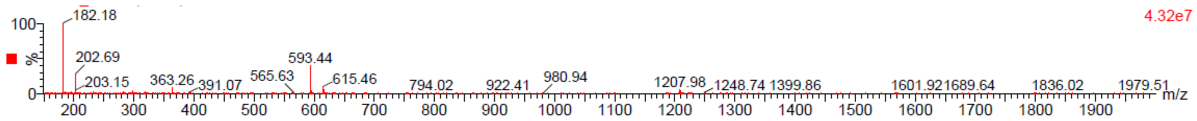

24 + 26

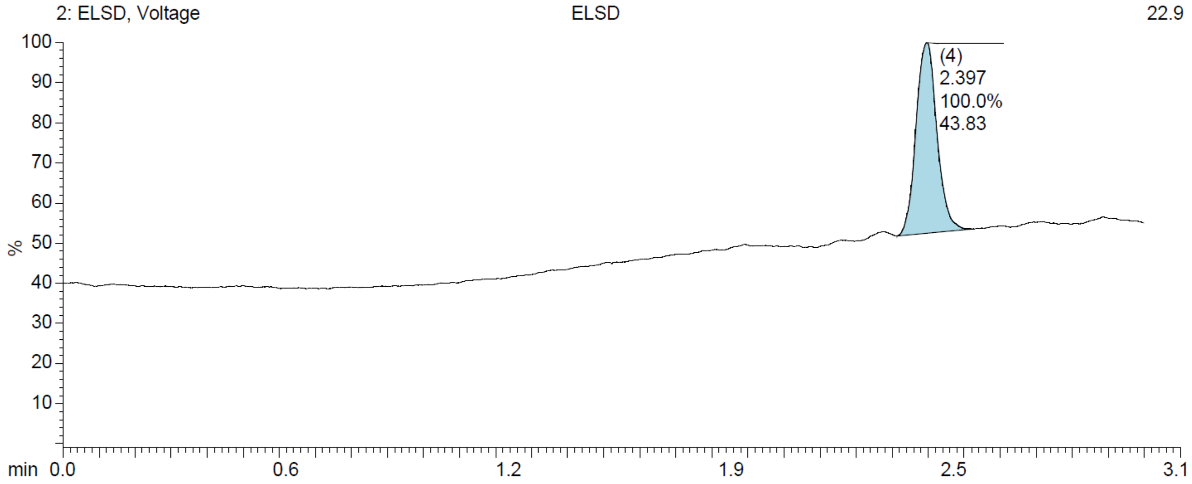

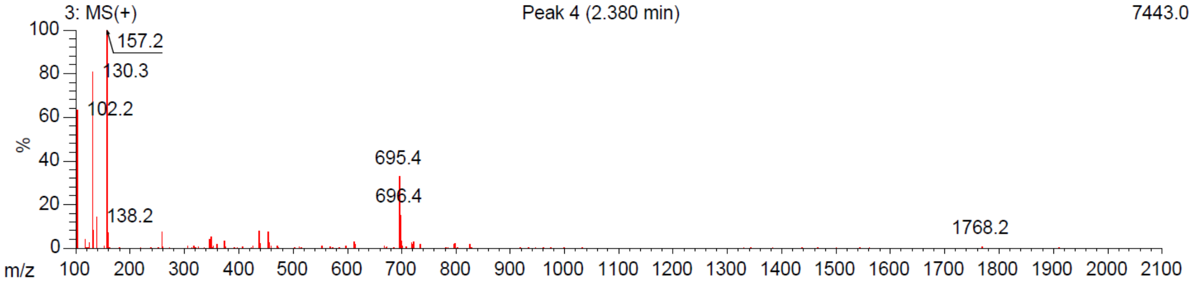

23+BCN-OH

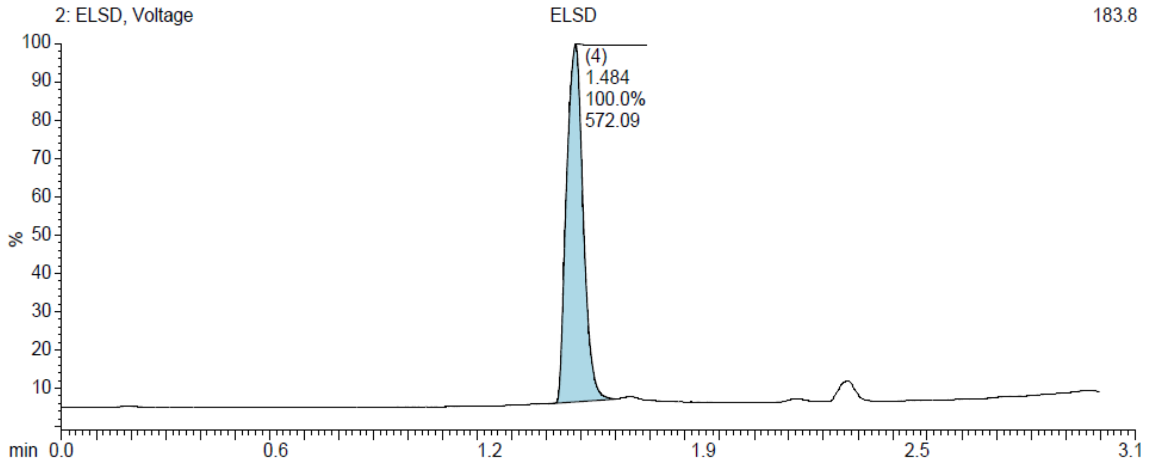

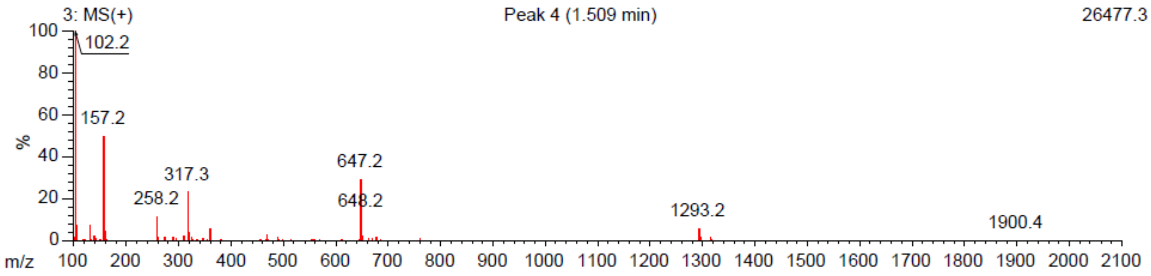

23 + 25

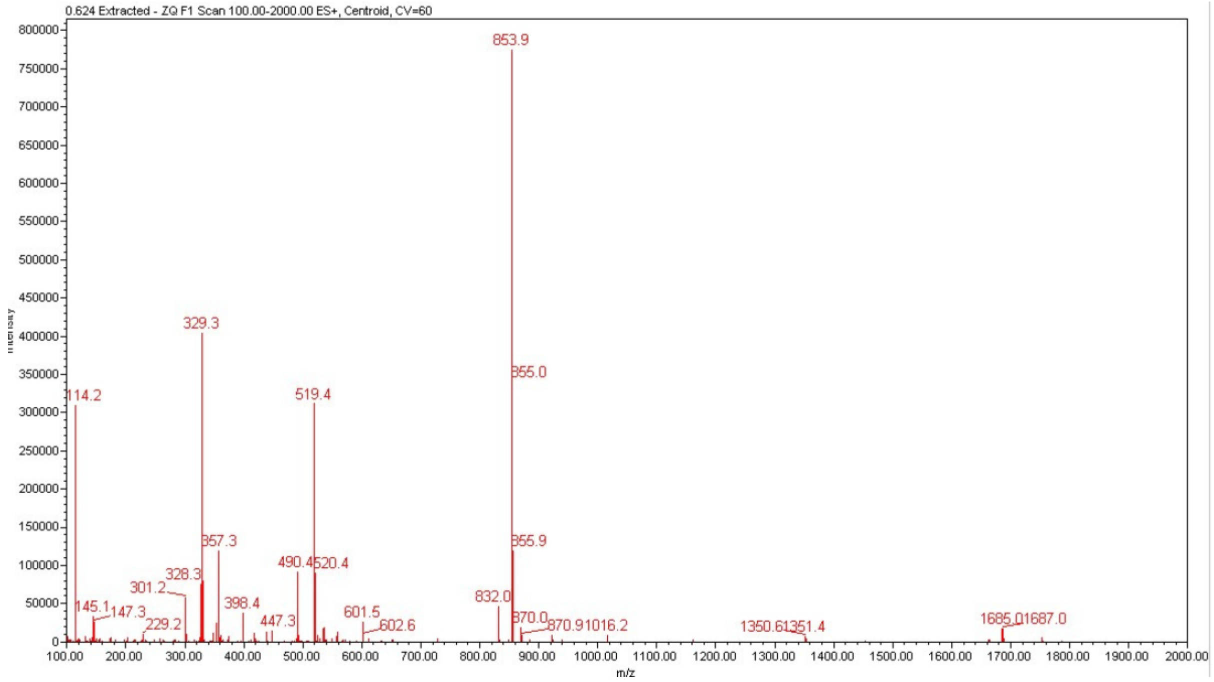

23 + **26**

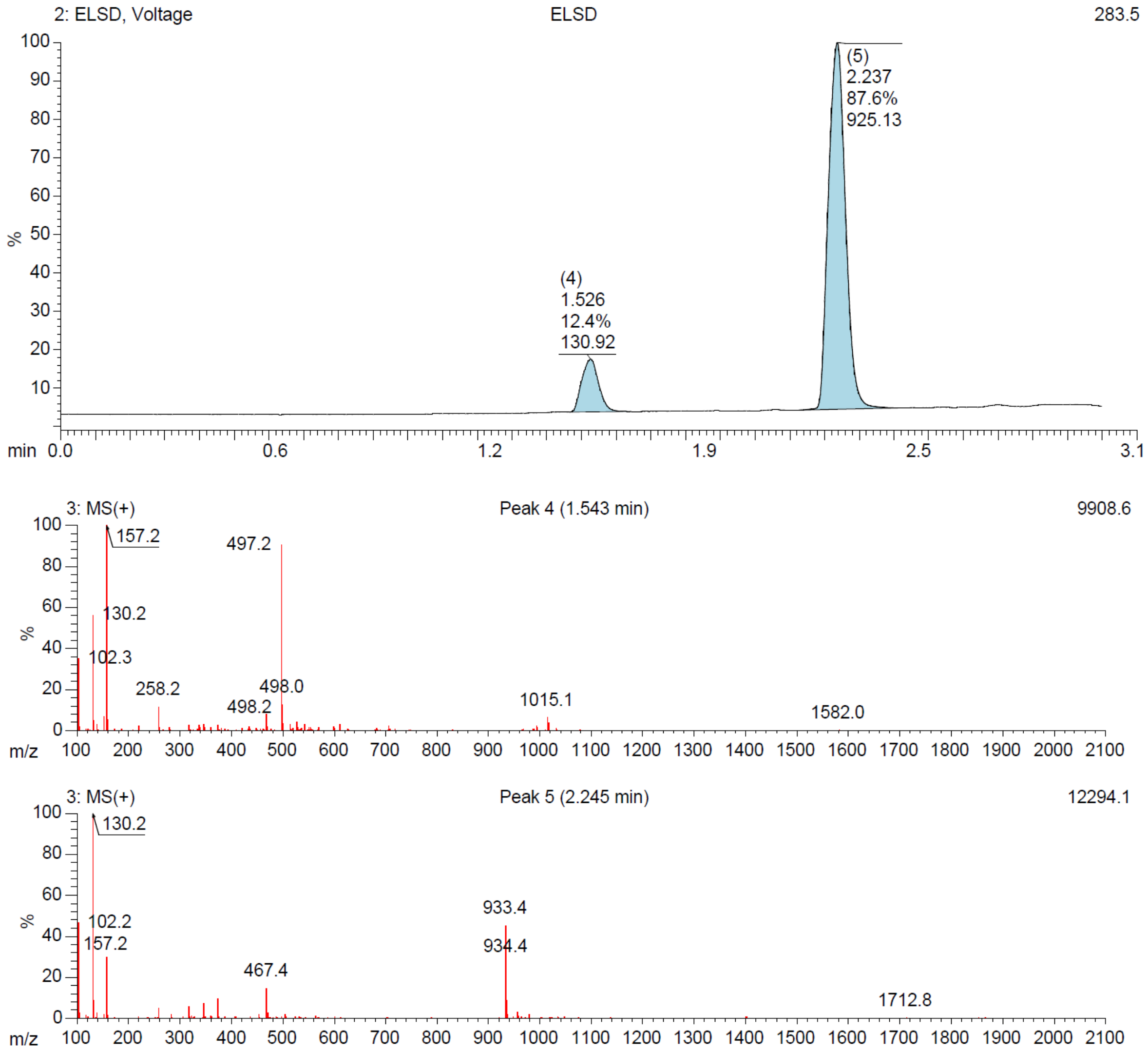
2: ELSD, Voltage
ELSD
283.5
(5)
2.237
87.6%
925.13
(4)
1.526
12.4%
130.92
min
0.0
0.6
1.2
1.9
2.5
3.1
%
3: MS(+)
Peak 4 (1.543 min)
9908.6
157.2
497.2
130.2
102.3
258.2
498.0
498.2
1015.1
1582.0
m/z
3: MS(+)
Peak 5 (2.245 min)
12294.1
130.2
933.4
102.2
157.2
934.4
467.4
1712.8
m/z

SI4: Localization of 9, 10, 12 and 17 in live cells

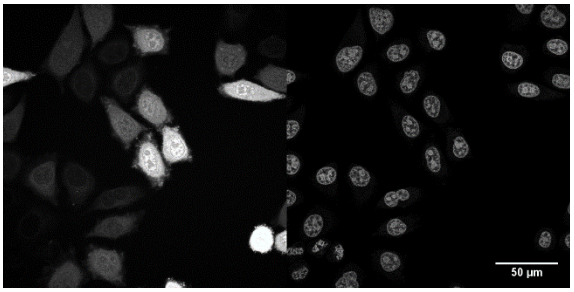

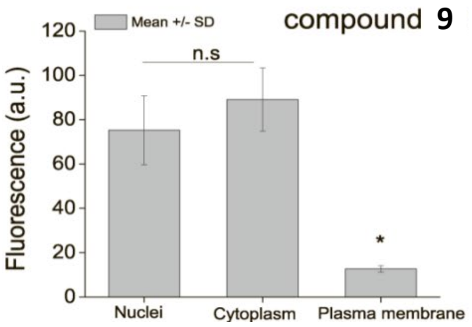

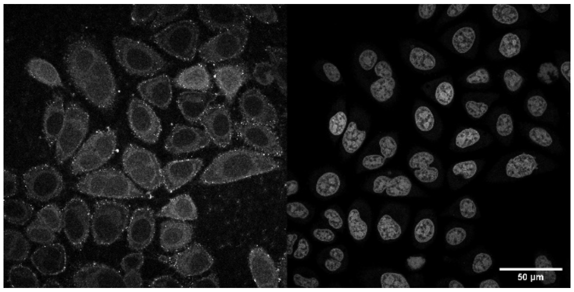

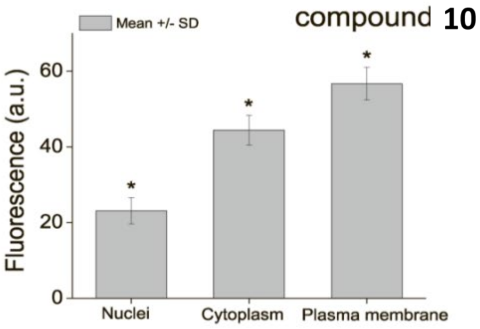

A: Localization of **9** in live HeLa cells. The extracellular dye concentration was 10 µM (ex. 405 nm, emission 410 – 500 nm, left panel). Nuclear stain: DRAQ5 10 µM (right panel). All images were collected with identical microscope settings (gain = 650). Compound **9** is mostly localized in cytosol (50.4% of total signal in a cell body) and in the nuclei (42.50 %) and is poorly presented in the plasma membrane (7.14%). In the cytoplasm, it appears preferably localized in the hydrophilic fraction. The selection of ROIs for the nuclear signal was achieved using an ImageJ macro (https://biii.eu/nuclei-segmentation-2d-imagej) which segments nuclei and separates clustered nuclei using a binary watershed. The result is an index-mask image for each input image. The nuclear signal is the fluorescent signal in the original image inside each ROI of the nuclei mask, and the cytosol signal is the fluorescent signal of the segmented cell with subtracted nuclear signal subtracted. * indicates $p < 0.05$, one-way ANOVA, n = 50 cells per group.

B: Localization of **10** in live HeLa cells. The extracellular dye concentration was 10 µM (ex. 405 nm, emission 410 – 500 nm, left panel). Nuclear stain: DRAQ5 10 µM (right panel). All images were collected with identical microscope settings (gain = 650). Compound **10** is concentrated on the plasma membrane (45.6 % of total signal in a cell body), to a lesser extent in cytoplasm (35.8 %) and is poorly presented in nuclei (18.60 %). * indicates $p < 0.05$, one-way ANOVA, n = 50 cells per group.

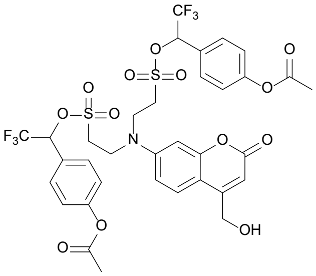

9

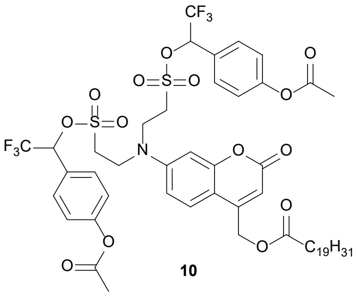

10

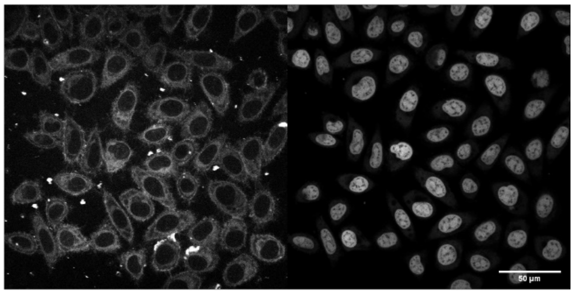

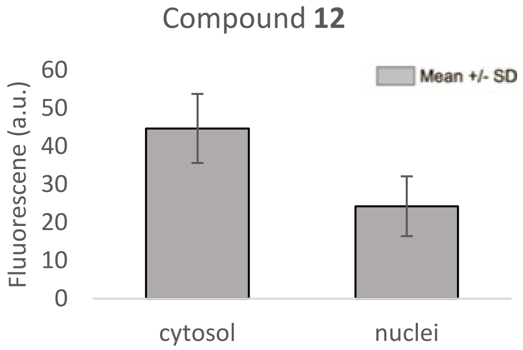

C: Localization of **12** in live HeLa cells. The extracellular dye concentration was 10 μM (ex. 405 nm, emission 410 – 500 nm, left panel). Nuclear stain: DRAQ5 10 μM (right panel). All images were collected with identical microscope settings (gain = 650). Compound **12** is more concentrated in cytoplasm (65%) and has lower concentration in nuclei (35%). $p < 0.05$, one-way ANOVA, n = 30 cells per group.

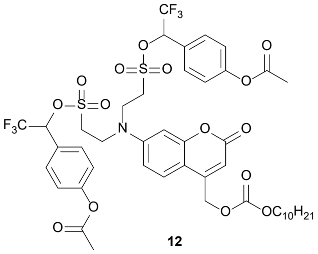

12

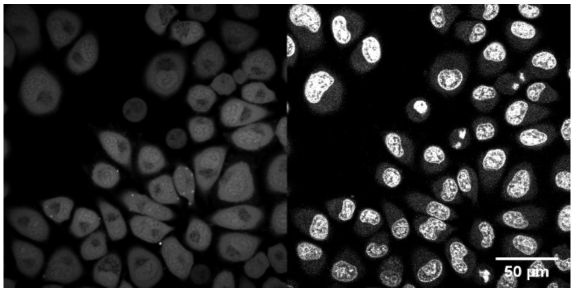

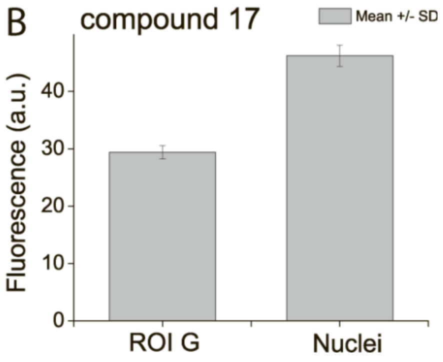

D: Localization of **17** in live HeLa cells. The extracellular dye concentration was 10 μM (ex. 405 nm, emission 410 – 500 nm, left panel). Nuclear stain: DRAQ5 10 μM (right panel). All images were collected with identical microscope settings (gain = 650). In the cytoplasm, compound **17** appears preferably localized in the hydrophilic fraction. The ratio in staining between the perinuclear (Golgi) region and the nuclei is approximately 38.9 : 61.1. n>50 cells, $p < 0.05$, students t-test.

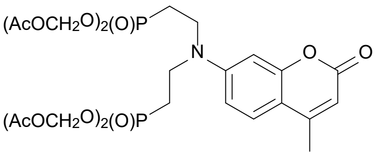

17

SI5: Localization of 23 and click reactions of 23 in live cells; click reactions of 21 and 24 with 26

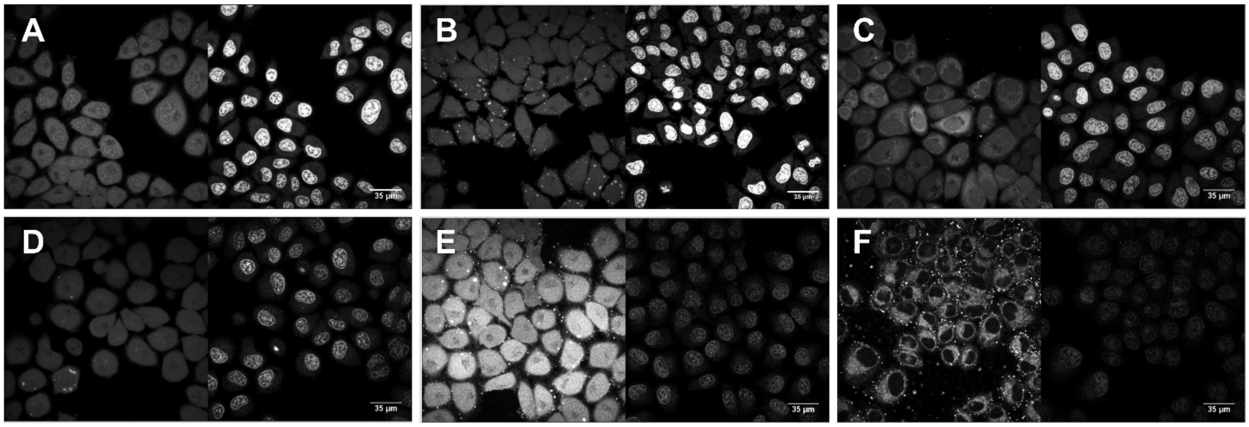

Sequential addition of coumarin azide (20 min incubation) followed by BCN click partner (15 min further incubation) to live HeLa cells. A: **23**; B: **23** + **BCN-OH**; C: **23** + **25**; D: **23** + **26**; E: **21** + **26**; F: **24** + **26**

The extracellular dye concentration was 10 µM (ex. 405 nm, emission 410 – 500 nm, left panel). Nuclear stain: DRAQ5 10 µM (right panels). All images were collected with identical microscope settings (gain = 650).

The localizations of the click products with the carbonate BCN derivative **25** with coumarin azides **21** and **23** are similar (Fig 2D: **21** + **25**; SI5C: **23 + 25**), showing some membrane localization, similar to the localization of compound **12** (SI4C). In contrast, the click products the arachidonic acid ester **26** show homogenous distribution similar to that observed when **BCN-OH** is used as click partner, indicating partial hydrolysis of the ester linkage (Fig SI5E: **21** + **26**; SI5D: **23 + 26**). The commercial diethyl coumarin azide **24** bound exclusively to internal membranes (Fig 2G: **24** + **25**; SI5F: **24** + **26**).

SI6: Fluorescence levels after click reaction in cells

Comparing products of azides **21** and **24** with **BCN-OH** forming compounds **27** and **30** (Figure 2), respectively, versus the reactions of azides **21** and **24** with the lipophilic carbonate (**25**). This control experiment suggests that the increase in fluorescence upon click reaction is more pronounced in the membrane location. * = p<0.05, one-way ANOVA, mean +/-SD, n = 50 cells per group.

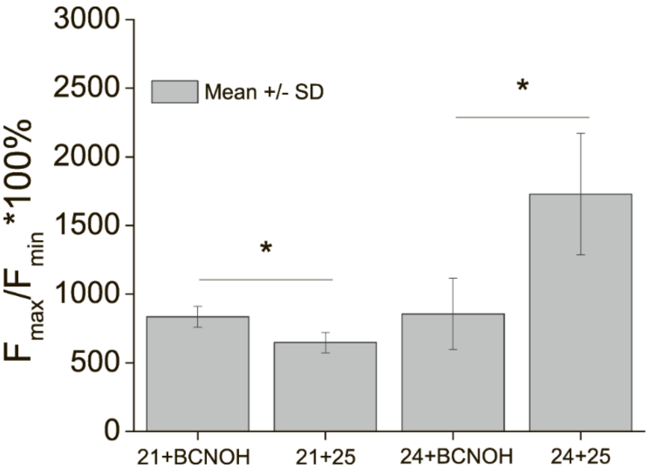

SI7: Microscope images of BCN click partner followed by coumarin azide added to live cells

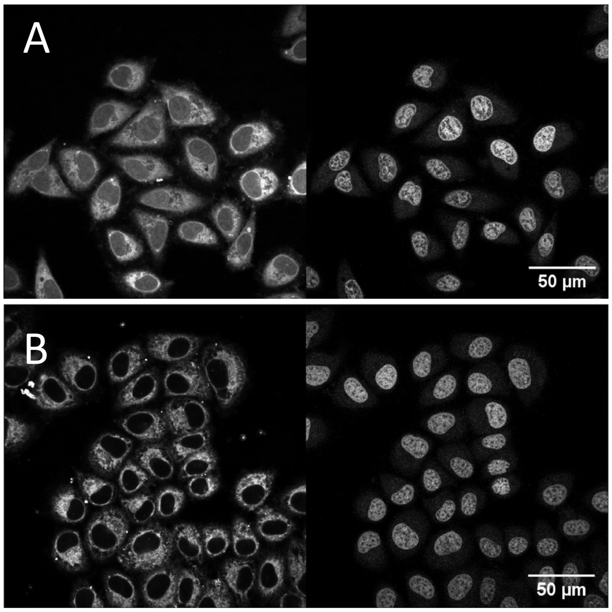

Sequential addition of **25** followed by **21** or **24** to live HeLa cells and incubation for 20 min. A: **25 + 21;** B: **25 + 24**. The extracellular dye concentration was 10 μM (ex. 405 nm, emission 410 – 500 nm, left panel). Nuclear stain: DRAQ5 10 μM (right panels). All images were collected with identical microscope settings (gain = 650).

For comparison, Figure 2 from the main manuscript is reproduced below.

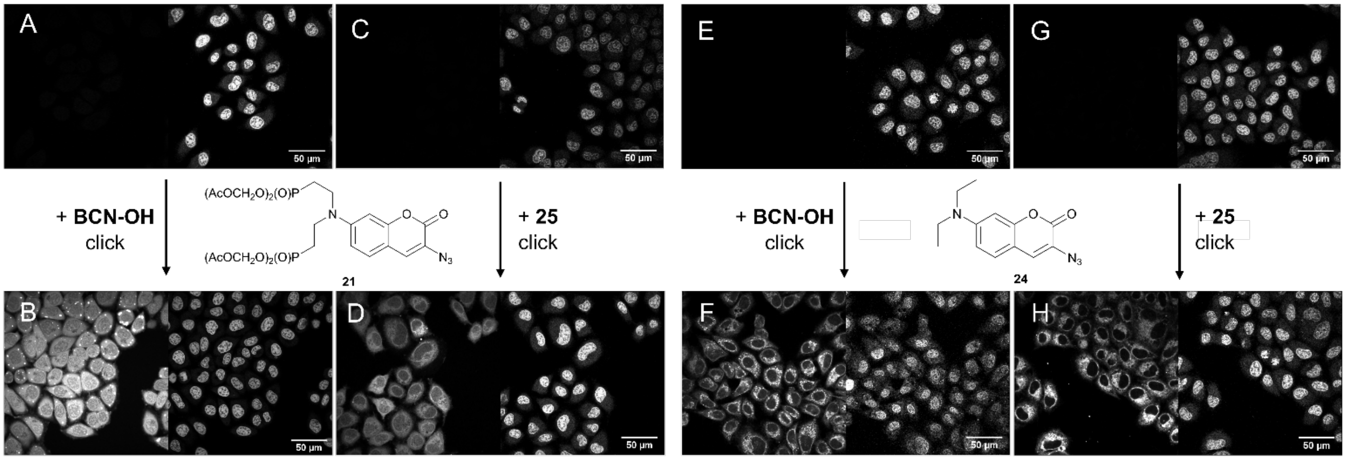

SI8: Microscope images of compounds 17 and 21 after incubation with esterase

Pre-incubation of the AM esters **17** (leading to formation of **36**) and **21** with porcine liver esterase (1 µL of 10 mM dye in DMSO incubated with 10 µL esterase for 15 min) prior to addition to live cells (diluted to a final dye concentration of 10 µM) prevented cell entry and led to extracellular localization of the dyes. The resulting images show that the dyes remained in the medium, as was also found for a pure sample of **36** added to cells independently.

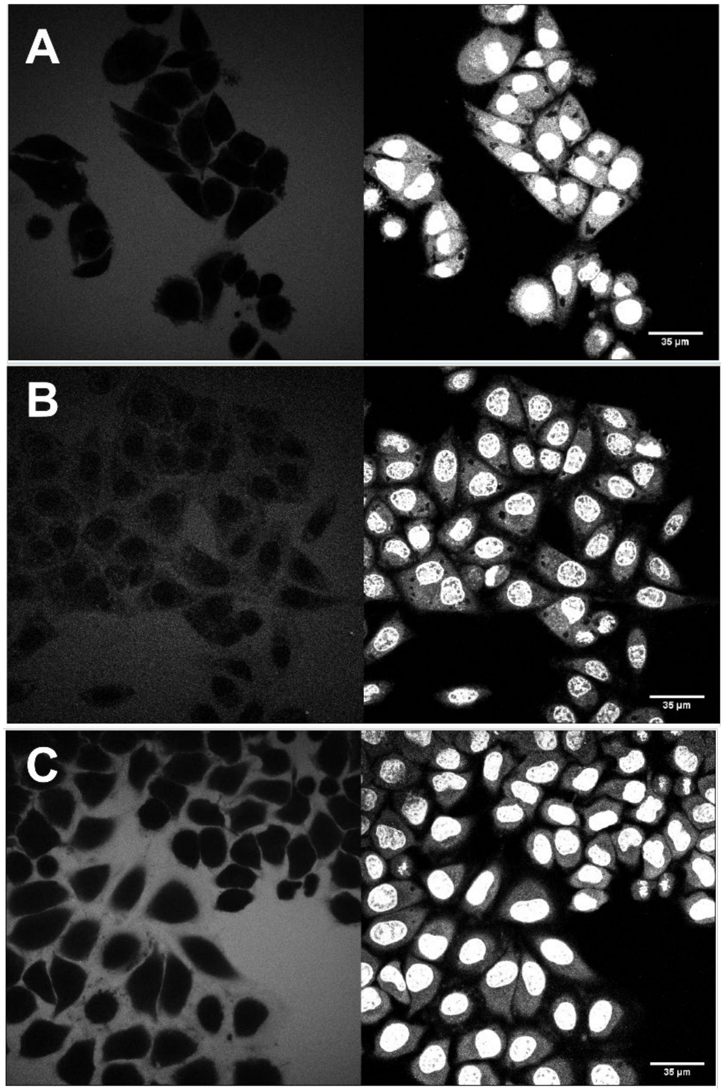

A: **17** preincubated with esterase; B: **21** preincubated with esterase; C: **36.** The extracellular dye concentration was 10 µM (ex. 405 nm, emission 410 – 500 nm, left panel). Nuclear stain: DRAQ5 10 µM (right panels). All images were collected with identical microscope settings (gain = 650).

SI9: *In vitro* tagging with purified protein SDS Page and mass spectra of click products

Click reactions were performed *in vitro* between GFP$^{Y39TAG\rightarrow BCN}$ and the coumarin azides **21**, **23** and **24**. The click products were observed both by SDS-PAGE with excitation at 360 nm and intact protein mass spectrometry. As negative controls, incubation of GFP$^{Y39TAG\rightarrow BCN}$ with **Cou1** and GFP$^{Y39TAG\rightarrow Boc}$ with **21**, **23** and **24** were used.

Note that some signal was observed at 360 nm for the combination GFP$^{Y39TAG\rightarrow Boc}$ + **24** (lane 7) in several independent replicate experiments. However, mass spectrometry confirmed that there was no covalent dye attachment as only the mass of GFP$^{Y39TAG\rightarrow Boc}$ was observed.

SDS-PAGE:

Replicate 1.

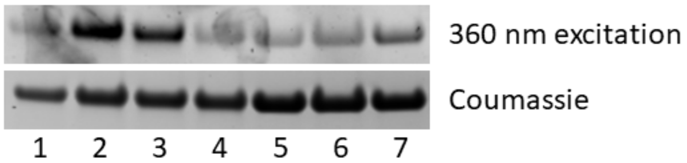

Lane 1: GFP$^{Y39TAG\rightarrow BCN}$ + 21; ratio 360:595 = **0.24**
Lane 2: GFP$^{Y39TAG\rightarrow BCN}$ + 23; ratio 360:595 = **0.38**
Lane 3: GFP$^{Y39TAG\rightarrow BCN}$ + 24; ratio 360:595 = **0.28**
Lane 4: GFP$^{Y39TAG\rightarrow BCN}$ + Cou1; ratio 360:595 = 0.11
Lane 5: GFP$^{Y39TAG\rightarrow Boc}$ + 21; ratio 360:595 = 0.08
Lane 6: GFP$^{Y39TAG\rightarrow Boc}$ + 23; ratio 360:595 = 0.11
Lane 7: GFP$^{Y39TAG\rightarrow Boc}$ + 24; ratio 360:595 = 0.13.

Replicate 2.

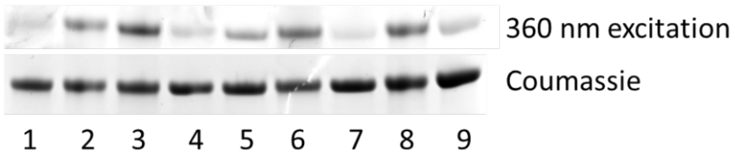

Lane 1: GFP$^{Y39TAG\rightarrow BCN}$ + Cou1; ratio 360:595 = 0.29
Lane 2: GFP$^{Y39TAG\rightarrow BCN}$ + 21; ratio 360:595 = **0.51**
Lane 3: GFP$^{Y39TAG\rightarrow BCN}$ + 24; ratio 360:595 = **0.75**
Lane 4: GFP$^{Y39TAG\rightarrow Boc}$ + 21; ratio 360:595 = 0.19
Lane 5: GFP$^{Y39TAG\rightarrow Boc}$ + 24; ratio 360:595 = 0.24
Lane 6: GFP$^{Y39TAG\rightarrow BCN}$ + 23; ratio 360:595 = **0.73**
Lane 7: GFP$^{Y39TAG\rightarrow Boc}$ + 23; ratio 360:595 = 0.12
Lane 8: GFP$^{Y39TAG\rightarrow BCN}$ + 21; ratio 360:595 = **0.53**
Lane 9: GFP$^{Y39TAG\rightarrow Boc}$ + 21; ratio 360:595 = 0.17.

Ratios of the median intensity within the regions of interest (identical in size for each lane) were calculated in ImageJ after subtracting the background from each image. Combinations of protein and

dye where coumarin fluorescence on the protein was expected are highlighted in bold for each replicate.

Note that some signal was consistently observed at 360 nm for the combination GFP$^{Y39TAG\rightarrow Boc}$ + **24** (Replicate 1: lane 7; replicate 2: lane 5) in multiple independent replicate experiments. However, mass spectrometry confirmed that there was no covalent dye attachment as only the mass of GFP$^{Y39TAG\rightarrow Boc}$ was observed.

Mass spectra:

21 + GFP-BCN

Compound	Compound mass	Protein	Protein mass	Calculated mass of click product with protecting groups intact	Mass after loss of 2 AM-esters	Observed
**21**	706	GFP-BCN	28862	29568	29424	29423

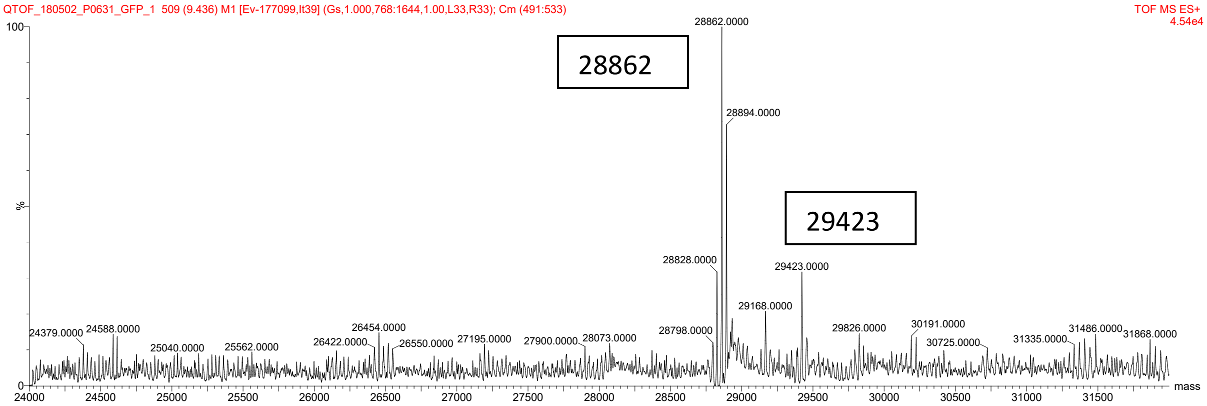

23 + GFP-BCN

Compound	Compound mass	Protein	Protein mass	Calculated mass of click product with protecting groups intact	Mass after loss of one AM- ester	Observed
**23**	496	GFP-BCN	28862	29358	29286	29286

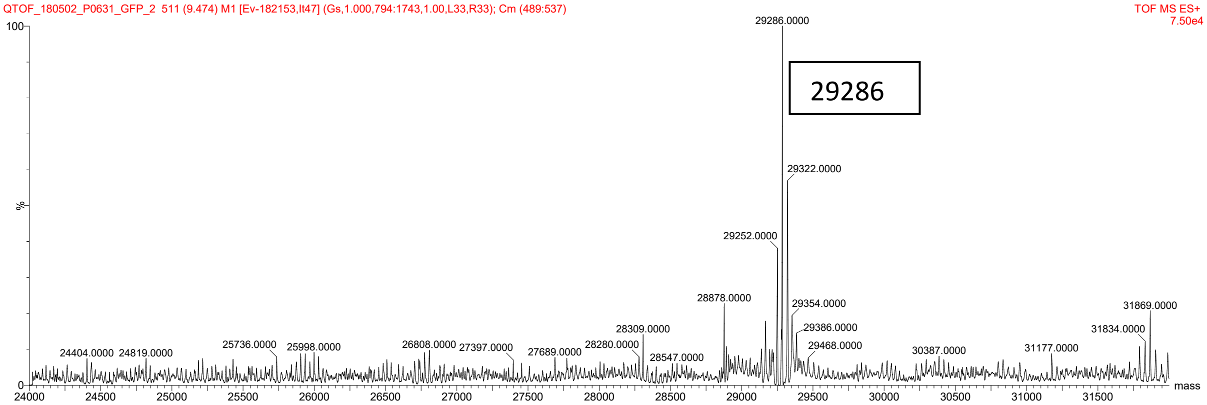

24 + GFP-BCN

Compound	Compound mass	Protein	Protein mass	Calculated mass of click product with protecting groups intact	Observed
**24**	258	GFP-BCN	28862	29120	29119

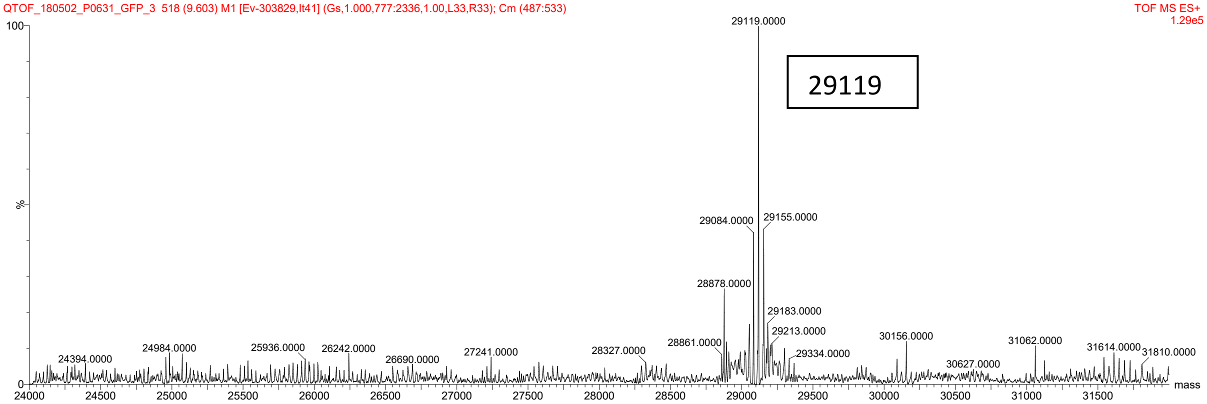

Cou1 + GFP-BCN (negative control)

Compound	Compound mass	Protein	Protein mass	Observed
**Cou1**	258	GFP-BCN	28862	28862

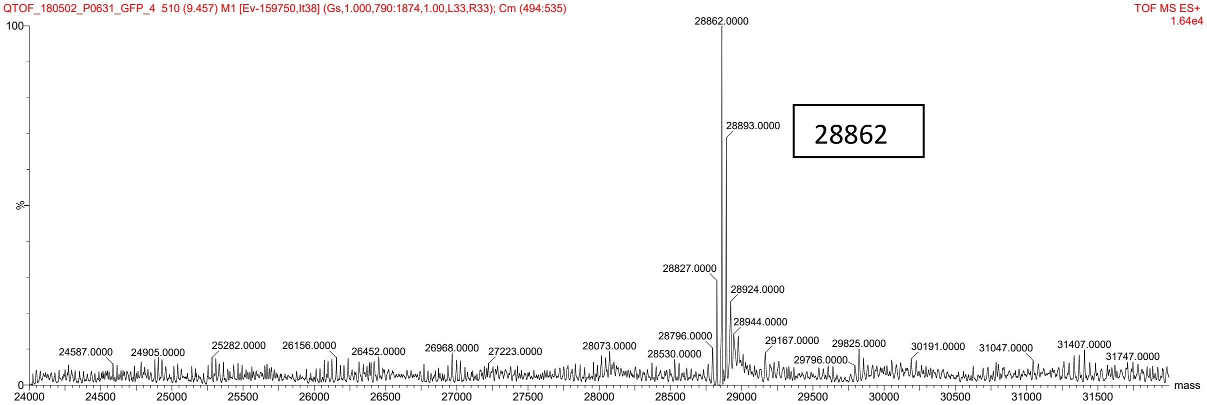

24 + GFP-Boc (negative control)

Compound	Compound mass	Protein	Protein mass	Observed
**24**	258	GFP-Boc	28785	28785

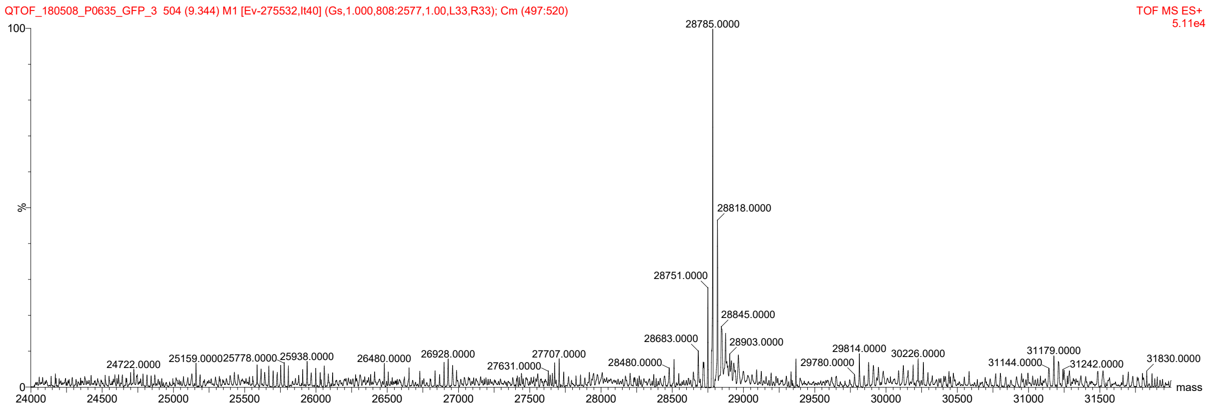

QTOF_180508_P0635_GFP_3 504 (9.344) M1 [Ev-275532,It40] (Gs,1.000,808:2577,1.00,L33,R33); Cm (497:520)
TOF MS ES+
5.11e4
28785.0000
28818.0000
28751.0000
28845.0000
28683.0000
28903.0000
24722.0000
25159.0000
25778.0000
25938.0000
26480.0000
26928.0000
27631.0000
27707.0000
28480.0000
29780.0000
29814.0000
30226.0000
31144.0000
31179.0000
31242.0000
31830.0000
%
mass

Sources, materials and equipment

All chemicals were obtained from commercial sources (Acros, Sigma, Alfa Aesar, TCI or Merck) and were used without further purification. Solvents for flash chromatography were from VWR and dry solvents were from Sigma or Acros. Deuterated solvents were obtained from Deutero GmbH, Karlsruhe, Germany. *Endo*-bicyclo[6.1.0]non-4-yn-9-yl-methanol (endo-BCN-OH) was purchased from SiChem GmbH, Bremen, Germany. All reactions were carried out using dry solvents under inert atmosphere unless stated otherwise in the respective experimental procedure. Compounds **1, 2** and **3** were prepared according to our previous reports (reference 1). TLC was performed on pre-coated plates of silica gel (Merck, 60 F254) using UV light (254 or 366 nm) or a solution of phosphomolybdic acid in EtOH (10 g phosphomolybdic acid in 100 mL EtOH) for visualization. Preparative column chromatography was performed using silica gel 60 (grain size 0.04-0.063 mm) from Macherey-Nagel GmbH, Germany with a pressure of 1-1.5 bar. For RP flash column chromatography LiChroprep® RP-18 material (Merck, grain size 0.040-0.063 mm) or Polygoprep® C18 (Macherey-Nagel, grain size 0.060-0.080 mm) was employed. HPLC analysis was performed on a Knauer Smartline pump 1000 using a Knauer Smartline UV Detector 2500 with a LiChroCART® 250-5 mm cartridge (LiChrospher 100 RP18 (10 µm, Merck, Darmstadt, Germany), or a Shimadzu system equipped with a photodiode array detector (Duisburg, Germany) with a RP18 column (NUCLEODUR C18 ec 5 µm, 4 mm x 250 mm, Macherey-Nagel, Düren, Germany). Preparative HPLC was performed using a Knauer K-1800 preparative pump with a K-2501 UV detector and a Merck Prepbar steel column (250 x 50 mm) filled with RP18 material (LiChrospher®, 220 g, 12 µm, Merck, Darmstadt, Germany) or a Shimadzu system with a RP18 column (NUCLEODUR C18 ec 5 µm, 10 mm x 250 mm, Macherey-Nagel, Düren, Germany. UHPLC-MS analyses were performed on Agilent 1290 series equipment consisting of an Agilent 1290 quaternary pump, a 1290 sampler, a 1290 thermostatted column compartment and a 1290 Diode array detector VL+ equipped with a quadrupole LC/MS 6120 and an Infinity 1260 ELSD. Water was of Milli-Q quality and was obtained after filtration of distilled water through a Milli-Q® cartridge system. For all HPLC experiments, the eluents were acetonitrile-water or methanol-water mixtures with 0.05% TFA unless stated otherwise; compositions are given in % acetonitrile or methanol.

^{1}H-, ^{13}C-, ^{19}F- and ^{31}P-NMR spectra were obtained on a 400 MHz Bruker UltraShield™ spectrometer. Chemical shifts of ^{1}H- and ^{13}C-NMR spectra are referenced to solvent resonances, ^{31}P-NMR spectra are referenced to 85% phosphoric acid. *J* values are given in Hz and chemical shifts in ppm. Splitting patterns are designated as follows: s, singlet; d, doublet; t, triplet; q, quartet; p, pentet; m, multiplet; b, broad. ^{13}C- and ^{31}P-NMR spectra were broadband hydrogen decoupled. Mass spectra (ESI) were recorded using a Waters Micromass ZQ mass spectrometer or a HP Esquire-LC mass spectrometer. High-resolution mass spectra were recorded at the University of Heidelberg (ICR Apex-Qe instrument).
Melting points were determined on a Buechi B-540 and are uncorrected.

Experimental details

Cell culture
HeLa Kyoto cells were grown in DMEM with 4.5 g/L glucose and 10% FBS and split 1:10 every 2-3 days using trypsin. 24 hours before each experiment, 10000-20000 cells were seeded into each well of an 8 well chambered 170 µm coverglass (iBiDi, Munich, Germany) in complete medium.

Microscopy
Live-cell imaging of HeLa cells was performed on a Zeiss LSM780 confocal microscope at 37°C. Growth medium was exchanged for Hepes-buffered live cell imaging solution (ThermoFisher Scientific, catalog #A14291DJ) 1-2 hours before the experiment, and the nuclear marker DRAQ5 was added 30 minutes before imaging. The coumarin derivatives were excited with 405 nm laser light and emission was monitored between 410 and 500 nm. DRAQ5 was excited with 633 nm laser light and detected between 650 – 700 nm.

In-vitro labeling of purified proteins
eGFP$^{Y39TAG\text{->}BCN}$ and eGFP$^{Y39TAG\text{->}BOC}$ were expressed in *E. coli* and purified via His-tag on Ni-NTA beads as described earlier.[2] 100 nM protein solution in PBS was incubated with 1 µM coumarin compounds **21**, **23**, **24** and **Cou1**, respectively, at 37°C for 15 minutes. The samples were denatured at 95°C with 4x sample buffer, loaded on Novex 4-12% Bis-Tris gels

(ThermoFisher Scientific, Waltham, MA, USA) and separated in MES running buffer at 150V. Fluorescent images were taken before fixation with 360 nm excitation on a Typhoon gel scanner and the gels were subsequently stained with Coomassie blue in 20% methanol.

Intact mass spectrometry measurements of labelled proteins

Protein samples were acidified using 1% TFA prior to injection onto the Acquity UPLC System (Waters Corporation). Approximately 5 µg of each sample were loaded onto a protein separation column (Acquity UPLC Protein BEH C4 column, 2.1 mm x 150 mm, 1.7 um). The outlet of the analytical column was coupled directly to a quadrupole time of flight (Q-TOF) Premier mass spectrometer (Waters/Micromass) using the standard ESI source in positive ion mode. Solvent A was water, 0.1% formic acid, and solvent B was acetonitrile, 0.1% formic acid. The samples were loaded in 96% A, 4% B at a constant flow of 0.2 mL min-1. The column was held at 4% B for 5 min before ramping to 25% B by 6 min. A linear gradient to 80% B was then applied until 17 min. For the Q-TOF, a spray voltage of 3.5 kV was applied with a cone voltage of 35 V and extraction cone at 5 V. The desolvation temperature was set at 350 °C, with source temperature 120 °C. Desolvation gas was nitrogen at a flow rate of 500 L min-1. Collision energy was set at 5 eV with argon in the collision cell at a pressure of 4.5·10-5 mbar. Data was acquired in continuum mode over the mass range 500–3500 m/z with a scan time of 0.5 s and an interscan delay of 0.1 s. Data were externally calibrated against a reference standard of intact myoglobin, acquired immediately prior to sample data acquisition. Spectra from the chromatogram protein peak were then summed and intact mass was calculated using the MaxEnt1 maximum entropy algorithm (Waters/Micromass) to give the zero charge deconvoluted molecular weight.

Syntheses

(7-*N,N*-Bis(2-chlorosulfonylethyl)amino)-2-oxo-2H-chromen-4-yl)methylene arachidonate (**6**) and

(7-*N,N*-bis(2-(1-(4-acetoxyphenyl)-2,2,2-trifluoroethyl)sulfonylethyl)amino-2-oxo-2H-chromen-4-yl)methylene arachidonate (**10**)

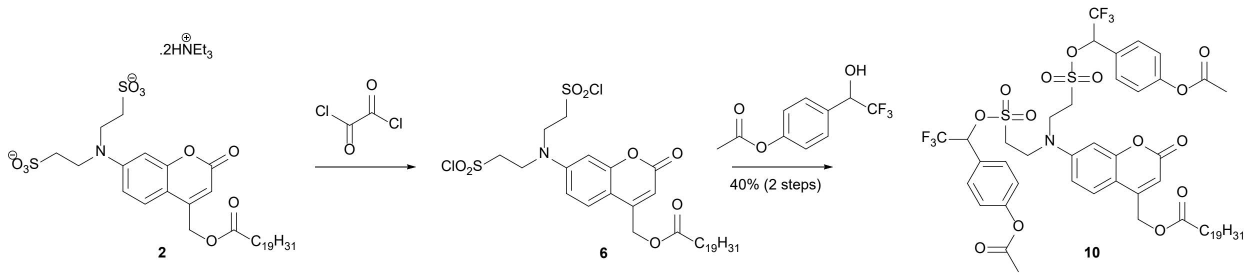

In a Schlenk tube equipped with a stirrer bar, 35 mg of sticky brown **2** (35 mg; 39 μmol) was put under Ar and DCM (2 mL) was added. The solution was cooled to 0ºC with stirring, following which oxalyl chloride (8.4 μL, 100 μmol) was added. DMF (1 drop) was then added. The mixture was stirred and allowed to warm to room temperature. After approximately 40 minutes stirring at RT the orange solution was put under vacuum for 2 hours to remove all volatile components including unreacted oxalyl chloride to yield **6**. The mass spectrum confirmed that the bissulfonyl chloride **6** had been formed (observed 730 $[M+H]^+$). The sticky brown product was redissolved in DCM and DABCO (1.2 equivalents) and the alcohol were added. After stirring for 2 hours at room temperature, the solvent was removed under vacuum and the mass spectrum confirmed formation of **10**, which was purified by HPLC. Note that the compound exists as a mixture of two diastereomers with very similar NMR spectra; the ^{19}F, phenyl ring, ethylene chain and acetate resonances were observed separately for the diastereomers.

^{1}H NMR (400 MHz, $CDCl_3$): δ = 7.46 (two diastereomers observed, d, 4H, $^3J_{HH}$ = 6Hz, $CF_3CHCC\underline{H}$), 7.36 (d, 1H, $^3J_{HH}$ = 9Hz, H-5), 7.19 (two diastereomers observed, d, 4H, $^3J_{HH}$ = 6Hz, C(=O)OCCH), 6.44 (unresolved dt, 1H, H-6), 6.33 (unresolved t, 1H, H-8), 6.26 (br s, 1H, H-3), 5.78 (q, 2H, $^3J_{HF}$ = 6Hz, $CHCF_3$), 5.42 – 5.31 (m, 8H), 5.24 (s, 2H, CH_2O), 3.79 – 3.71 (two diastereomers observed, m, 4H, NCH_2), 3.28 – 3.20 (two diastereomers observed, m, 4H, CH_2S), 2.82 (m, 6H), 2.47 (t, j = 8Hz, 2H, $C{=}OCH_2$), 2.31 (two diastereomers observed, s, 6H, $C{=}OCH_3$), 2.15 (m, 2H), 2.06 (m, 2H), 1.78 (m, 3H), 1.3 – 1.2 (m, 8H), 0.88 (t, j = 7Hz, 3H, $CH_2C\underline{H}_3$).

^{13}C{^{1}H} NMR (101 MHz, $CDCl_3$): δ = 172.7 ($\underline{C}{=}OCH_3$), 169.0 ($\underline{C}{=}OCH_2$), 160.7 (C-2), 155.8, 152.6, 149.1 (C-4), 147.7, 130.5, 129.5, 129.2, 128.6, 128.5, 128.3, 128.0, 127.8, 127.5, 126.7 (C-5), 125.4, 122.1 (q, $^1J_{CF}$ = 281 Hz, CF_3), 109.1 (C-6), 109.0 (C-3), 108.7, 99.5 (C-8), 77.6 (q, $^2J_{CF}$ = 35 Hz, $\underline{C}HCF_3$), 60.9 (CH_2O), 49.1 (CH_2S), 46.0 (NCH_2), 33.4 ($C{=}O\underline{C}H_2$), 31.5, 29.7, 29.3, 27.2, 26.5, 25.6, 24.7, 22.6, 21.1 ($C{=}O\underline{C}H_3$), 14.1 ($CH_2\underline{C}H_3$).

^{19}F NMR ($CDCl_3$): δ = -75.7 (two diastereomers observed, d, $^3J_{FH}$ = 6Hz).

HRMS: Chemical Formula: $C_{54}H_{62}F_6NO_{14}S_2$ $[M+H]^+$

Exact Mass: Calculated 1126.35160 Found 1126.35335

Chemical Formula: $C_{54}H_{61}F_6NNaO_{14}S_2$ $[M+Na]^+$

Exact Mass: Calculated 1148.33354 Found 1148.33357

UV (EtOH): λ_{max} = 356 nm, ε = 16000 $Lmol^{-1}cm^{-1}$

(7-*N,N*-Bis(2-chlorosulfonylethyl)amino)-2-oxo-2H-chromen-4-yl)methylene palmitate (**7**) and (7-*N,N*-bis(2-(1-(4-acetoxyphenyl)-2,2,2-trifluoroethyl)sulfonylethyl)amino-2-oxo-2H-chromen-4-yl)methylene palmitate (**11**)

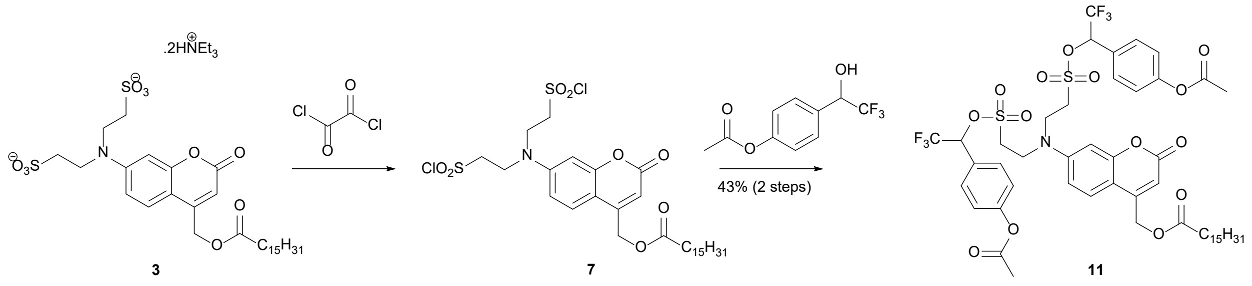

In an analogous manner to the synthesis of **10** via **6** from **2**, the sulfocoumarin palmitate **3** (10 mg, 12 μmol) was converted to the bissulfonyl chloride **7** (observed 681 $[M+H]^+$), and subsequently to the protected sulfocoumarin **11** (5 mg, 4.6 μmol).

Yield: 43% over two steps.

^{1}H NMR (400 MHz, $CDCl_3$): δ = 7.48 (two diastereomers observed, d, 4H, $^3J_{HH}$ = 6Hz, $CF_3CHCC\underline{H}$), 7.38 (d, 1H, $^3J_{HH}$ = 9Hz, H-5), 7.18 (two diastereomers observed, d, 4H, $^3J_{HH}$ = 6Hz, C(=O)OCCH), 6.46 (unresolved dt, 1H, H-6), 6.34 (unresolved t, 1H, H-8), 6.30 (br s, 1H, H-3), 5.77 (q, 2H, $^3J_{HF}$ = 6Hz, $CHCF_3$), 5.25 (s, 2H, CH_2O), 3.79 – 3.71 (two diastereomers observed, m, 4H, NCH_2), 3.28 – 3.20 (two diastereomers observed, m, 4H, CH_2S), 2.46 (t, j = 8Hz, 2H, $C{=}OCH_2$), 2.32 (two diastereomers observed, s, 6H, $C{=}OCH_3$), 1.68 (m, 2H), 1.33 – 1.2 (m, 26H), 0.87 (t, j = 7Hz, 3H, $CH_2C\underline{H}_3$).

$^{13}C\{^1H\}$ NMR (101 MHz, $CDCl_3$): δ = 173.3 ($\underline{C}{=}OCH_3$), 169.3 ($\underline{C}{=}OCH_2$), 158.8 (C-2), 155.9, 152.8, 147.9 (C-4), 129.7, 126.9 (C-5), 125.46, 122.1 (q, $^1J_{CF}$ = 281 Hz, CF_3), 109.3 (C-3), 108.9, 99.6 (C-8), 77.6 (q, $^2J_{CF}$ = 35 Hz, $\underline{C}HCF_3$), 61.0 (CH_2O), 49.2 (CH_2S), 46.1 (NCH_2), 34.2 ($C{=}O\underline{C}H_2$), 32.1, 29.8, 29.8, 29.7, 29.6, 29.5, 29.4, 29.3, 25.0, 22.8, 21.2 ($C{=}O\underline{C}H_3$), 14.3 ($CH_2\underline{C}H_3$).

^{19}F NMR ($CDCl_3$): δ = -75.7 (two diastereomers observed, d, $^3J_{FH}$ = 2.5 Hz).

UV (EtOH): λ_{max} = 356 nm, ε = 35000 $Lmol^{-1}cm^{-1}$

HRMS: Chemical Formula: $C_{50}H_{61}F_6NNaO_{14}S_2$ $[M+Na]^+$

Exact Mass: Calculated 1100.3335 Found 1100.3348

(7-*N,N*-Bis(2-sulfonylethyl)amino)-2-oxo-2H-chromen-4-yl)methylene decyl carbonate (**4**)

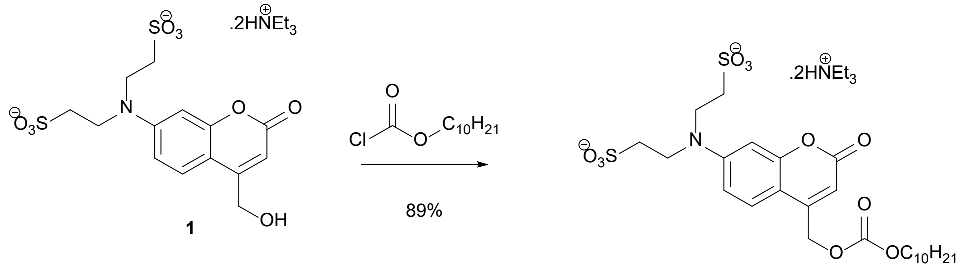

4

A solution of **1** (170 mg, 0.28 mmol) in $CHCl_3$ (1 mL), MeCN (1 mL) and pyridine (1 mL) was evaporated at 23 °C/0.004 mbar. Under an argon atmosphere, DMAP (20 mg, 0.16 mmol), anhydrous DCM (2 mL) and dry pyridine (100 µL, 1.26 mmol) were added. With cooling in an ice bath, neat decyl chloroformate (153 µL, 0.66 mmol) was added. After 15 min the cooling bath was removed and stirring continued at 23 °C for 3 h. MeOH (300 µL) was added with cooling in an ice bath. After 30 min volatiles were removed at 0.004 mbar. The crude product was purified by reversed-phase chromatography (LiChroprep RP18, 50 mM TEAB, 40→90% MeOH) to yield **4**. Yield: 197 mg (89%) foam.

^1H NMR (400 MHz, $CDCl_3$) δ = 9.18 (s, 2H, Et_3NH^+), 7.23 (d, $^3J_{HH}$ = 9Hz, 1H, H-5), 6.69 (dd, $^3J_{HH}$ = 9Hz, $^4J_{HH}$ = 2Hz, 1H, H-6), 6.62 (d, $^4J_{HH}$ = 2Hz, 1H, H-8), 6.04 (s, 1H, H-3), 5.16 (s, 2H, 4-CH_2O), 4.08 (t, $^3J_{HH}$ = 7Hz, 2H, OCH_2), 3.89 – 3.68 (m, 4H, CH_2), 3.10 (q, $^3J_{HH}$ = 7Hz, 12H, $N(C\underline{H}_2CH_3)_3$), 3.03 – 2.87 (m, 4H, CH_2), 1.66 – 1.53 (m, 2H, $OCH_2C\underline{H}_2CH_2$), 1.29 (t, $^3J_{HH}$ = 7Hz, 18H, 2 x $N(CH_2C\underline{H}_3)_3$), 1.15 (s, 13H), 0.76 (t, $^3J_{HH}$ = 7Hz, 3H, CH_2CH_3).

^{13}C NMR (101 MHz, $CDCl_3$) δ = 161.39, 155.99, 154.61, 150.24, 149.11, 124.68, 109.27, 106.92, 106.48, 98.19, 77.63, 77.31, 76.99, 68.89, 64.29, 48.02, 47.41, 46.37, 31.74, 29.37, 29.34, 29.15, 29.07, 28.48, 25.52, 22.55, 14.03, 8.80.

HRMS: Chemical Formula: $C_{25}H_{35}NO_{11}S_2$ $[M-2H]^{2-}$

Exact Mass: Calculated 294.5831 Found 294.5832

UV (MOPS buffer): λ_{max} = 377 nm, ε = 15000 $Lmol^{-1}cm^{-1}$

(7-*N,N*-Bis(2-chlorosulfonylethyl)amino)-2-oxo-2H-chromen-4-yl)methylene decyl carbonate (**8**) and (7-*N,N*-bis(2-(1-(4-acetoxyphenyl)-2,2,2-trifluoroethyl)sulfonylethyl)amino-2-oxo-2H-chromen-4-yl)methylene decyl carbonate (**12**)

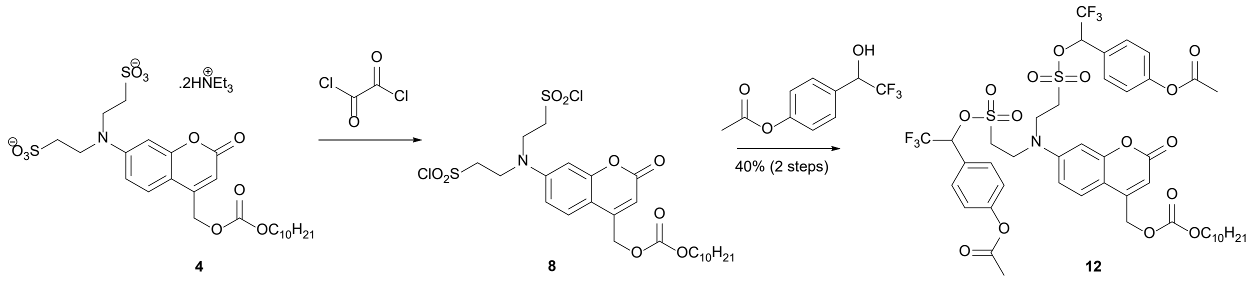

In a Schlenk tube equipped with a stirrer bar, 27 mg of sticky brown **4** (34 μmol) was put under Ar and DCM (2 mL) was added. The solution was cooled to 0ºC with stirring, followed by addition of oxalyl chloride (8.5 μL, 100 umol). DMF (1 drop) was then added. The mixture was stirred and allowed to warm to room temperature. After approximately 20 minutes at RT the orange solution was put under vacuum for 2 hours to remove all volatile components (including unreacted oxalyl chloride) to yield **8**, which was characterised by mass spectrometry (observed 628 $[M+H]^+$).

The resulting solid was redissolved in DCM (2 mL). Into a separate flask were weighed the alcohol[3] (25 mg, 106 μmol) and DABCO (20 mg, 180 μmol). These were dissolved in DCM (3 mL) and the solution was transferred to the flask containing **8** via cannula. The resulting orange solution was stirred at room temperature for one hour. The solvent was subsequently removed under dynamic vacuum and the product was purified by silica gel chromatography (98:2 DCM: MeOH). The first fraction (R_f 0.6, fractions 11-13) was then subjected to preparative HPLC (C18, 70-100% MeCN, 25 min) resulting in collection of one main fraction at 18 minutes containing compound **12**. Yield after purification: 13 mg (13 μmol, 40%). Note that the compound exists as a mixture of two diastereomers with very similar NMR spectra; the ^{19}F, phenyl ring, ethylene chain and acetate resonances were observed separately for the diastereomers.

^{1}H NMR (400 MHz, $CDCl_3$): δ = 7.49 (two diastereomers observed, d, 4H, $^3J_{HH}$ = 9Hz, $CF_3CHCC\underline{H}$), 7.37 (dd, 1H, $^3J_{HH}$ = 9Hz, $^5J_{HH}$ = 1Hz, H-5), 7.18 (two diastereomers observed, d, 4H, $^3J_{HH}$ = 9Hz, C(=O)OCCH), 6.45 (unresolved dt, 1H, $^3J_{HH}$ = 9Hz, H-6), 6.33 (br s, 2H, H-8 and H-3), 5.78 (q, 2H, $^3J_{HF}$ = 6Hz, $CHCF_3$), 5.28 (s, 2H, 4-CH_2O), 4.20 (t, 2H, $^3J_{HH}$ = 7Hz,

OCH_2) 3.79-3.70 (two diastereomers observed, m, 4H, NCH_2), 3.30-3.19 (two diastereomers observed, m, 4H, CH_2S), 2.31 (two diastereomers observed, s, 6H, C=OCH_3), 1.69 (p, 2H, $^3J_{HH}$ = 7Hz, $OCH_2\underline{CH_2}CH_2$), 1.40 – 1.20 (br m, 14H, decyl), 0.88 (t, 3H, $^3J_{HH}$ = 7Hz, $CH_2C\underline{H}_3$).
$^{13}C\{^1H\}$ NMR (101 MHz, $CDCl_3$): δ = 169.3 ($\underline{C}$=OCH_3), 161.4 (C-2), 155.9, 154.9, 152.8, 152.7, 149.1 (C-4), 147.9, 129.7 ($CF_3CHC\underline{C}H$), 126.9, 125.6 (C-5), 122.8 (C(=O)OC$\underline{C}$H), 122.2 (q, $^1J_{CF}$ = 281 Hz, CF_3), 109.3 (C-6), 109.2 (C-3), 108.7, 99.6 (C-8), 77.6 (q, $^2J_{CF}$ = 27 Hz, $\underline{C}HCF_3$), 69.3 (OCH_2), 64.3 (4-CH_2O), 49.2 (CH_2S), 46.1 (NCH_2), 32.0, 29.7, 29.6, 29.4, 29.3, 28.7 ($OCH_2\underline{C}H_2CH_2$), 25.8, 22.8, 21.2 (C=O$\underline{C}H_3$), 14.2 ($CH_2\underline{C}H_3$).
^{19}F NMR ($CDCl_3$): δ = -75.70 (two diastereomers observed, d, $^3J_{FH}$ = 4Hz).
UV (EtOH): λ_{max} = 356 nm, ε = 15000 $Lmol^{-1}cm^{-1}$
HRMS: Chemical Formula: $C_{45}H_{51}F_6NNaO_{15}S_2$ $[M+Na]^+$
Exact Mass: Calculated 1046.2502 Found 1046.2496

(7-*N,N*-Bis(2-sulfonylethyl)amino)-2-oxo-2H-chromen-4-yl)methanol (**5**) and (7-*N,N*-bis(2-(1-(4-acetoxyphenyl)-2,2,2-trifluoroethyl)sulfonylethyl)amino-2-oxo-2H-chromen-4-yl)methanol (**9**)

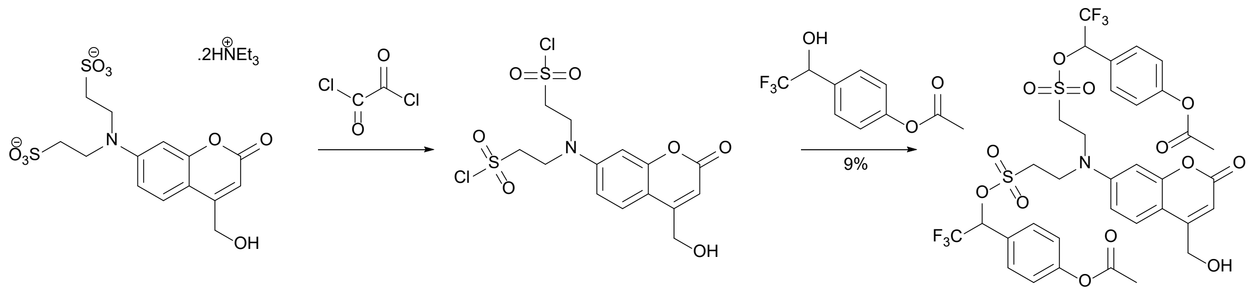

1 **5** **9**

In a Schlenk tube equipped with a stirrer bar, 100 mg of the sticky brown triethylammonium salt of coumarin sulfonate **1** (0.164 mmol) was put under Ar and DCM (5 mL) was added. The suspension was cooled to 0ºC with stirring. Subsequently oxalyl chloride (0.1 mL) was added and the colour became darker. DMF (1 drop) was then added, following which a pale orange precipitate formed. The mixture was stirred and allowed to warm to room temperature. After approximately 20 minutes at RT there was no more precipitate present and the pale orange solution was placed under vacuum for 2 hours to remove all volatile components (including unreacted oxalyl chloride) to yield **5** as a solid, which was used without further characterization.

Into a separate Schlenk flask were weighed the benzyl alcohol[3] (70 mg, 0.3 mmol) and DABCO (45 mg, 0.4 mmol). These were dissolved in DCM (5 mL) and the colourless solution was transferred to a flask containing **5** dissolved in 4 mL DCM via cannula. The resulting dark brown solution was stirred at room temperature for one hour. The solvent was removed under dynamic vacuum and the product was purified by silica gel chromatography (96:4 DCM: MeOH). The first fraction (R_f 0.3, fractions 16-24) was then subjected to preparative HPLC (C18, 60% MeCN) resulting in collection of two fractions. The first was unreacted alcohol and the second was compound **9**. Yield after purification: 12 mg (0.014 mmol, 9%). Note that the compound exists as a mixture of two diastereomers with very similar NMR spectra; the ^{19}F, phenyl ring, ethylene chain and acetate resonances were observed separately for the diastereomers.

^{1}H NMR (400 MHz, $CDCl_3$): δ = 7.46 (two diastereomers observed, d, 4H, $^{3}J_{HH}$ = 6Hz, $CF_3CHCC\underline{H}$), 7.38 (dd, 1H, $^{3}J_{HH}$ = 9Hz, $^{5}J_{HH}$ = 1Hz, H-5), 7.17 (two diastereomers observed, d, 4H, $^{3}J_{HH}$ = 6Hz, C(=O)OCCH), 6.44 (dt, 1H, $^{3}J_{HH}$ = 9Hz, $^{5}J_{HH}$ = 2Hz, H-6), 6.39 (s, 1H, H-3), 6.32 (unresolved dd, 1H, $^{4}J_{HH}$ = 2Hz, H-8), 5.77 (q, 2H, $^{3}J_{HF}$ = 6Hz, $CHCF_3$), 4.83 (s, 2H, CH_2OH), 3.75 (m, 4H, NCH_2), 3.24 (m, 4H, CH_2S), 2.31 (two diastereomers observed, s, 6H, CH_3).

$^{13}C\{^{1}H\}$ NMR (101 MHz, $CDCl_3$ CH_3): δ = 169.2 ($C{=}OCH_3$), 161.6 ($C{=}OCH_2$), 155.8, 154.3 (CCH_2), 152.7, 147.6 (C-7), 129.6, 126.9, 125.5 (C-5), 122.8, 122.1 (q, $^{1}J_{CF}$ = 282 Hz, CF_3), 109.1 (C-6), 108.2 (C-3), 99.6 (C-8), 77.4 (q, $^{2}J_{CF}$ = 50 Hz, $\underline{C}HCF_3$), 60.9 (CH_2OH), 49.2 (CH_2S), 46.1 (NCH_2), 21.2 (CH_3).

^{19}F NMR (376 MHz, $CDCl_3$): δ = -75.7 (two diastereomers observed, d, $^{3}J_{FH}$ = 6Hz).

HRMS: Chemical Formula: $C_{34}H_{32}F_6NO_{13}S_2$ $[M+H]^+$

Exact Mass: Calculated 840.12193 Found 840.12274

Chemical Formula: $C_{34}H_{31}F_6KNO_{13}S_2$ $[M+K]^+$

Exact Mass: Calculated 878.07781 Found 878.07722

UV (EtOH): λ_{max} = 355 nm, ε = 23000 $Lmol^{-1}cm^{-1}$

Synthesis of (7-*N,N*-bis(2-(1-(4-acetoxyphenyl)-2,2,2-trifluoroethyl)sulfonylethyl)amino-2-oxo-2H-chromen-4-yl)methylene arachidonate (**10**): alternative route.

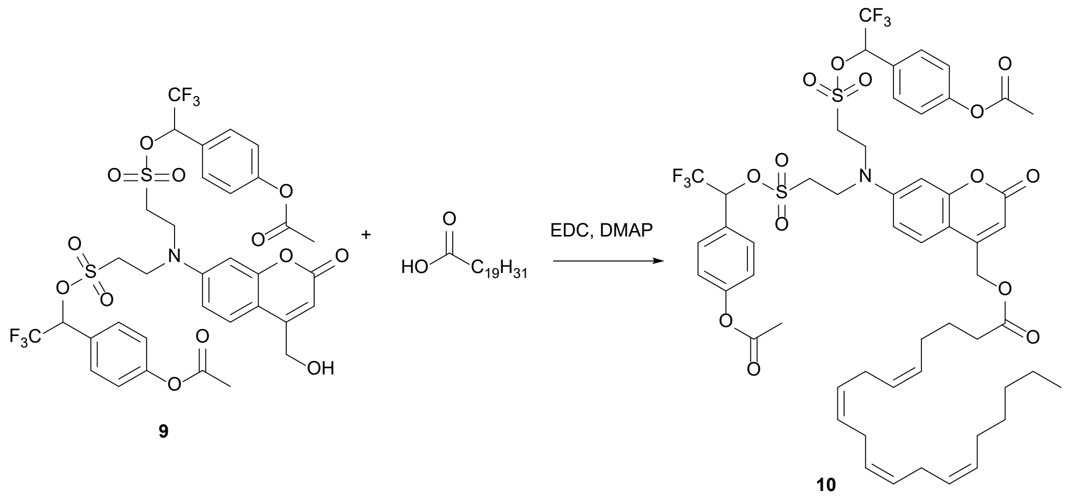

Arachidonic acid (4 mg, 0.013 mmol) was combined with DMAP (1.4 mg, 0.012 mmol) in a Schlenk flask equipped with a magnetic stirrer bar, placed under Ar and dissolved in dry DCM. In a separate Schlenk flask, EDC.HCl (4.6 mg, 0.024 mmol) was also put under Ar and dissolved in DCM. The EDC solution was added to the AA solution and stirred for approximately 3 minutes. Compound **9** (10 mg, 0.012 mmol) in DCM was then added and the mixture was stirred for two hours at room temperature. The reaction was quenched with a water/ethyl acetate mixture. After extraction into ethyl acetate and washing with water and brine, the organic fraction was dried over sodium sulfate and the solvent was removed under vacuum. Purification by flash chromatography (96:4 DCM:methanol solvent mixture) led to collection of a pale yellow fraction which was contaminated with free arachidonic acid. This fraction was subjected to preparative HPLC (isocratic, 90% MeCN) to obtain pure **10**. The NMR spectrum was superimposable with that of the **10** obtained via **2.** Yield after purification: 4 mg (0.004 mmol, 30%).

(7-*N,N*-Bis(2-(1-(4-acetoxyphenyl)-2,2,2-trifluoroethyl)sulfonylethyl)amino-2-oxo-2H-chromen-4-yl)methylene butyrate (**13**)

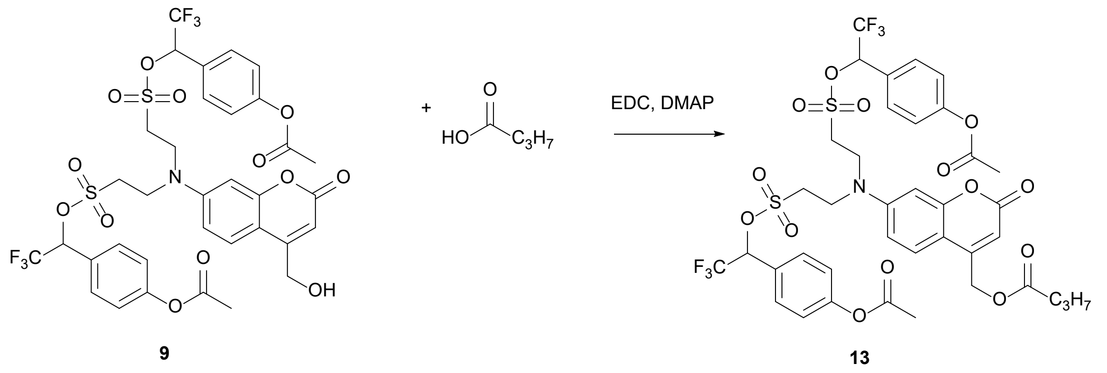

An analogous procedure to the synthesis of **10** from **9** was followed using butyric acid to yield **13.** Yield after purification: 35%. Note that the compound exists as a mixture of two diastereomers with very similar NMR spectra; the ^{19}F, phenyl ring, ethylene chain and acetate resonances were observed separately for the diastereomers.

^{1}H NMR (400 MHz, $CDCl_3$): δ = 7.48 (two diastereomers observed, d, 4H, $^{3}J_{HH}$ = 6Hz, $CF_3CHCC\underline{H}$), 7.36 (d, 1H, $^{3}J_{HH}$ = 9Hz, H-5), 7.18 (two diastereomers observed, d, 4H, $^{3}J_{HH}$ = 6Hz, C(=O)OCCH), 6.44 (unresolved dt, 1H, $^{3}J_{HH}$ = 9Hz, H-6), 6.33 (br s, 1H, H-8), 6.27 (br s, 1H, H-3), 5.77 (q, 2H, $^{3}J_{HF}$ = 6Hz, $CHCF_3$), 5.24 (s, 2H, CH_2O), 3.75 (two diastereomers observed, m, 4H, NCH_2), 3.24 (two diastereomers observed, m, 4H, CH_2S), 2.44 (t, 2H, $^{3}J_{HH}$ = 7Hz, $C{=}OCH_2$), 2.32 (two diastereomers observed, s, 6H, $C{=}OCH_3$), 1.73 (m, 2H, $CH_2C\underline{H}_2CH_3$), 0.99 (t, 3H, $^{3}J_{HH}$ = 7Hz, $CH_2C\underline{H}_3$).

^{19}F NMR ($CDCl_3$): δ = -75.7 (two diastereomers observed, d, $^{3}J_{FH}$ = 4Hz).

HRMS: Chemical Formula: $C_{38}H_{37}F_6NNaO_{14}S_2$ $[M+Na]^+$

Exact Mass: Calculated 932.1457 Found 932.1455

Bis-N,N-(2-ethylphosphonic acid)(3-hydroxyphenyl)amine tetraethyl ester (**15**) and (2-ethylphosphonic acid)(3-hydroxyphenyl)amine diethyl ester (**22**)

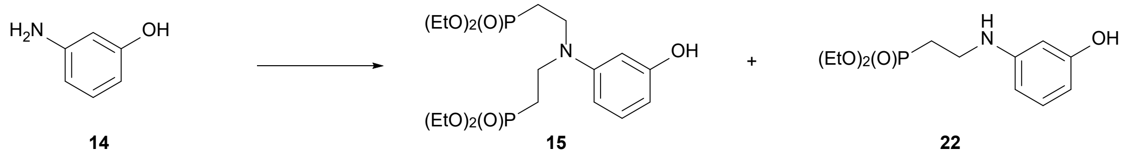

A solution of 3-aminophenol 14 (10.9 g, 100 mmol) and diethyl 2-bromoethylphosphonate (50 g, 204 mmol) in EtOH (40 mL) was stirred at 60 °C for 1 h. Saturated aqueous NaHCO3 (140) was added under reflux at such a rate as to maintain the pH below 7 (4 h). After stirring at 90 °C for 16 h the reaction was briefly refluxed with addition of saturated aqueous NaHCO3 solution (50

mL). The mixture was cooled to room temperature and extracted three times with EtOAc (3 x 75 mL). The combined extracts were washed with brine (100 mL) and volatiles were removed under reduced pressure. The oil obtained (42 g) was purified by chromatography (EtOAc:cyclohexane 8:2).

Yield: 17.7 g amber oil (mixture of **15** with monoalkylated byproduct **22**)

Yield: 4.1 g (9%) colorless oil, pure **15**

^{1}H NMR (400 MHz, $CDCl_3$): δ = 8.41 (s, 1H, OH), 7.04 (t, *J*=8Hz, 1H, H-5), 6.31 (d, *J*=8.0Hz, 1H), 6.27 (s, 1H, H-2), 6.20 (d, *J*=8Hz, 1H), 4.19 – 4.02 (m, 8H, 4 x OCH_2), 3.65-3.49 (m, 4H, 2 x NH_2), 2.06 (dt, *J*=16Hz, 9Hz, 4H, 2 x PCH_2), 1.32 (t, *J*=7Hz, 12H, 4 x CH_3).

^{13}C{^{1}H} NMR (101 MHz, $CDCl_3$): δ = 158.47, 147.43, 130.28, 104.78, 104.37, 100.16, 77.42, 77.10, 76.78, 61.88 (d, J_{CP}=7Hz, 4 x OCH_2), 44.84 (s, 2 x NCH_2), 24.84 (d, J_{CP}=137Hz, 2 x PCH_2), 16.42 (d, J_{CP}=6Hz, 4 x CH_3).

^{31}P{^{1}H} NMR (162 MHz, $CDCl_3$): δ = 29.41.

HRMS: Chemical Formula: $C_{18}H_{34}NO_7P_2$ $[M+H]^+$

Exact Mass: Calculated 438.1805 Found 438.1806

Chemical Formula: $C_{18}H_{33}NNaO_7P_2$ $[M+Na]^+$

Exact Mass: Calculated 460.1624 Found 460.1625

Byproduct **22**

Yield: 25%

^{1}H NMR (400 MHz, $CDCl_3$): δ = 8.05 (bs, 1H, NH), 7.00 (t, *J*=8Hz, 1H, H-5), 6.29 (dd, *J*=8Hz, 2Hz, 1H), 6.18 (t, *J*=2Hz, 1H, H-2), 6.14 (m, 1H), 4.19-4.04 (m, 4H, 2 x OCH_2), 3.41 (dt, *J*=16Hz, 7Hz, 2H, NCH_2), 2.07 (dt, *J*=18Hz, 7Hz, 2H, PCH_2), 1.32 (t, *J*=7Hz, 6H).

^{31}P{^{1}H} NMR (162 MHz, $CDCl_3$): δ = 30.38.

^{13}C{^{1}H} NMR (101 MHz, $CDCl_3$): δ = 157.98, 148.73, 130.16, 105.42, 105.18, 100.35, 77.42, 77.10, 76.78, 62.13 (d, J_{CP}=6.6, 2 x OCH_2), 37.70 (d, J_{CP}=3.8, NCH_2), 25.46 (d, *J*=139.1, PCH_2), 16.40 (d, J_{CP}=6.1, 2 x CH_3).

HRMS: Chemical Formula: $C_{12}H_{21}NO_4P$ $[M+H]^+$

Exact Mass: Calculated 274.1208 Found 274.1204

Bis-N,N-(2-ethylphosphonic acid)(2-oxo-2*H*-chromen-4-methyl-7-yl)amine tetraethyl

ester (**16**)

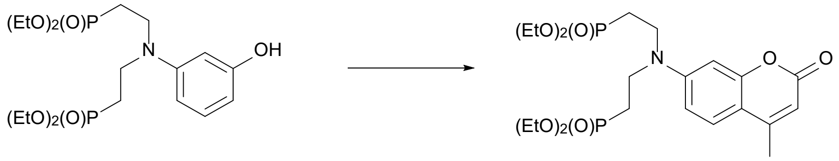

15 **16**

A solution of **15** (4.1 g, 9.4 mmol), zinc chloride (0.72 g, 5.3 mmol), absolute ethanol (10 mL) and ethyl acetoacetate (1.42 mL, 11.3 mmol) was stirred under reflux for 21 h under an argon atmosphere. The reaction was concentrated at ambient pressure, diluted with water (30 mL), extracted three times with EtOAc (3 x 50 mL), washed with brine and evaporated under reduced pressure. The residue obtained was purified on a column of silica gel 60 with EtOAc:MeOH (1:0 → 4:1) to afford **16**. Most of the starting material (3.1 g) was recovered. Yield: 450 mg (9%) amber oil, R_f EtOAc:MeOH 9:1 = 0.13

^{1}H NMR (400 MHz, $CDCl_3$): δ = 7.28 (d, *J*=9Hz, 1H, H-5), 6.49 (d, *J*=8Hz, 1H, H-6), 6.33 (s, 1H, H-8), 5.80 (s, 1H, H-3), 4.06 – 3.90 (m, 8H, 4 x OCH_2), 3.61 – 3.47 (m, 4H, 2 x NCH_2), 2.18 (s, 3H, 4-CH_3), 2.03 – 1.85 (m, 4H, 2 x PCH_2), 1.19 (t, *J*=7Hz, 12H, 4 x $CH_2C\underline{H}_3$).

$^{31}P\{^1H\}$ NMR (162 MHz, $CDCl_3$): δ = 27.96.

$^{13}C\{^1H\}$ NMR (101 MHz, $CDCl_3$): δ = 161.33 (s, CO), 155.51, 152.46, 148.80, 125.73, 109.98, 109.49, 108.48, 98.03, 77.48, 77.16, 76.84, 61.68 (d, J_{CP}=6.6, 4 x OCH_2), 44.60 (s, 2 x NCH_2), 23.52 (d, J_{CP} = 136.0, 2 x PCH_2), 18.12 (s, 4-CH_3), 16.17 (d, J_{CP} = 5.9, 4 x $CH_2\underline{C}H_3$).

HRMS: Chemical Formula: $C_{22}H_{36}NO_8P_2$ $[M+H]^+$

Exact Mass: Calculated 504.1911 Found 504.1915

Bis-N,N-(2-ethylphosphonic acid)(2-oxo-2*H*-chromen-4-methyl-7-yl)amine (**36**)

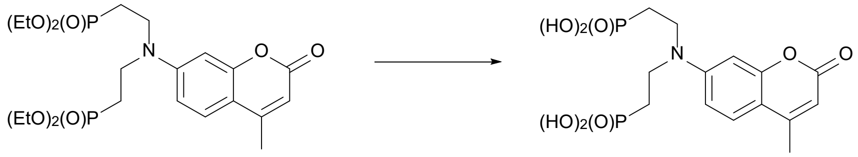

16 **36**

To **16** (450 mg, 0.89 mmol) was added bromotrimethylsilane (2.5 mL, 18.9 mmol) via syringe under argon. After stirring for 20 h at 23°C, volatiles were removed at 23°C/0.04 mbar. The greenish oil obtained was dissolved in MeOH (30 mL), evaporated under reduced pressure and

the foam obtained was dried at 23°C/0.04 mbar. This process was repeated two times. Finally, compound **36** was obtained as a tan solid. Yield: 349 mg (99%)

mp 211-212°C (dec.)

^{1}H NMR (400 MHz, MeOD): δ = 7.65 (d, *J*=9Hz, 1H, H-5), 6.88 (dd, *J*=9Hz, 2Hz, 1H, H-6), 6.69 (d, *J*=2Hz, 1H, H-8), 6.04 (s, 1H, H-3), 3.78 (dd, J=16Hz, 8Hz, 4H, 2 x NCH_2), 2.42 (s, 3H, CH_3), 2.21 – 2.05 (m, 4H, 2 x PCH_2).

^{31}P{^{1}H} NMR (162 MHz, MeOD): δ = 25.87.

^{13}C{^{1}H} NMR (101 MHz, MeOD): δ = 164.07, 156.95, 155.92, 150.42, 127.62, 112.19, 111.24, 110.17, 100.06, 49.85, 49.64, 49.43, 49.21, 49.00, 48.79, 48.57, 48.36, 46.92 (s, 2 x NCH_2), 25.93 (d, J_{CP} = 133Hz, 2 x PCH_2), 18.50 (s, 4-CH_3).

HRMS: Chemical Formula: $C_{14}H_{18}NO_8P_2^-$

Exact Mass: Calculated 390.0513 Found 390.0515

Bis-N,N-(2-ethylphosphonic acid)(2-oxo-2*H*-chromen-4-methyl-7-yl)amine tetrakis(acetoxymethyl) ester (**17**)

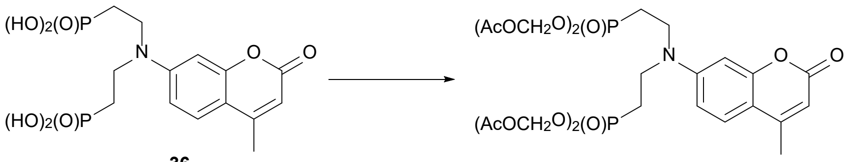

36 **17**

To **36** (320 mg, 0.82 mmol) was added dry MeCN (3 mL), dry diisopropylethylamine (1.7 mL, 9.8 mmol) and bromomethyl acetate (0.8 mL, 8.2 mmol) under an argon atmosphere. After stirring at 22 °C for 6 h volatiles were removed at 22 °C/0.04 mbar. The crude was partitioned between EtOAc (30 mL) and water (20 mL), the organic phase was separated and dried (Na_2SO_4). The crude **17** was purified by chromatography (EtOAc:MeOH (1:0 → 9:1). Yield: 213 mg (38%) colourless oil. R_f EtOAc:MeOH 9:1 = 0.34

^{1}H NMR (400 MHz, $CDCl_3$): δ = 7.38 (d, *J*=9Hz, 1H, H-5), 6.54 (d, *J*=9Hz, 1H, H-6), 6.41 (s, 1H, H-8), 5.92 (s, 1H, H-3), 5.73-5.49 (m, 8H, 4 x OCH_2), 3.71-3.53 (m, 4H, 2 x NCH_2), 2.28 (s, 3H, 4-CH3), 2.21 – 2.00 (m, 4H, 2 x PCH_2) , 2.08 (s, 12H, 4 x $COCH_3$).

^{31}P{^{1}H} NMR (162 MHz, $CDCl_3$): δ = 28.58.

$^{13}C\{^1H\}$ NMR (101 MHz, $CDCl_3$): δ = 169.29 (<u>C</u>OCH$_3$), 161.33, 155.63, 152.49, 148.64, 125.99, 110.54, 110.10, 108.70, 98.53, 81.14 (d, J_{CP} = 6Hz, 4 x OCH_2), 77.48, 77.16, 76.84, 44.29 (s, 2 x NCH_2), 24.35 (d, J_{CP} = 136Hz, 2 x PCH_2), 20.58 (s, CO<u>C</u>H$_3$), 18.31 (s, 4-CH_3).
HRMS: Chemical Formula: $C_{26}H_{35}NNaO_{16}P_2^+$ (Na salt)
Exact Mass: Calculated 702.1323 Found 702.1332

Bis-N,N-(2-ethylphosphonic acid)(3-hydroxy-4-formylphenyl)amine tetraethyl ester (**18**)

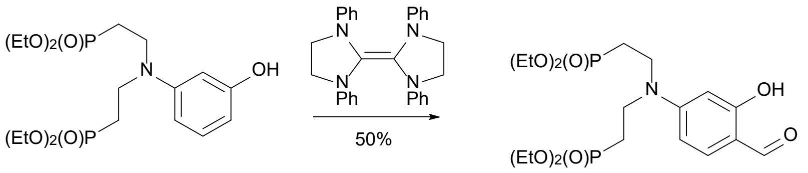

To **15** (9.2 mmol) were added bis(1,3-diphenylimidazolidinylidene)[4, 5] (4.68 g, 10.5 mmol) and dry DMF (5 mL) under an argon atmosphere. The mixture was heated to gentle reflux for 1 h. Volatiles were distilled off at 50 °C/0.2 mbar. After cooling to room temperature the remaining dark tar was hydrolyzed by addition of 1 N HCl (80 mL) and 37% HCl (10 mL, 125 mmol) and allowed to stand overnight. After additional stirring for 1 h solids were removed by filtration, washed with MeOH (50 mL) and the dark blue filtrate was evaporated under reduced pressure. The dark tar obtained was treated with phosphate buffer (100 ml) and brought to a pH of 5-6 by adding 1.1 M $NaHCO_3$ solution. The mixture was extracted four times with EtOAc (4 x 100 mL), the extracts were dried (Na_2SO_4) and evaporated under reduced pressure to give a dark tar (9.2 g) that was purified by chromatography with EtOAc:MeOH (1:0 → 9:1). Yield: 4.88 g (50%), t_R 70% MeOH = 4.9 min, R_f EtOAc:MeOH 9:1 = 0.2
^1H NMR (400 MHz, $CDCl_3$): δ = 11.53 (s, 1H, OH), 9.55 (s, 1H, CHO), 7.34 (d, J=8.8, 1H), 6.29 (dd, J=8.8, 2.4, 1H, H-6), 6.09 (d, J=2.4, 1H, H-8), 4.31 – 4.00 (m, 8H, 4 x OCH_2), 3.74 – 3.59 (m, 4H, 2 x NCH_2), 2.15 – 1.99 (m, 4H, 2 x PCH_2), 1.35 (t, J=7.1, 12H, 4 x CH_3).
$^{31}P\{^1H\}$ NMR (162 MHz, $CDCl_3$): δ = 27.61.
$^{13}C\{^1H\}$ NMR (101 MHz, $CDCl_3$): δ = 192.83 (CO), 164.43, 153.03, 135.80, 112.43, 104.53, 97.70, 77.48, 77.16, 76.84, 62.05 (d, J_{CP}=7Hz, 4 x OCH_2), 45.06 (s, 2 x NCH_2), 24.20 (d, J_{CP}=137Hz, 2 x PCH_2), 16.56 (d, J_{CP}=6Hz, 4 x CH_3).
HRMS: Chemical Formula: $C_{38}H_{66}N_2NaO_{16}P_4^+$ $[2M+Na]^+$
Exact Mass: Calculated 953.3255 Found 953.3256

Bis-N,N-(2-ethylphosphonic acid)(2-oxo-2*H*-chromen-3-nitro-7-yl)amine tetraethyl ester (**19**)

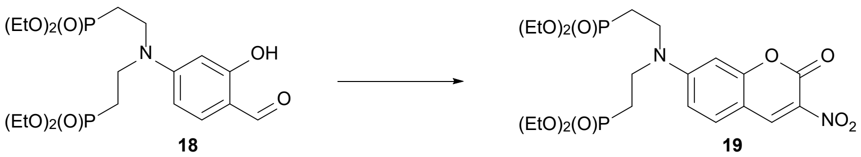

A mixture of **18** (4.88 g, 10.5 mmol), molecular sieves 3 Å (150 mg), AcOH (0.3 mL, 5.2 mmol), piperidine (0.1 mL, 1.5 mmol), n-BuOH (20 mL) and ethyl nitroacetate (1.28 mL, 11.6 mmol) was refluxed for 2 h under an argon atmosphere before more ethyl nitroacetate (0.5 mL, 4.5 mmol) was added. After 22 h the dark reaction was cooled to room temperature and volatiles were removed under reduced pressure to give a dark tar (8 g) that was purified by chromatography in two runs (A: EtOAc:MeOH 1:0 → 9:1; B: EtOAc:MeOH 9:1). After removal of solvent under reduced pressure the semi-solid residue obtained was triturated with EtOAc to give a brown solid. Yield: 1.5 g (27%), t_R 70% MeOH = 3.7 min, R_f EtOAc:MeOH 9:1 = 0.19. mp: 111 – 112.5°C

^{1}H NMR (400 MHz, $CDCl_3$): δ = 8.66 (s, 1H, H-4), 7.48 (d, *J*=9Hz, 1H, H-5), 6.71 (dd, *J*=9Hz, 2Hz, 1H, H-6), 6.45 (d, *J*=2Hz, 1H, H-8), 4.18 – 3.99 (m, 8H, 4 x OCH_2), 3.80 – 3.63 (m, 4H, 2 x NCH_2), 2.16 – 1.98 (m, 4H, 2 x CH_2P), 1.30 (t, J=7Hz, 12H, 4 x CH_3).

^{31}P{^{1}H} NMR (162 MHz, $CDCl_3$): δ = 26.87.

^{13}C{^{1}H} NMR (101 MHz, $CDCl_3$): δ = 158.35, 153.49, 152.93, 143.41, 132.84, 128.00, 111.17, 106.81, 97.39, 77.50, 77.18, 76.87, 62.10 (d, J_{CP}=7Hz, OCH_2), 45.39 (NCH_2) 23.92 (d, J_{CP}=138Hz, PCH_2), 16.45 (d, J_{CP}=6Hz, CH_3).

HRMS: Chemical Formula: $C_{21}H_{32}N_2NaO_{10}P_2^+$ $[M+Na]^+$

Exact Mass: Calculated 557.1424 Found 557.1442

Bis-N,N-(2-ethylphosphonic acid)(3-amino-2-oxo-2*H*-chromen-7-yl)amine tetraethyl ester (**37**)

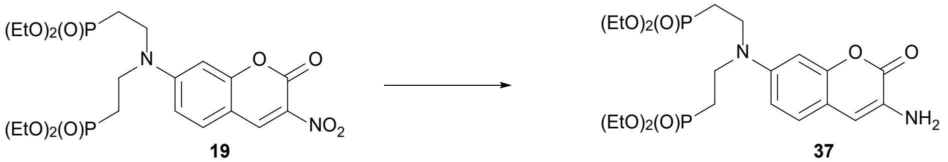

To a stirring mixture of 37% HCl (16 mL, 192 mmol) and tin chloride (6.27 g, 33.1 mmol) was added **19** (1.11 g, 2.08 mmol) over the course of 30 min. The orange mixture was stirred for 4 h

at 23 °C. The reaction was poured onto crushed ice (50 mL) and with cooling in an ice bath aqueous NaOH (20%, 300 mmol) was added portion wise until a pH of 7. Solids were removed by filtration, washed with brine (100 mL) and EtOAc (100 mL), the filtrate saturated with NaCl and extracted three times with EtOAc (3 x 75 mL). The combined organic layers were dried (Na_2SO_4), filtered and evaporated under reduced pressure to afford compound **37** as a red oil. Yield: 930 mg (89%), R_f EtOAc:MeOH 9:1 = 0.26.

^{1}H NMR (400 MHz, $CDCl_3$): δ = 8.66 (s, 1H, H-4), 7.48 (d, *J*=9Hz, 1H, H-5), 6.71 (dd, *J*=9Hz, 2Hz, 1H, H-6), 6.45 (d, *J*=2Hz, 1H, H-8), 4.18 – 3.99 (m, 8H, 4 x OCH_2), 3.78 – 3.66 (m, 4H, 2 x NCH_2), 2.16 – 1.98 (m, 4H, 2 x PCH_2), 1.30 (t, *J*=7Hz, 12H, 4 x CH_3).

^{31}P{^{1}H} NMR (162 MHz, $CDCl_3$): δ = 26.87.

^{13}C{^{1}H} NMR (101 MHz, $CDCl_3$): δ = 158.35, 153.49, 152.93, 143.41, 132.84, 128.00, 111.17, 106.81, 97.39, 77.50, 77.18, 76.87, 62.10 (d, J_{CP}=7Hz, CH_2O), 45.39 (NCH_2), 23.92 (d, J_{CP}=138Hz, PCH_2), 16.45 (d, J_{CP}=6Hz, CH_3).

HRMS: Chemical Formula: $C_{21}H_{34}N_2NaO_8P_2^+$ [M+Na]$^+$

Exact Mass: Calculated 527.1683 Found 527.1695

Bis-N,N-(2-ethylphosphonic acid)(3-azido-2-oxo-2*H*-chromen-7-yl)amine tetraethyl ester (**20**)

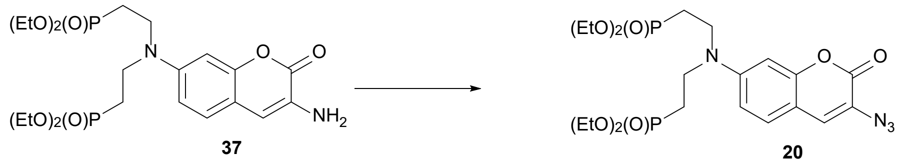

The crude amine **37** (860 mg, 1.7 mmol) was dissolved in 18% HCl (62.5 mmol). With cooling in an ice bath sodium nitrite (116 mg, 1.7 mmol) was added. The dark solution was stirred at 0-5 °C for 1 h. An ice cold solution of NaOAc (25 %, 62.5 mmol) was added to adjust the pH to 4. Sodium azide (222 mg, 3.4 mmol) was added and stirring continued at 0-5 °C for 5 h. The reaction was saturated with NaCl and extracted three times with EtOAc (3 x 50 mL). The combined extracts were evaporated under reduced pressure. The residue obtained was purified by chromatography (EtOAc:MeOH 9:1) to give **20**. Yield: 450 mg (50%), t_R (80% MeOH) = 4.3 min, R_f EtOAc:MeOH 9:1 = 0.26.

^{1}H NMR (400 MHz, $CDCl_3$): δ = 7.14 (d, *J*=9Hz, 1H, H-5), 6.98 (s, 1H, H-4), 6.52 (dd, *J*=9Hz, 2Hz, 1H, H-6), 6.39 (d, *J*=2Hz, 1H, H-8), 4.14 – 3.89 (m, 8H, 4 x OCH_2), 3.68 – 3.46 (m, 4H, 2 x NCH_2), 2.12 – 1.83 (m, 4H, 2 x PCH_2), 1.24 (t, *J*=7.1, 12H, 4 x CH_3).

$^{31}P\{^{1}H\}$ NMR (162 MHz, $CDCl_3$): δ = 27.87.

$^{13}C\{^{1}H\}$ NMR (101 MHz, $CDCl_3$): δ = 157.87, 153.72, 148.25, 128.51, 126.99, 120.59, 109.69, 109.14, 98.02, 77.61, 77.29, 76.97, 61.88 (d, J_{CP}=7Hz, $POCH_2$), 44.87 (s, NCH_2), 23.75 (d, J_{CP}=136Hz, PCH_2), 16.38 (d, J_{CP}= 6Hz, CH_3).

HRMS: Chemical Formula: $C_{21}H_{32}N_4NaO_8P_2^+$ $[M+Na]^+$

Exact Mass: Calculated 553.1588 Found 553.1592

Bis-N,N-(2-ethylphosphonic acid)(3-azido-2-oxo-2*H*-chromen-7-yl)amine (**38**)

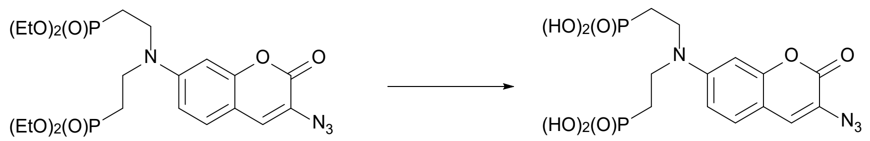

To dry **20** (430 mg, 0.81 mmol) was added bromotrimethylsilane (2.5 mL, 18.9 mmol) under an argon atmosphere. The resulting solution was stirred at 23 °C for 16 h protected from light. Volatiles were removed at 23 °C/0.06 mbar to obtain a dark foam. To this was added MeOH (30 mL) and volatiles were removed under reduced pressure. The brown foam obtained was dried at 23 °C/0.06 mbar to afford the desired compound **38**. Yield: 339 mg, t_R 50 mM TEAF: 80% MeOH = 1.7 min; 70% MeOH = 1.8 min; 60% MeOH = 2.2 min; 50% MeOH = 2.9 min.

^{1}H NMR (400 MHz, MeOD): δ = 7.40 (d, *J*=9Hz, 1H, H-5), 7.19 (s, 1H, H-4), 6.91 (d, *J*=9Hz, 1H, H-6), 6.78 (s, 1H, H-8), 3.86 – 3.66 (m, 2 x PCH_2), 2.25 – 2.03 (m, 4H, 2 x PCH_2).

$^{31}P\{^{1}H\}$ NMR (162 MHz, MeOD): δ = 25.64.

$^{13}C\{^{1}H\}$ NMR (101 MHz, MeOD): δ = 157.78, 153.24, 146.49, 146.46, 128.89, 126.88, 121.52, 111.65, 111.61, 100.19, 48.55, 48.35, 48.13, 47.92, 47.71, 47.49, 47.28, 47.07, 46.66, 45.00, 24.39 (d, J_{CP}=134Hz, PCH_2).

MS: Chemical Formula: $C_{13}H_{15}N_4O_8P_2^-$ $[M-H]^-$

Mass: Calculated 417.04 Found 417.0

Chemical Formula: $C_{26}H_{31}N_8O_{16}P_4^-$ $[2M-H]^-$

Exact Mass: Calculated 835.1 Found 835.5

Bis-N,N-(2-ethylphosphonic acid)(3-azido-2-oxo-2*H*-chromen-7-yl)amine tetrakis(acetoxymethylester) (**21**)

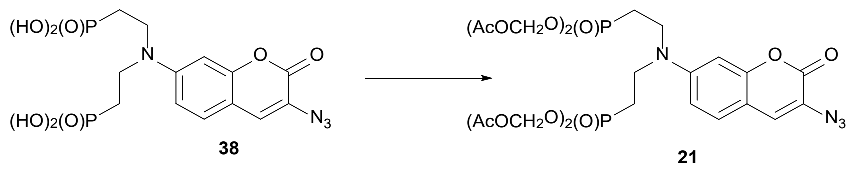

To **38** (286 mg, 0.68 mmol) were added dry MeCN (3mL), diisopropylethylamine (1.49 mL, 8.6 mmol) and bromomethyl acetate (700 µL, 7.1 mmol). The resulting solution was stirred protected from light at 23 °C for 17 h. Volatiles were removed at 23 °C/0.06 mbar. The semi-solid dark residue obtained was partitioned between EtOAc (30 mL) and water (10 mL). The organic phase was separated, dried (Na_2SO_4) and evaporated under reduced pressure to give a dark oil (407 mg). This crude material was purified by chromatography (EtOAc:MeOH 95:5) to give **21**.

Yield: 286 mg (59%), t_R 80% MeOH = 2.4 min, 70% MeOH = 4.9 min, R_f EtOAc:MeOH 9:1 = 0.57, 95:5= 0.38.

^{1}H NMR (400 MHz, $CDCl_3$): δ = 7.28 (d, *J*=9Hz, 1H, H-5), 7.12 (s, 1H, H-4), 6.61 (dd, *J*=9Hz, 2Hz, 1H, H-6), 6.51 (d, *J*=Hz, 1H, H-8), 5.77 – 5.61 (m, 8H, 4 x OCH_2), 3.77 – 3.61 (m, 4H, 2 x NCH_2), 2.27 – 2.11 (m, 4H, 2 x PCH_2) 2.17 (s, 12 H, 4 x CH_3).

^{31}P{^{1}H} NMR (162 MHz, $CDCl_3$): δ = 28.45.

^{13}C{^{1}H} NMR (101 MHz, $CDCl_3$): δ = 169.53 (CH_3CO), 158.01, 153.85, 148.01, 128.77, 126.87, 121.54, 109.95, 109.83, 98.59, 81.37 (d, J_{CP}=6Hz, $\underline{C}H_2O$), 77.48, 77.16, 76.85, 44.58, 24.62 (d, J_{CP}=136Hz, $P\underline{C}H_2$).

HRMS: Chemical Formula: $C_{25}H_{32}N_4NaO_{16}P_2^+$ $[M+Na]^+$

Exact Mass: Calculated 729.1181 Found 729.1204

(2-Ethylphosphonic acid)(2-oxo-2*H*-chromen-4-methyl-7-yl)amine diethyl ester (**39**) and (2-ethylphosphonic acid)(2-oxo-2*H*-chromen-4-methyl-7-yl)ethylamine diethyl ester (**40**)

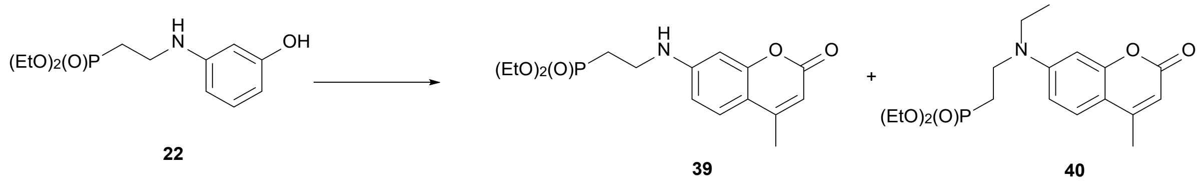

A solution of **22** (3.91 g, 14.3 mmol), anhydrous zinc chloride (1 g, 7.3 mmol) and ethyl acetoacetate (2.17 mL, 17.1 mmol) in absolute EtOH (4.5 mL) was refluxed under an argon atmosphere. After 20 h, the mixture was diluted with water (50 mL) and 1 N HCl (1 mL) and extracted three times with EtOAc (3 x 50 mL). The combined organic phases were evaporated

under reduced pressure. The semi-pure compound obtained by chromatography was further purified by preparative HPLC to afford both **39** and **40**.

39 Yield: 1.5 g (31%) tan solid, t_R 60% MeOH = 9 min.

mp: 72.5-74.5ºC

^{1}H NMR (400 MHz, $CDCl_3$): δ = 7.18 (d, *J*=9Hz, 1H, H-5), 6.41 (dd, *J*=9Hz, 2Hz, 1H, H-6), 6.30 (d, *J*=2Hz, 1H, H-8), 5.78 (s, 1H, H-3), 5.39 (s, 1H, NH), 4.08 – 3.88 (m, 4H, 2xOCH_2), 3.44 – 3.25 (m, 2H, NCH_2), 2.17 (s, 3H, 4-CH_3), 2.00 (dt, *J*=18Hz, 7Hz, 2H, PCH_2), 1.19 (t, J=7Hz, 6H, 2xCH_3).

^{31}P{^{1}H} NMR (162 MHz, $CDCl_3$): δ = 28.93.

^{13}C{^{1}H} NMR (101 MHz, $CDCl_3$): δ = 161.89, 155.73, 153.24, 151.13, 125.52, 110.35, 110.31, 108.92, 97.74, 77.68, 61.81 (d, J_{CP}=6.5, 2 x OCH_2), 37.22 (d, J_{CP}=3.4, NCH_2), 25.13 (d, J_{CP}=139.1, PCH_2), 18.40 (s, 4-CH_3), 16.36 (d, J_{CP}=6.0, 2 x $CH_2\underline{C}H_3$).

HRMS: Chemical Formula: $C_{16}H_{23}NO_5P$ $[M+H]^+$

Exact Mass: Calculated 340.1308 Found 340.1310

40 Yield: 480 mg (9%) amber oil; t_R 70% MeOH = 6.8 min, t_R 60% MeOH = 24.5 min

^{1}H NMR (400 MHz, $CDCl_3$): δ = 7.42 (d, *J*=9Hz, 1H, H-5), 6.62 (dd, *J*=Hz, 2Hz, 1H, H-6), 6.51 (d, *J*=2Hz, 1H, H-8), 5.98 (s, 1H, H-3), 4.15 (dd, *J*=15Hz, 7Hz, 4H, 2xOCH_2), 3.66 (dd, *J*=16Hz, 8Hz, 2H, $NC\underline{H}_2$), 3.45 (q, *J*=7Hz, 2H, $NC\underline{H}_2CH_3$), 2.36 (s, 3H, CH_3), 2.18 – 1.99 (m, 2H, PCH_2), 1.37 (t, *J*=7Hz, 6H, 2 x CH_3), 1.22 (t, *J*=7Hz, 3H, CH_3).

^{31}P{^{1}H} NMR (162 MHz, $CDCl_3$): δ = 28.15.

^{13}C{^{1}H} NMR (101 MHz, $CDCl_3$): δ = 161.75, 161.74, 155.79, 152.76, 149.75, 125.70, 109.52, 109.07, 108.46, 97.76, 61.73 (d, *J*=6Hz, OCH_2), 44.66 (d, J_{CP}=83Hz, $CH_2\underline{C}H_2N$), 23.89 (d, J_{CP}=135Hz, PCH_2), 18.32 (s, 4-CH_3), 16.41 (d, J_{CP}=6Hz, $OCH_2\underline{C}H_3$), 12.23 (s, $NCH_2\underline{C}H_3$).

HRMS: Chemical Formula: $C_{18}H_{27}NO_5P$ $[M+H]^+$

Exact Mass: Calculated 368.1621 Found 368.1625

(2-Ethylphosphonic acid)(2-oxo-2*H*-chromen-4-(2-dimethylaminoethenyl)-7-yl)ethylamine diethyl ester (**41**)

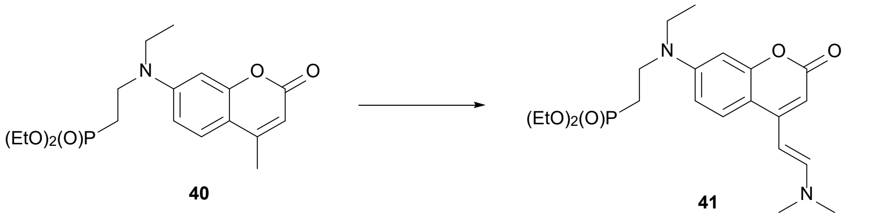

40 41

A solution of **40** (470 mg, 1.3 mmol) in DMF-DMA (1 mL, 7.5 mmol) and DMF (2 mL) was stirred at 140 °C for 24 h under an argon atmosphere. Volatiles were distilled off and the colored foam obtained was purified by chromatography (EtOAc:MeOH 9:1). Yield: 431 mg (80%) amber foam, t_R 100% MeOH = 1.9 min, 85% MeOH = 3.2 min, 70% MeOH = 8.7 min.

^{1}H NMR (400 MHz, $CDCl_3$): δ = 7.58 (d, *J*=9Hz, 1H, H-5), 7.26 (d, *J*=13Hz, 1H, $C\underline{H}$=CHN), 6.58 (dd, *J*=9Hz, 2Hz, 1H, H-6), 6.51 (d, *J*=2Hz, 1H, H-8), 5.90 (s, 1H, H-3), 5.23 (d, *J*=13Hz, 1H, =CHN), 4.24 – 4.06 (m, 4H, 2 x OCH_2), 3.73 – 3.61 (m, 2H, $NC\underline{H}_2CH_2$), 3.44 (q, *J*=7.1, 2H, $NC\underline{H}_2CH_3$), 3.02 (s, 6H, $N(CH_3)_2$), 2.17 – 2.00 (m, 2H, PCH_2), 1.37 (t, *J*=7Hz, 6H, 2 x $OCH_2C\underline{H}_3$), 1.22 (t, *J*=7.1, 3H, $NCH_2C\underline{H}_3$).

^{31}P{^{1}H} NMR (162 MHz, $CDCl_3$): δ = 28.52.

^{13}C{^{1}H} NMR (101 MHz, $CDCl_3$): δ = 163.28, 156.29, 152.24, 149.38, 146.76, 125.07, 108.83, 108.00, 98.52, 93.81, 87.26, 77.37, 77.05, 76.74, 61.87, 61.84 (d, J_{CP}=6Hz, OCH_2), 45.15, 44.31, 40.89 ($N(CH_3)_2$), 24.06 (d, J_{CP}=135Hz, PCH_2), 16.53 (d, J_{CP}=6Hz, $OCH_2\underline{C}H_3$), 12.41 (s, $NCH_2\underline{C}H_3$).

HRMS: Chemical Formula: $C_{21}H_{32}N_2O_5P$ $[M+H]^+$

Exact Mass: Calculated 423.2043 Found 423.2045

(2-Ethylphosphonic acid)(2-oxo-2*H*-chromen-4-formyl-7-yl)ethylamine diethyl ester (**42**)

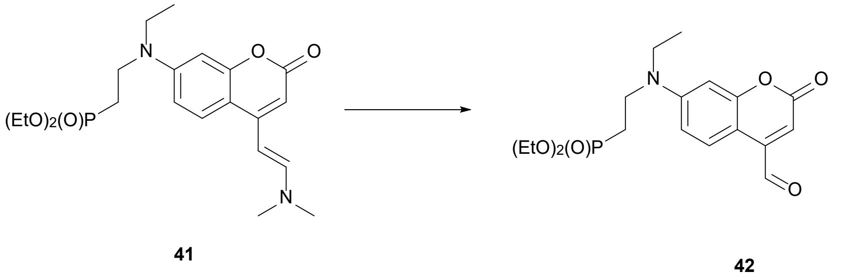

41 42

A mixture of **41** (460 mg, 1.1 mmol), sodium periodate (700 mg, 3.3 mmol), water (4.5 mL) and THF (4.5 mL) was vigorously stirred at 23 °C. After 2 h the dark reaction was diluted with EtOAc (100 mL), filtered, washed two times with saturated aqueous $NaHCO_3$ (2 x 50 mL), brine

(50 mL) and water (5 mL). Volatiles were removed under reduced pressure and the black-red tar obtained was dried at 23 °C/0.08 mbar. Yield: 461 mg tar, t_R 85% MeOH = 2.1 min, 70% MeOH = 3.6 min.

^{1}H NMR (400 MHz, $CDCl_3$): δ = 10.04 (s, 1H, CHO), 8.36 (d, *J*=9Hz, 1H, H-5), 6.65 (dd, *J*=9Hz, 3Hz, 1H, H-6), 6.54 (d, *J*=3Hz, 1H, H-8), 6.52 (s, 1H, H-3), 4.27 – 4.04 (m, 4H, 2 x OCH_2), 3.74 - 3.62 (m, 2H, $NC\underline{H}_2CH_2$), 3.47 (q, *J*=7Hz, 2H, $NC\underline{H}_2CH_3$), 2.14 – 2.04 (m, 2H, PCH_2), 1.38 (t, *J*=7.1, 6H, 2 x $OCH_2C\underline{H}_3$), 1.23 (t, *J*=7Hz, 3H, $NCH_2C\underline{H}_3$).

^{31}P{^{1}H} NMR (162 MHz, $CDCl_3$): δ = 27.95.

^{13}C{^{1}H} NMR (101 MHz, $CDCl_3$): δ = 192.51 (CHO), 161.72, 157.35, 150.48, 143.92, 127.42, 118.41, 109.65, 104.39, 98.06, 77.48, 77.16, 76.84, 62.06 (d, J_{CP}=7Hz, OCH_2), 45.36, 44.50, 24.18 (d, J_{CP}=136Hz, PCH_2), 16.62 (d, J_{CP}=6Hz, 2 x $OCH_2\underline{C}H_3$) 12.43 (s, $NCH_2\underline{C}H_3$).

HRMS: Chemical Formula: $C_{36}H_{48}N_2NaO_{12}P_2$ $[2M+Na]^+$

Exact Mass: Calculated 785.2575 Found 785.2599

(2-Ethylphosphonic acid)(2-oxo-2*H*-chromen-4-hydroxymethyl-7-yl)ethylamine diethyl ester (**43**)

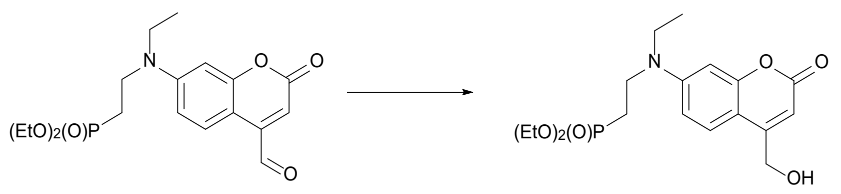

42 **43**

To a solution of **42** (460 mg, 1.2 mmol) in MeOH (20 mL) was added $NaBH_4$ (91 mg, 2.4 mmol). After 2 h, 1 N HCl (1 mL) was added and the slightly colored reaction mixture was evaporated under reduced pressure. The residue obtained was purified by chromatography (EtOAc:MeOH 99:1 → 92:8). Yield: 293 mg (63%) yellow oil, R_f EtOAc:MeOH 9:1 =0.37; t_R 70% MeOH = 3.2 min.

^{1}H NMR (400 MHz, $CDCl_3$): δ = 7.33 (d, *J*=9Hz, 1H, H-5), 6.54 (dd, *J*=9Hz, 2Hz, 1H, H-4), 6.45 (d, *J*=2Hz, 1H, H-8), 6.33 (s, 1H, H-3), 4.80 (s, 2H, 4-CH_2), 4.23 – 4.01 (m, 4H, 2 x OCH_2), 3.60 (dd, *J*=16Hz, 8Hz, 2H, $NC\underline{H}_2CH_2$), 3.40 (q, *J*=7Hz, 2H, $NC\underline{H}_2CH_3$), 2.12 – 1.97 (m, 2H, PCH_2), 1.34 (t, *J*=7Hz, 6H, 2 x $OCH_2C\underline{H}_3$), 1.18 (t, *J*=7Hz, 3H, $NCH_2C\underline{H}_3$).

^{31}P{^{1}H} NMR (162 MHz, $CDCl_3$): δ = 28.44.

$^{13}C\{^1H\}$ NMR (101 MHz, $CDCl_3$): δ = 162.60, 156.02, 155.56, 149.77, 124.71, 108.71, 107.22, 106.04, 98.15, 77.48, 77.16, 76.84, 62.12 (d, J_{CP} = 6Hz, O<u>C</u>H_2CH_3), 60.59 (4-CH_2), 45.23, 44.31, 24.00 (d, J_{CP} = 136Hz, PCH_2), 16.55 (d, J_{CP} = 7Hz, OCH_2<u>C</u>H_3), 12.38 (NCH_2<u>C</u>H_3).
HRMS: Chemical Formula: $C_{18}H_{27}NO_6P$ $[M+H]^+$
Exact Mass: Calculated 384.1571 Found 384.1575

(2-Ethylphosphonic acid)(4-azido-2-oxo-2*H*-chromen-7-yl)ethylamine diethyl ester (**44**)

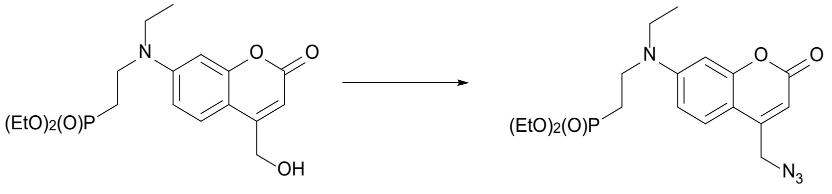

43 **44**

To **43** (265 mg, 0.69 mmol), anhydrous DCM (2 mL), NEt_3 (209 µL, 1.5 mmol) and diphenyl phosphoryl azide (237 µL, 1.1 mmol) were added. After stirring at 22 °C for 17 h the solution was diluted with DCM (20 mL), washed with water (20 mL) and phosphate buffer (20 mL) and evaporated under reduced pressure. The crude (437 mg) was purified by chromatography (EtOAc). Yield: 204 mg (72%) orange oil, R_f EtOAc = 0.17; t_R 70% MeOH = 7.9 min.
^1H NMR (400 MHz, $CDCl_3$): δ = 7.37 (d, *J*=9Hz, 1H, H-5), 6.62 (dd, *J*=9Hz, 2Hz, 1H, H-6), 6.53 (d, *J*=2Hz, 1H, H-8), 6.18 (s, 1H, H-3), 4.46 (s, 2H, 4-CH_2), 4.25 – 4.08 (m, 4H, 2 x OCH_2), 3.73 – 3.60 (m, 2H, NC<u>H</u>$_2CH_2$), 3.45 (q, *J*=7Hz, 2H, NC<u>H</u>$_2CH_3$), 2.15 – 2.01 (m, 2H, PCH_2), 1.37 (t, *J*=7Hz, 6H, 2 x OCH_2C<u>H</u>$_3$), 1.22 (t, *J*=7Hz, 3H, NCH_2C<u>H</u>$_3$).
$^{31}P\{^1H\}$ NMR (162 MHz, $CDCl_3$): δ = 28.06.
$^{13}C\{^1H\}$ NMR (101 MHz, $CDCl_3$): δ = 161.50, 156.42, 150.21, 148.78, 125.12, 108.93, 108.86, 107.01, 98.36, 77.48, 77.16, 76.84, 62.01 (d, J_{CP} = 6Hz, OCH_2), 50.92 (4-CH_2), 45.36, 44.51, 24.14 (d, J_{CP} = 136Hz, PCH_2), 16.61 (d, J_{CP} = 6Hz, OCH_2<u>C</u>H_3), 12.40 (NCH_2<u>C</u>H_3).
HRMS: Chemical Formula: $C_{18}H_{25}N_4NaO_5P$ $[M+Na]^+$
Exact Mass: Calculated 431.1457 Found 431.1455
UV (2-PrOH)

λmax	ε	log ε
372	24086	4.38
246	16498	4.22

202 40311 4.61

(2-Ethylphosphonic acid)(4-azido-2-oxo-2*H*-chromen-7-yl)ethylamine (**45**)

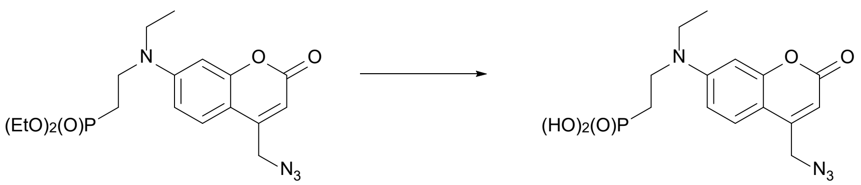

44 **45**

To dry azide **44** (196 mg, 0.48 mmol) was added bromotrimethylsilane (2.5 mL, 18.9 mmol). After stirring for 16 h at 23 °C volatiles were removed at 23 °C/0.03 mbar. The resulting foam was dissolved in MeOH (30 mL) and evaporated under reduced pressure. The foam obtained was dried at 23°C/0.04 mbar. Yield (crude): 210 mg.

^{1}H NMR (400 MHz, MeOD): δ = 7.73 (d, *J*=8Hz, 1H, H-5), 7.19 (d, *J*=9Hz, 1H, H-6), 7.14 (s, 1H, H-8), 6.36 (s, 1H, H-3), 4.72 (s, 2H, CH_2N_3), 3.89 – 3.74 (m, 2H, $NC\underline{H}_2CH_2$), 3.67 (q, *J*=7.1, 2H, $NC\underline{H}_2CH_3$), 2.18 – 1.95 (m, 2H, PCH_2), 1.22 (t, *J*=7.1, 3H, CH_3).

^{31}P{^{1}H} NMR (162 MHz, MeOD): δ = 23.83.

^{13}C{^{1}H} NMR (101 MHz, MeOD): δ = 162.49, 156.75, 151.52, 146.95, 127.54, 114.65, 113.81, 111.86, 105.06, 51.39, 50.29, 49.73, 25.54 (d, J_{CP} = 135Hz, PCH_2), 11.82 (CH_3).

HRMS: Chemical Formula: $C_{14}H_{18}N_4O_5P$ $[M+H]^+$

Exact Mass: Calculated 353.1009 Found 353.1014

(2-Ethylphosphonic acid)(4-azido-2-oxo-2*H*-chromen-7-yl)ethylamine bis(acetoxymethyl)ester (**23**)

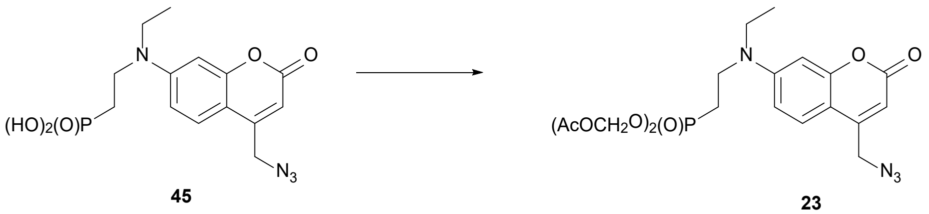

45 **23**

To **45** (200 mg, 0.57 mmol) were added dry MeCN (3 mL), dry diisopropylethylamine (1.2 mL, 6.9 mmol) and bromomethyl acetate (0.5 mL, 5.1 mmol). After stirring at 22 °C for 6 h volatiles were removed at 22 °C/0.04 mbar. The crude mixture was partitioned between EtOAc (30 mL) and water (20 mL), the organic phase was separated, dried (Na_2SO_4) and purified by chromatography (EtOAc). Yield: 137 mg (49%) orange oil, R_f EtOAc = 0.41.

^1H NMR (400 MHz, $CDCl_3$): δ = 7.33 (d, *J*=9Hz, 1H, H-5), 6.56 (dd, *J*=9Hz, 2.3, 1H, H-6), 6.47 (d, *J*=2.3, 1H, H-8), 6.13 (s, 1H, H-3), 5.75 – 5.58 (m, 4H, 2 x OCH_2), 4.43 (s, 2H, 4-CH_2), 3.70 – 3.58 (m, 2H, $NC\underline{H}_2CH_2$), 3.40 (q, *J*=7Hz, 2H, $NC\underline{H}_2CH_3$), 2.27 – 2.05 (m, 2H, PCH_2), 2.13 (s, 6 H, $COCH_3$), 1.18 (t, *J*=7Hz, 3H, CH_3).
$^{31}P\{^1H\}$ NMR (162 MHz, $CDCl_3$): δ = 28.80.
$^{13}C\{^1H\}$ NMR (101 MHz, $CDCl_3$): δ = 169.37 ($\underline{C}OCH_3$), 161.24, 156.25, 149.96, 148.74, 125.10, 108.80, 107.05, 98.28, 81.24 (d, J_{CP} = 6Hz, CH_2O), 77.49, 77.17, 76.85, 50.73, 45.17, 43.77, 24.66 (d, J_{CP} = 135Hz, PC), 20.71 ($CO\underline{C}H_3$), 12.23 ($CH_2\underline{C}H_3$).
HRMS: Chemical Formula: $C_{20}H_{26}N_4O_9P$ $[M+H]^+$
Exact Mass: Calculated 497.1432Found 497.1439

((1R,8S,9S)-Bicyclo[6.1.0]non-4-yn-9-yl)methyl decyl carbonate (**25**)

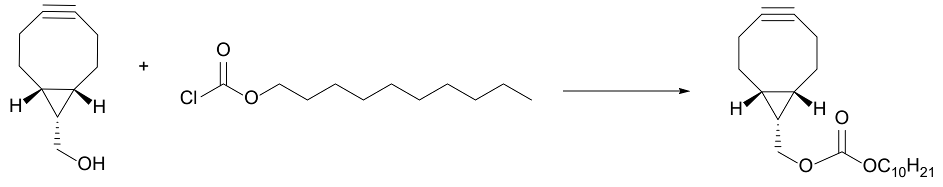

25

To a solution of *endo* BCN-OH (148 mg, 1 mmol) and DMAP (43 mg, 0.35 mmol) in dry pyridine (4 mL) was added decyl chloroformate (231 µL, 1 mmol) under an argon atmosphere. After stirring at 24°C for 3.5 h the suspension was diluted with water (50 mL) and extracted three times with EtOAc (3 x 50 mL). The combined extracts were dried (Na_2SO_4), filtered and evaporated under reduced pressure. The crude **25** (350 mg) was purified by chromatography (cyclohexane:EtOAc 95:5).

Yield: 151 mg (46%), colorless oil, R_f cyclohexane:EtOAc 9:1 = 0.48

^1H NMR (400 MHz, $CDCl_3$): δ = 4.10 (t, *J*=7Hz, 2H), 4.03 (d, *J*=7Hz, 2H), 2.40 (d, *J*=13Hz, 2H), 2.26 (t, *J*=14Hz, 2H), 2.13 (d, *J*=15Hz, 2H), 1.72 – 1.57 (m, 2H), 1.47 – 1.28 (m, 6H), 1.24 (s, 10H), 0.85 (t, *J*=7Hz, 3H), 0.81 – 0.63 (m, 3H).
$^{13}C\{^1H\}$ NMR (101 MHz, $CDCl_3$): δ = 155.40, 98.64, 77.43, 77.11, 76.79, 72.10, 67.99, 33.15, 31.84, 29.48, 29.47, 29.26, 29.20, 28.66, 25.67, 23.22, 22.96, 22.64, 21.28, 14.08.
HRMS: Chemical Formula: $C_{21}H_{34}NaO_3$ $[M+H]^+$
Exact Mass: Calculated 357.2400 Found 357.2399

((1R,8S,9s)-Bicyclo[6.1.0]non-4-yn-9-yl)methyl arachidonate (**26**)

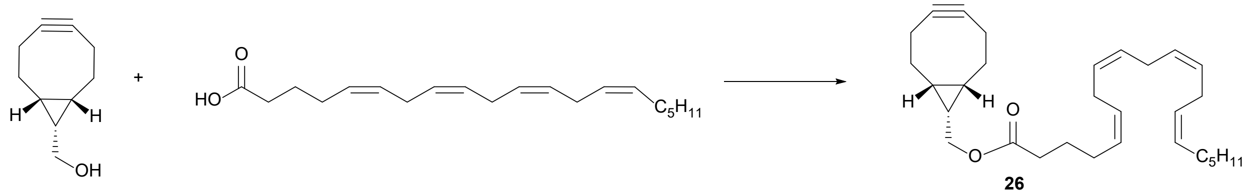

26

A solution of dry *endo* BCN-OH (163 mg, 1.1 mmol), anhydrous DCM (6 mL), arachidonic acid (363 mg, 1.2 mmol), EDC HCl (300 mg, 1.6 mmol) and DMAP (40 mg, 0.4 mmol) was stirred at 24 °C for 3.5 h. The reaction was diluted with EtOAc (50 mL), washed two times with 10% brine (2 x 20 mL), dried (Na_2SO_4) and evaporated under reduced pressure. The crude product was purified by chromatography (cyclohexane:EtOAc 9:1). Yield: 344 mg (73%), colorless oil, Rf cyclohexane:EtOAc 9:1 = 0.68

^{1}H NMR (400 MHz, $CDCl_3$): δ = 5.52 – 5.26 (m, 8H, 4 x HC=CH), 4.01 (d, *J*=7Hz, 2H, CH_2O), 2.92 – 2.75 (m, 6H, 3 x $(CH=CH)_2C\underline{H_2}$), 2.47 – 2.37 (m, 2H), 2.37 – 2.22 (m, 4H), 2.22 – 2.11 (m, 4H), 2.07 (q, *J*=7Hz, 2H), 1.73 (p, *J*=7Hz, 2H), 1.48 – 1.22 (m, 8H), 0.91 (t, *J*=6Hz, 3H, CH_3), 0.82 – 0.65 (m, 3H).

^{13}C{^{1}H} NMR (101 MHz, $CDCl_3$): δ = 173.73 (C=O), 130.49, 128.98, 128.83, 128.59, 128.22, 128.16, 127.85, 127.53, 98.75, 77.38, 77.06, 76.75, 68.50, 33.79, 33.27, 31.52, 29.33, 27.22, 26.57, 25.63, 24.89, 23.47, 22.99, 22.59, 21.38, 14.09.

HRMS: Chemical Formula: $C_{30}H_{44}NaO_2$ $[M+Na]^+$

Exact Mass: Calculated 459.3234 Found 459.3238

NMR spectra

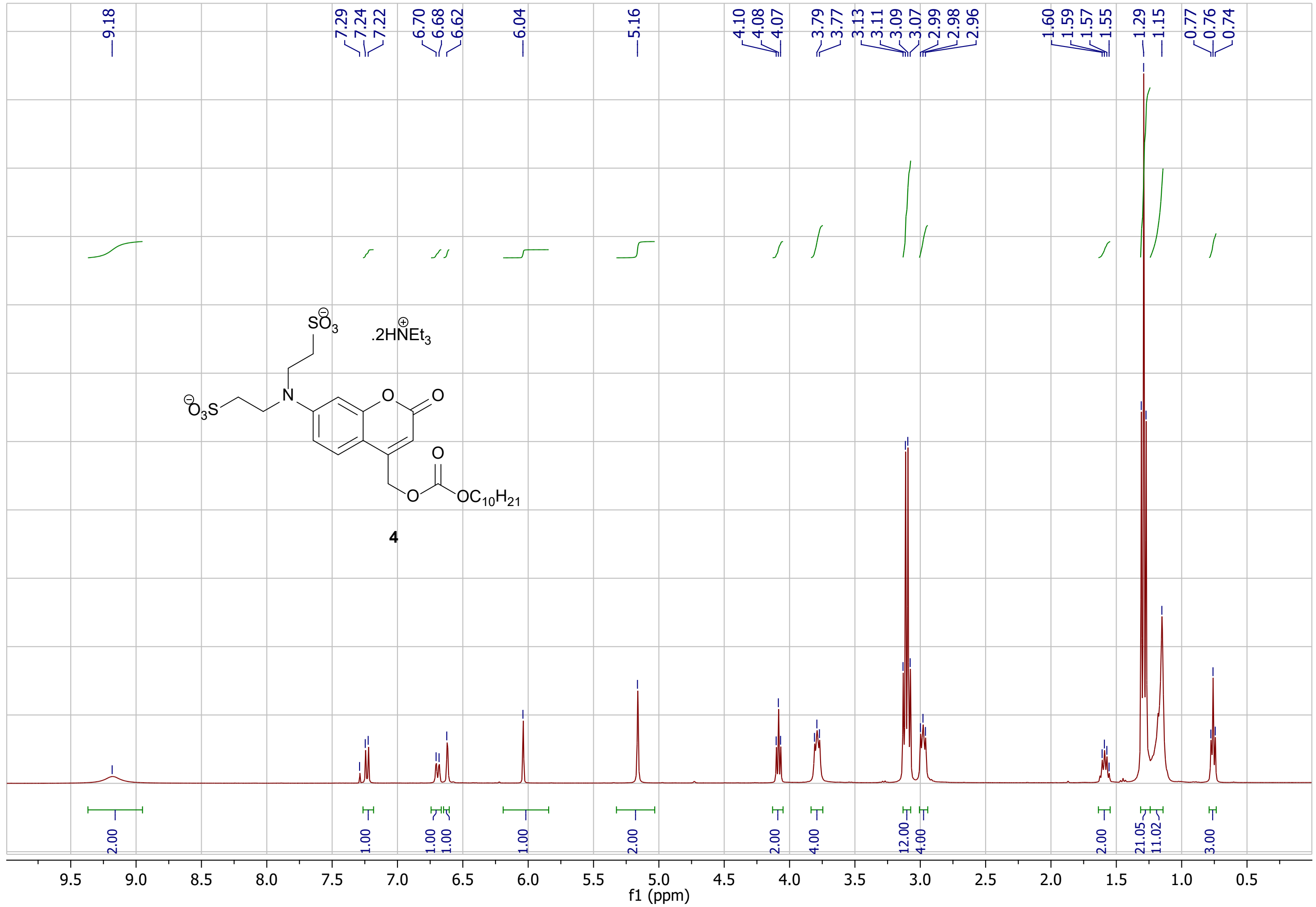

^{1}H NMR spectrum of **4** ($CDCl_3$)

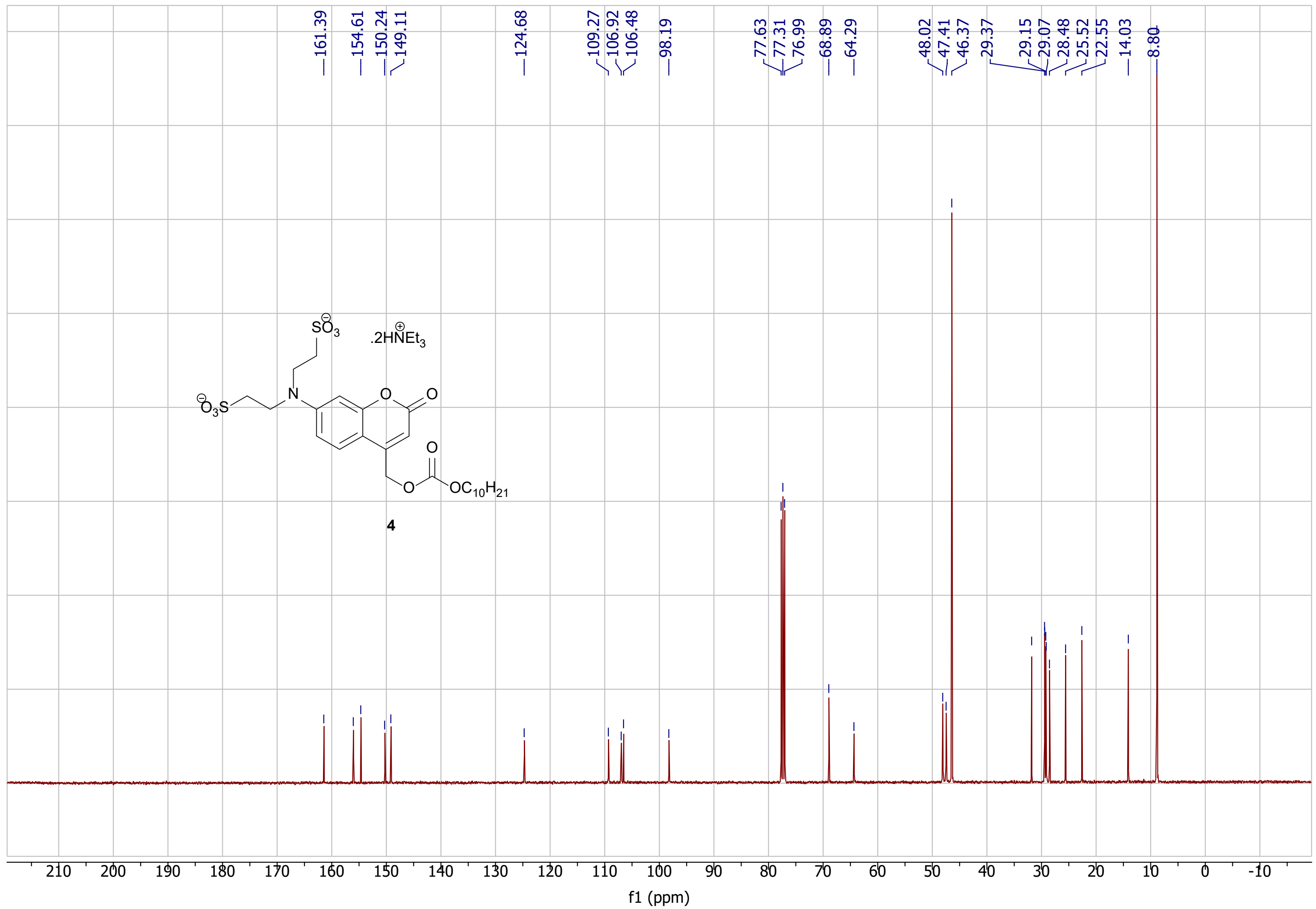

^{13}C NMR spectrum of **4** ($CDCl_3$)

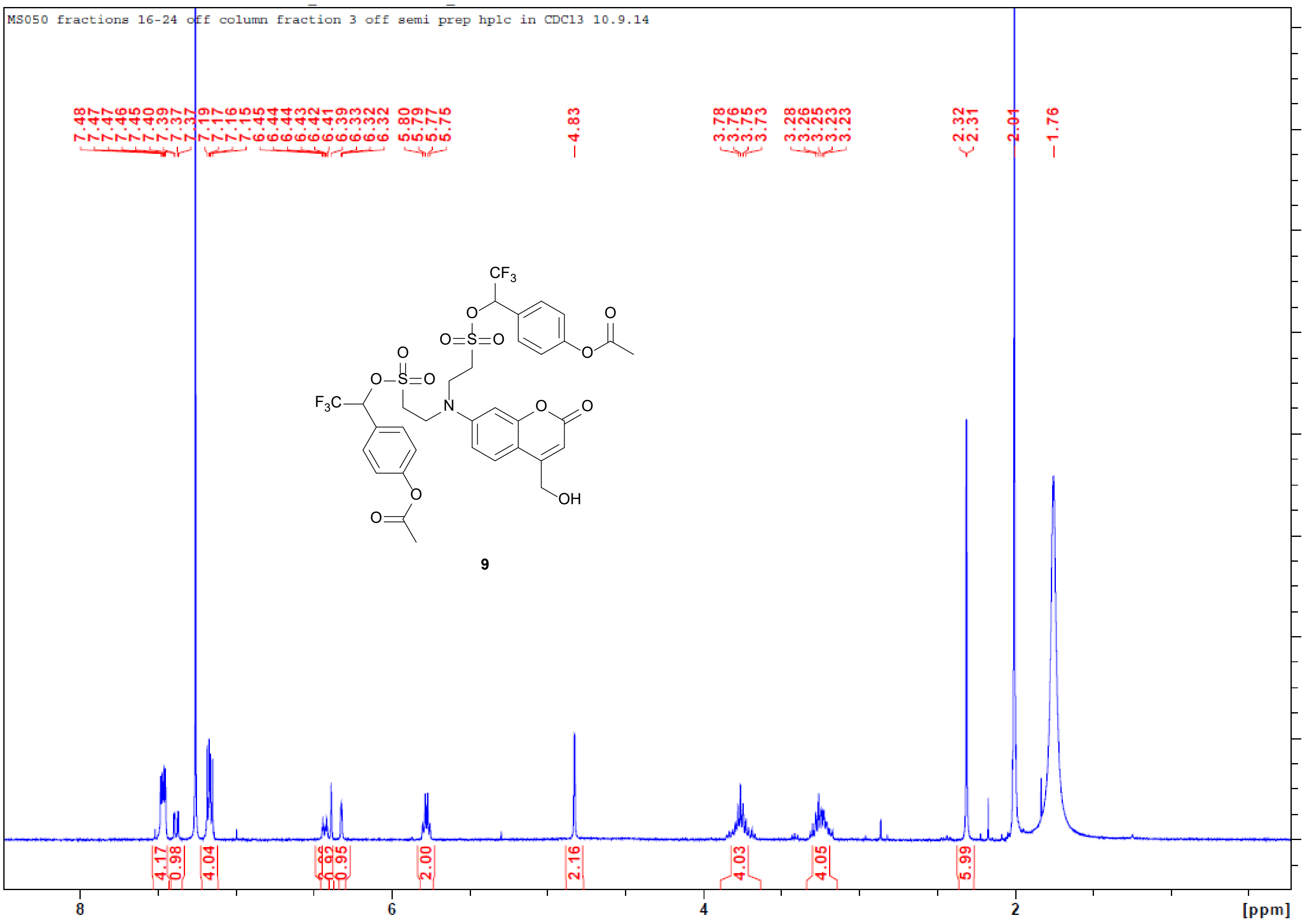

^{1}H NMR spectrum of **9** ($CDCl_3$)

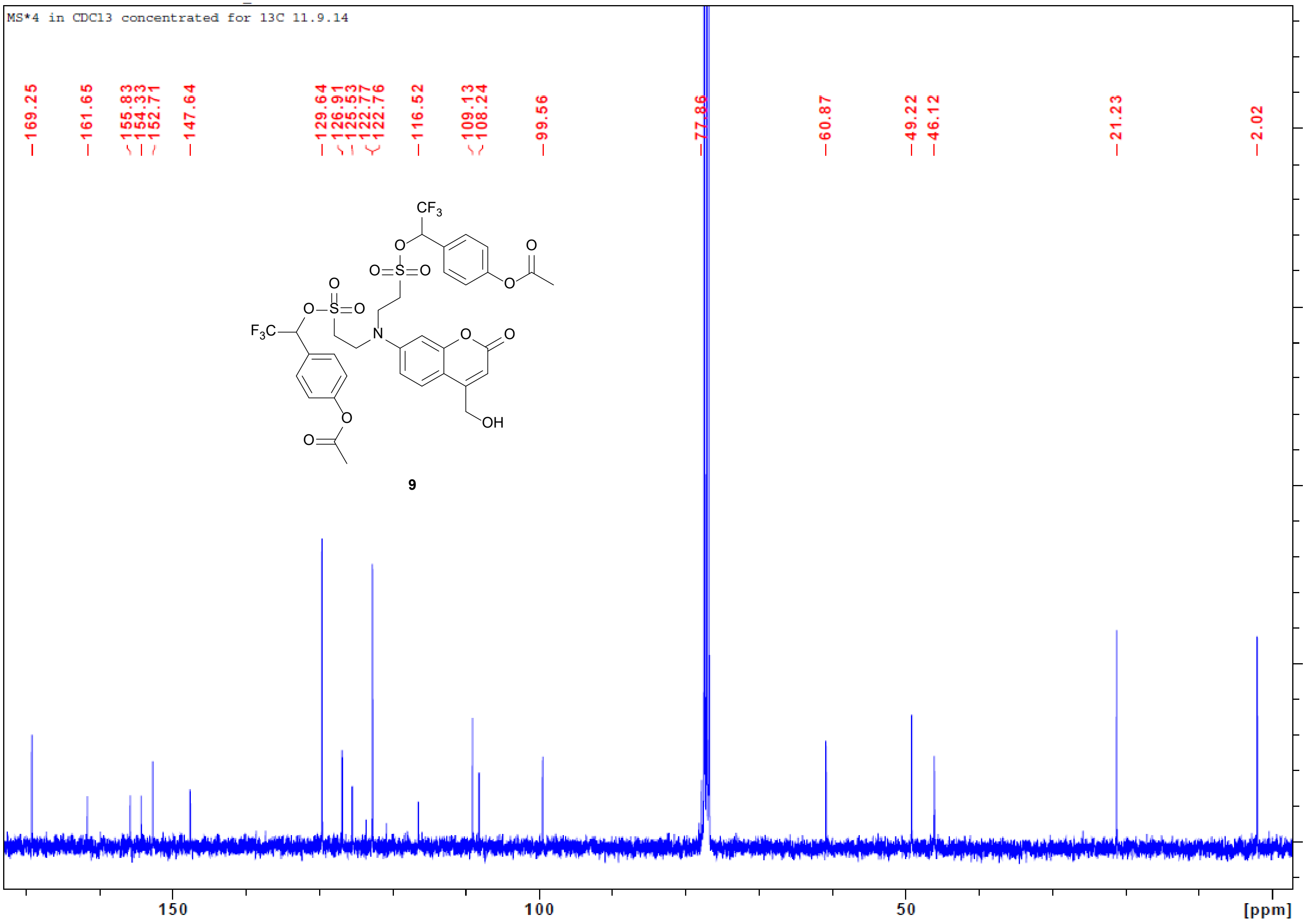

^{13}C NMR spectrum of **9** ($CDCl_3$)

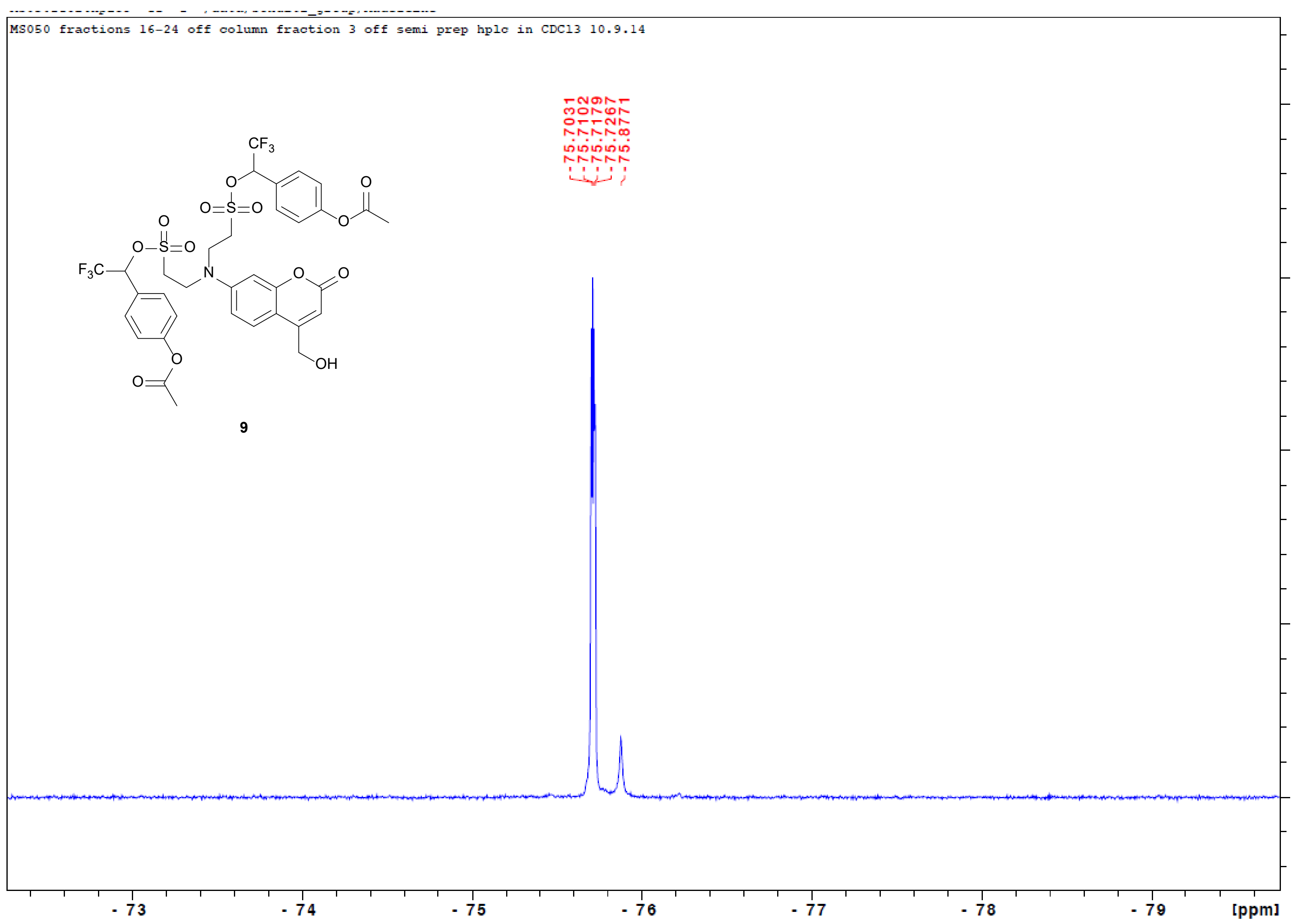

^{19}F NMR spectrum of **9** ($CDCl_3$)

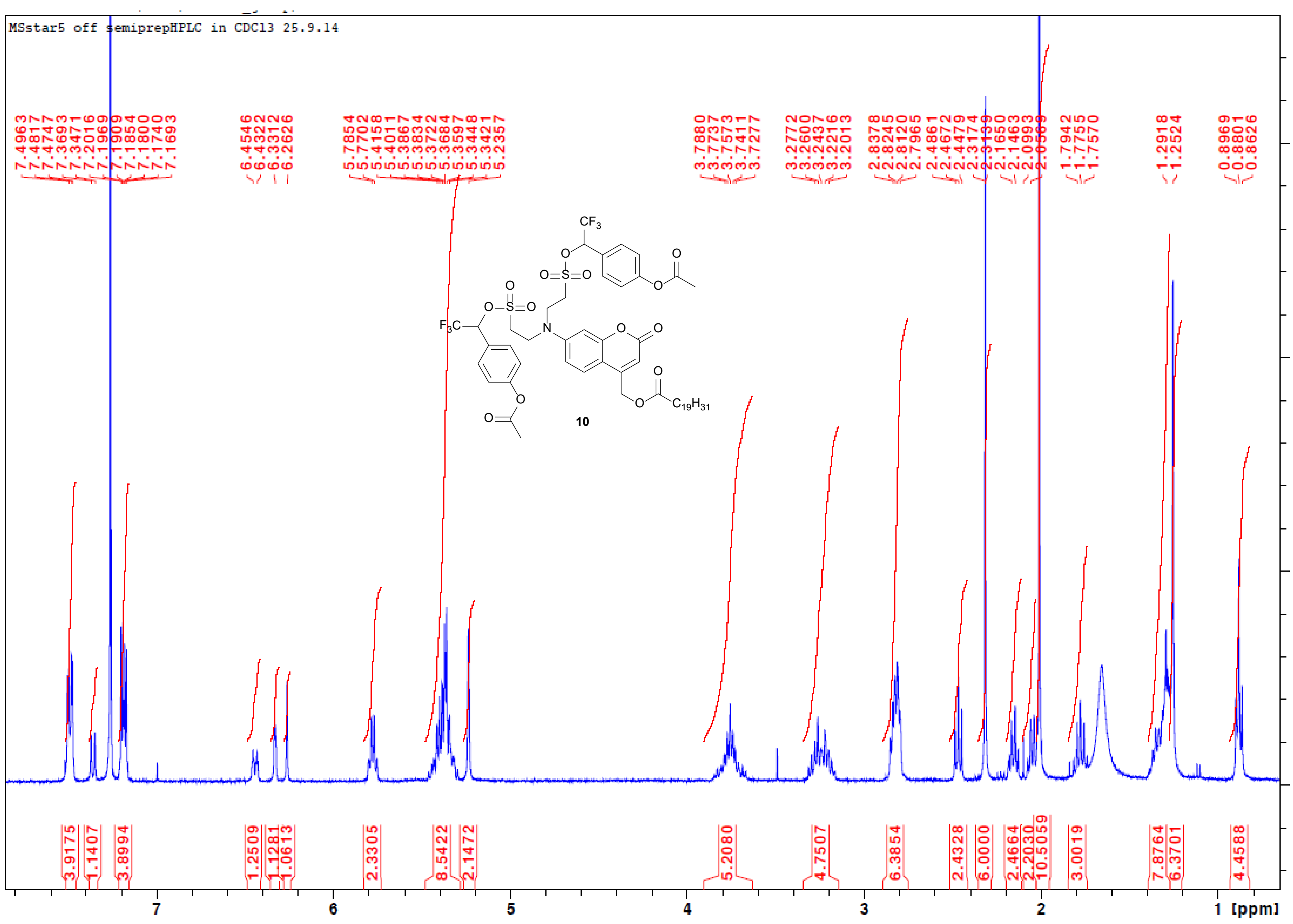

^{1}H NMR spectrum of **10** ($CDCl_3$)

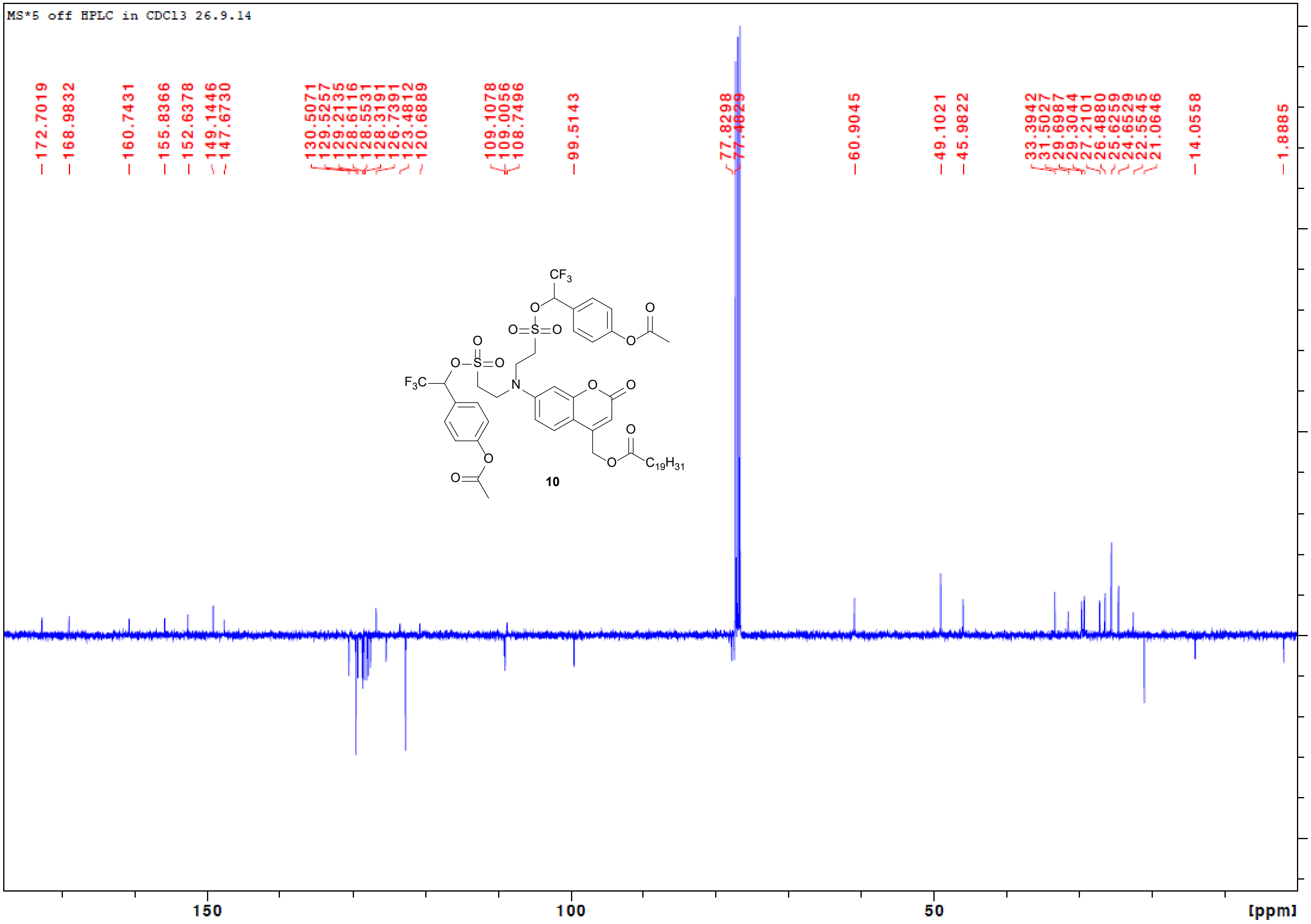

^{13}C NMR spectrum of **10** ($CDCl_3$)

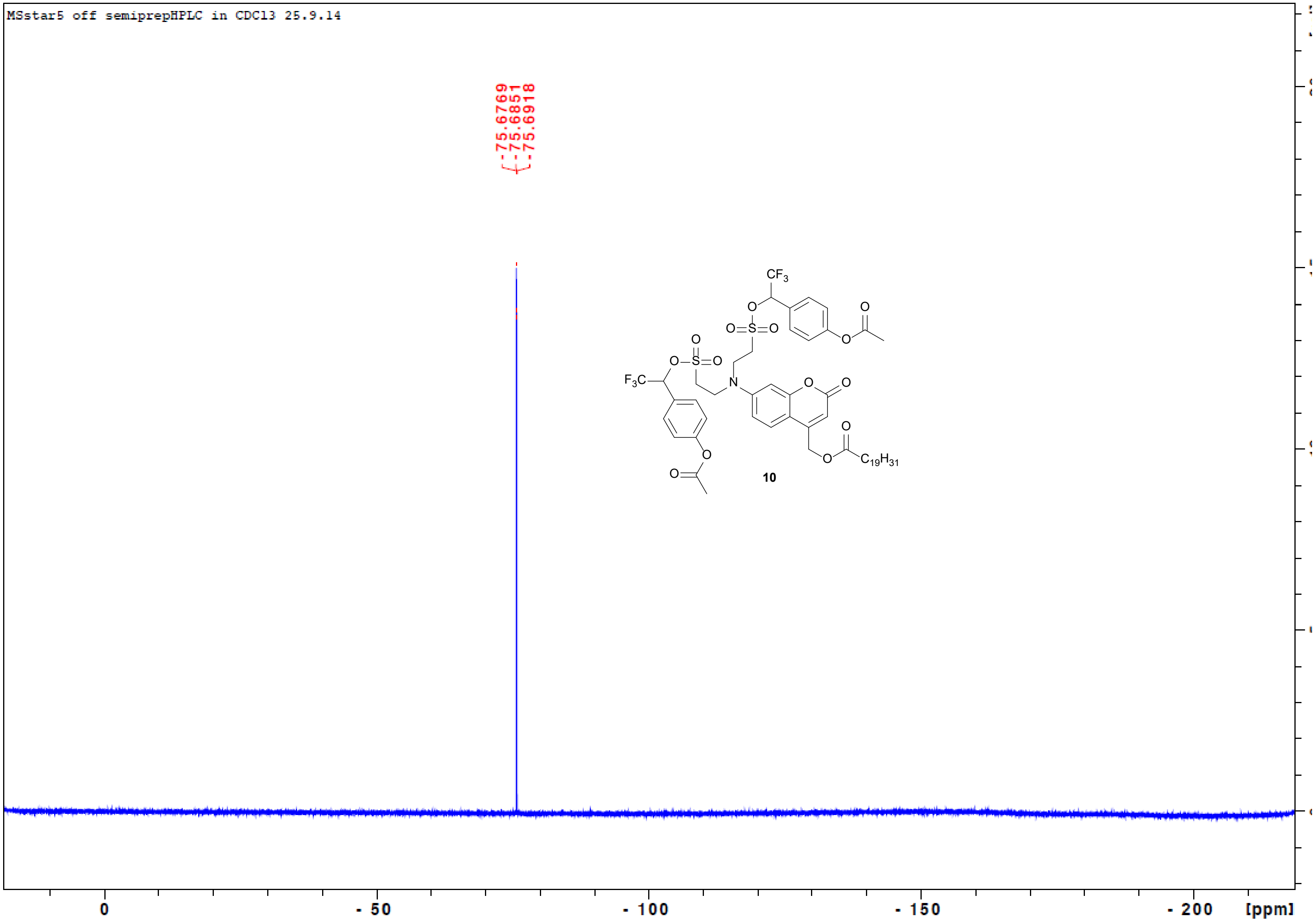

^{19}F NMR spectrum of **10** ($CDCl_3$)

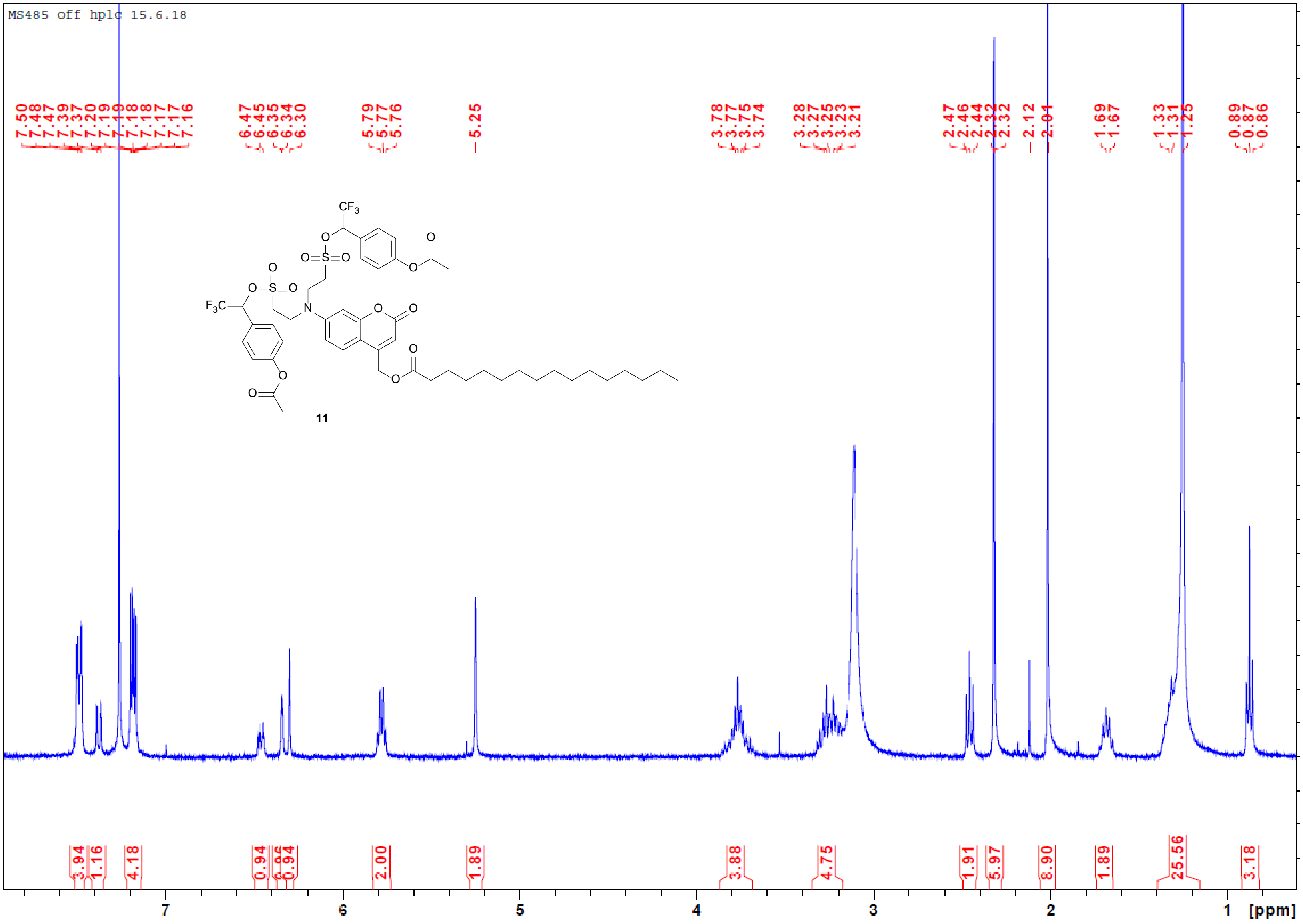

^{1}H NMR spectrum of **11** ($CDCl_3$)

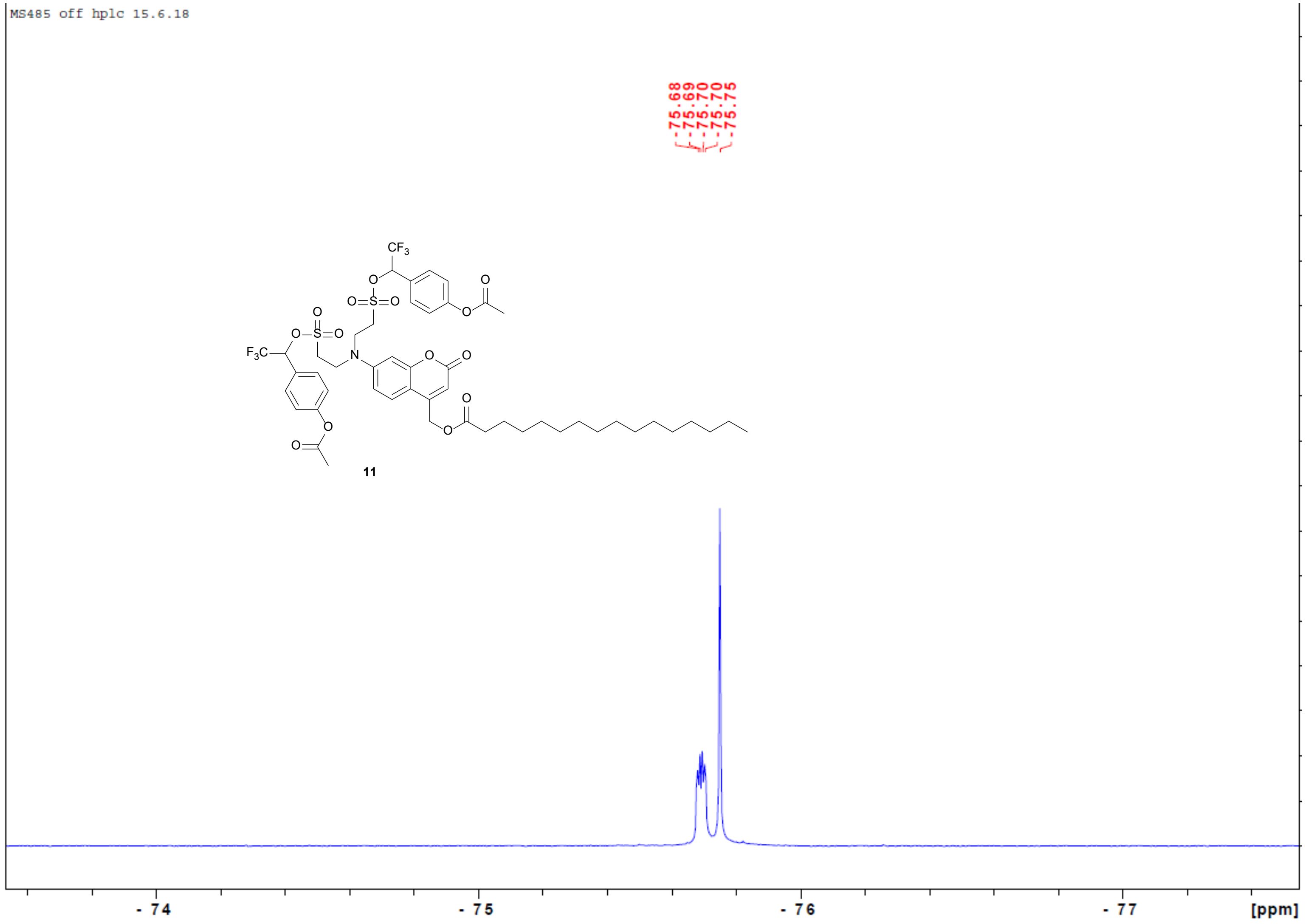

^{19}F NMR spectrum of **11** ($CDCl_3$). Sample contained residual TFA from HPLC.

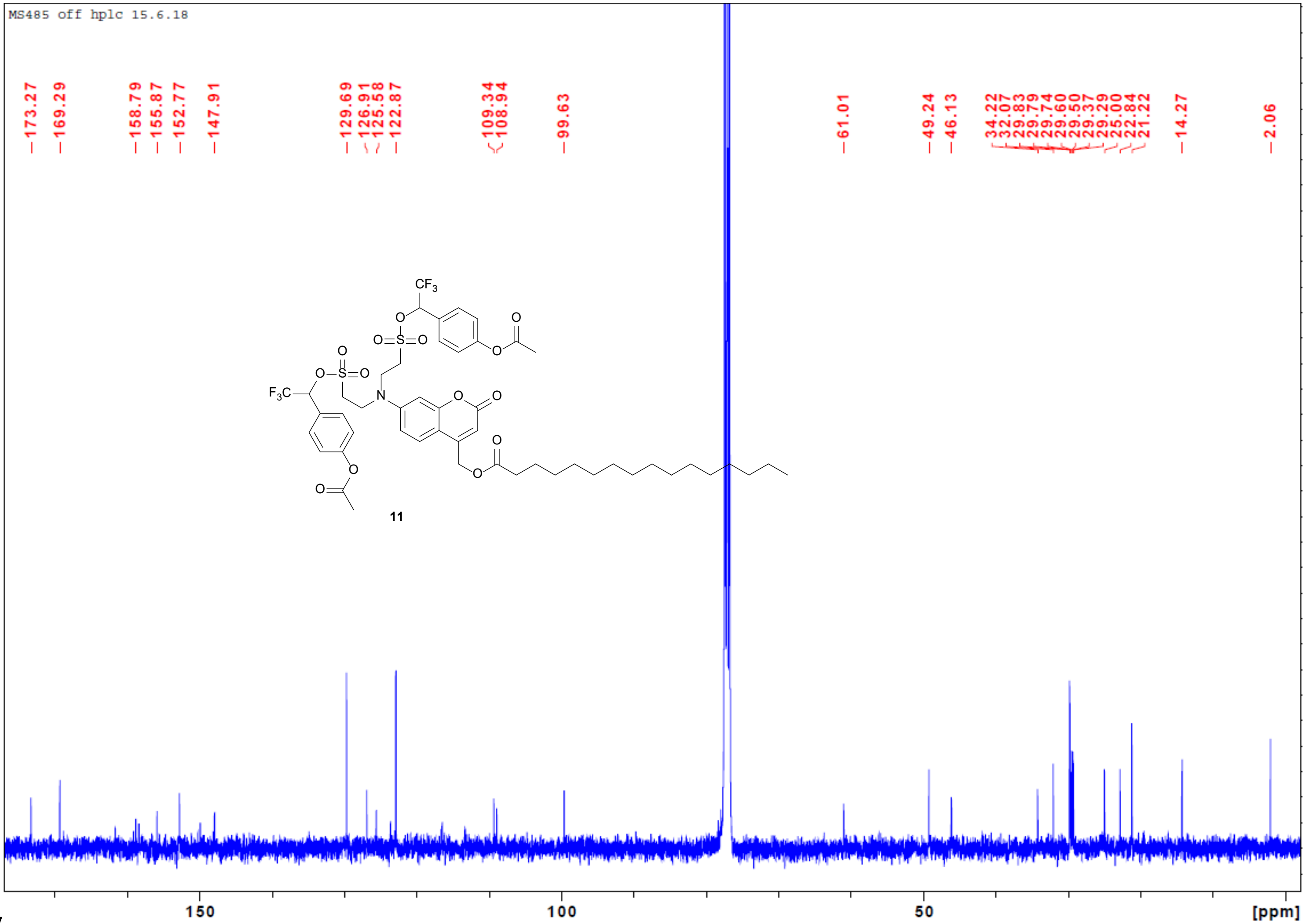

V

^{13}C NMR spectrum of **11** ($CDCl_3$)

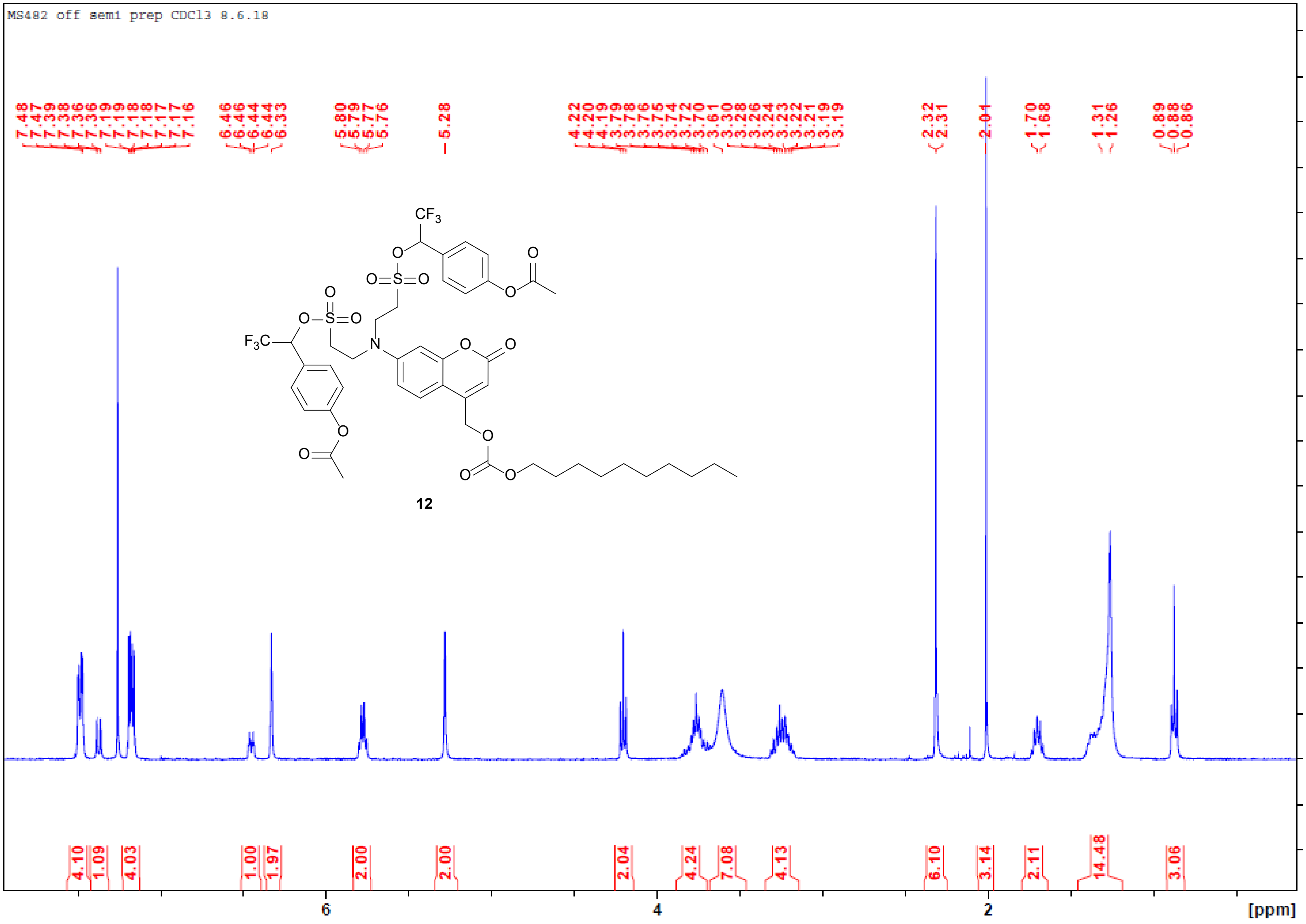

^{1}H NMR spectrum of **12** ($CDCl_3$)

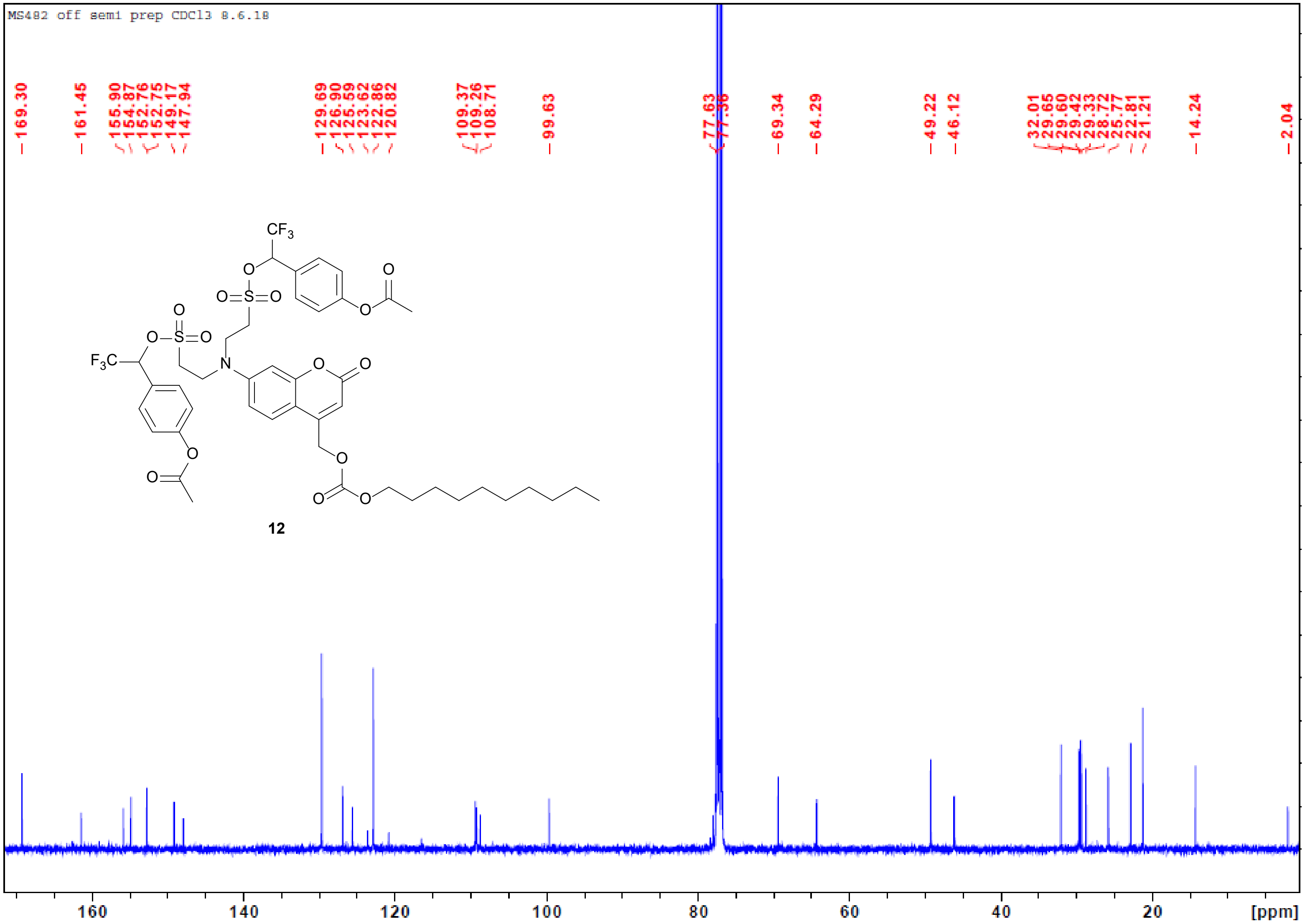

^{13}C NMR spectrum of **12** ($CDCl_3$)

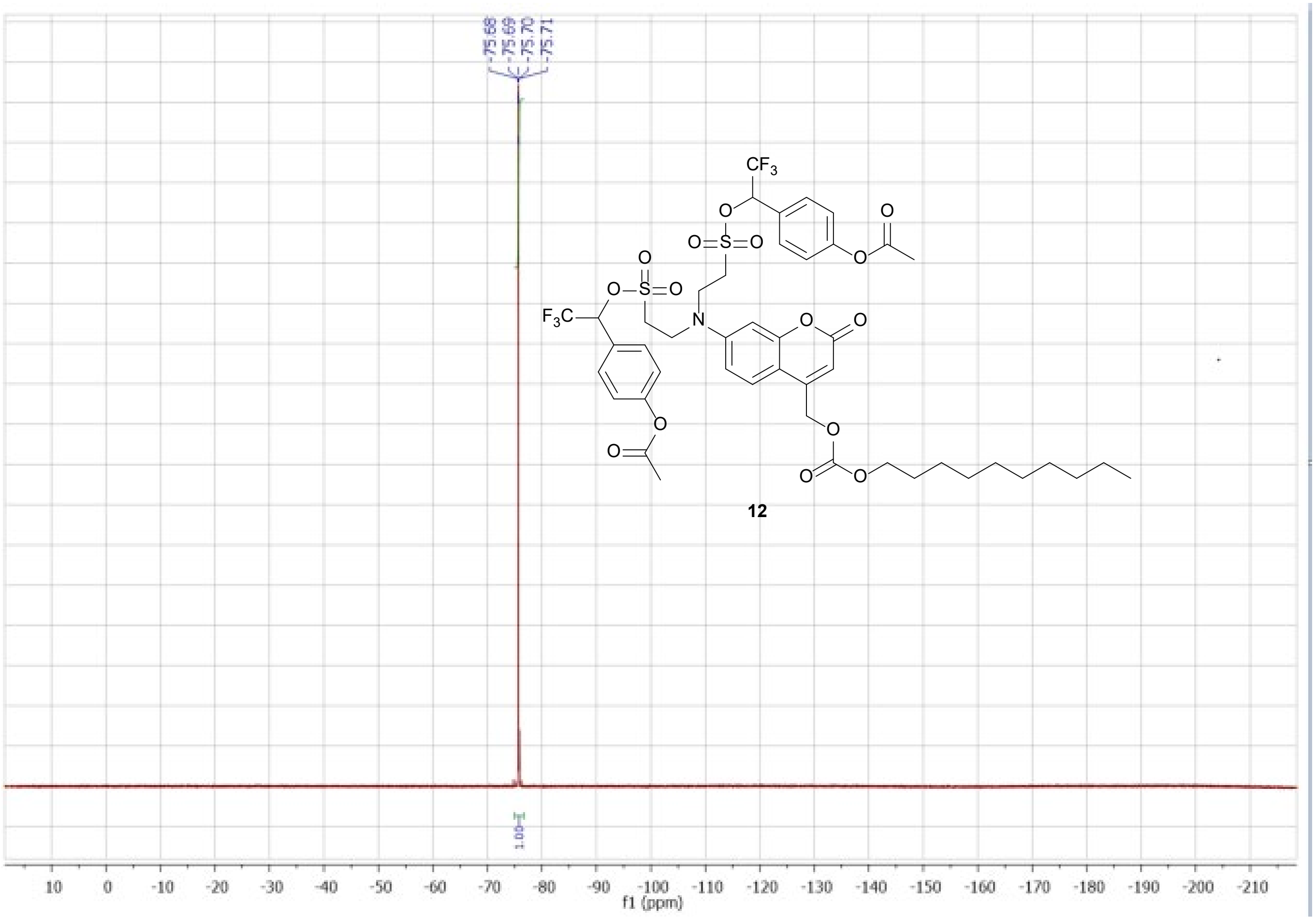

^{19}F NMR spectrum of **12** ($CDCl_3$)

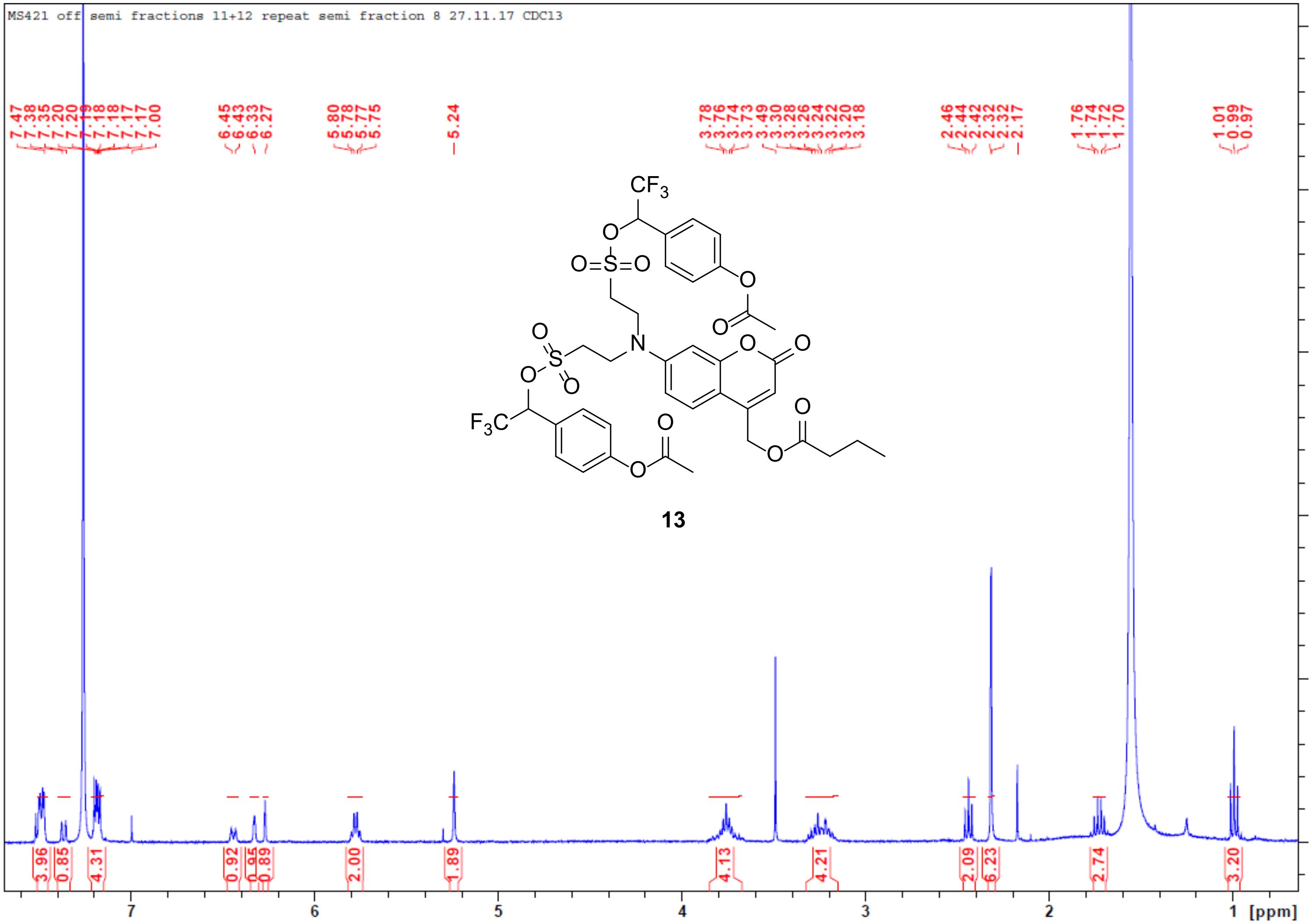

^{1}H NMR spectrum of **13** ($CDCl_3$)

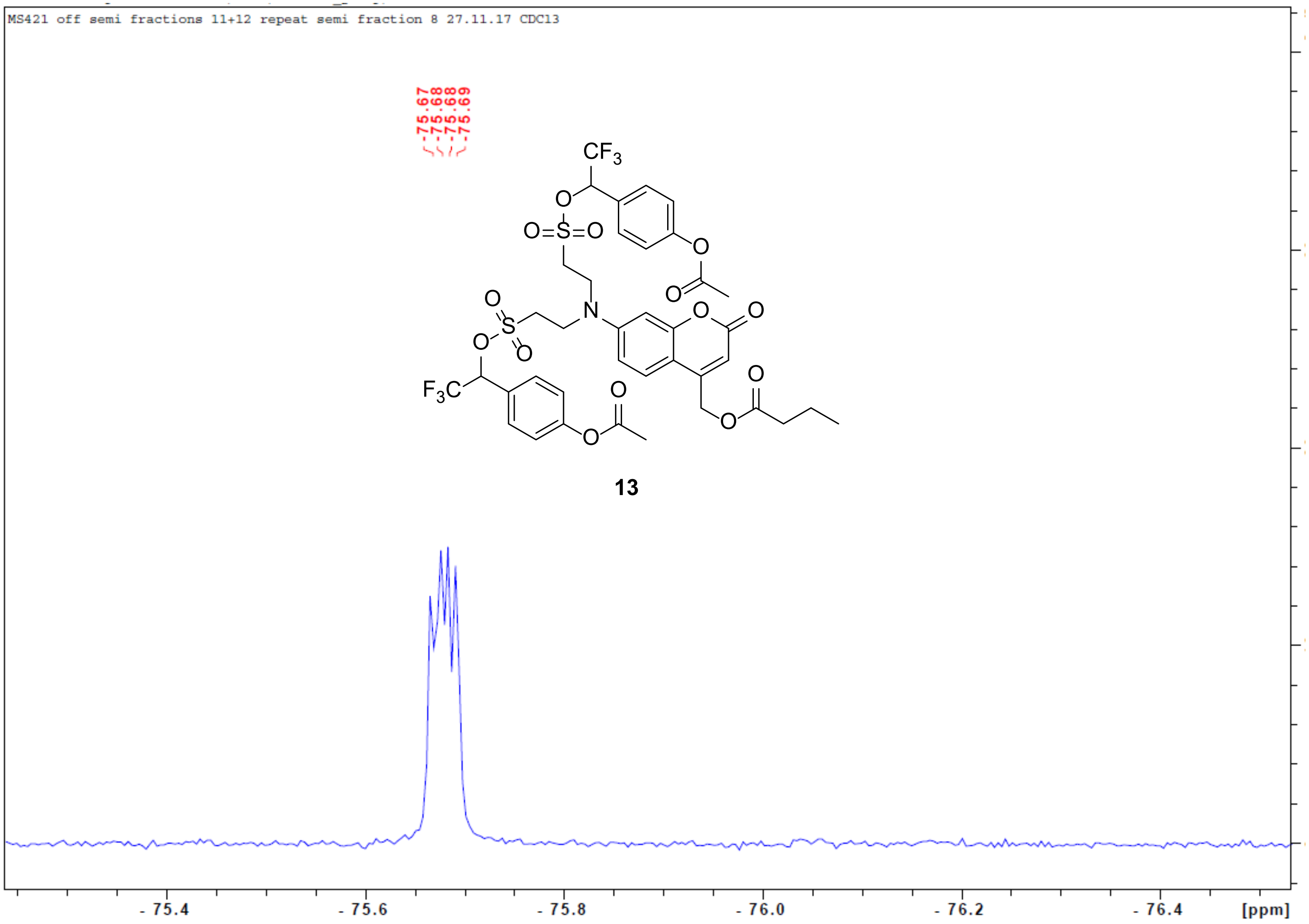

^{19}F NMR spectrum of **13** ($CDCl_3$)

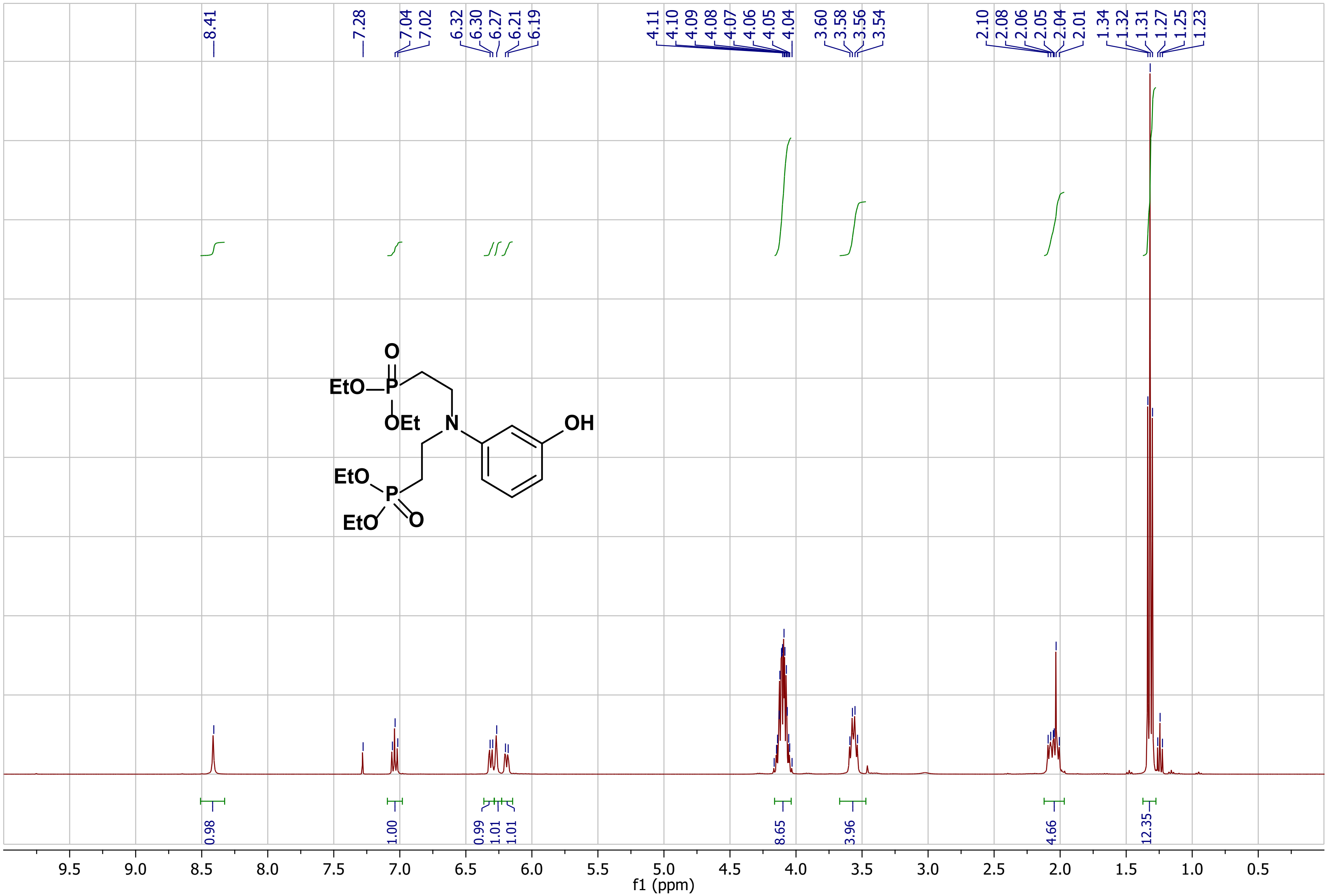

^{1}H NMR spectrum of **15** ($CDCl_3$)

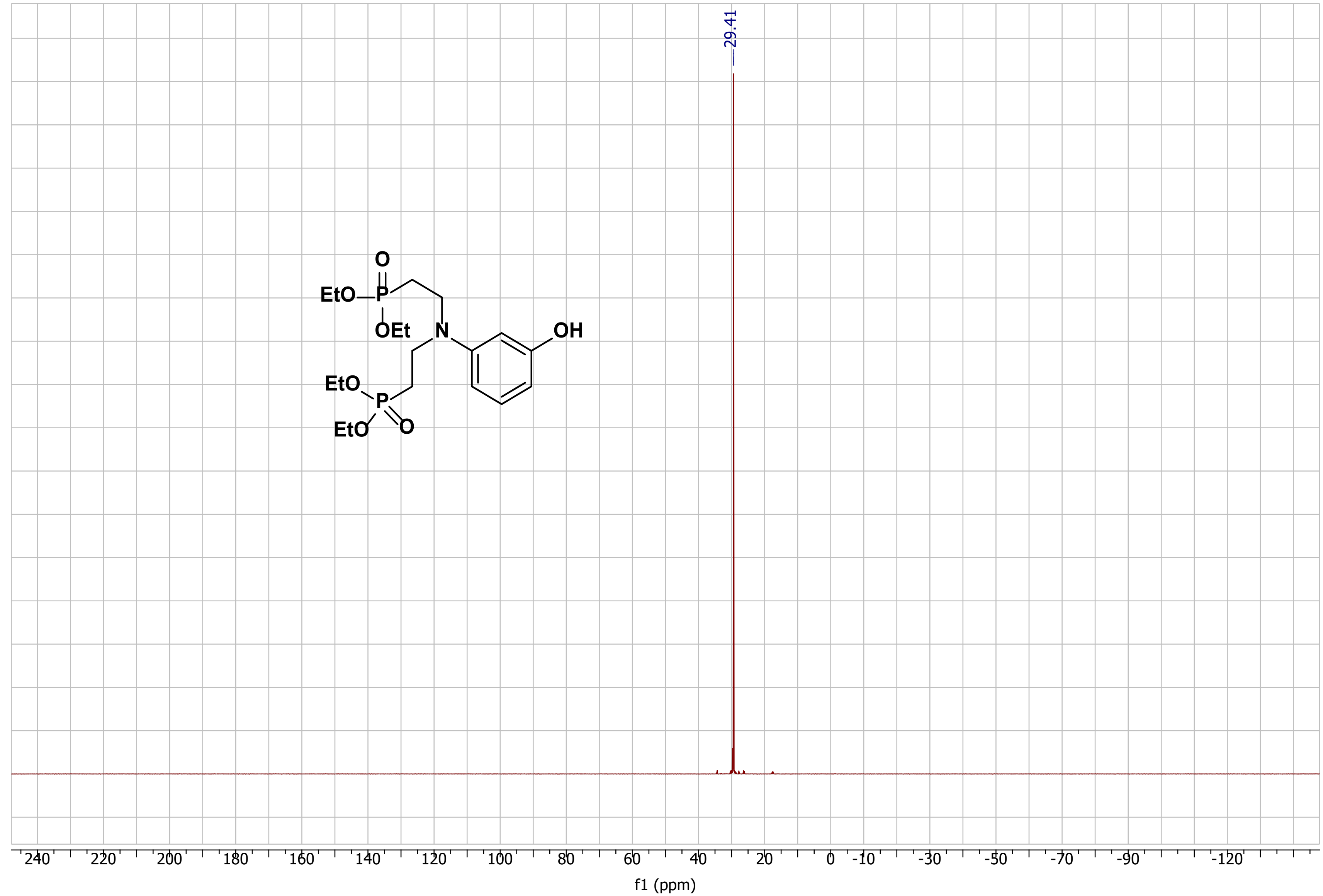

^{31}P NMR spectrum of **15** ($CDCl_3$)

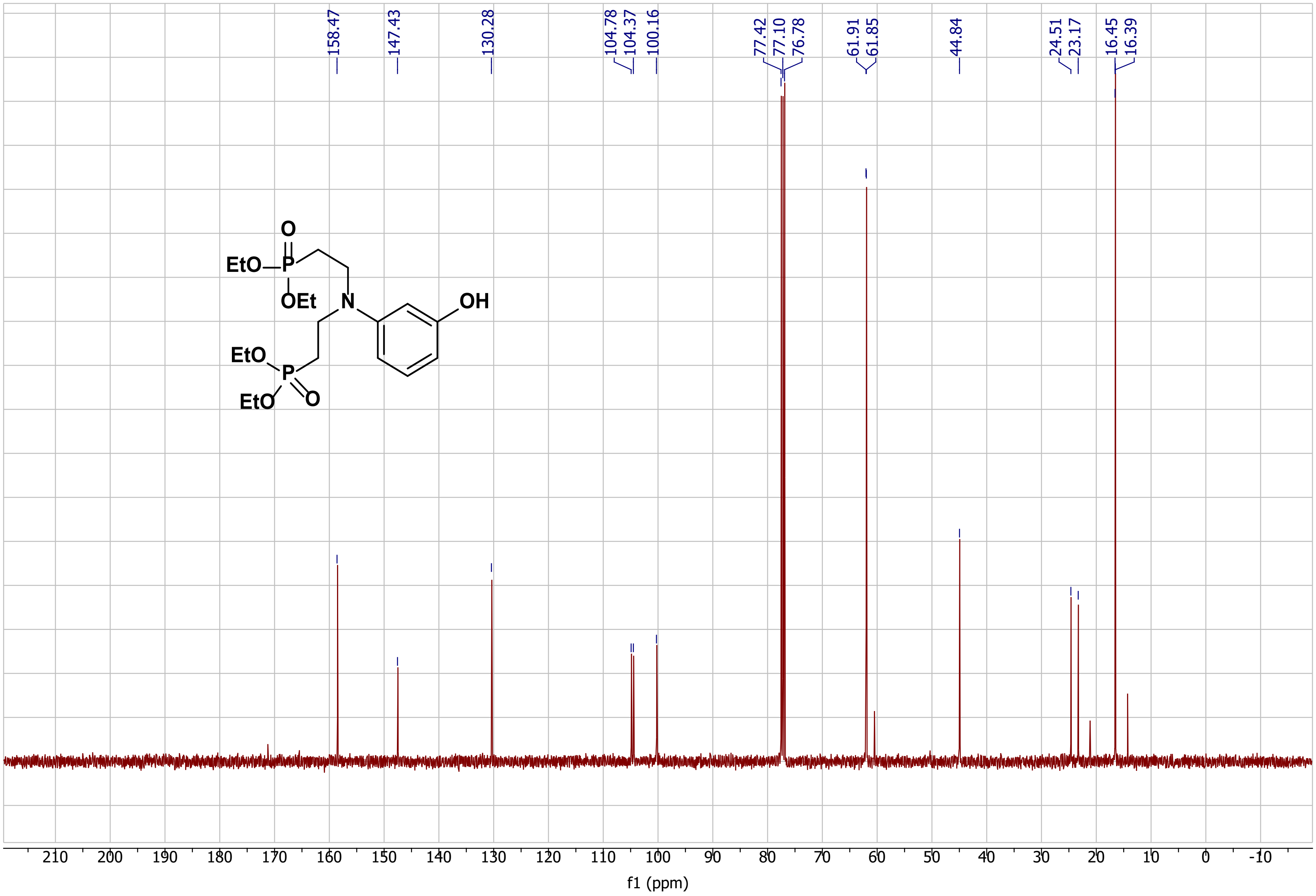

^{13}C NMR spectrum of **15** ($CDCl_3$)

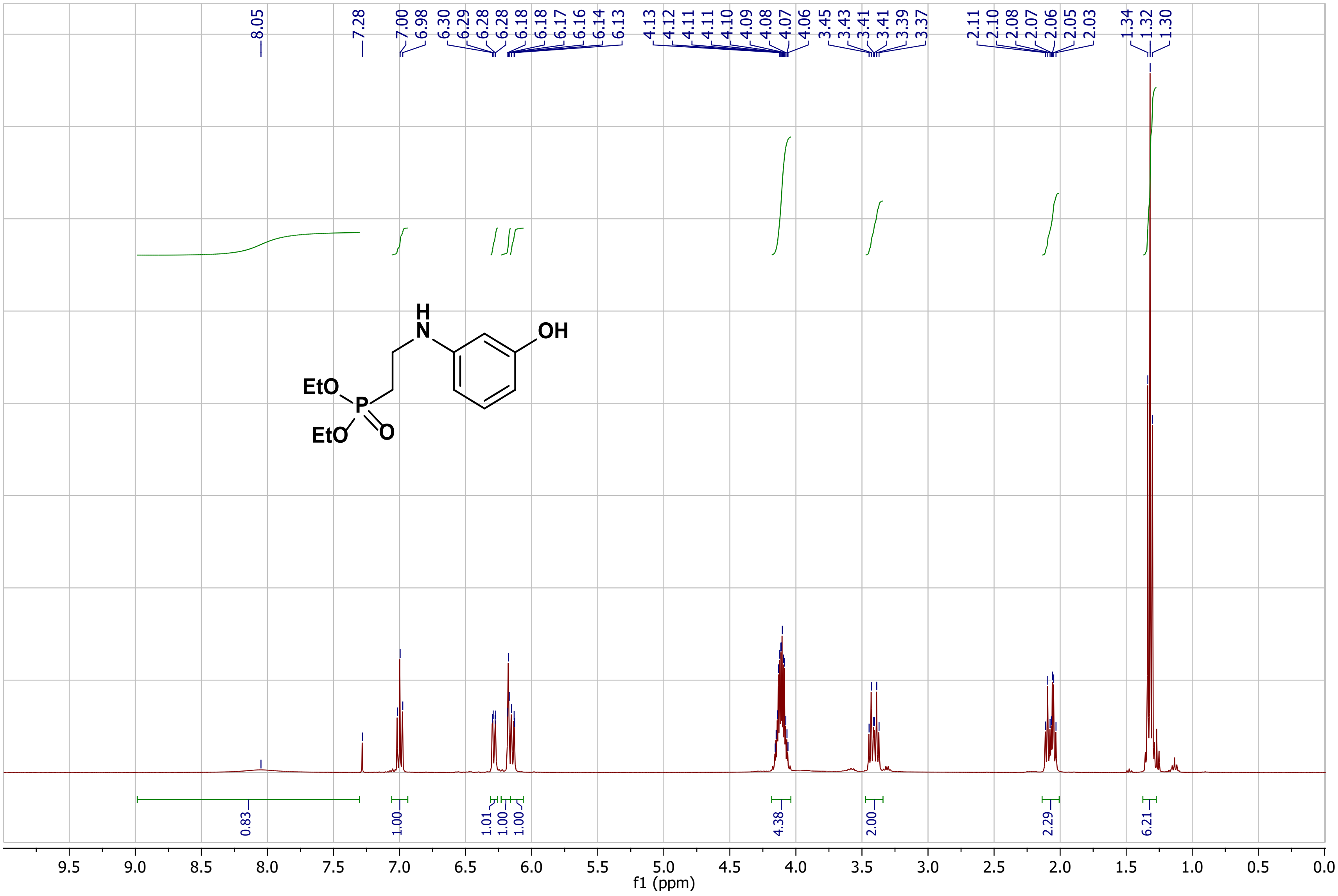

^{1}H NMR spectrum of **22** ($CDCl_3$)

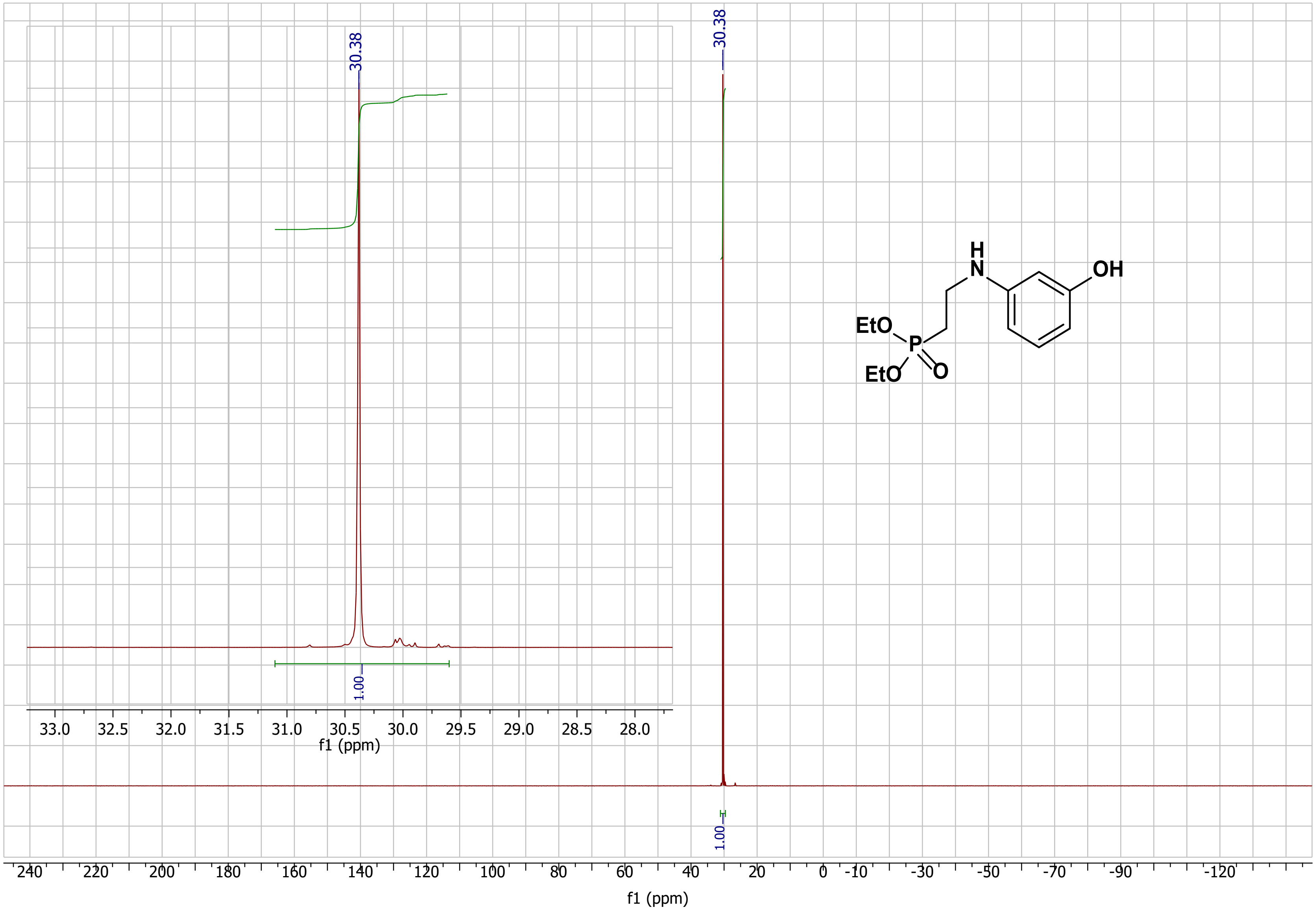

^{31}P NMR spectrum of **22** ($CDCl_3$)

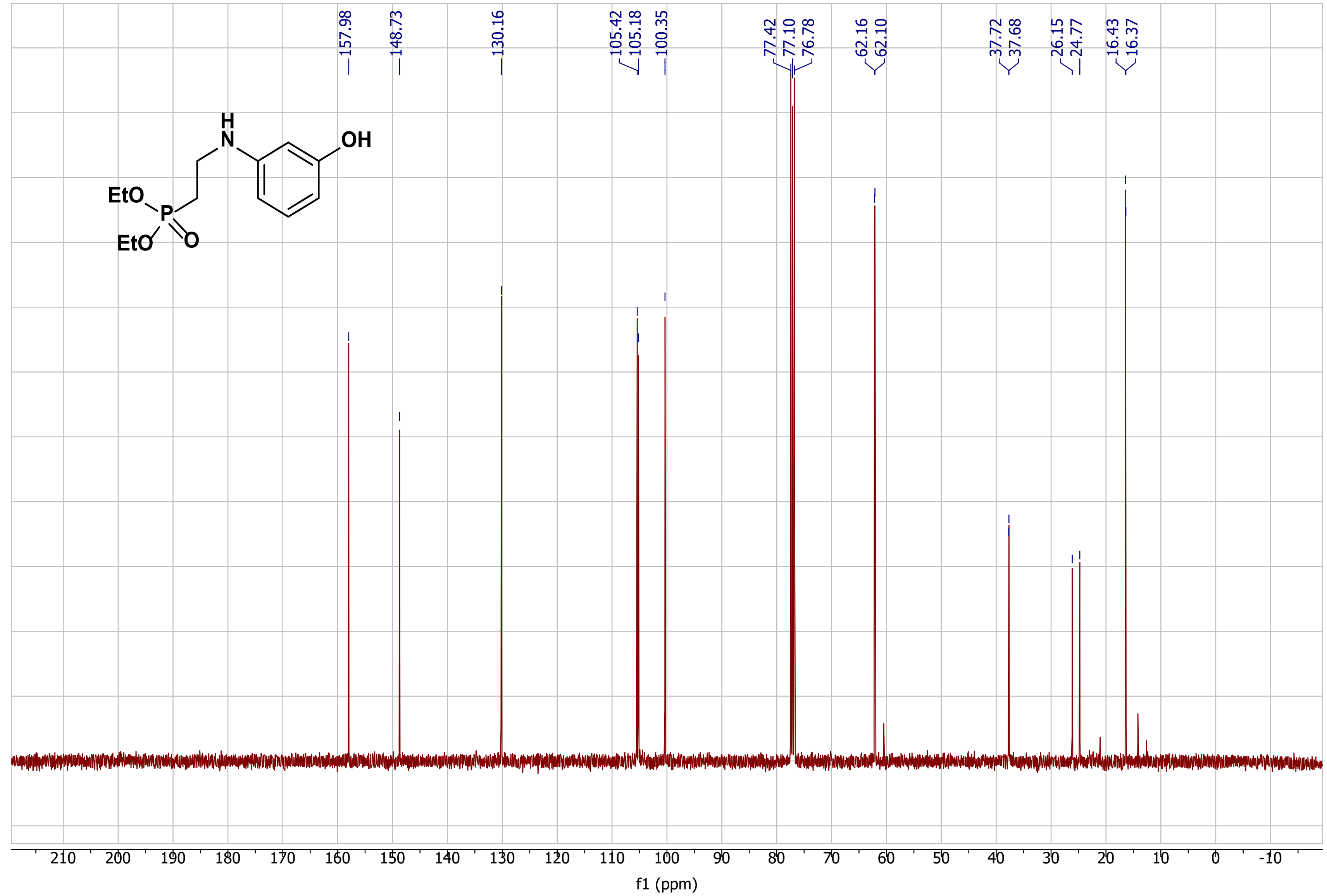

^{13}C NMR spectrum of **22** ($CDCl_3$)

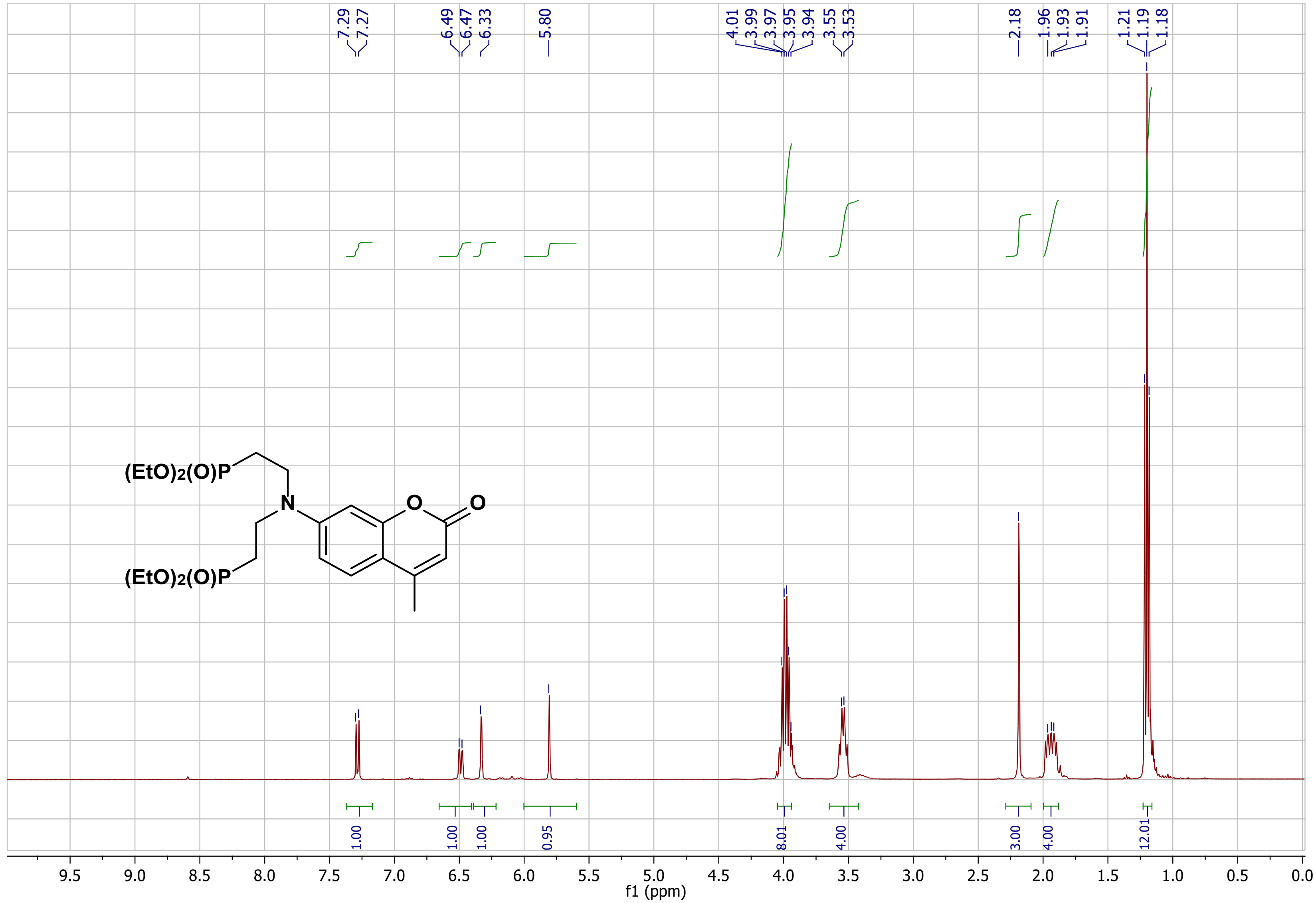

^{1}H NMR spectrum of **16** ($CDCl_3$)

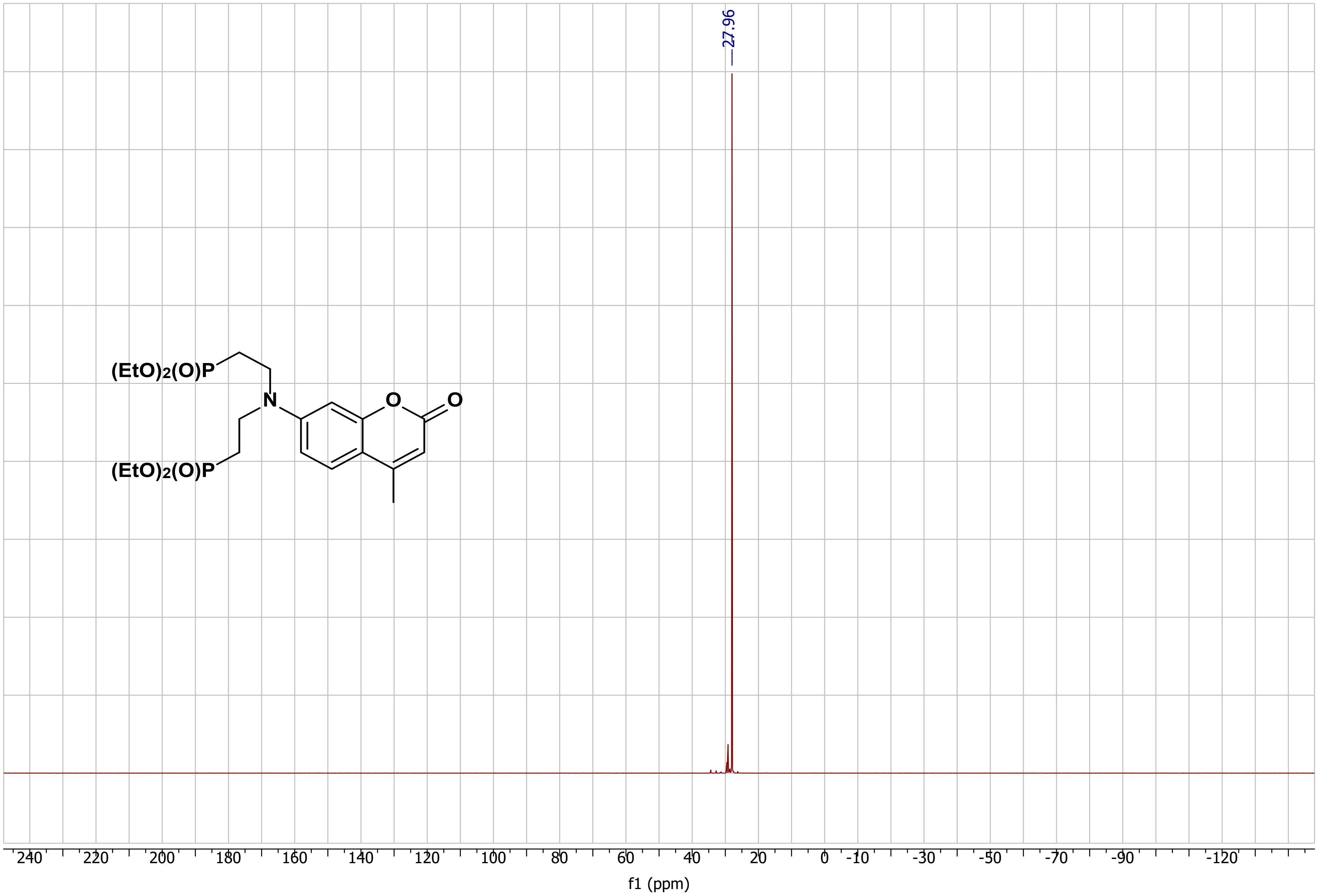

^{31}P NMR spectrum of **16** ($CDCl_3$)

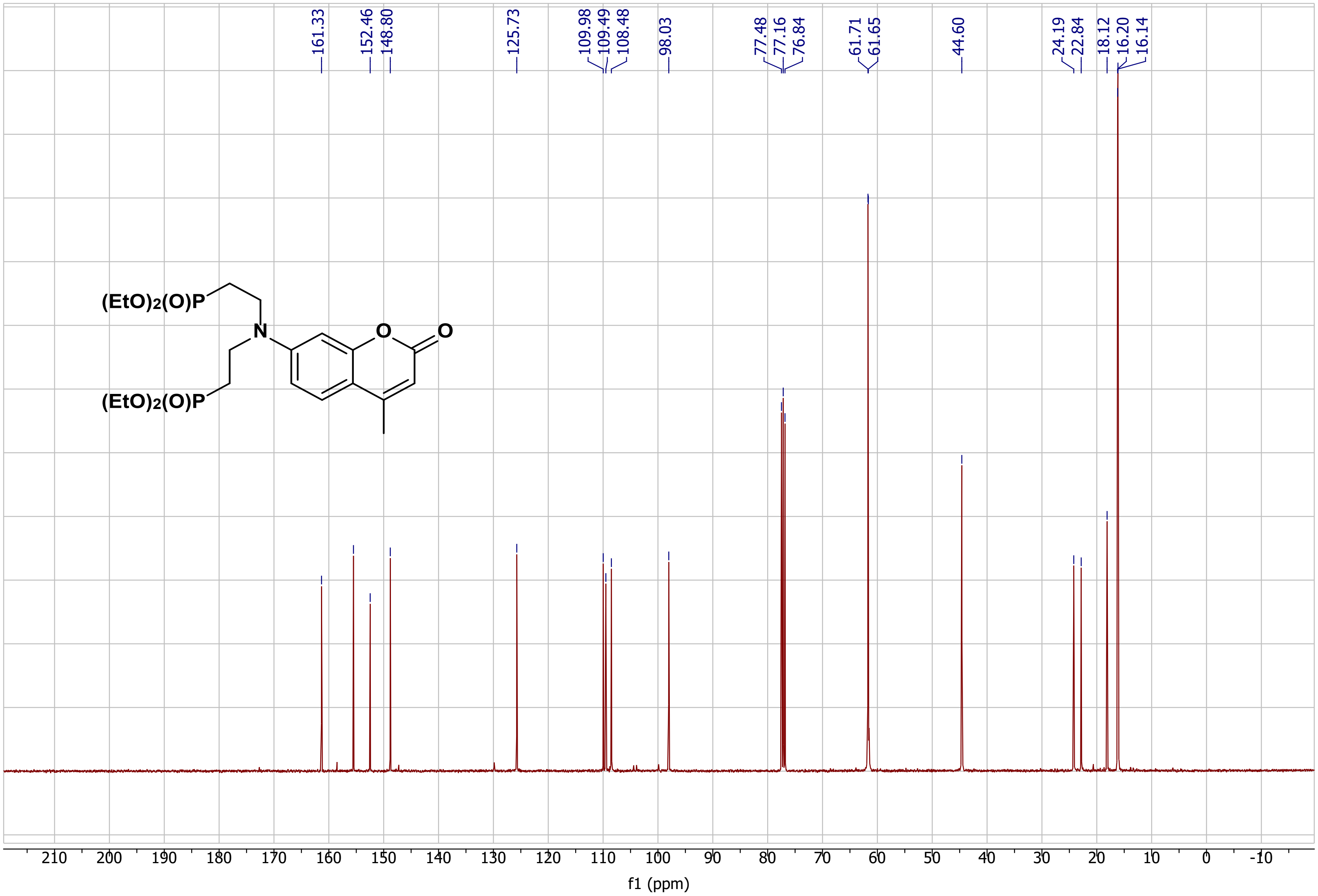

^{13}C NMR spectrum of **16** ($CDCl_3$)

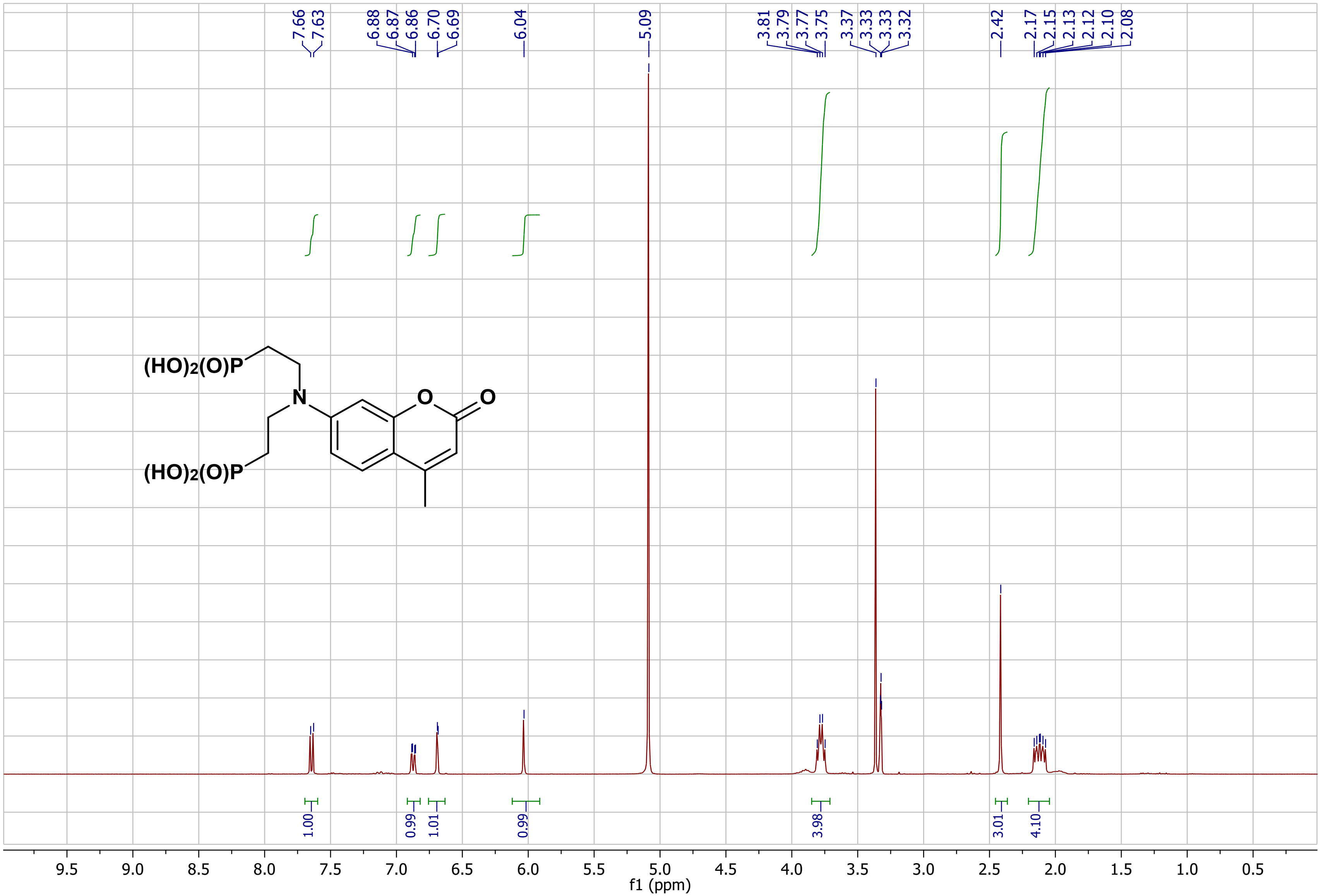

^{1}H NMR spectrum of **36** (CD_3OD)

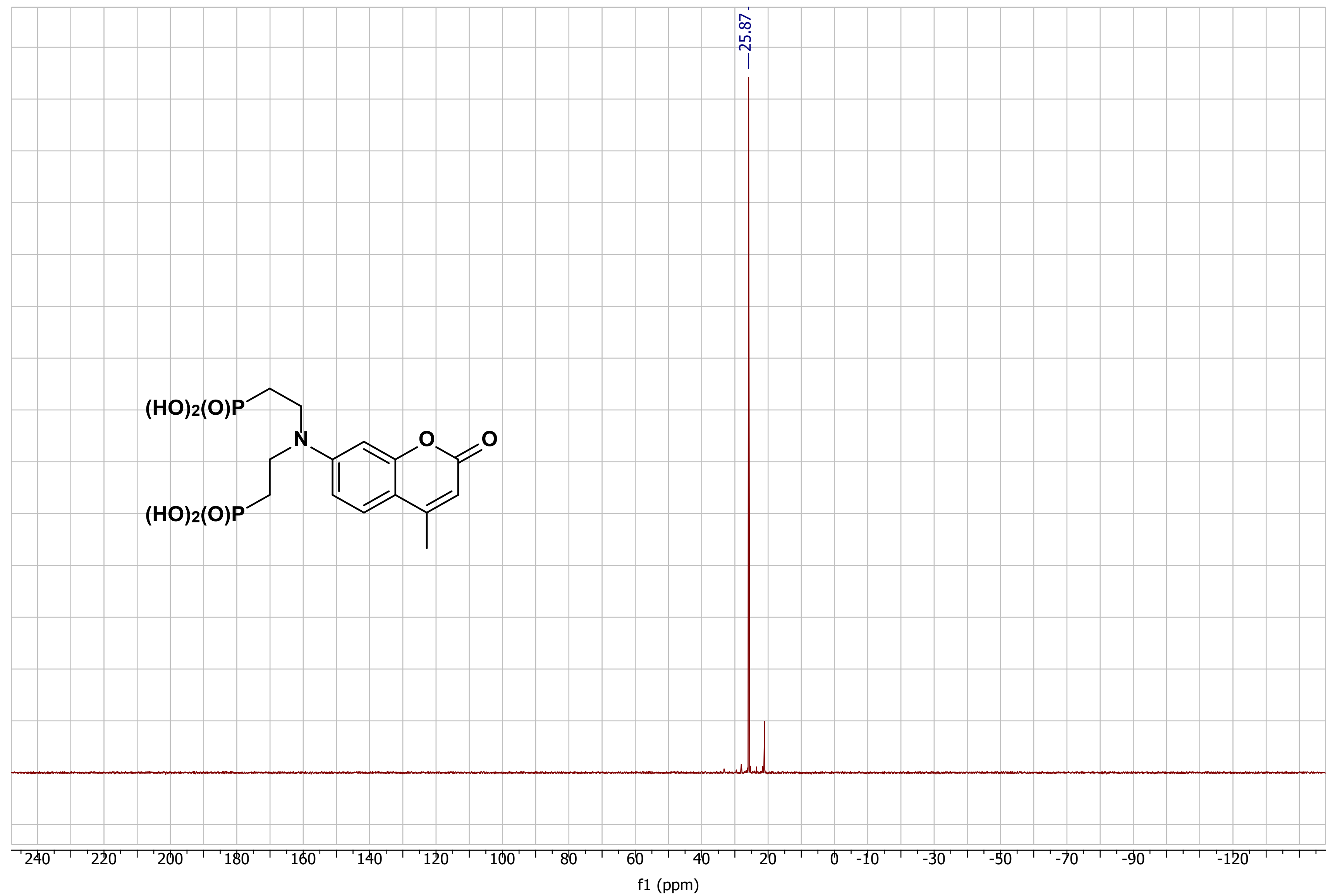

^{31}P NMR spectrum of **36** (CD_3OD)

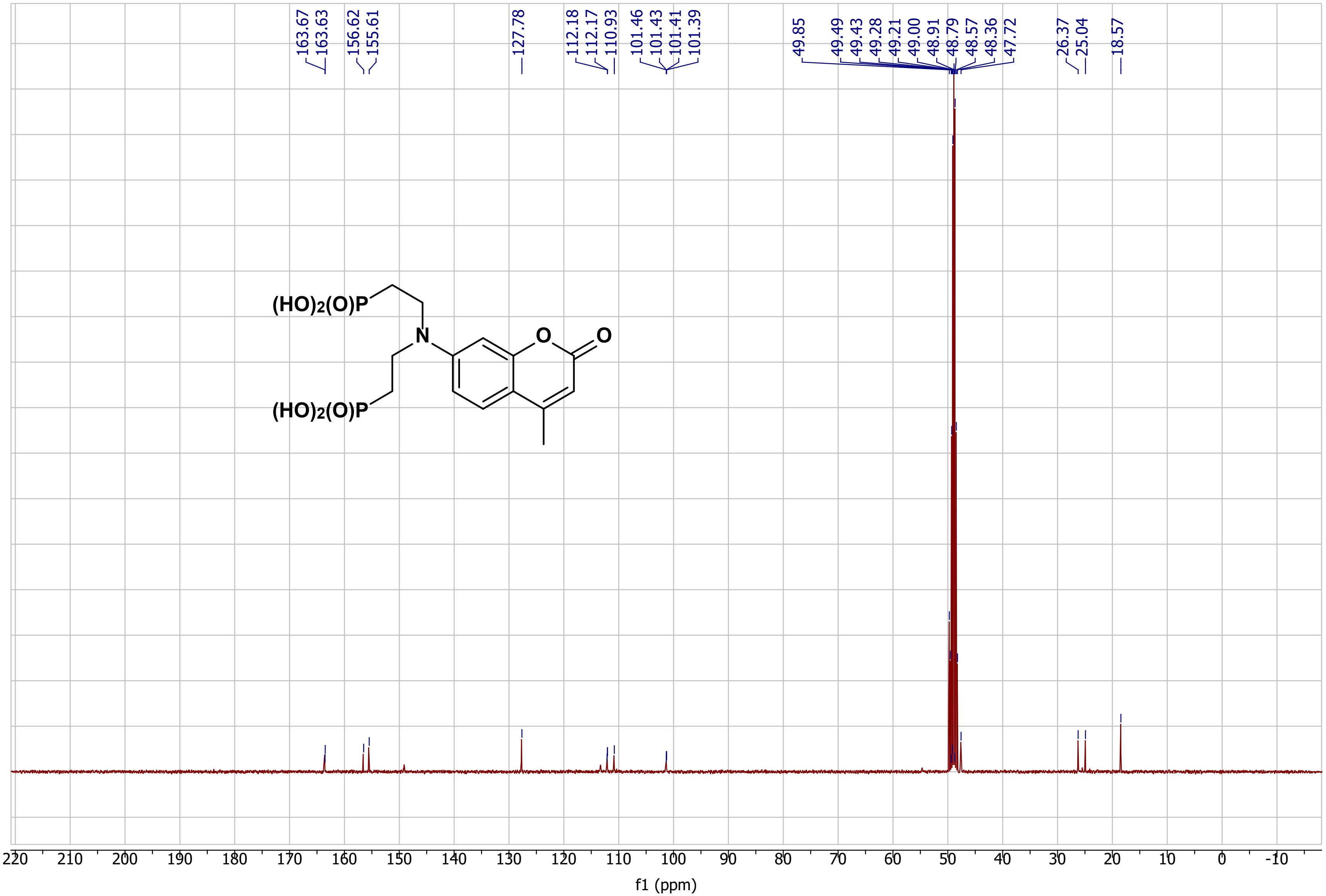

^{13}C NMR spectrum of **36** (CD_3OD)

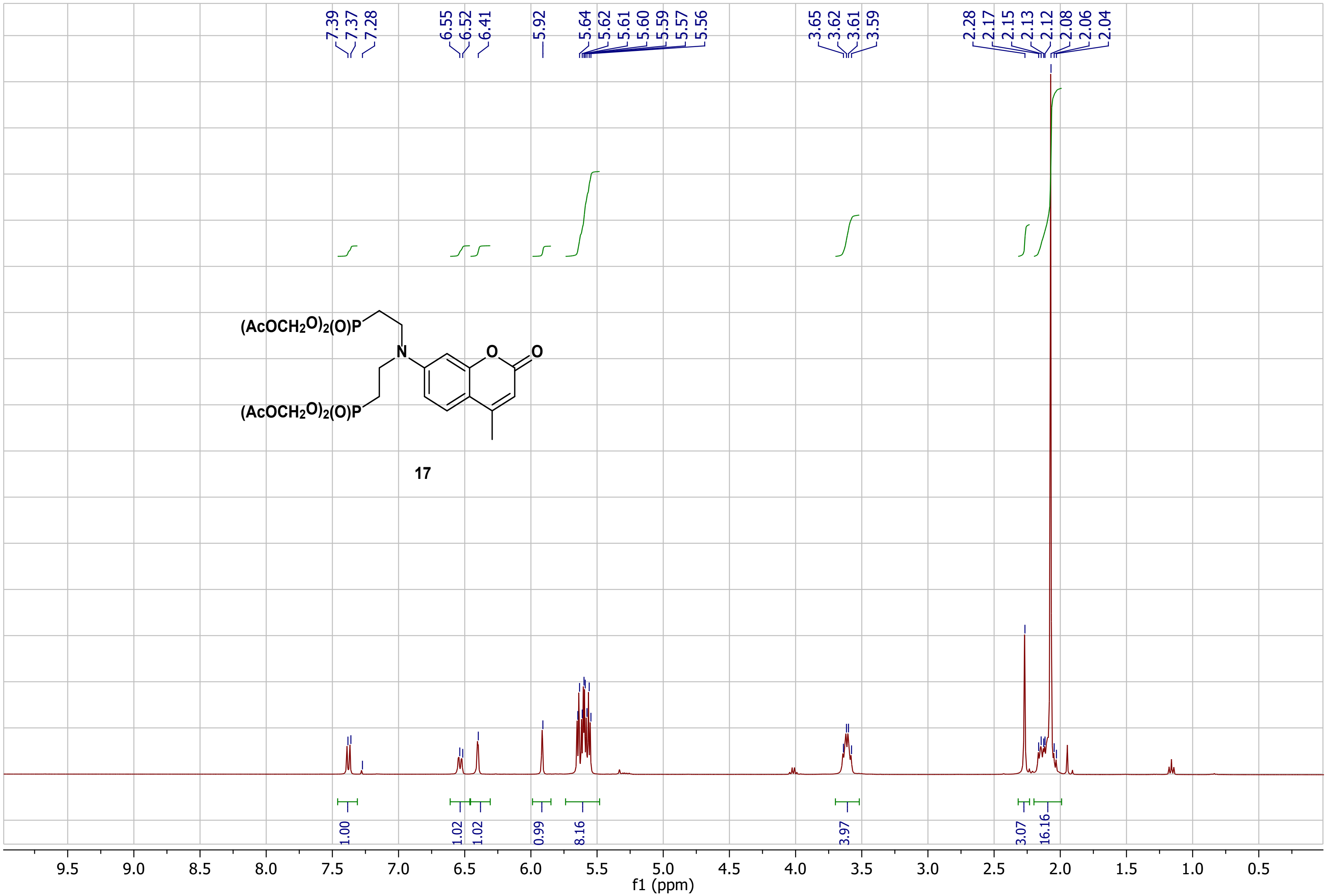

^{1}H NMR spectrum of **17** ($CDCl_3$)

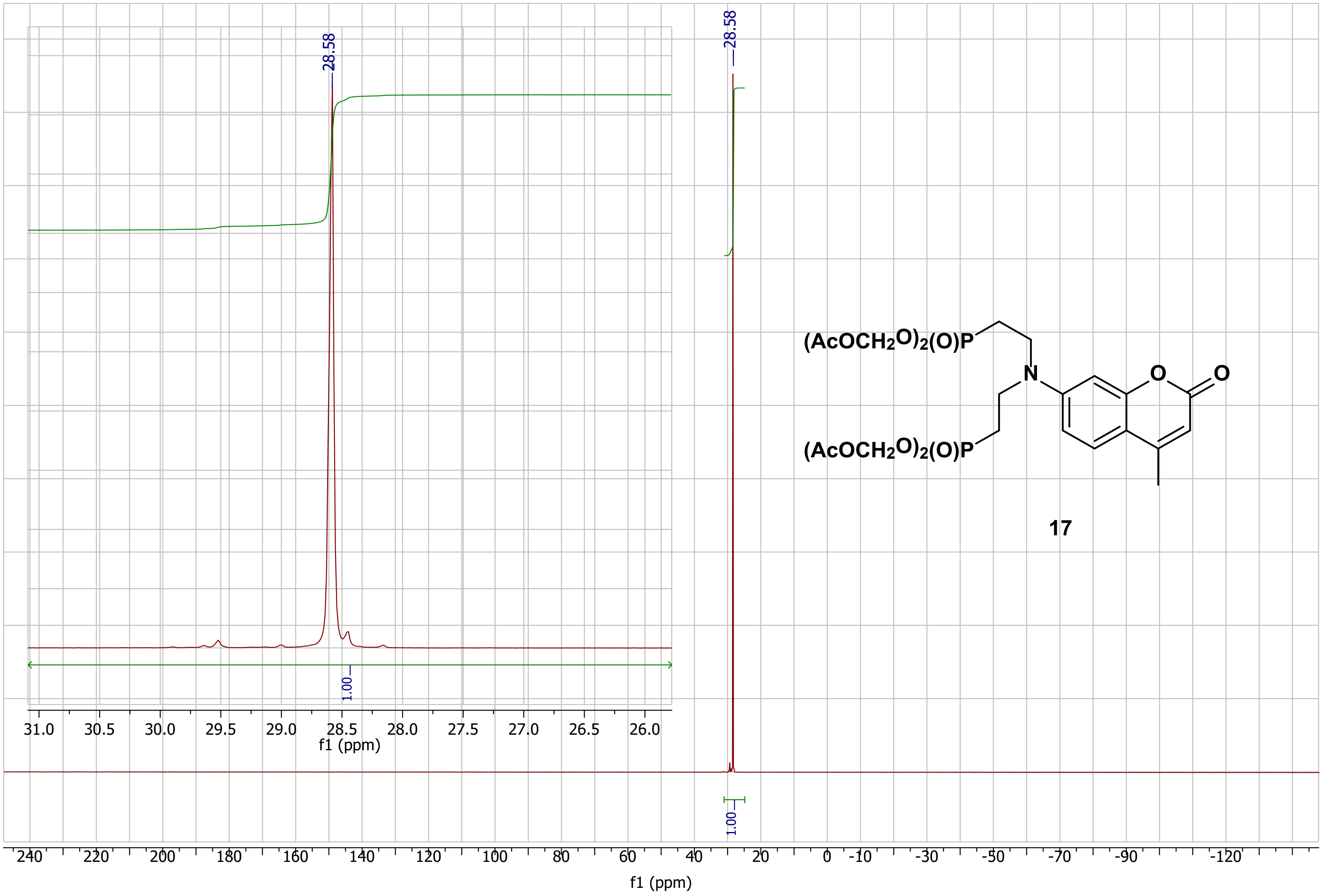

^{31}P NMR spectrum of **17** ($CDCl_3$)

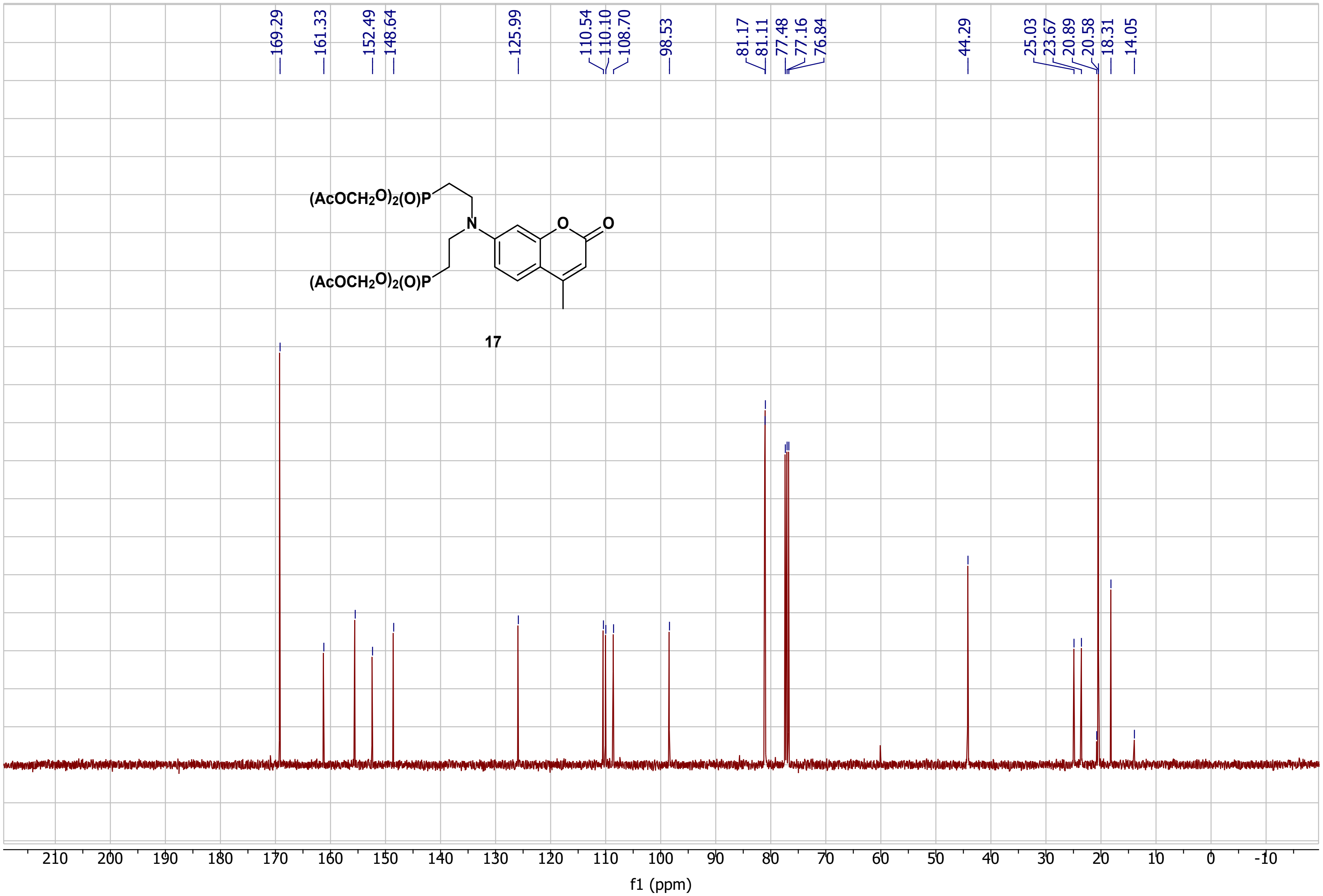

^{13}C NMR spectrum of **17** ($CDCl_3$)

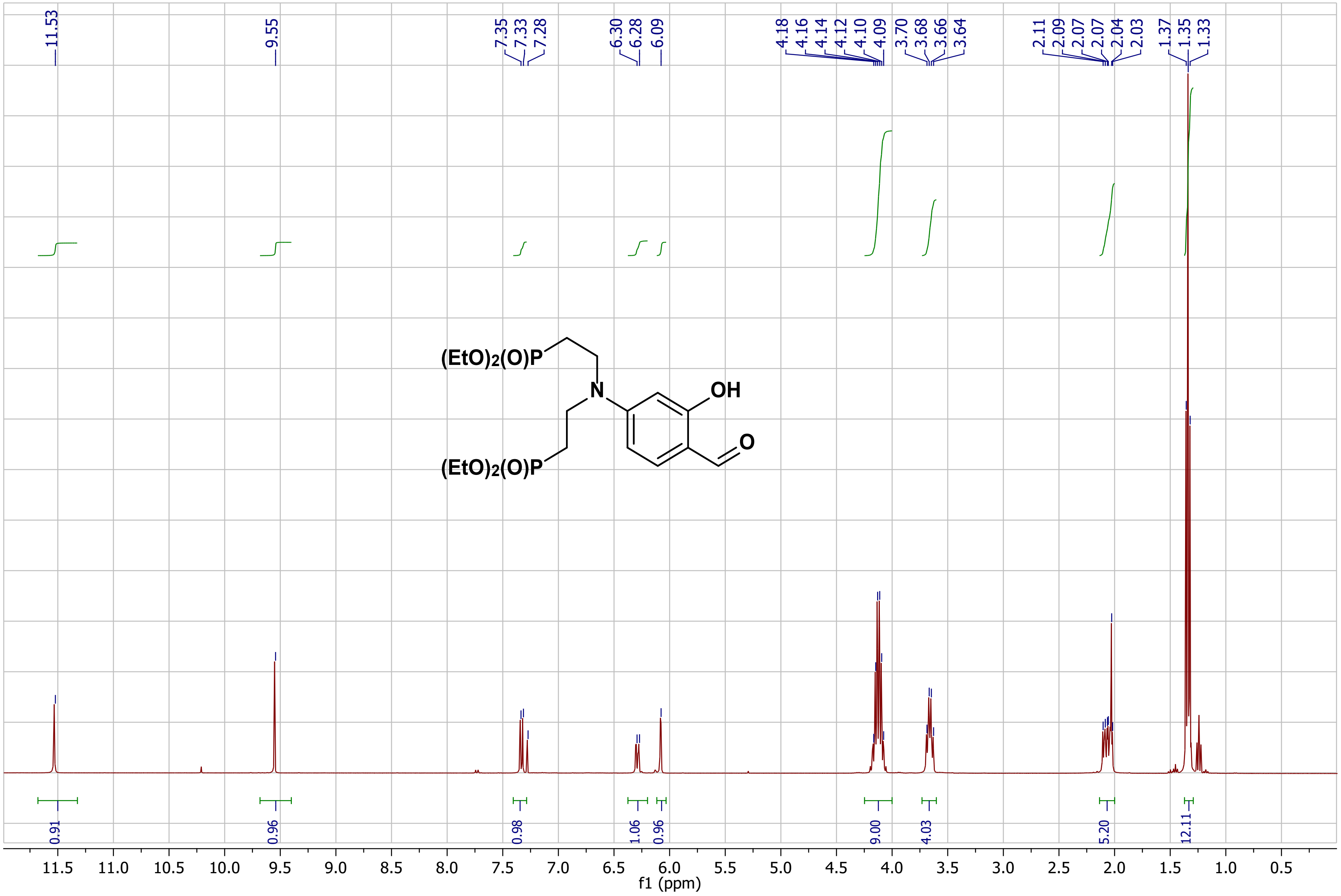

^{1}H NMR spectrum of **18** ($CDCl_3$)

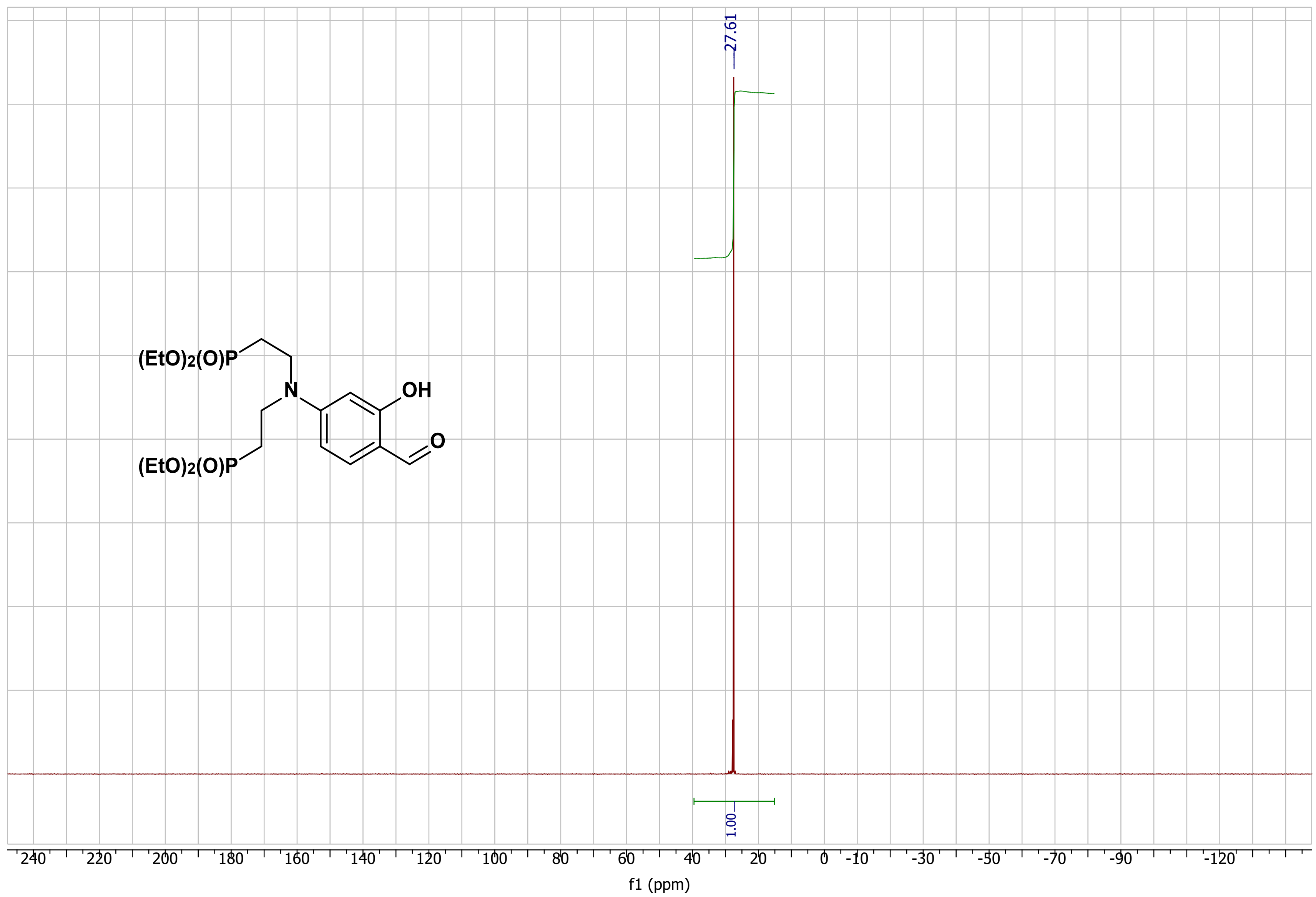

^{31}P NMR spectrum of **18** ($CDCl_3$)

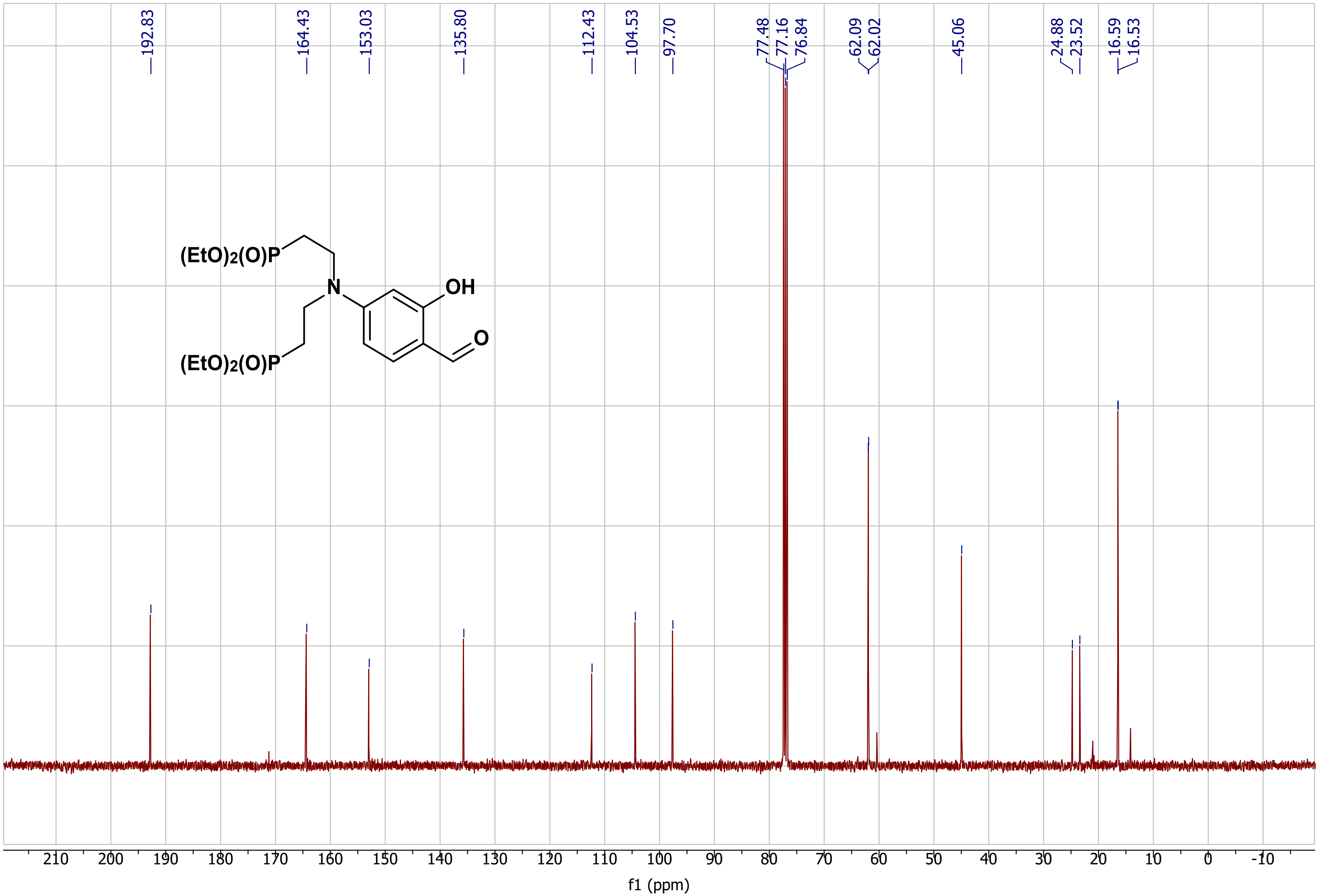

^{13}C NMR spectrum of **18** ($CDCl_3$)

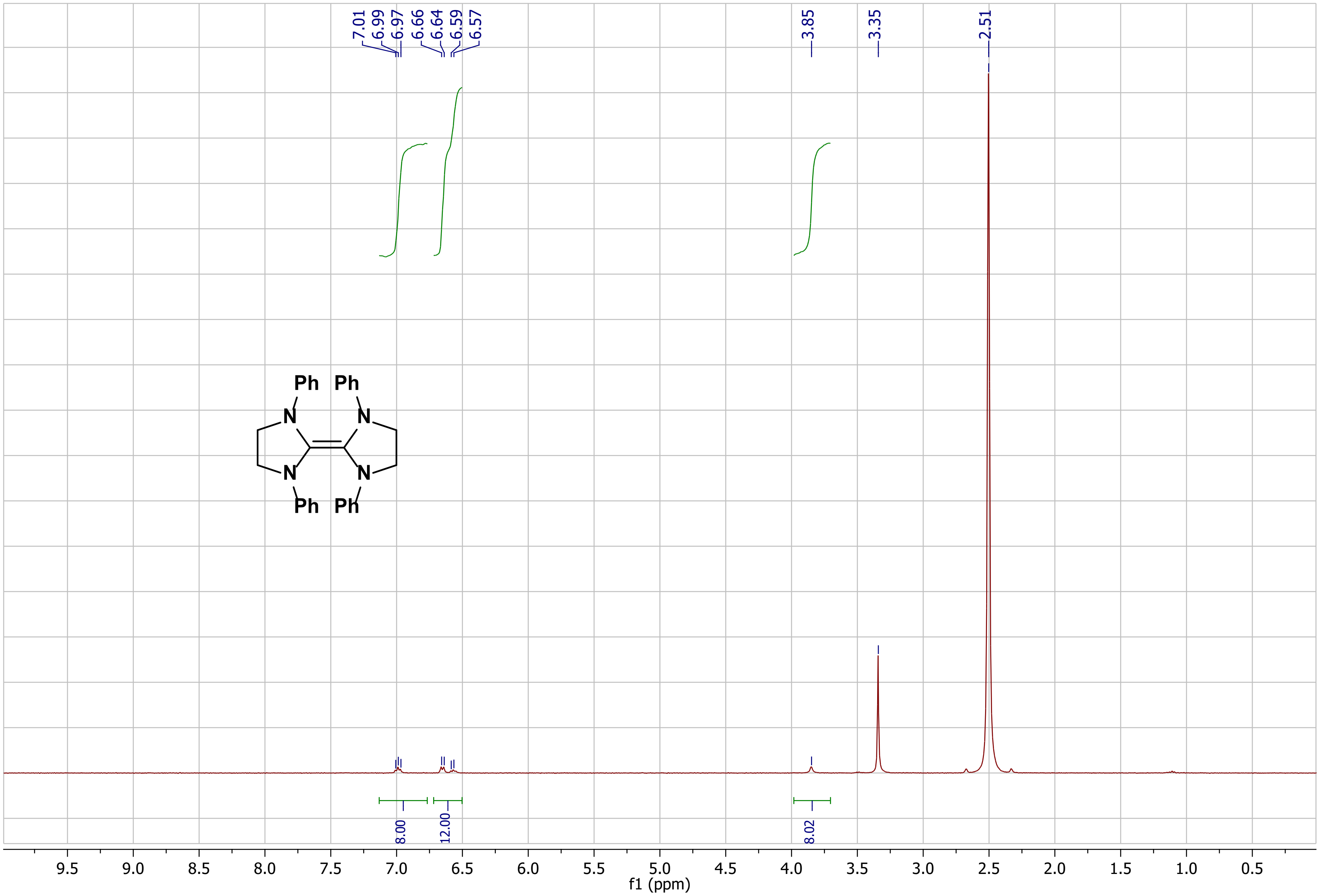

^1H NMR spectrum of **RM552** (DMSO-d6)

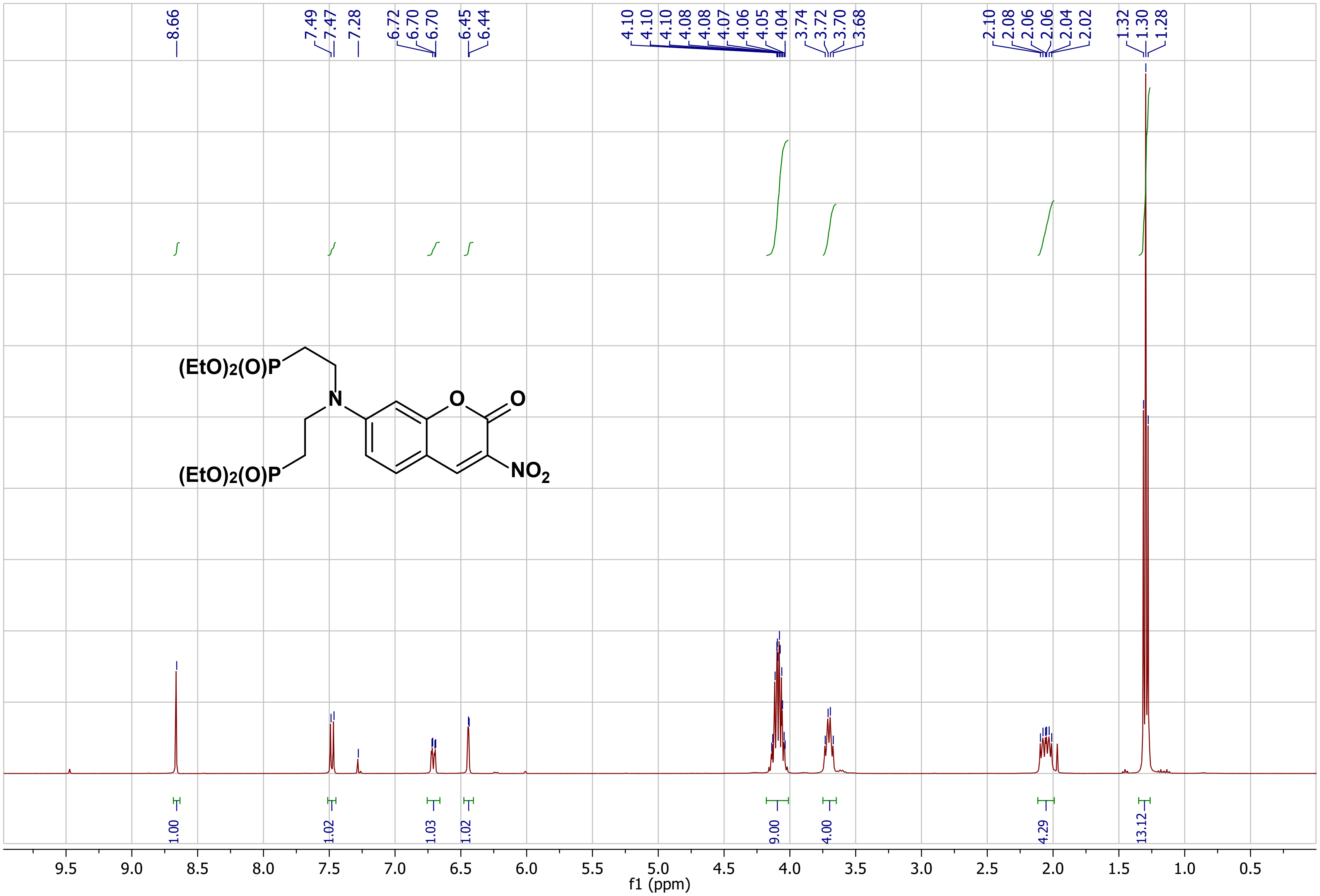

^{1}H NMR spectrum of **19** ($CDCl_3$)

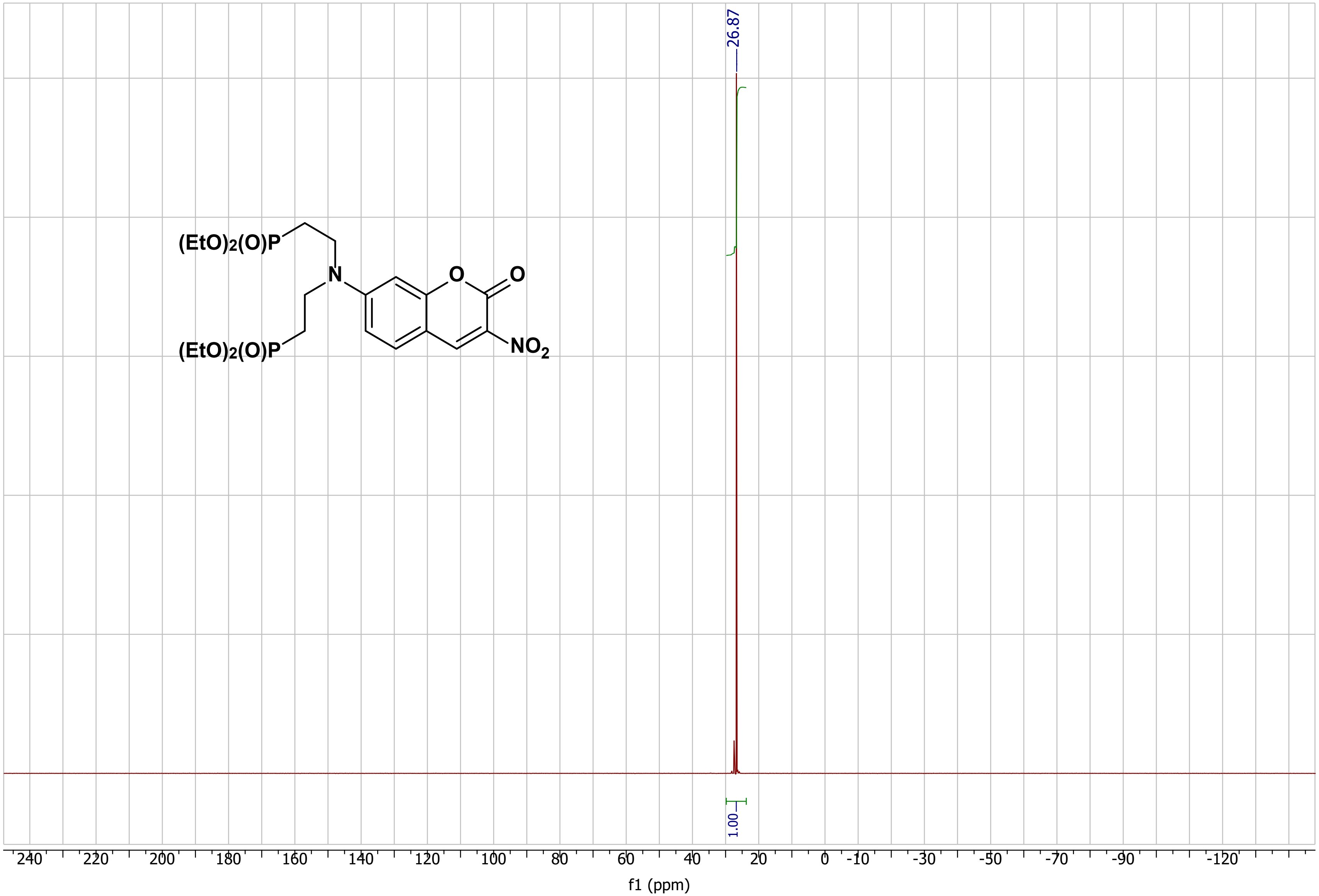

^{31}P NMR spectrum of **19** ($CDCl_3$)

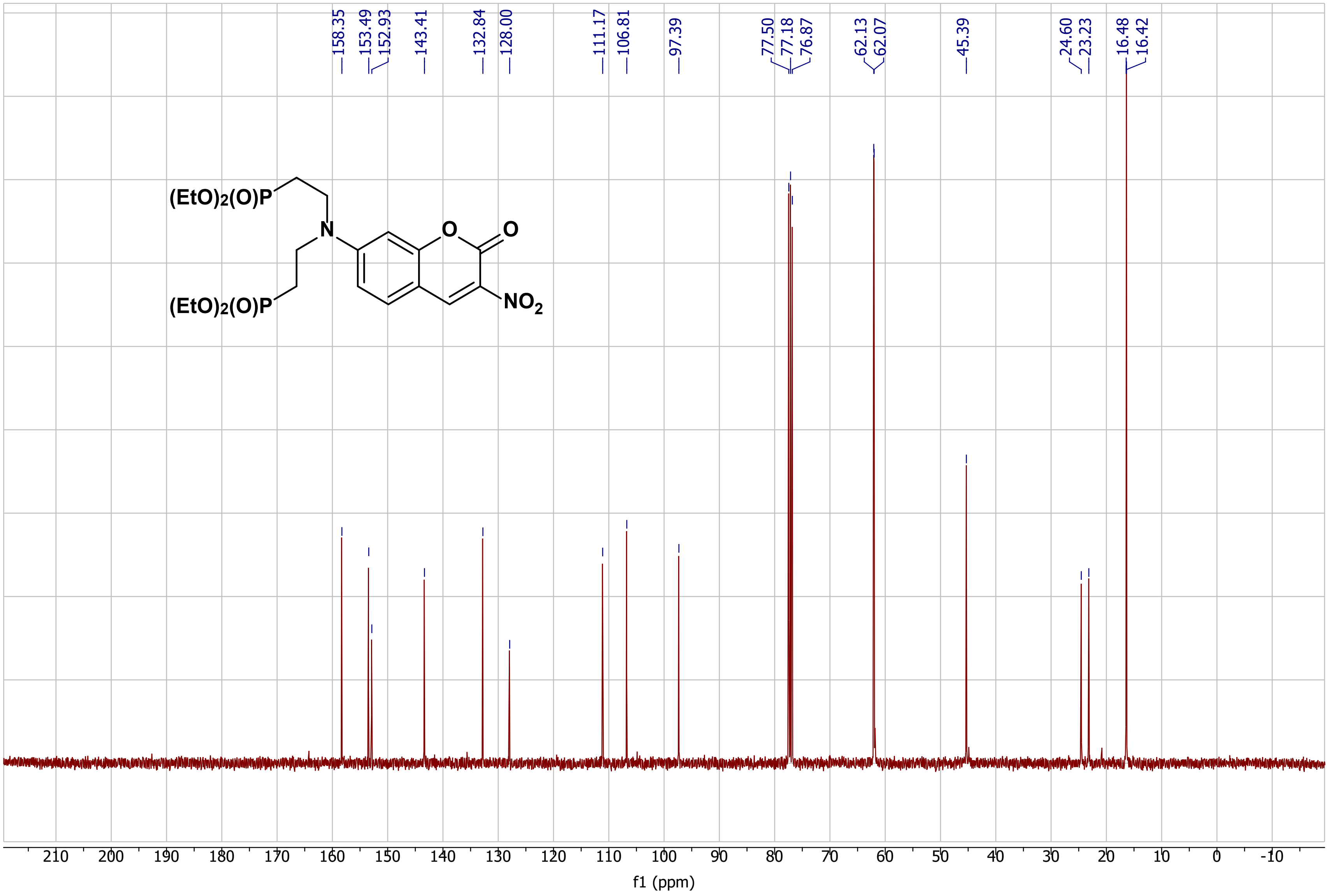

^{13}C NMR spectrum of **19** ($CDCl_3$)

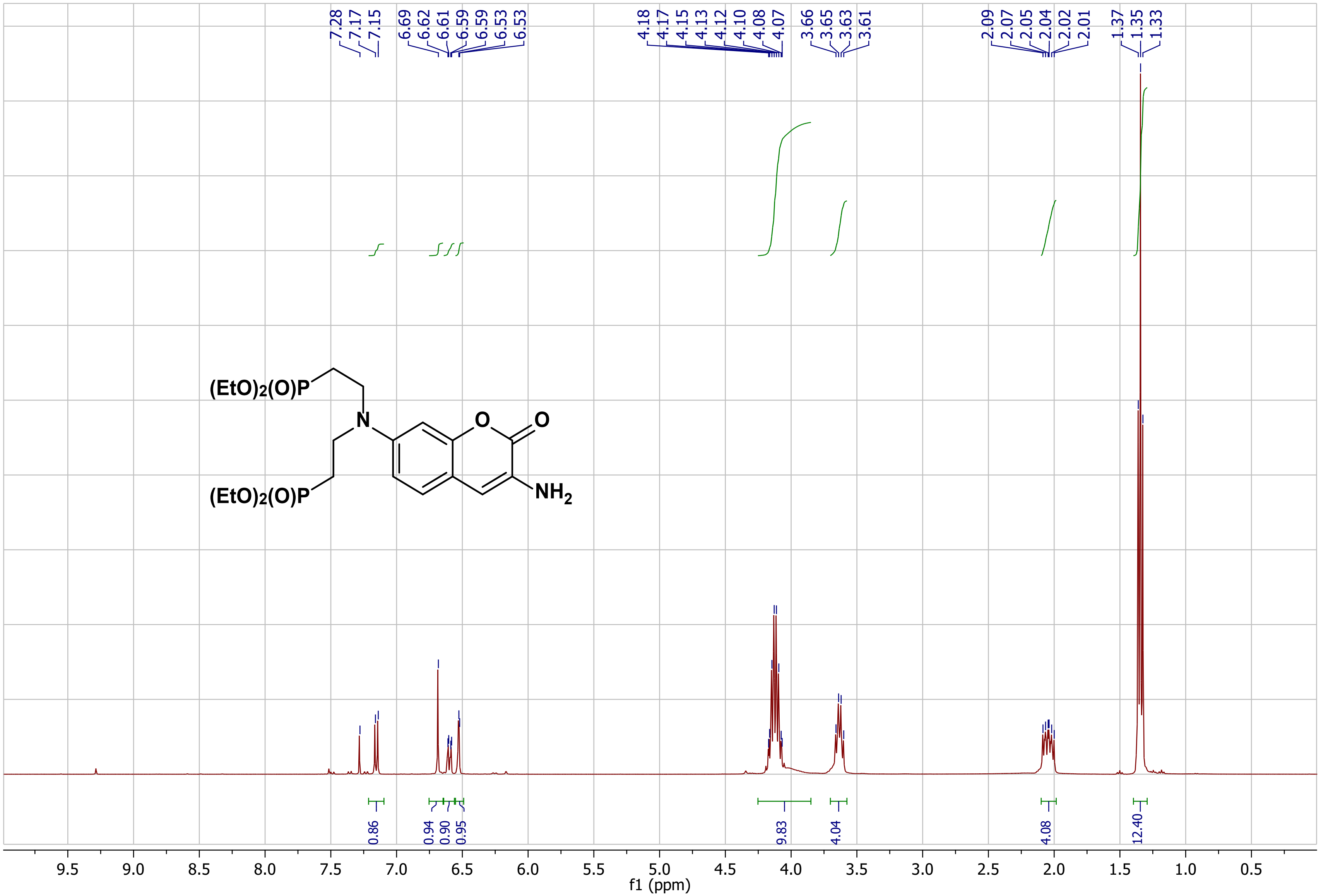

^{1}H NMR spectrum of **37** ($CDCl_3$)

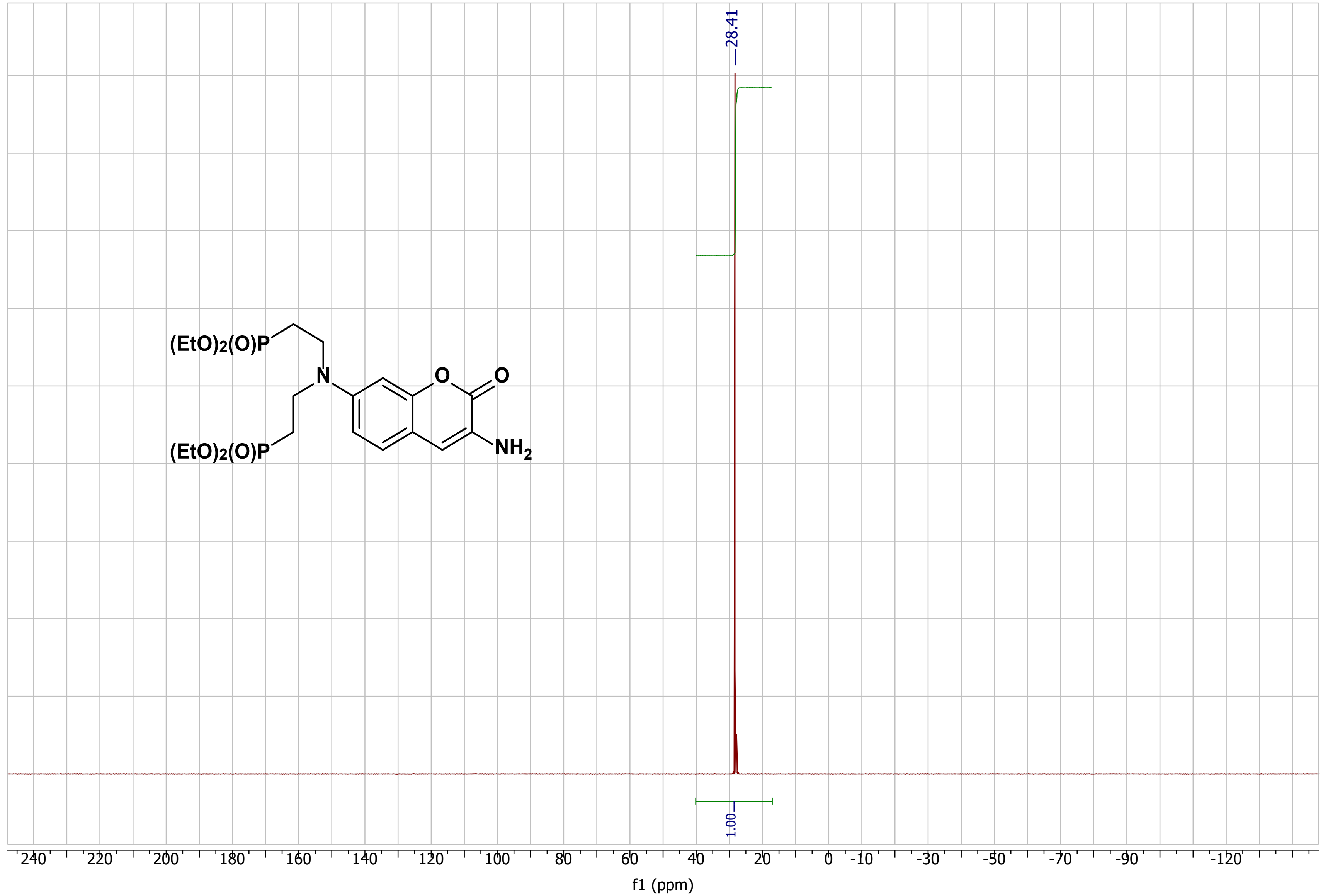

^{31}P NMR spectrum of **37** ($CDCl_3$)

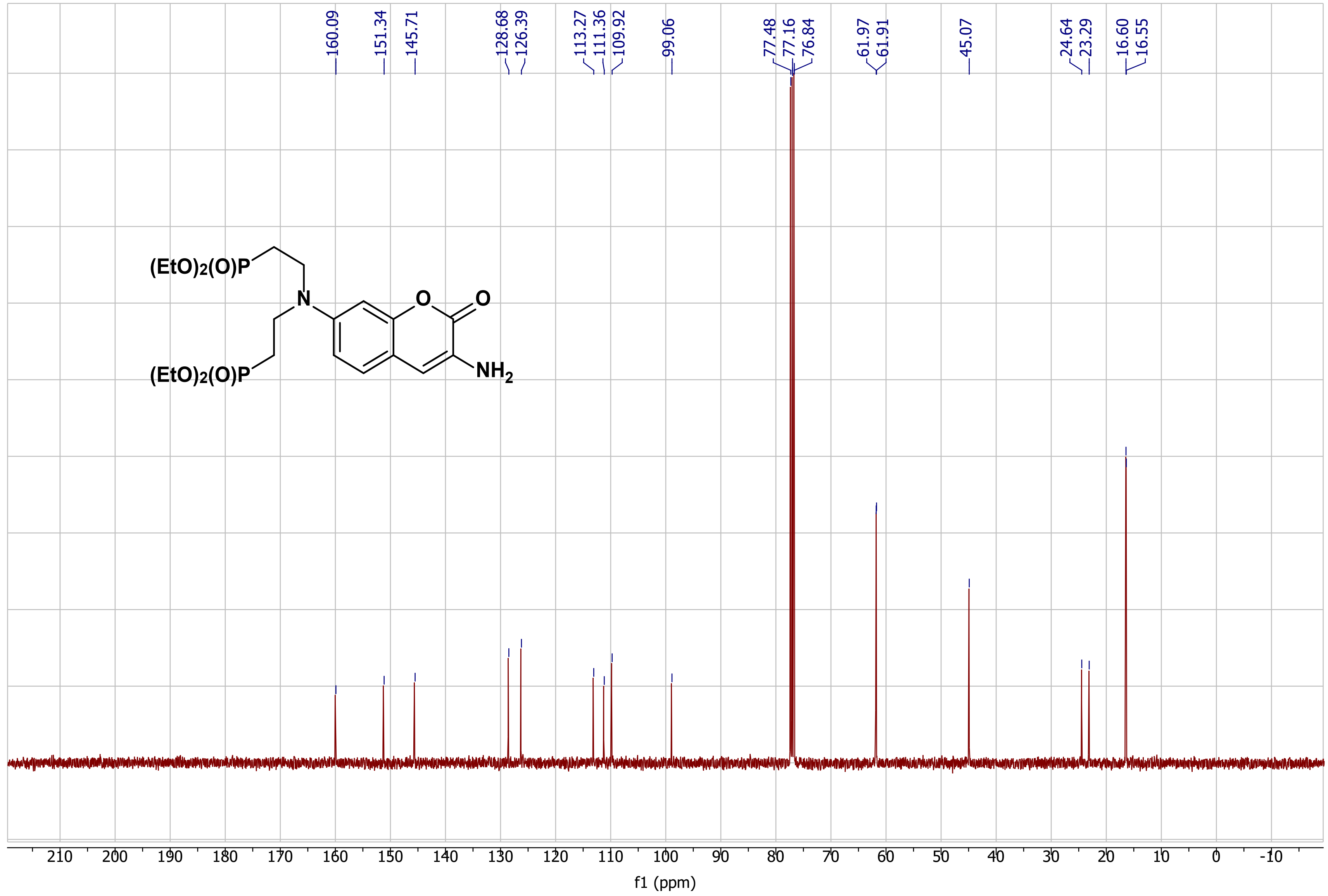

^{13}C NMR spectrum of **37** ($CDCl_3$)

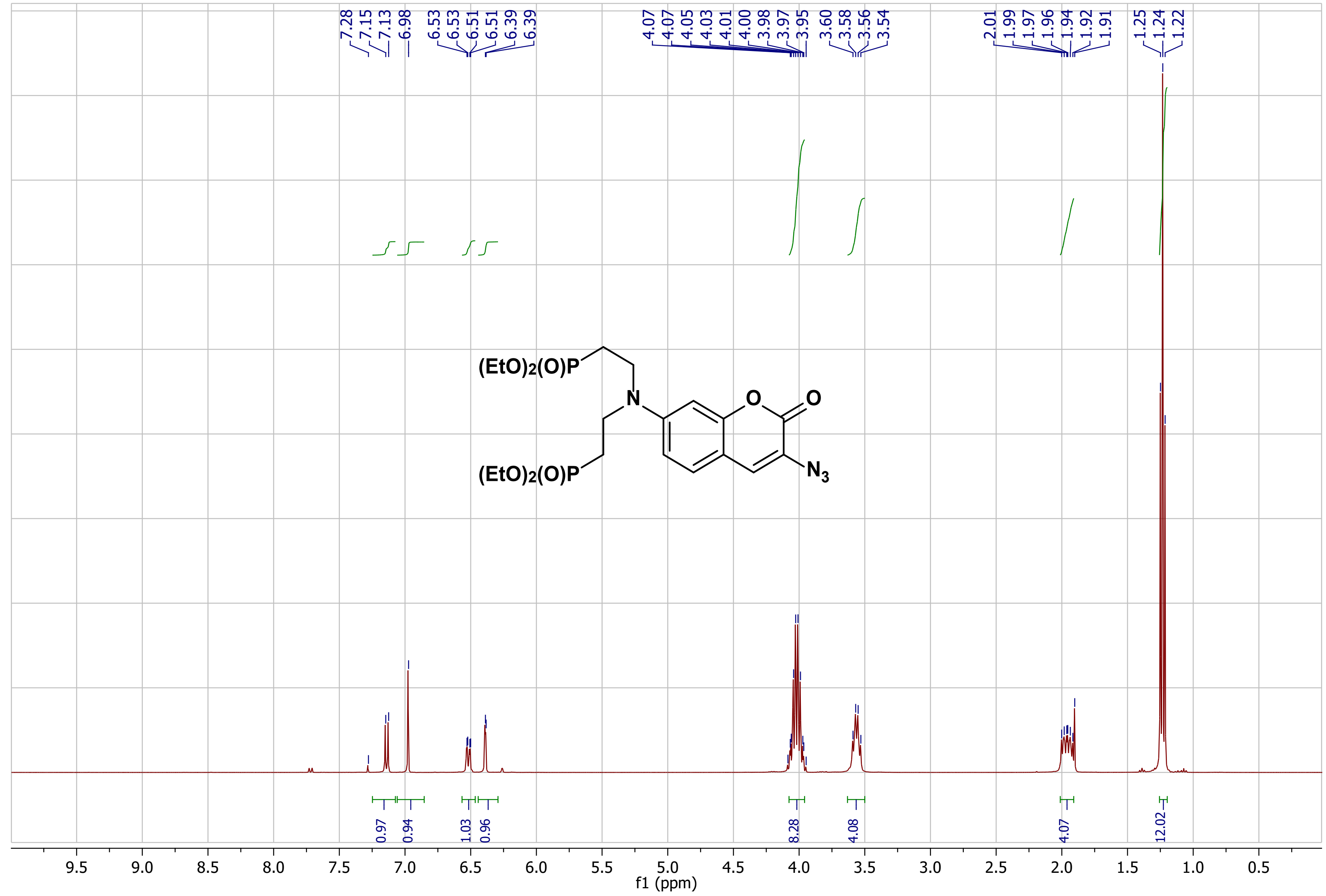

^{1}H NMR spectrum of **20** ($CDCl_3$)

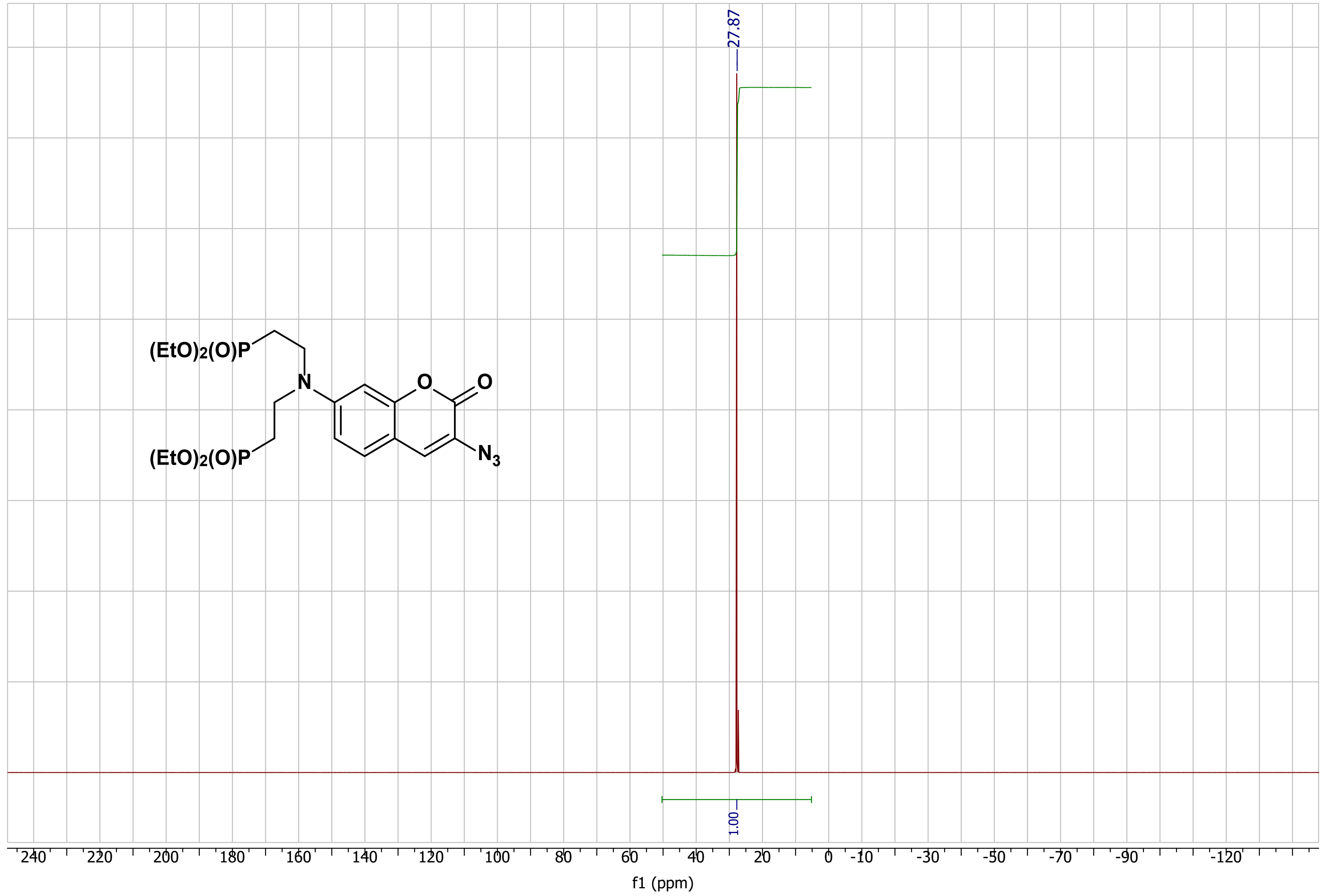

^{31}P NMR spectrum of **20** ($CDCl_3$)

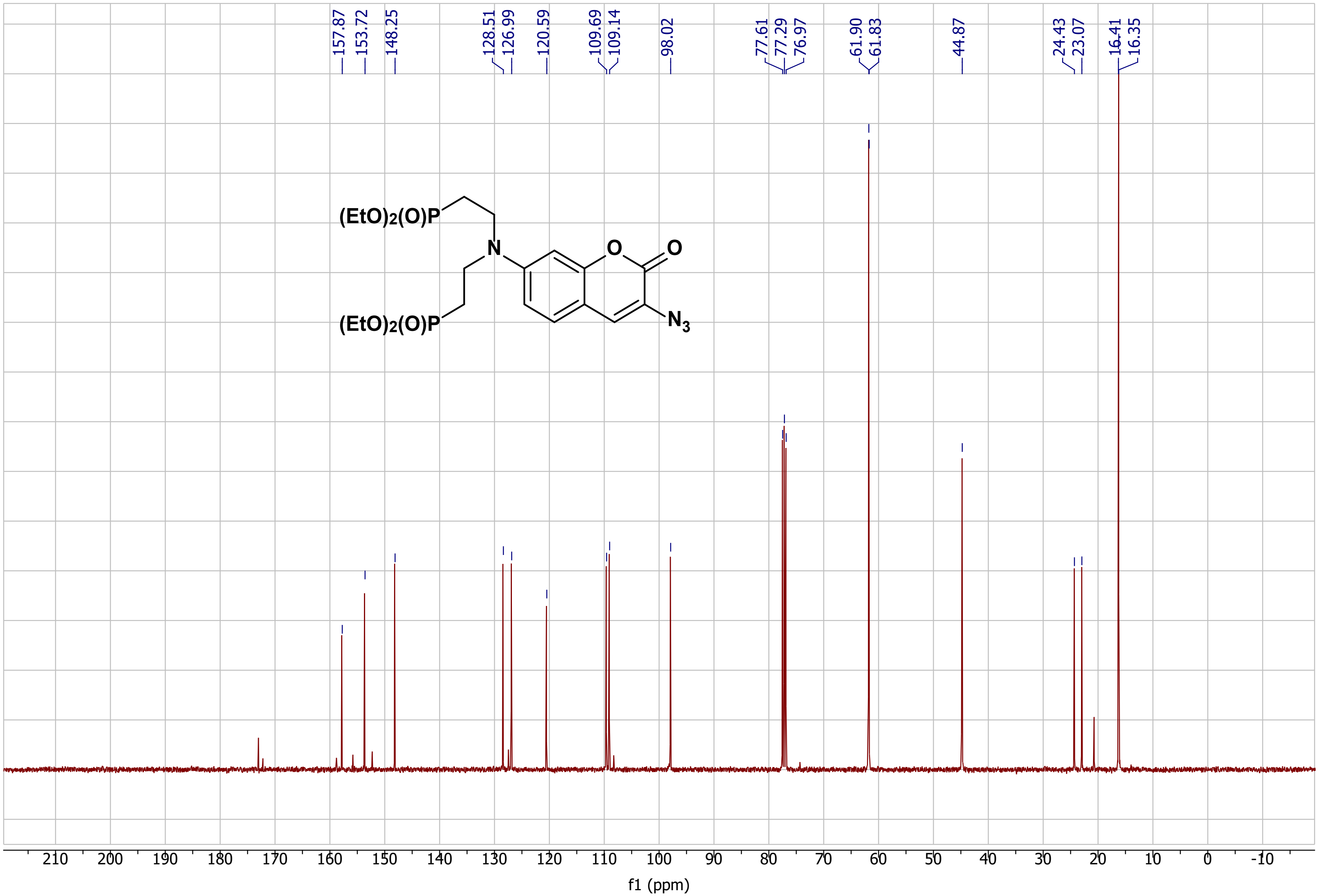

^{13}C NMR spectrum of **20** ($CDCl_3$)

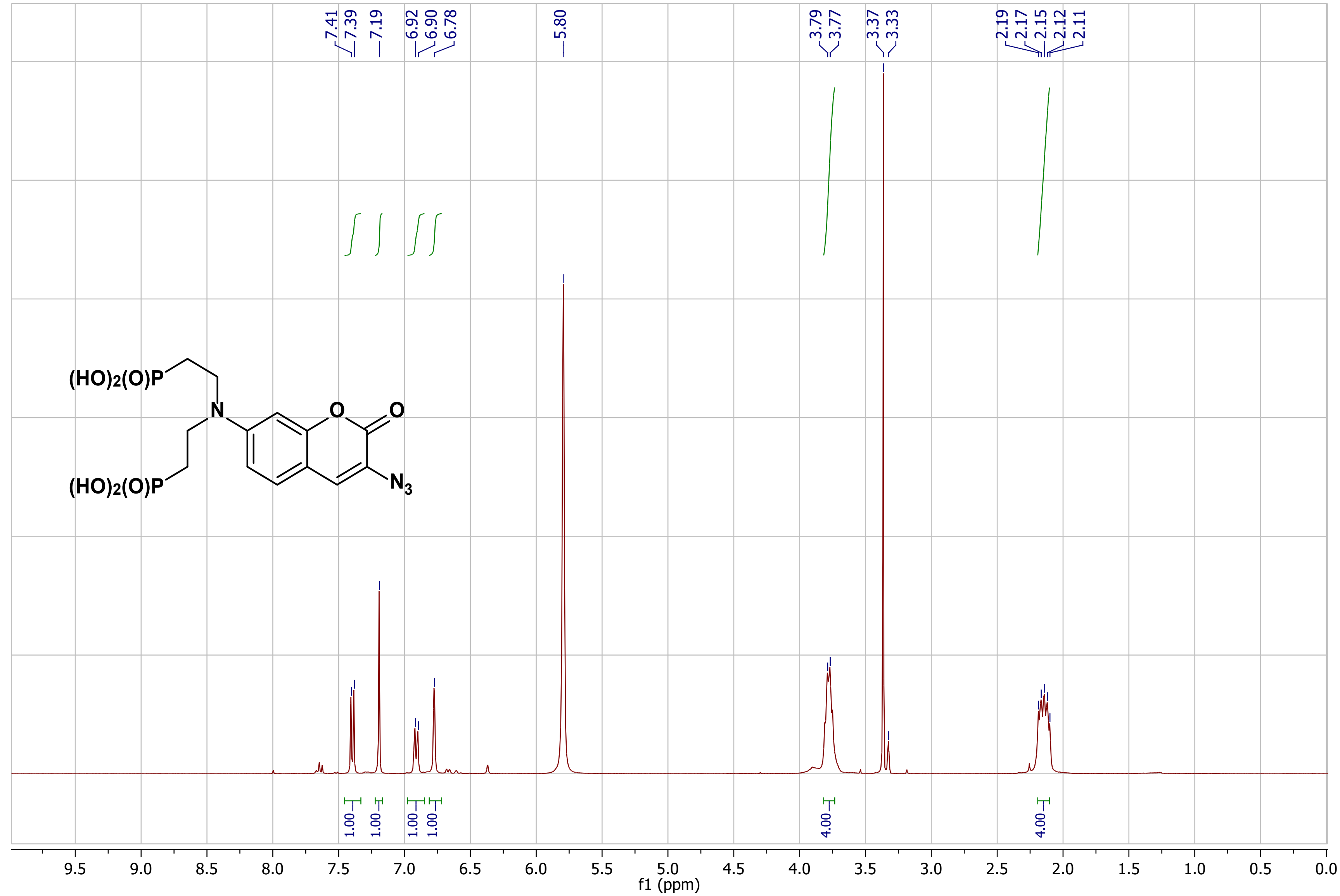

^{1}H NMR spectrum of **38** (CD_3OD)

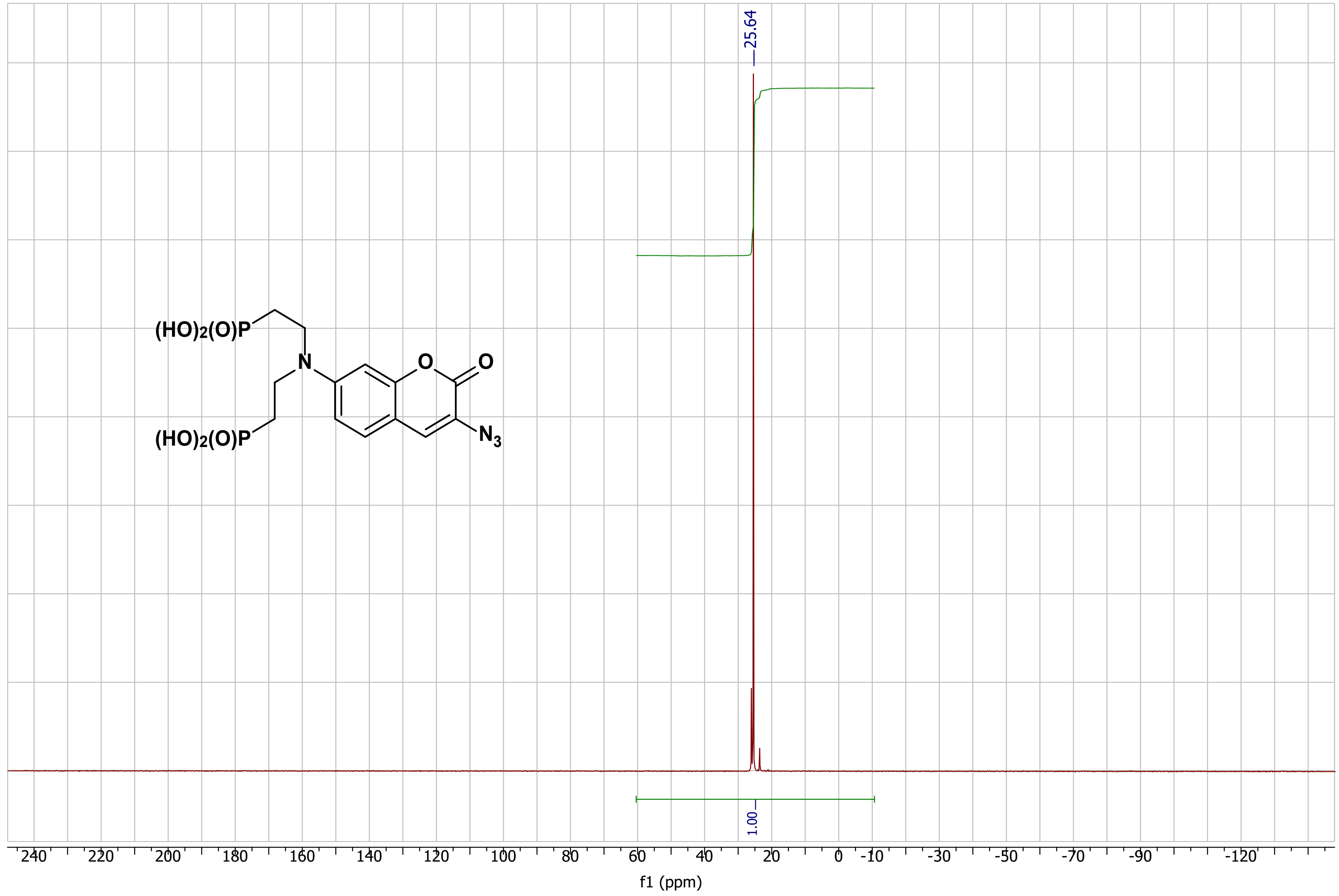

^{31}P NMR spectrum of **38** (CD_3OD)

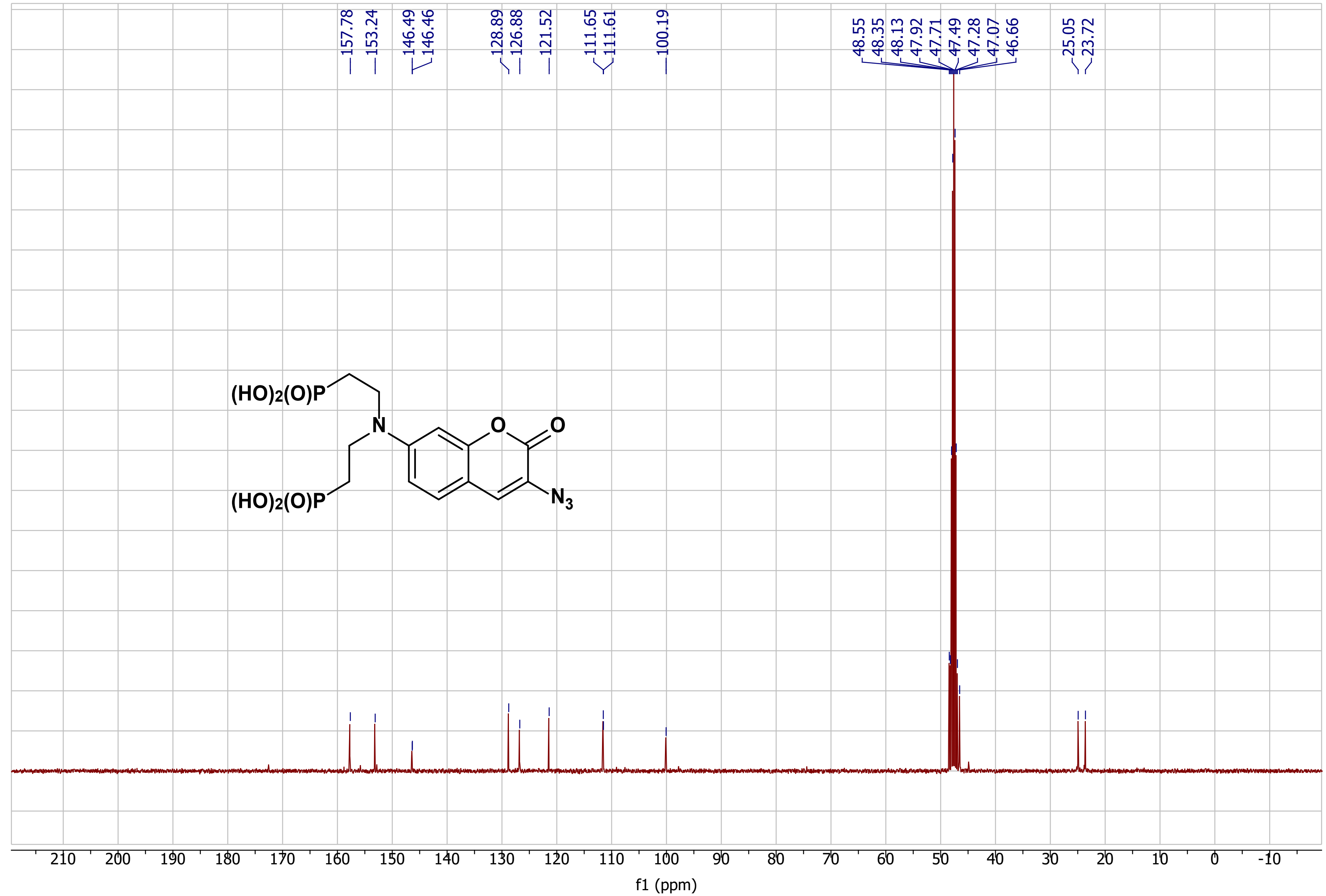

^{13}C NMR spectrum of **38** (CD_3OD)

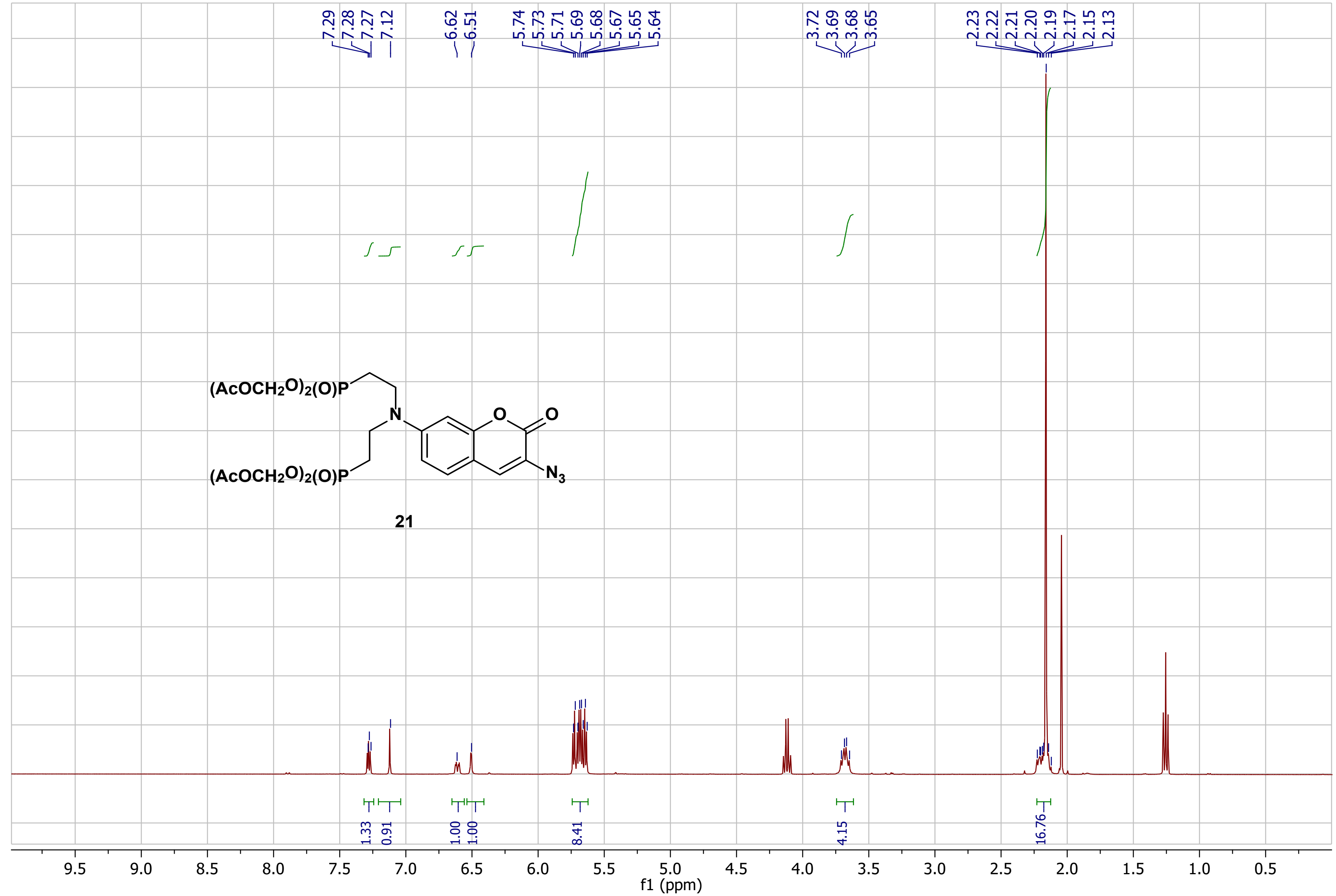

^{1}H NMR spectrum of **21** ($CDCl_3$)

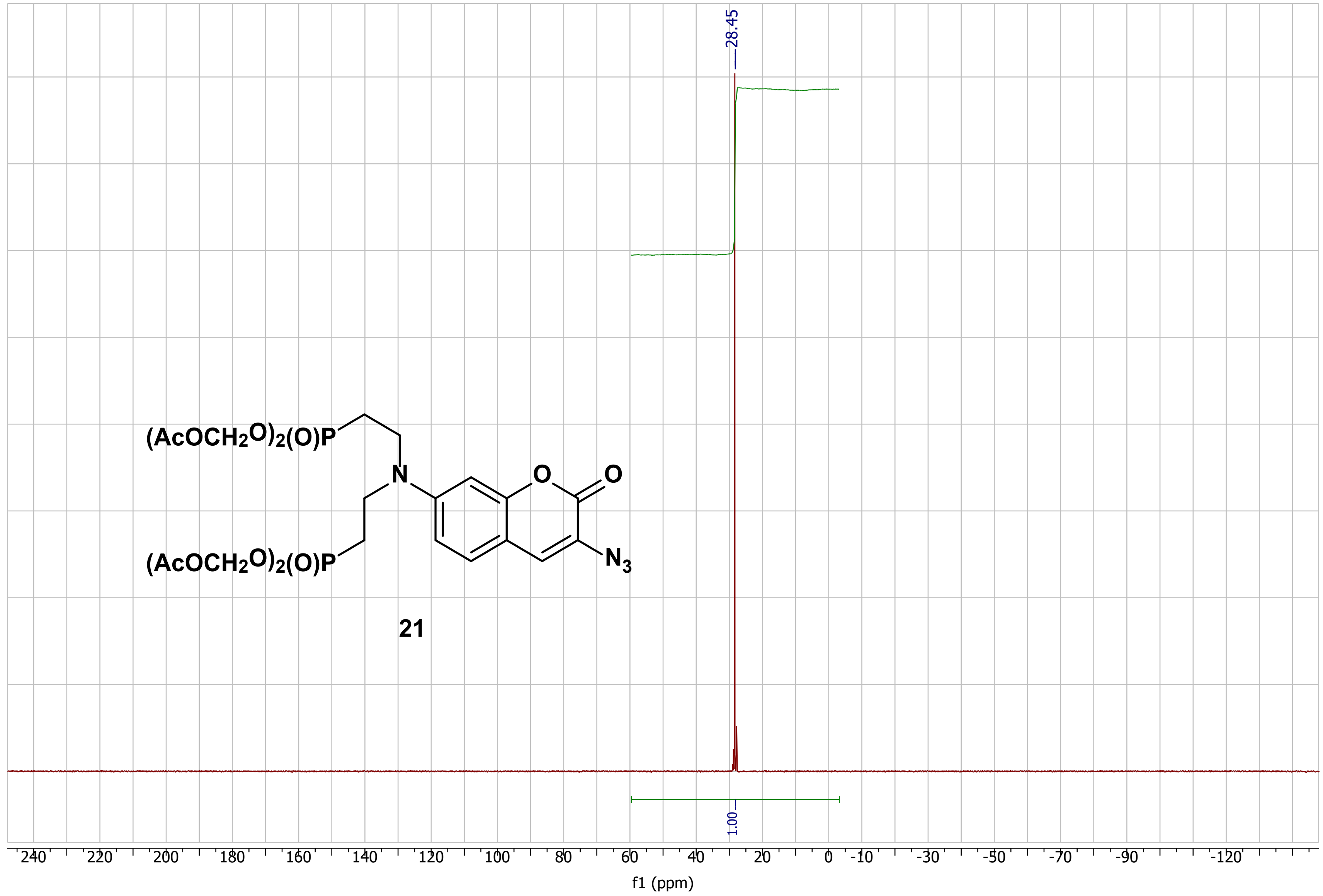

^{31}P NMR spectrum of **21** ($CDCl_3$)

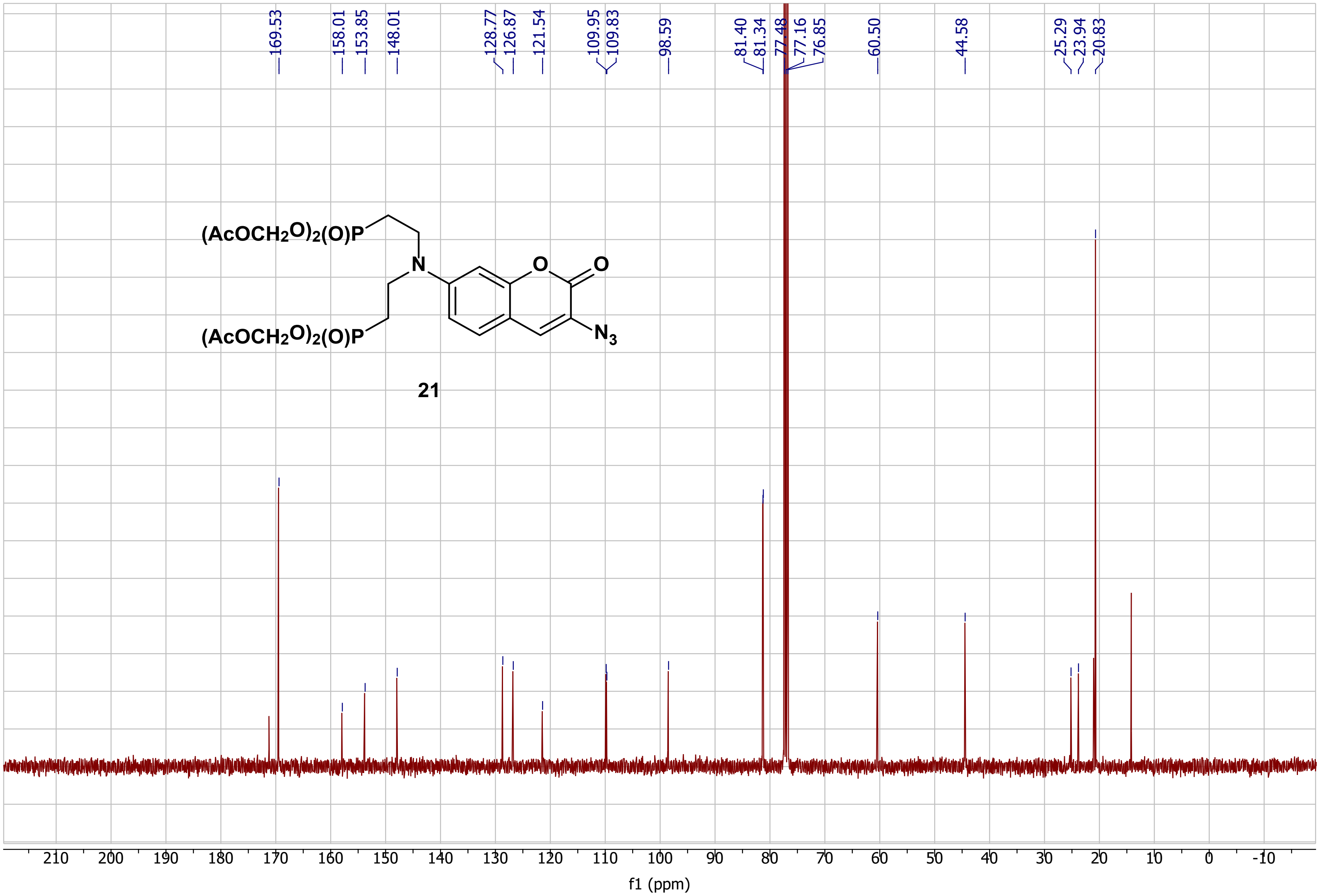

^{13}C NMR spectrum of **21** ($CDCl_3$)

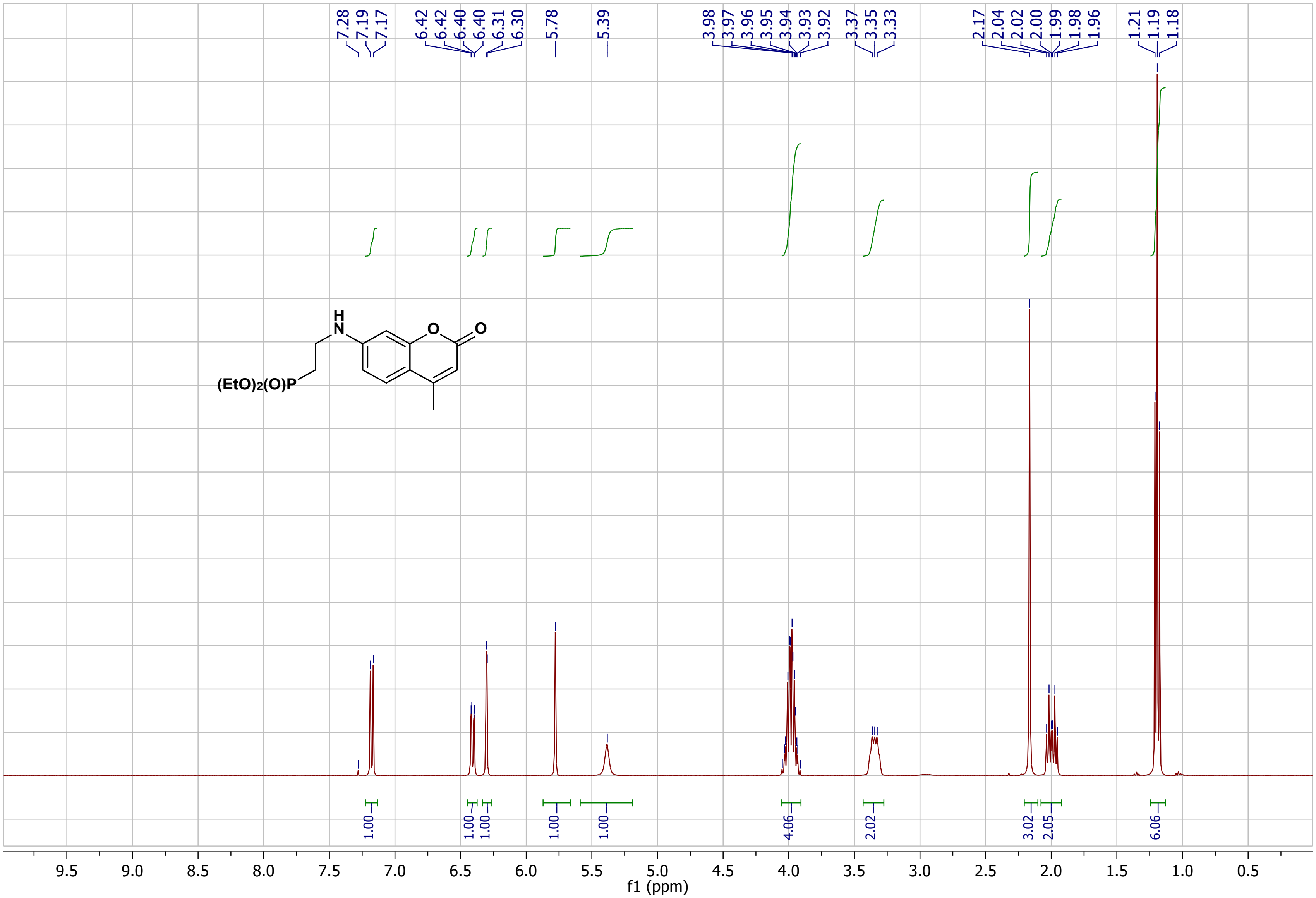

^{1}H NMR spectrum of **39** ($CDCl_3$)

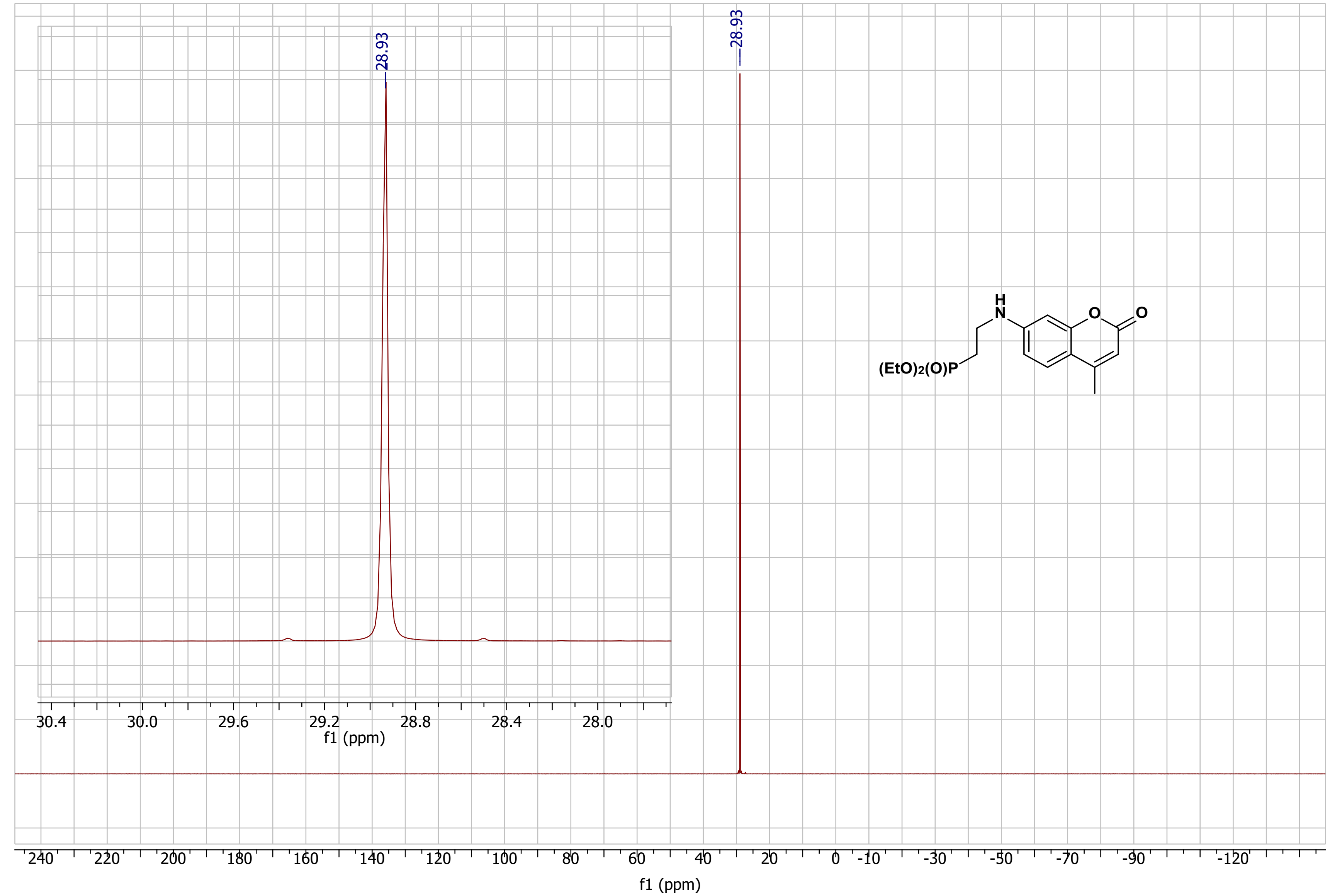

^{31}P NMR spectrum of **39** ($CDCl_3$)

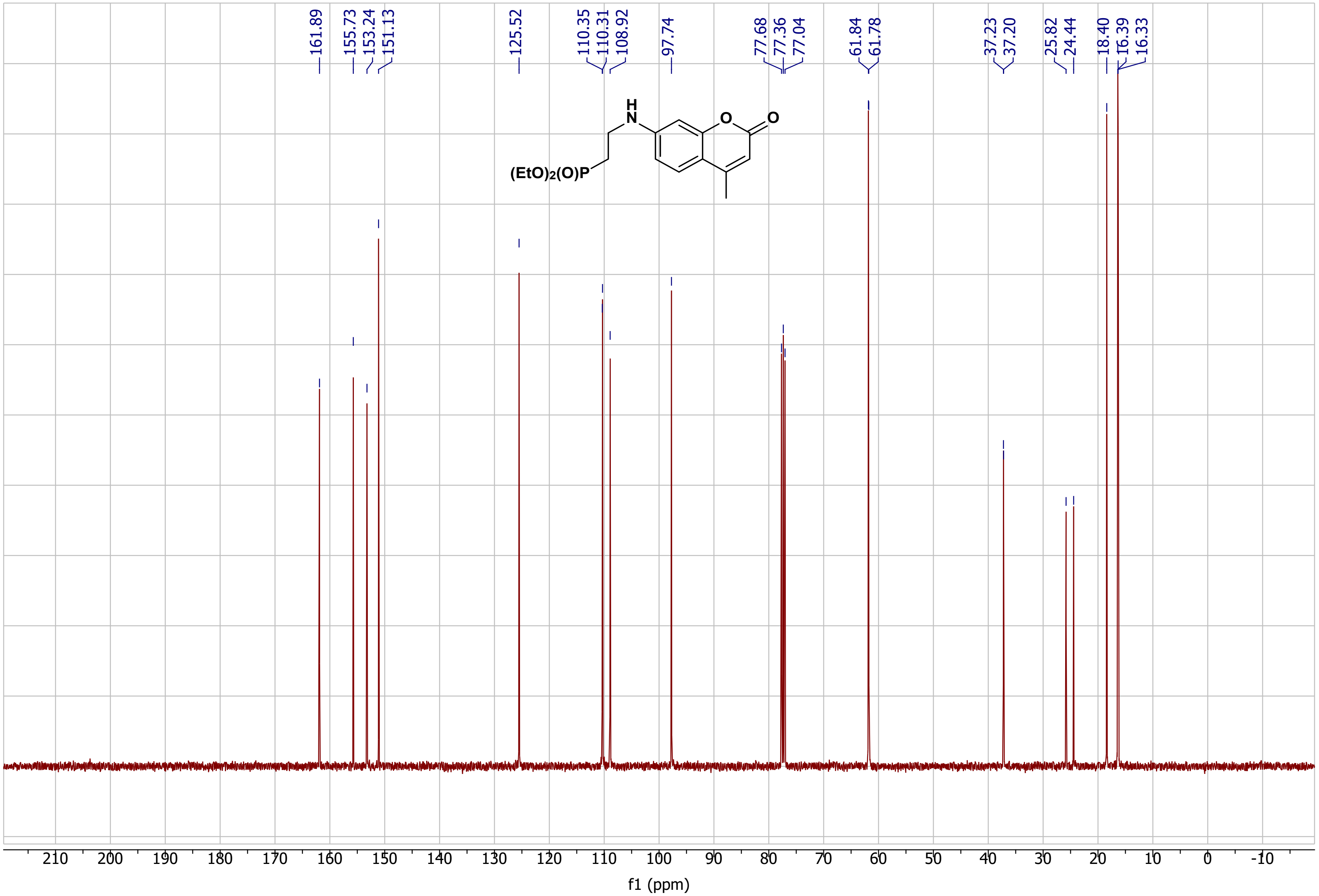

^{13}C NMR spectrum of **39** ($CDCl_3$)

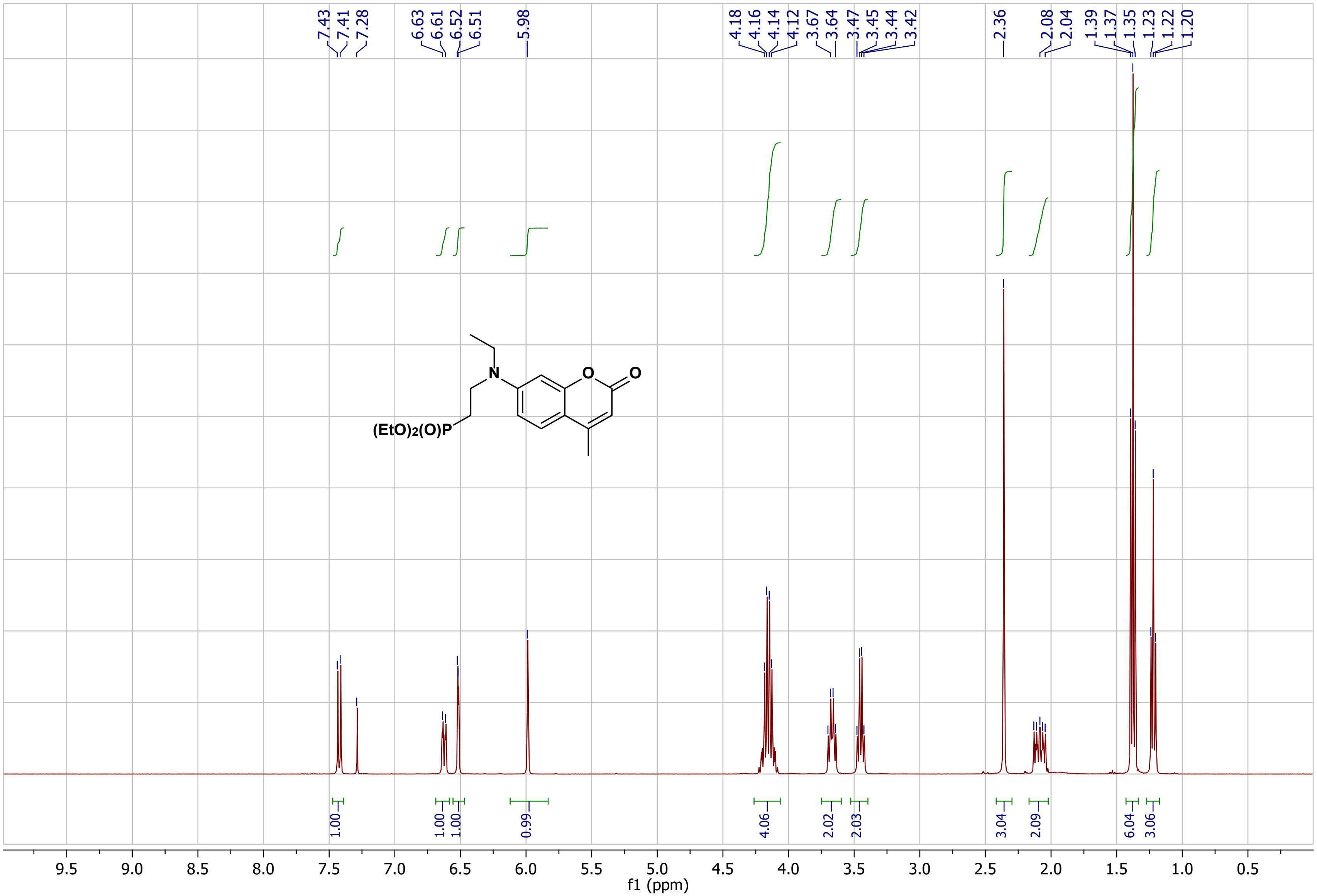

^{1}H NMR spectrum of **40** ($CDCl_3$)

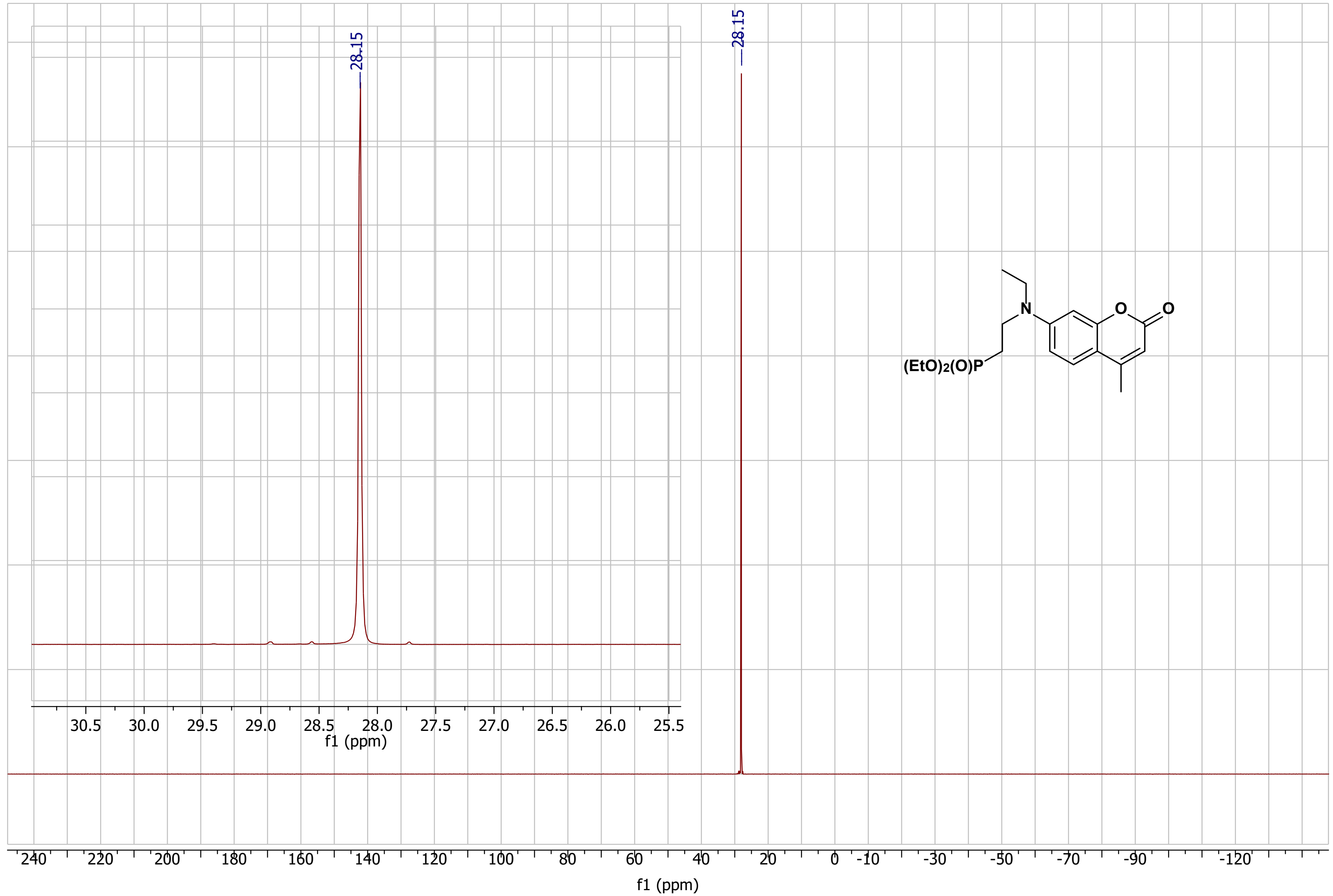

^{31}P NMR spectrum of **40** ($CDCl_3$)

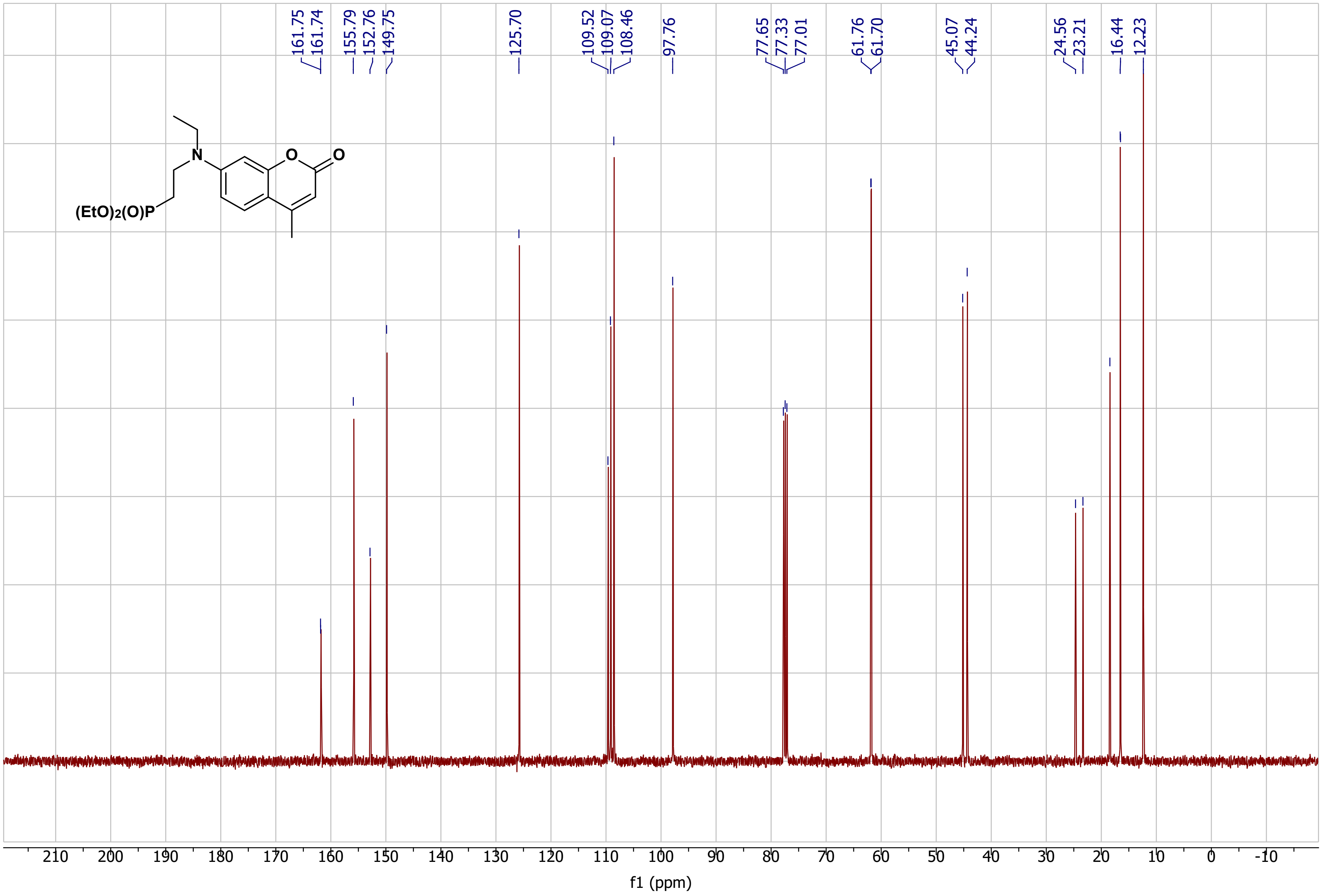

^{13}C NMR spectrum of **40** ($CDCl_3$)

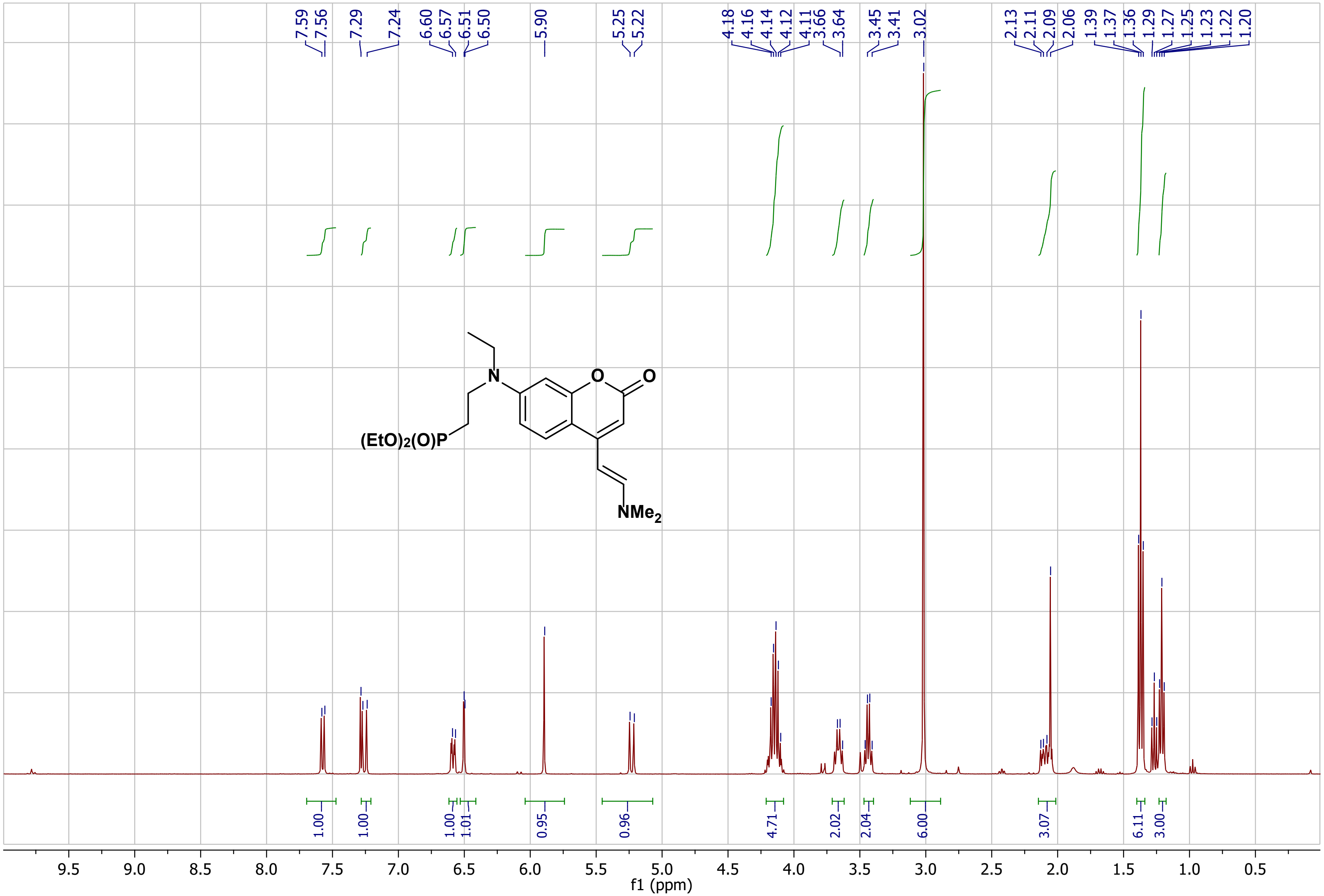

^{1}H NMR spectrum of **41** ($CDCl_3$)

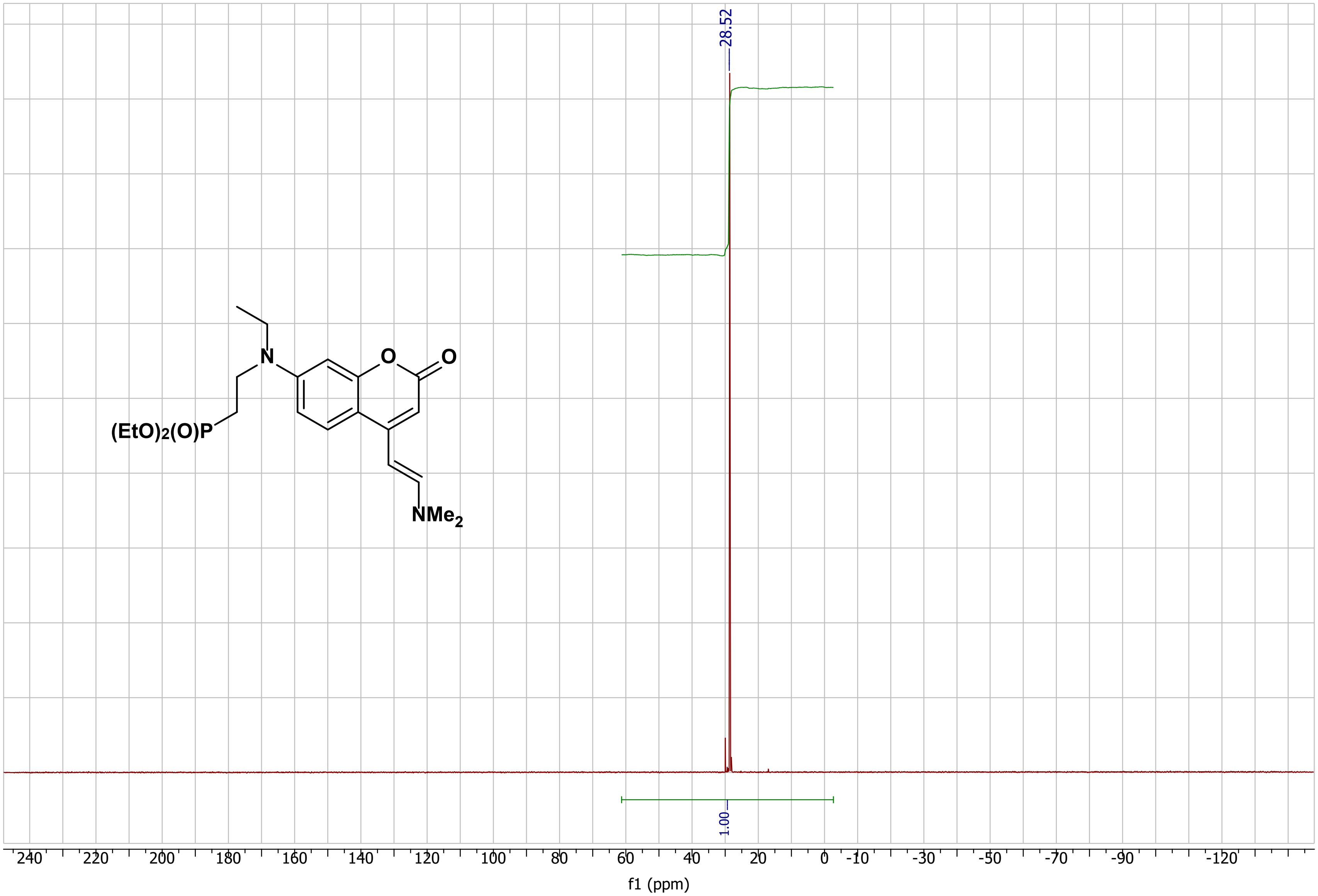

^{31}P NMR spectrum of **41** ($CDCl_3$)

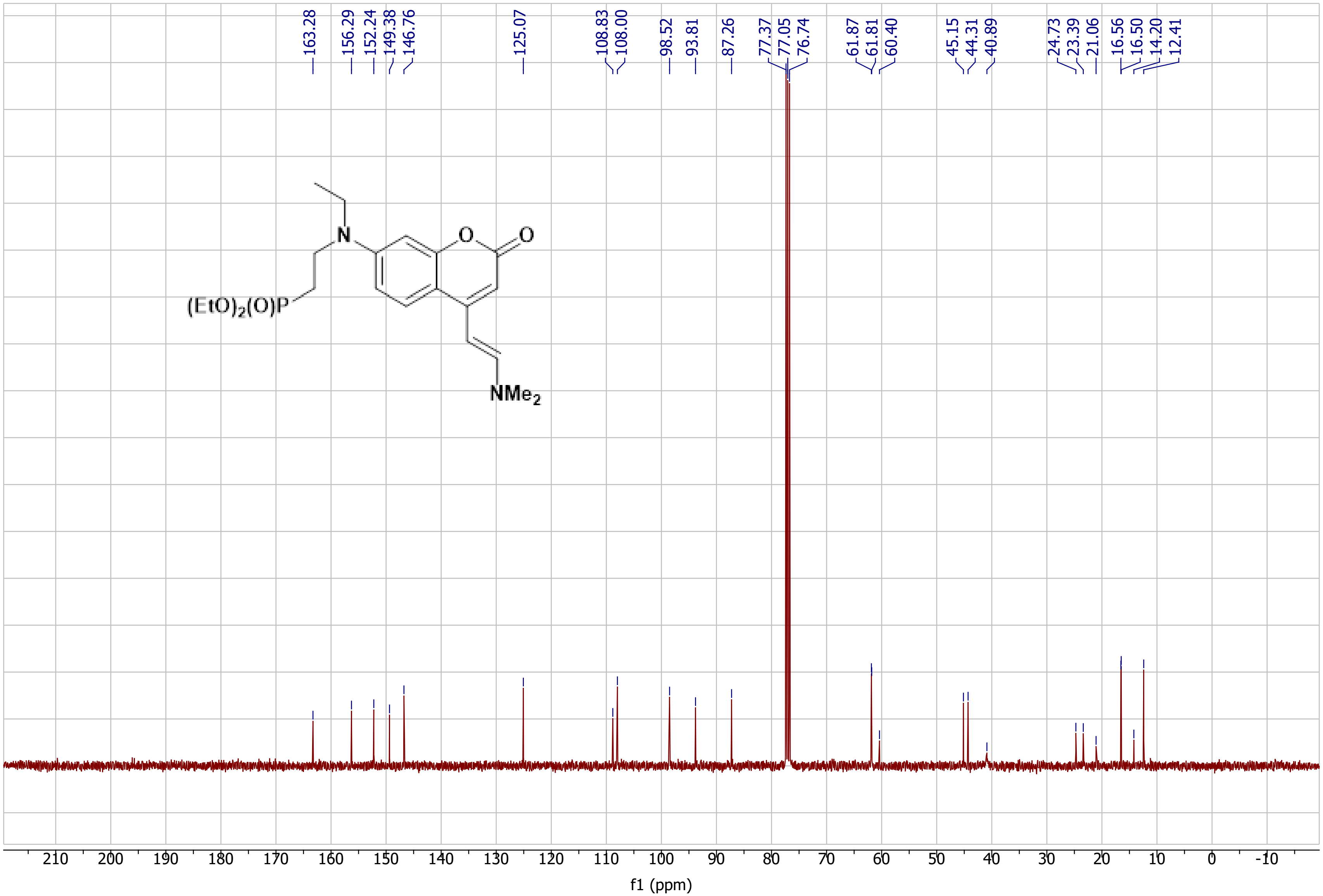

^{13}C NMR spectrum of **41** ($CDCl_3$)

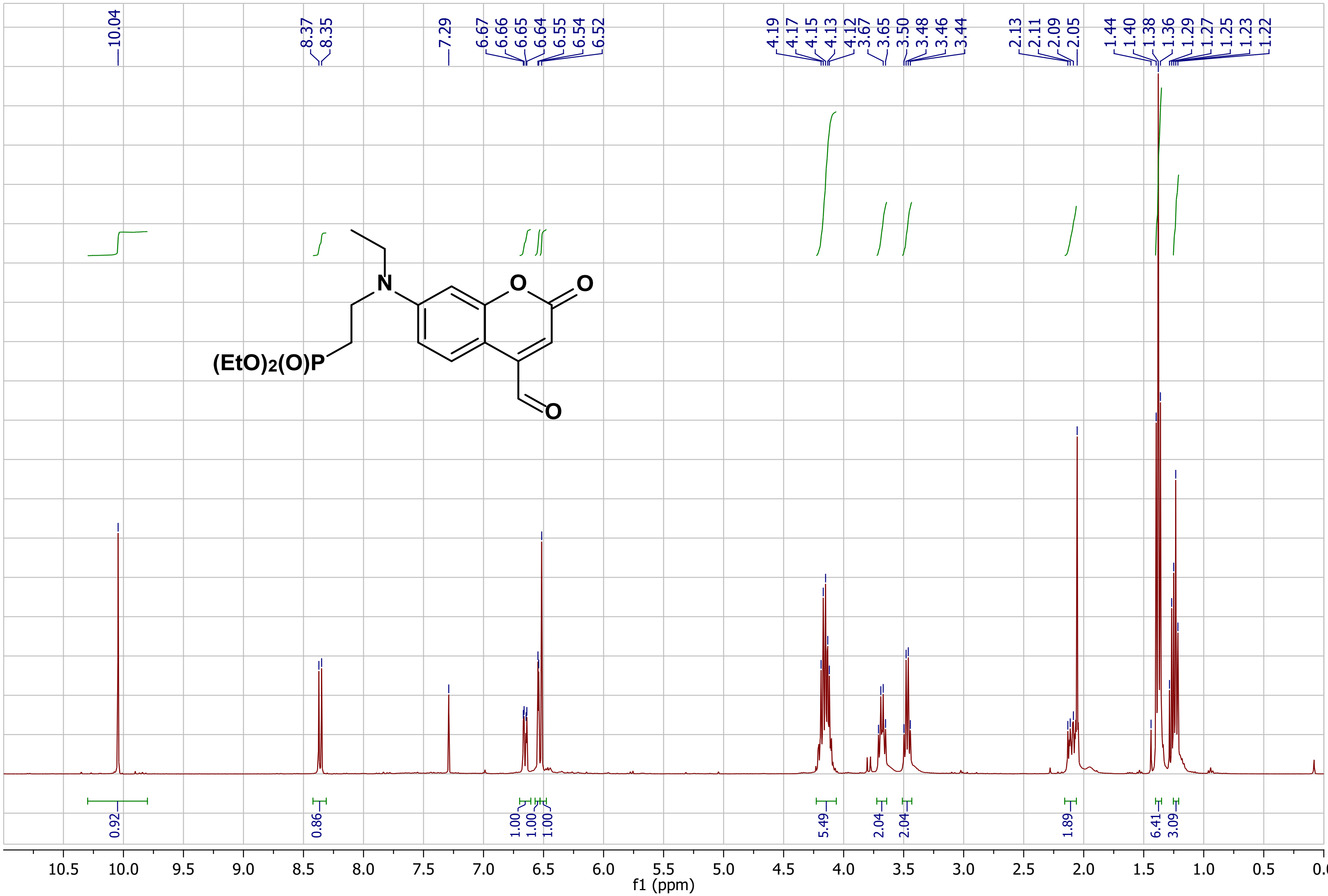

^{1}H NMR spectrum of **42** ($CDCl_3$)

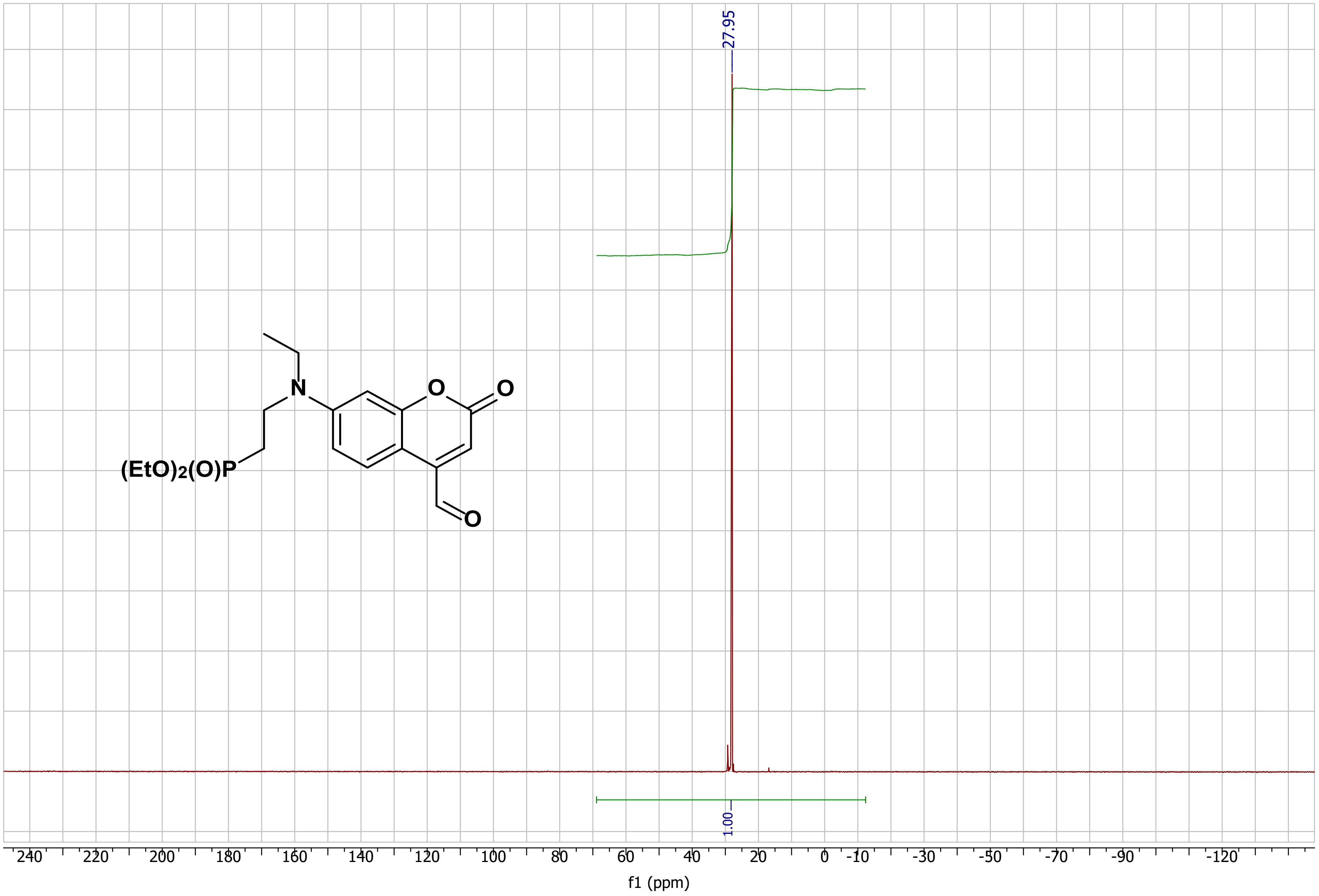

^{31}P NMR spectrum of **42** ($CDCl_3$)

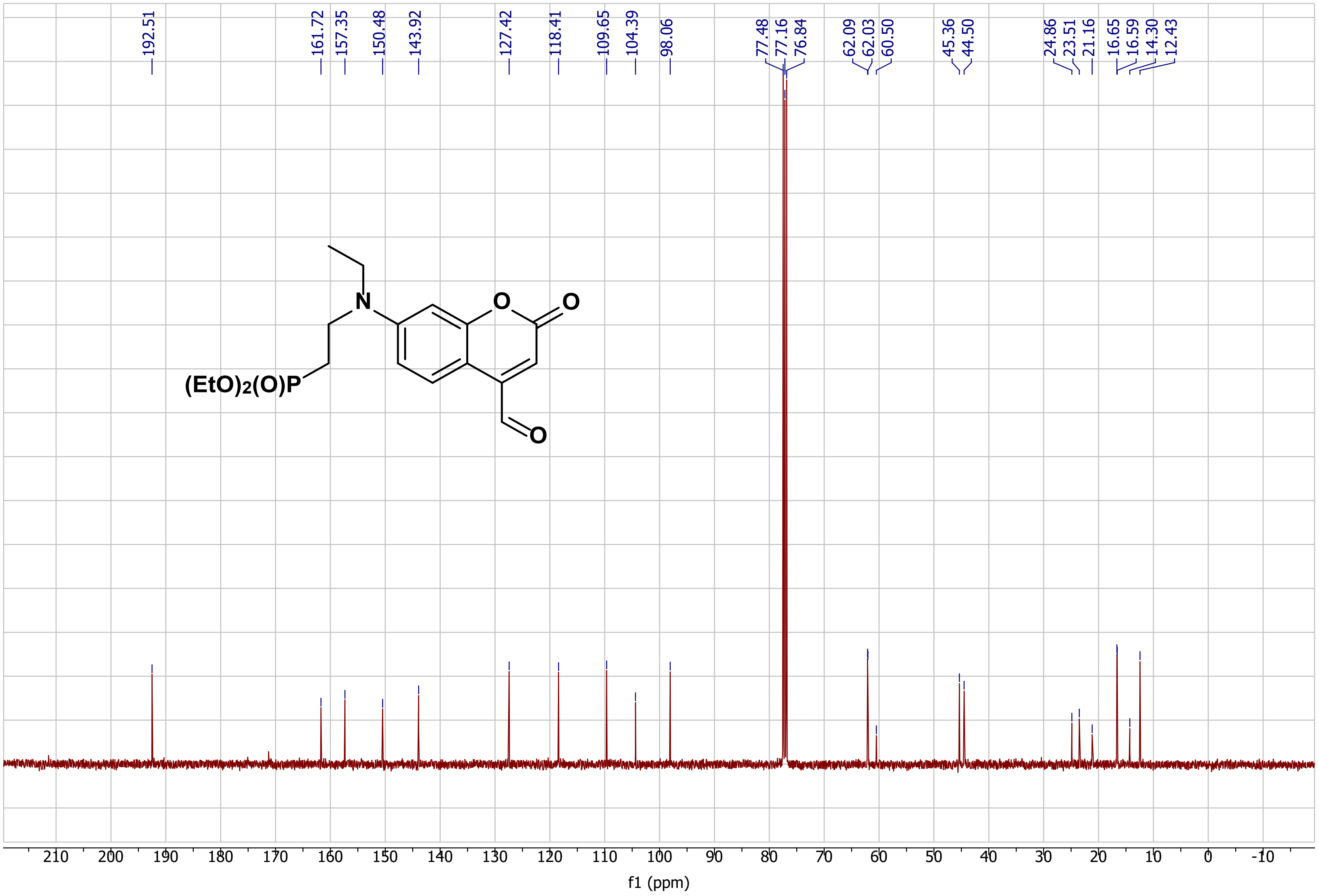

^{13}C NMR spectrum of **42** ($CDCl_3$)

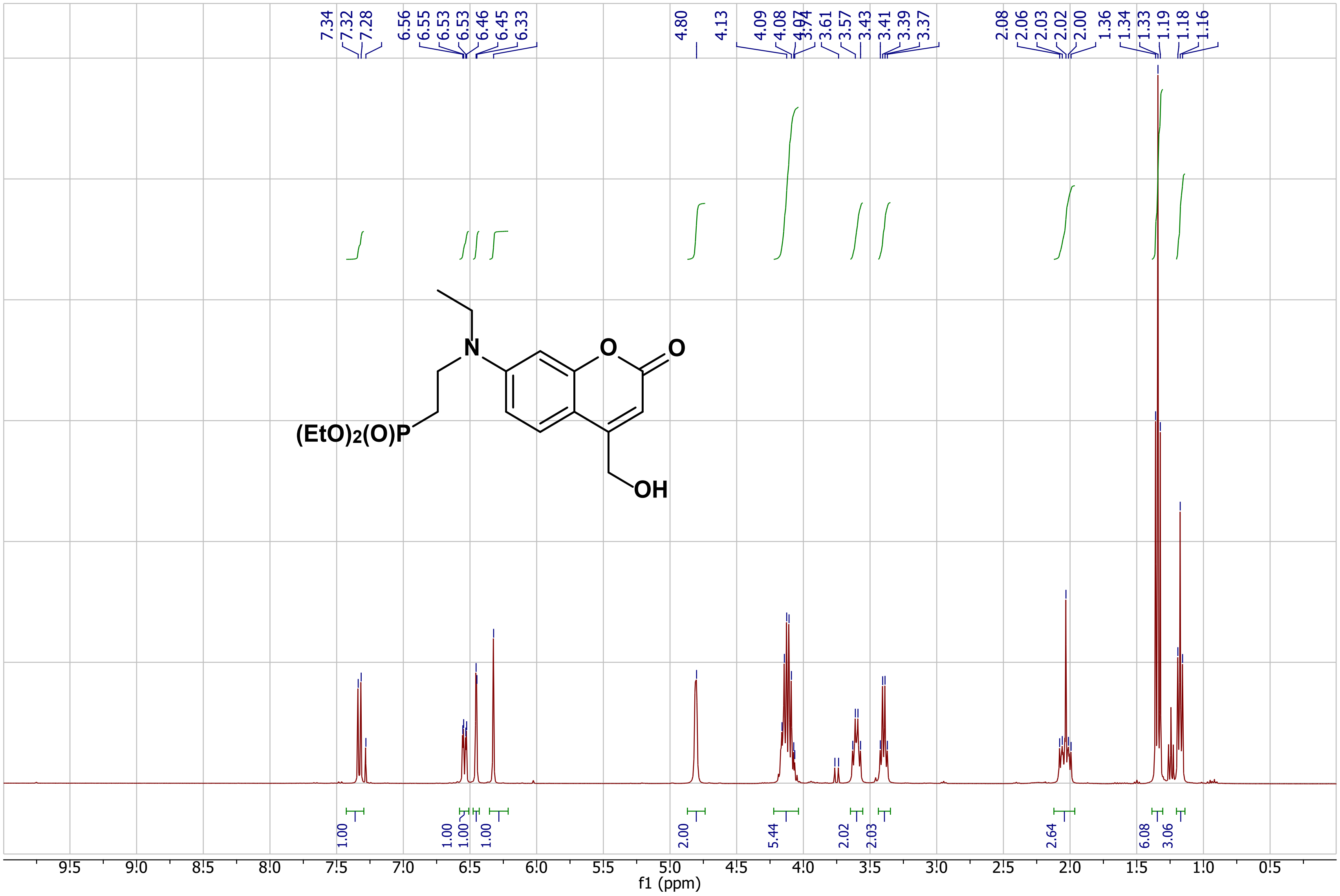

^{1}H NMR spectrum of **43** ($CDCl_3$)

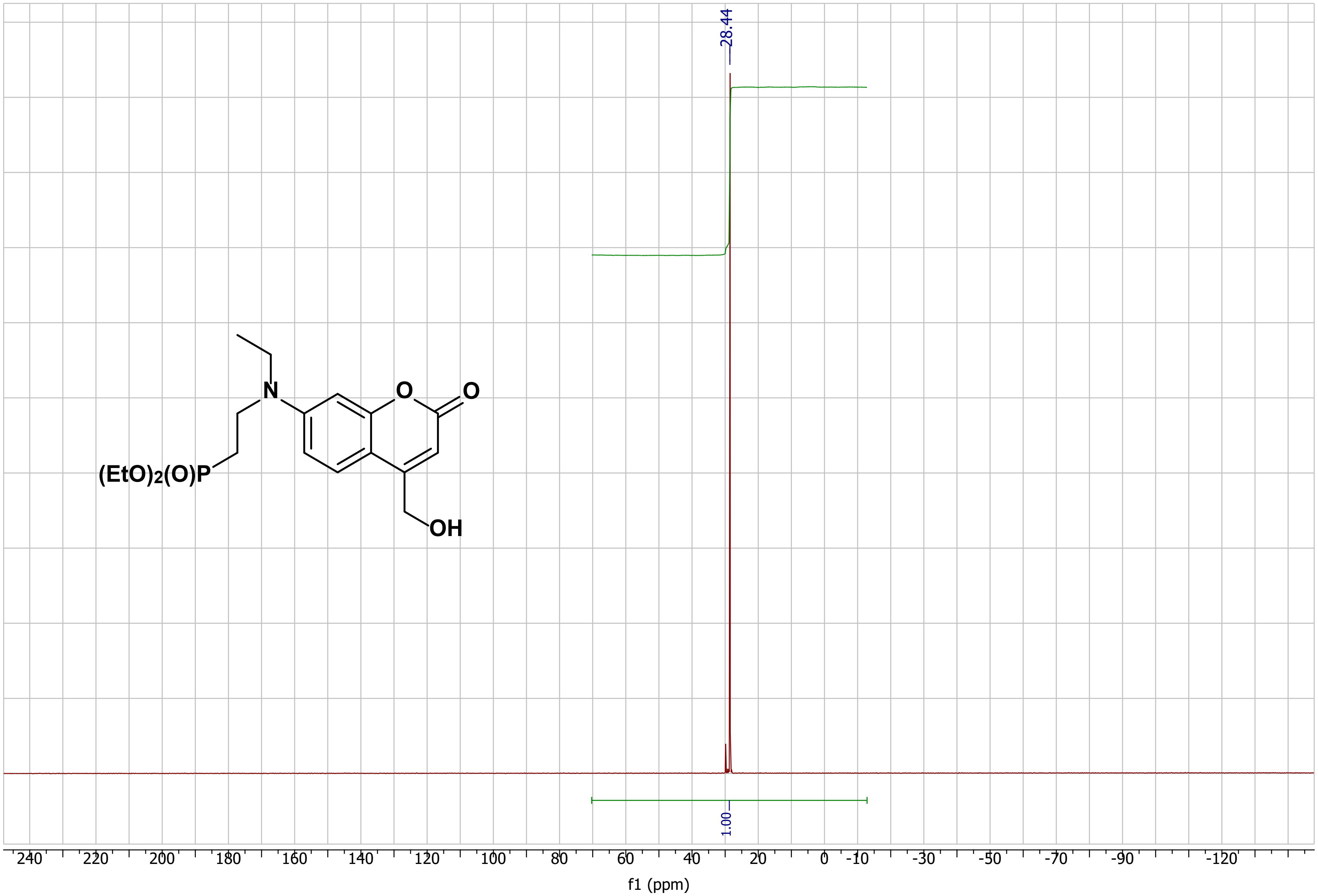

^{31}P NMR spectrum of **43** ($CDCl_3$)

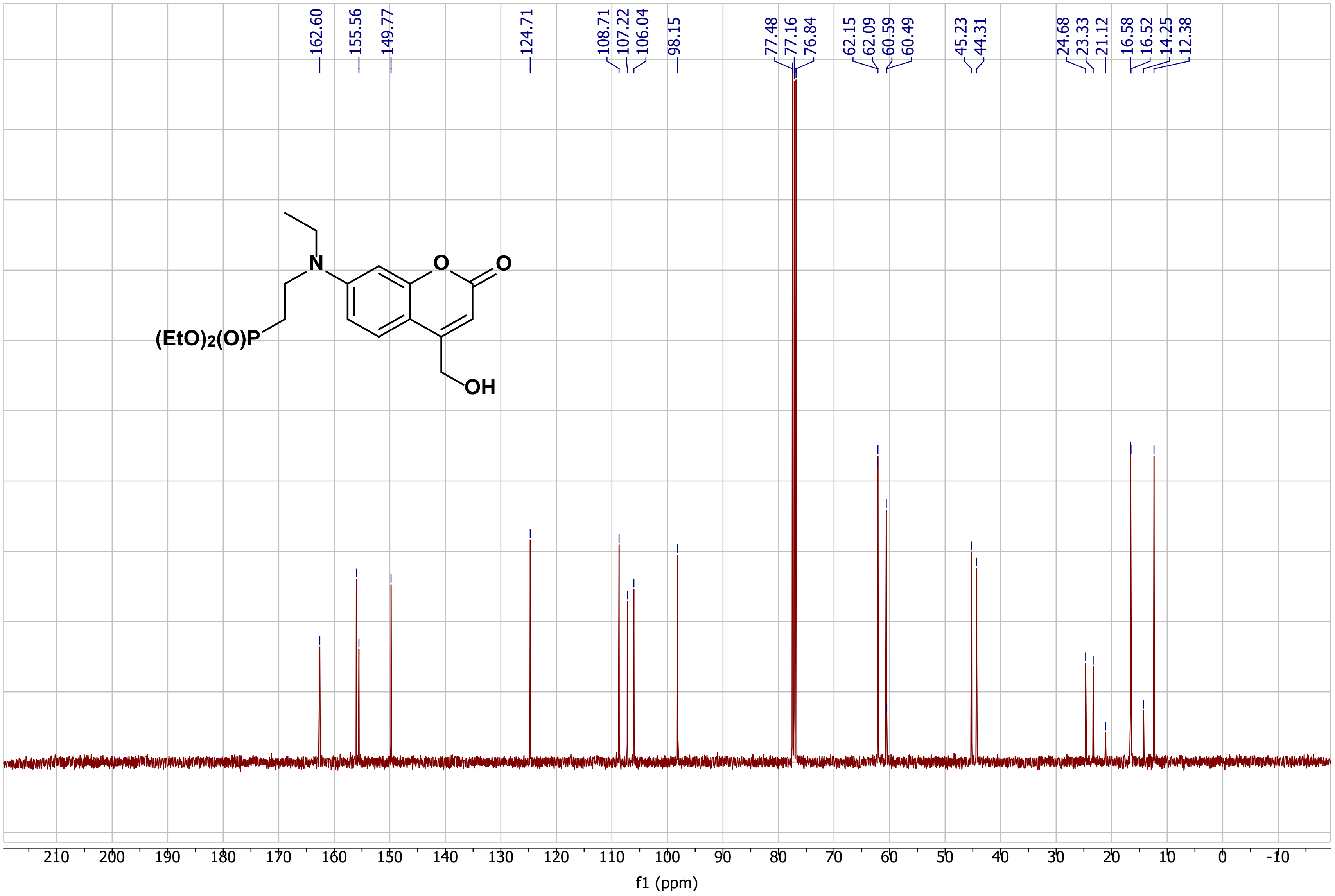

^{13}C NMR spectrum of **43** ($CDCl_3$)

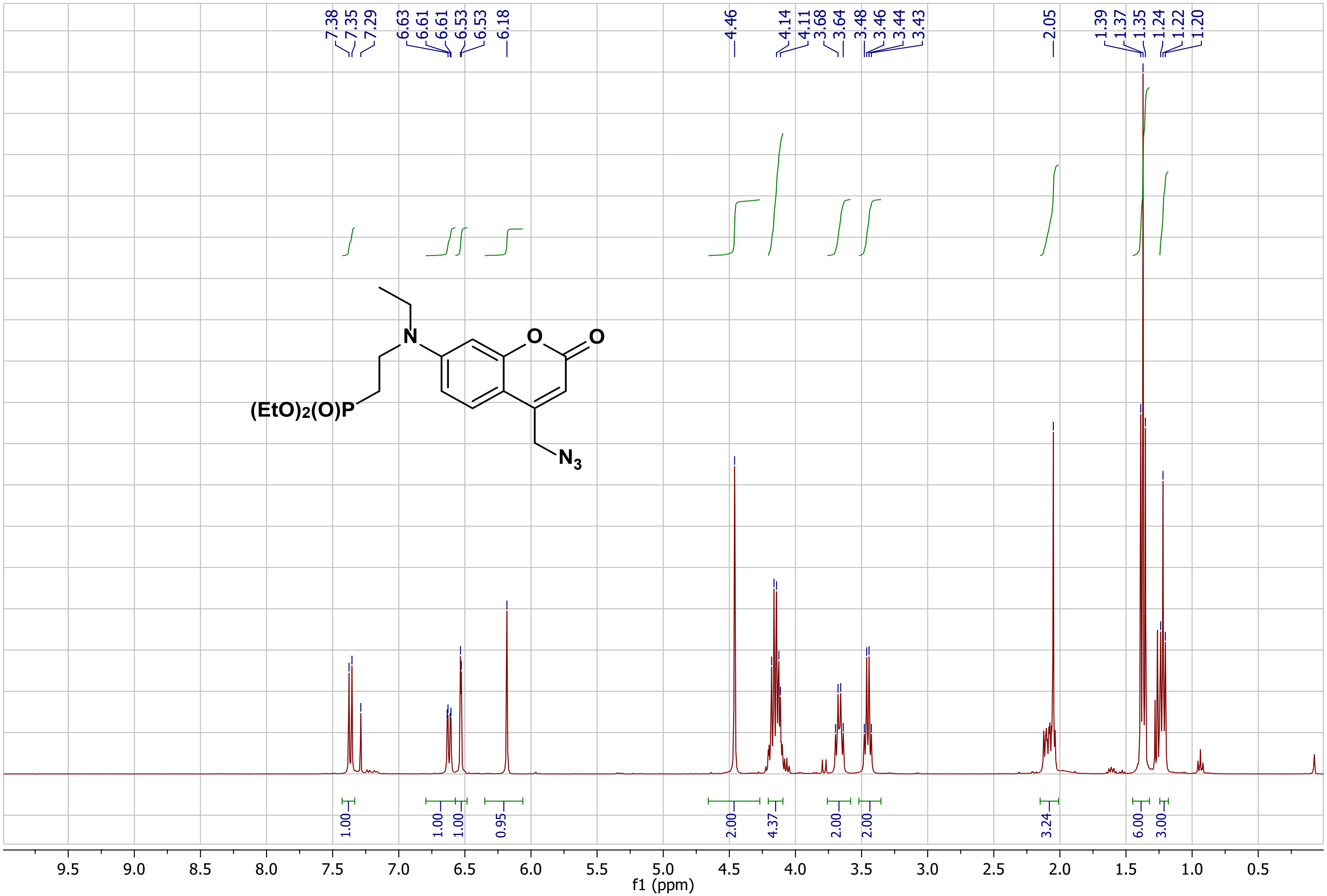

^{1}H NMR spectrum of **44** ($CDCl_3$)

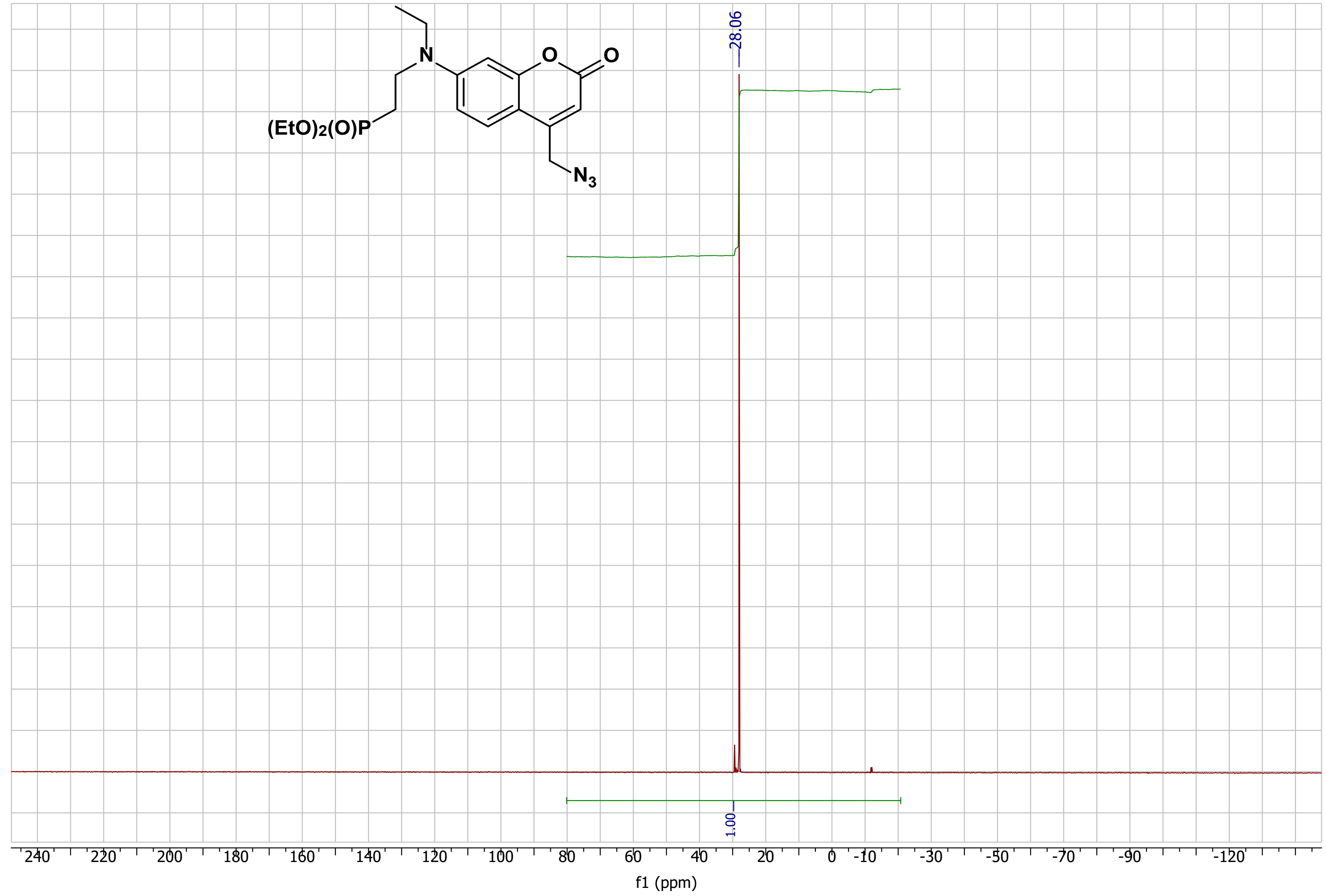

^{31}P NMR spectrum of **44** ($CDCl_3$)

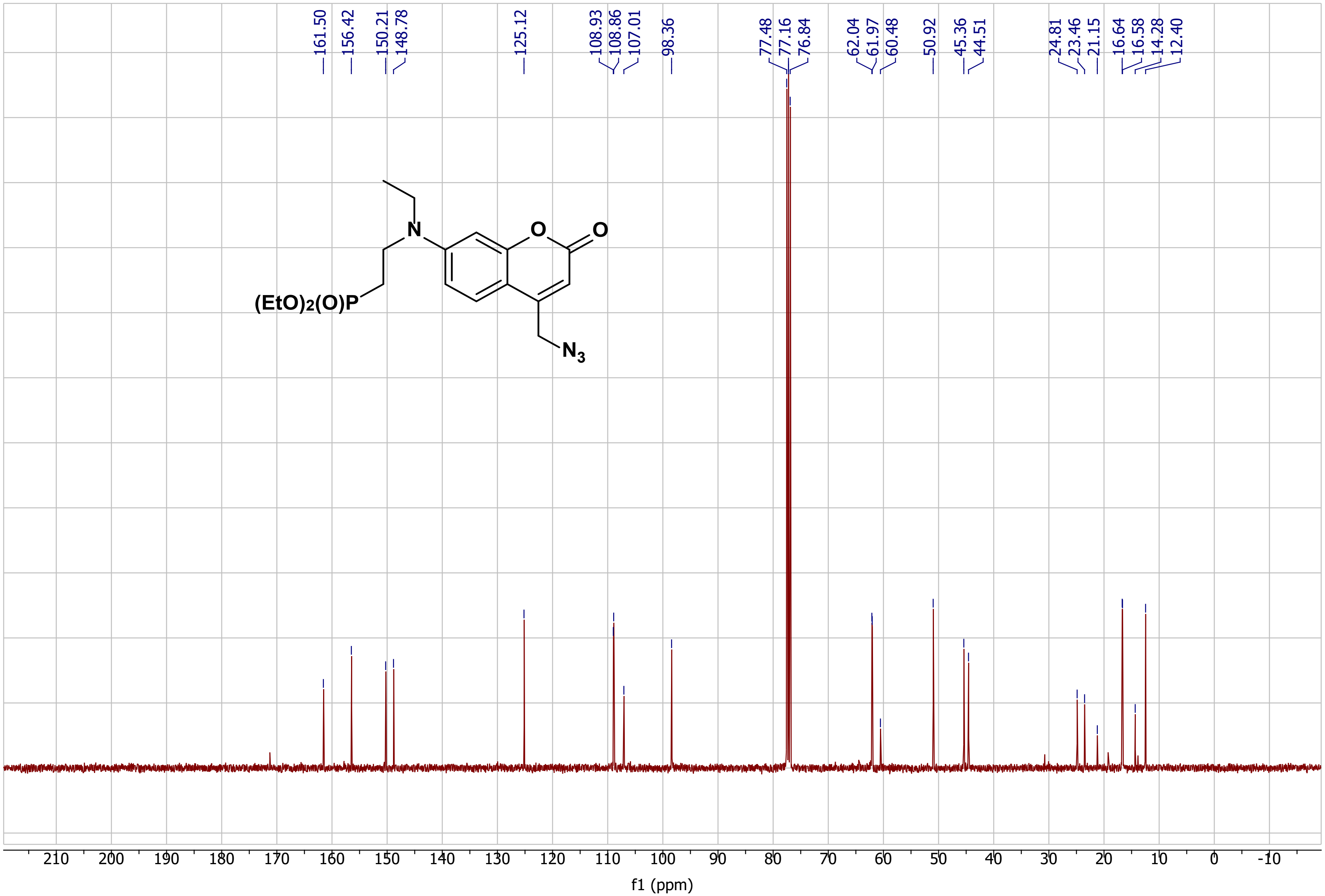

^{13}C NMR spectrum of **44** ($CDCl_3$)

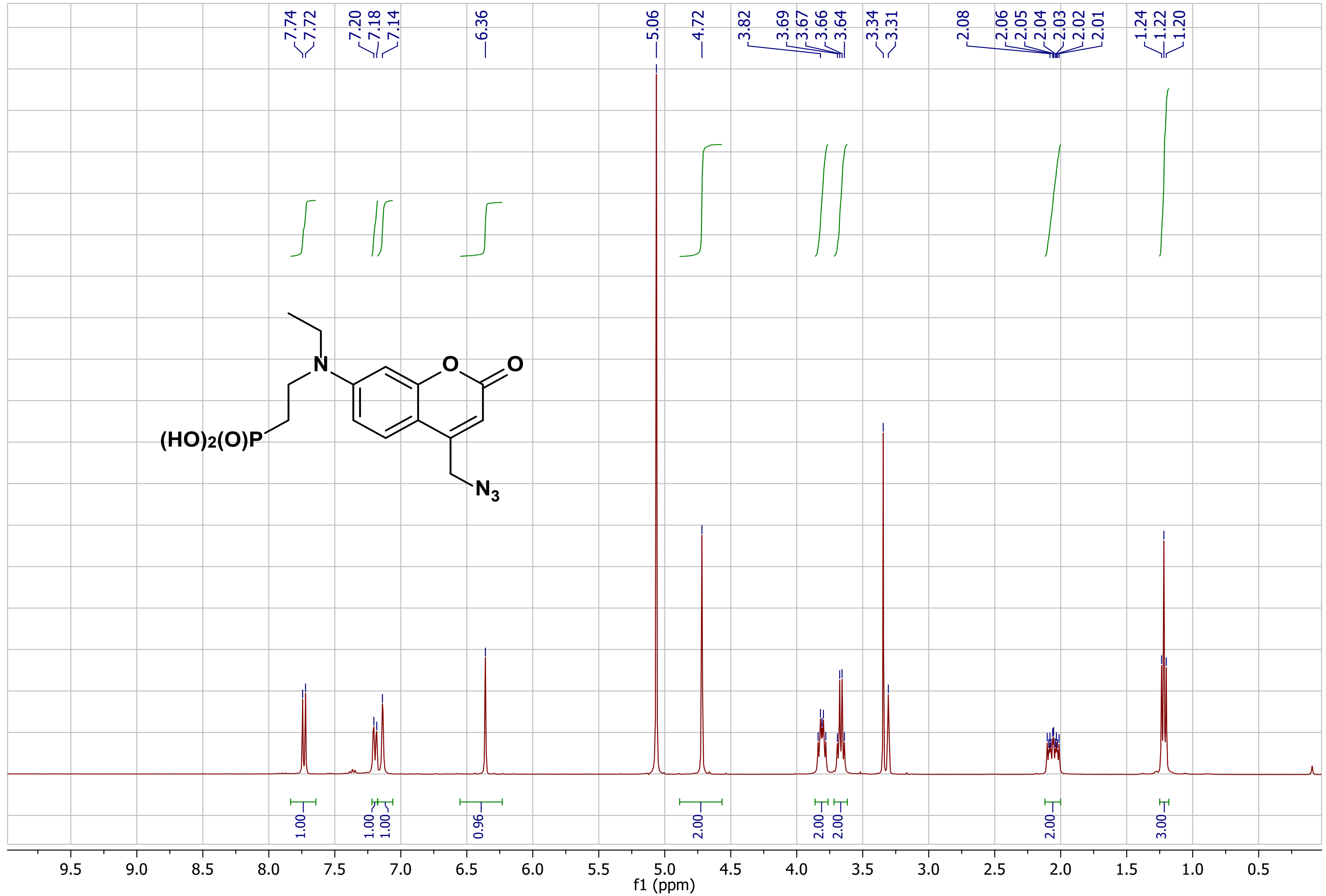

^1H NMR spectrum of **45** (CD_3OD)

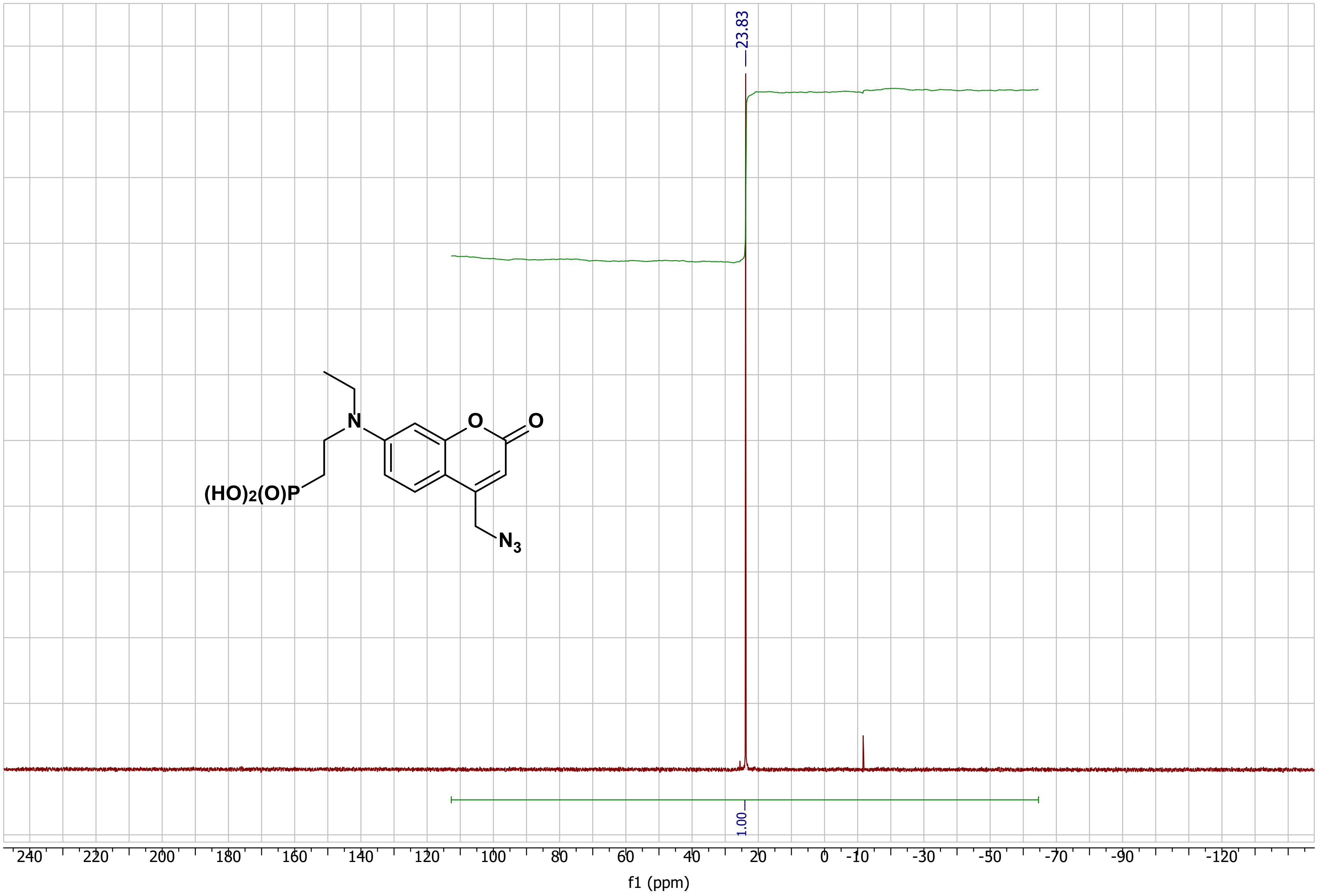

^{31}P NMR spectrum of **45** (CD_3OD)

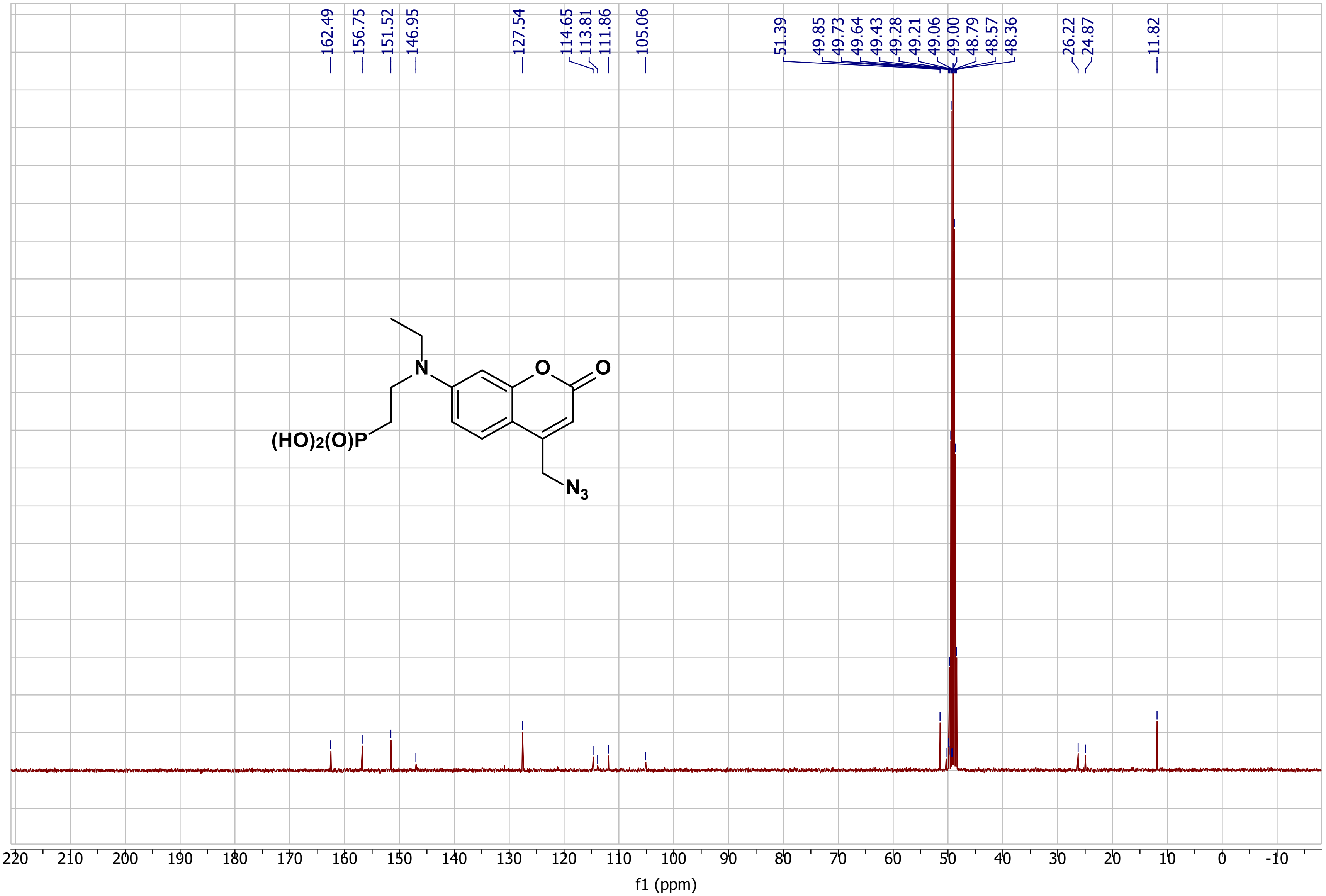

^{13}C NMR spectrum of **45** (CD_3OD)

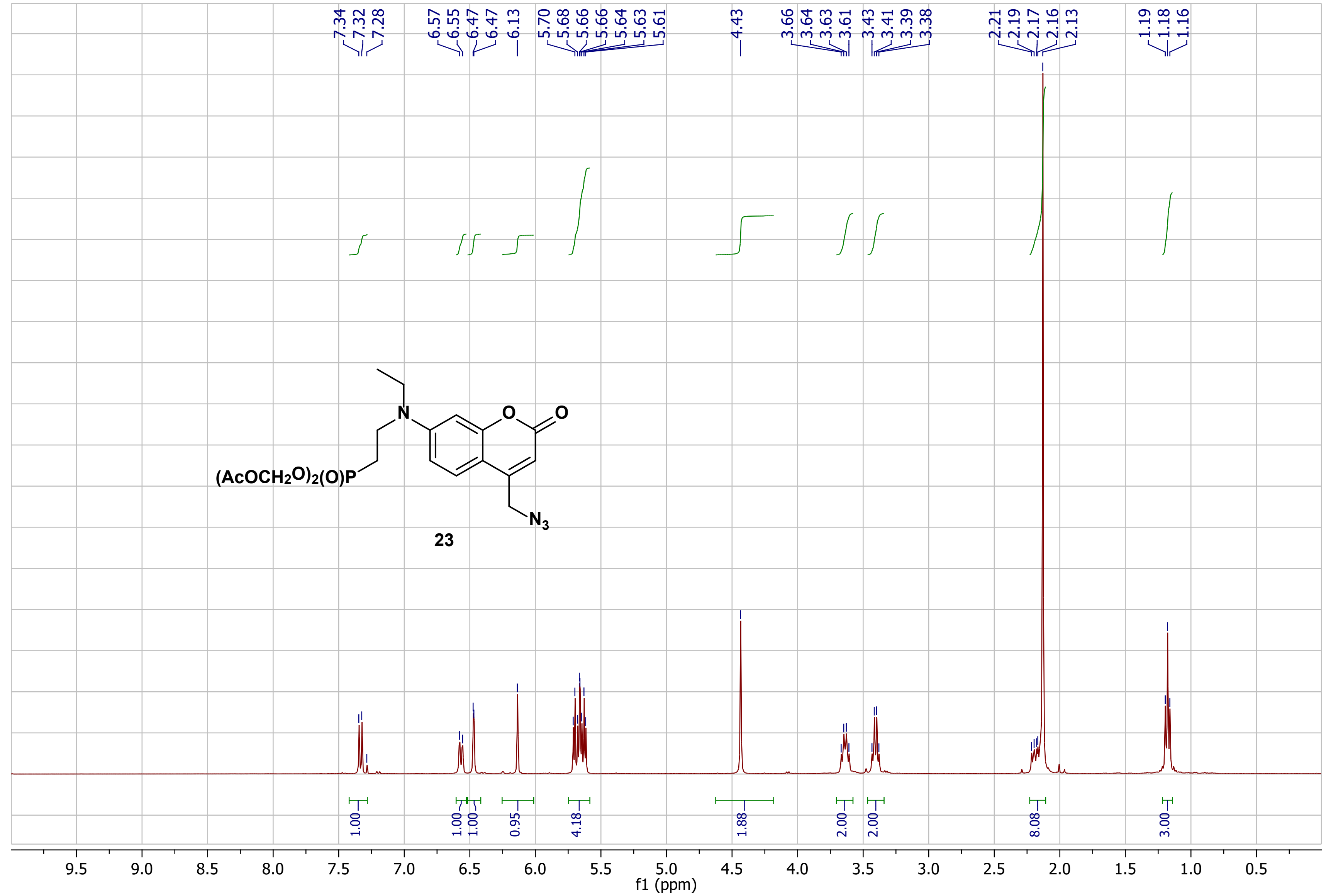

^{1}H NMR spectrum of **23** ($CDCl_3$)

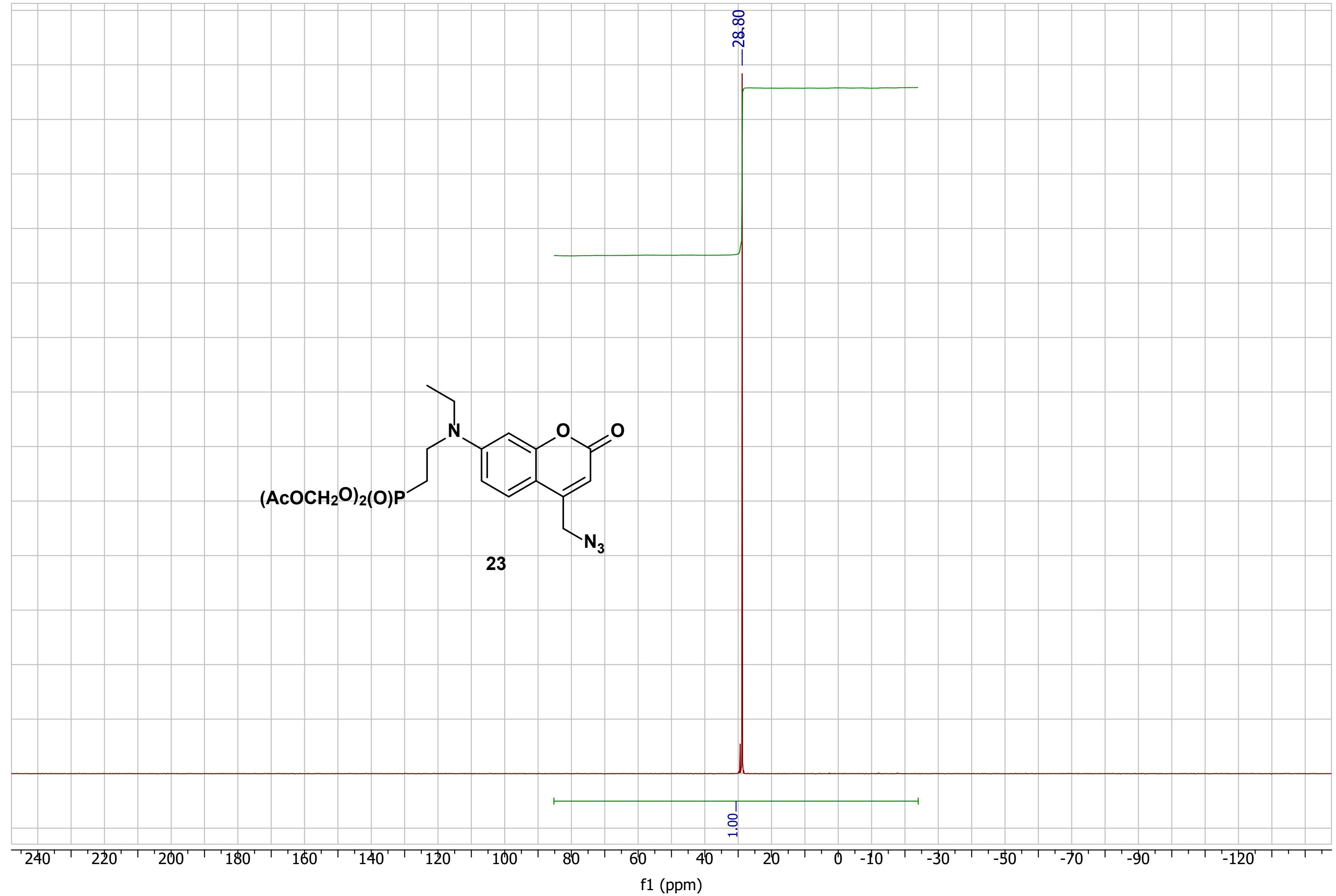

^{31}P NMR spectrum of **23** ($CDCl_3$)

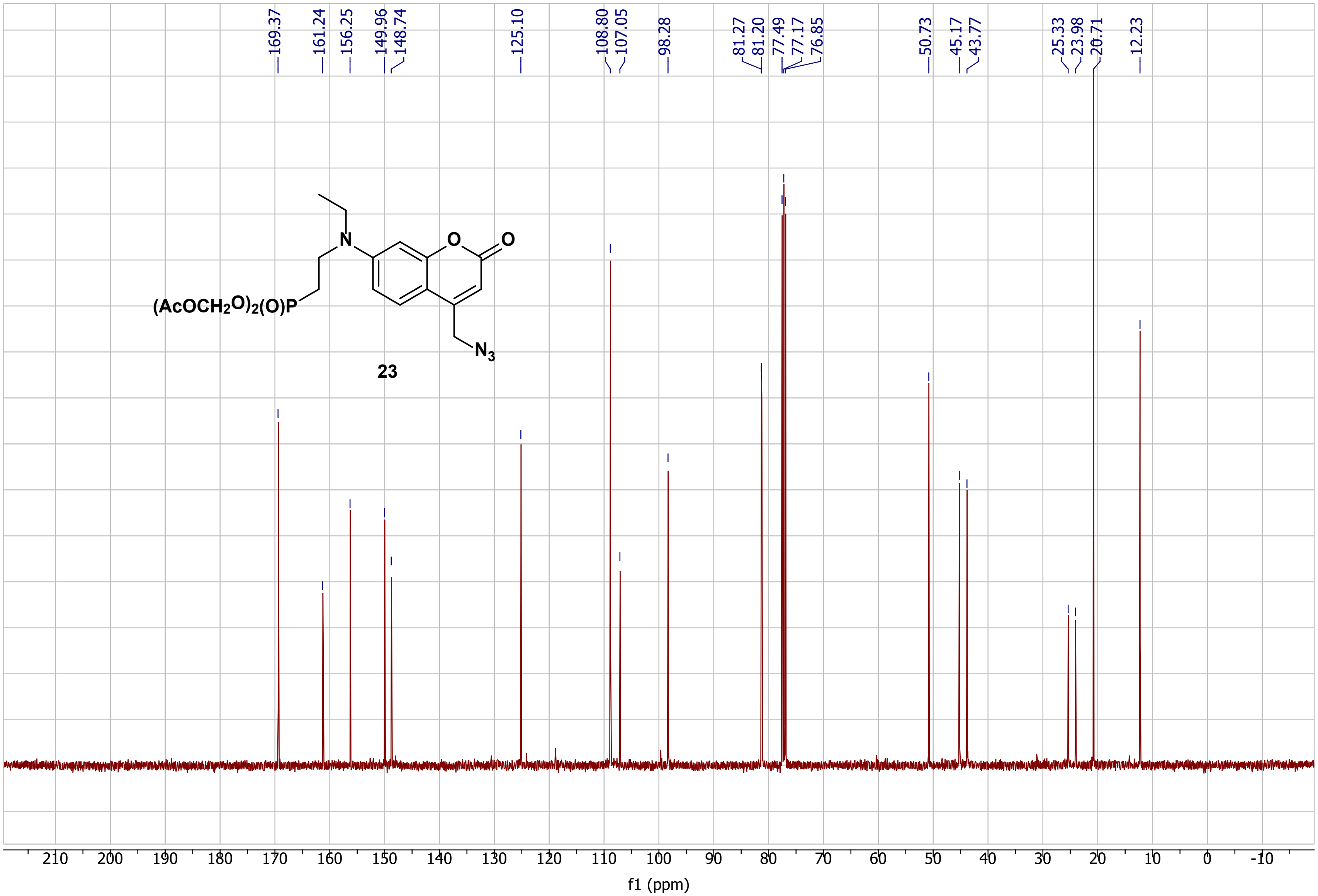

^{13}C NMR spectrum of **23** ($CDCl_3$)

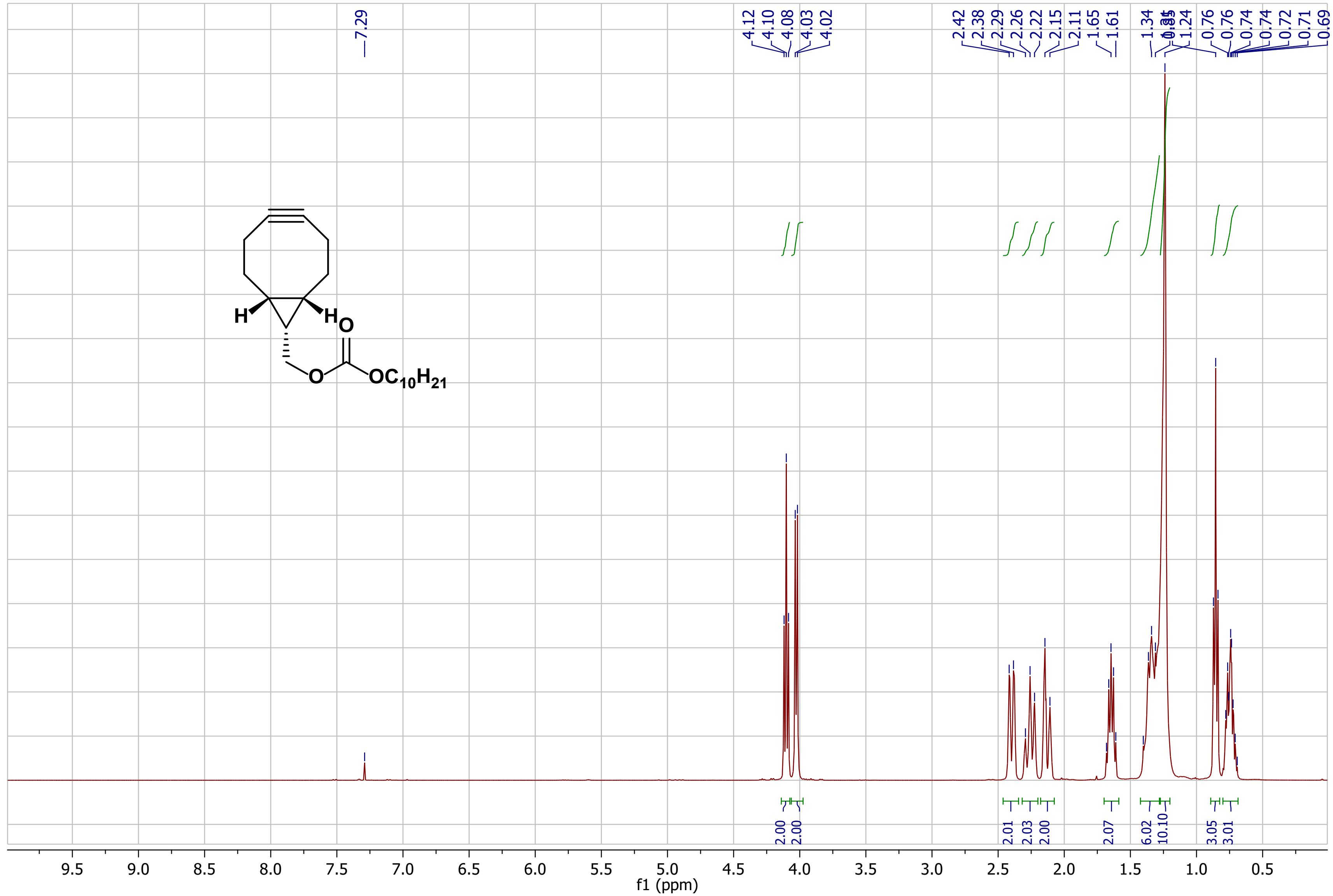

^{1}H NMR spectrum of **25** ($CDCl_3$)

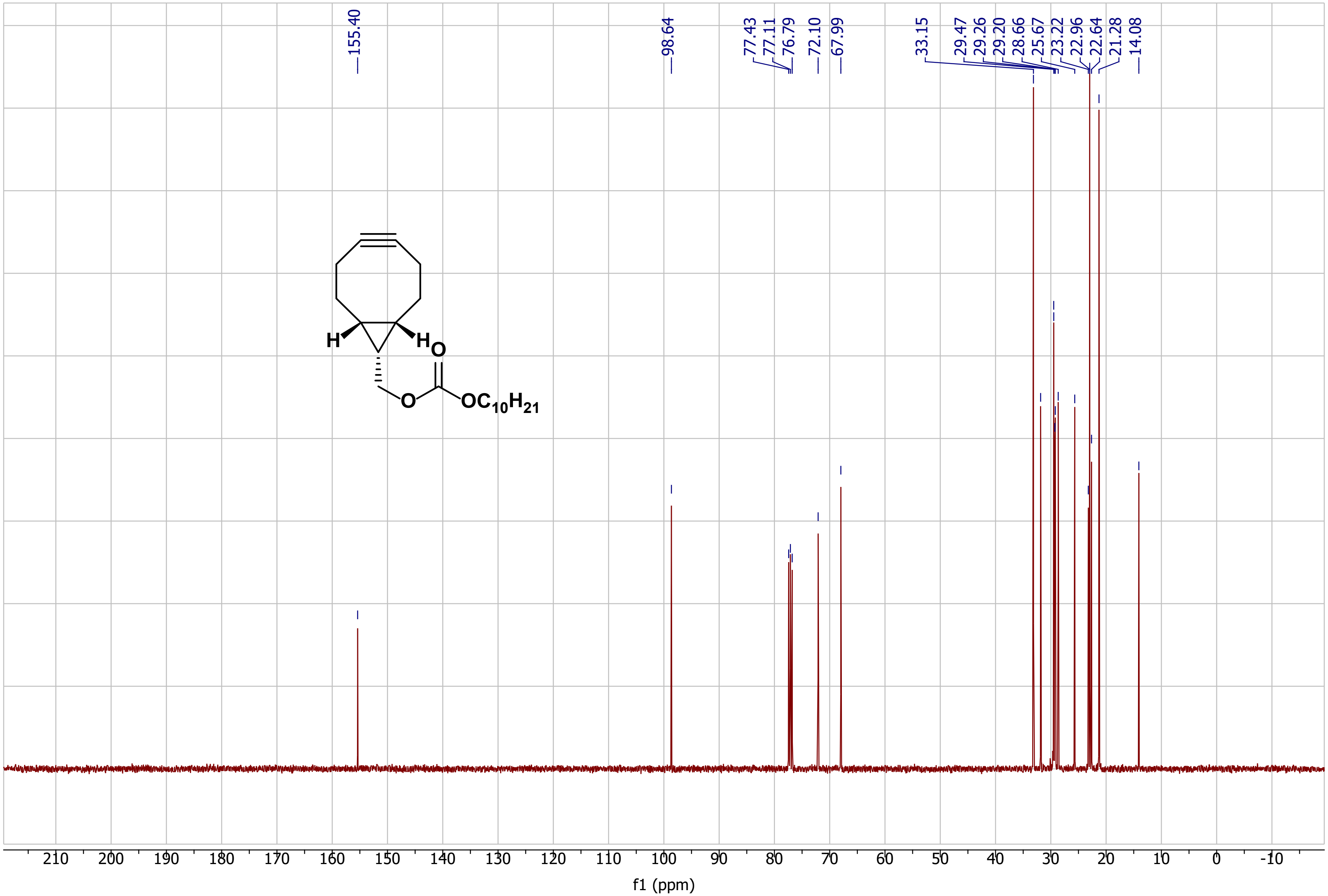

^{13}C NMR spectrum of **25** ($CDCl_3$)

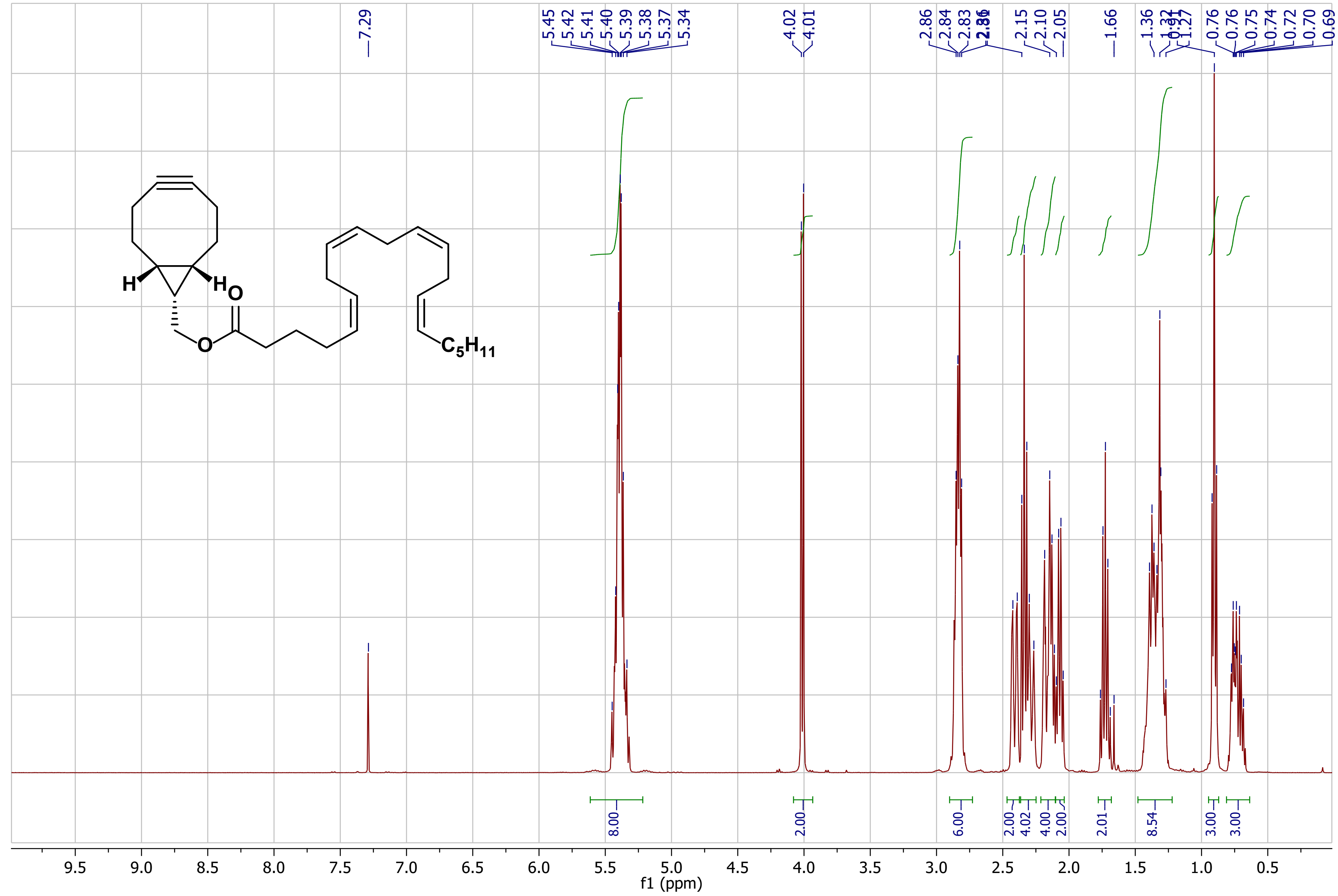

^{1}H NMR spectrum of **26** ($CDCl_3$)

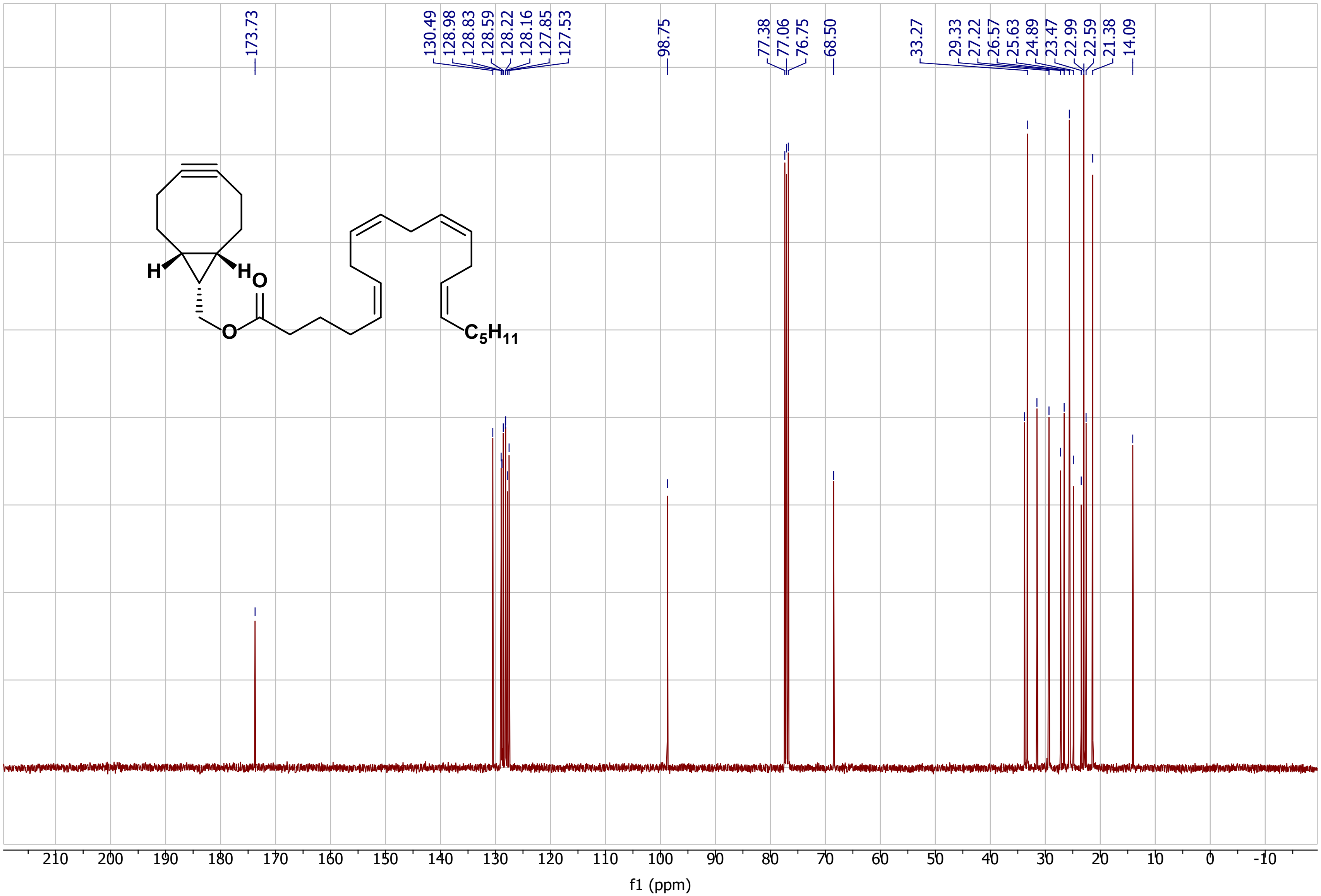

^{13}C NMR spectrum of **26** ($CDCl_3$)

References

1. Nadler, A.; Yushchenko, D. A.; Müller, R.; Stein, F.; Feng, S.; Mulle, C.; Carta, M.; Schultz, C., Exclusive photorelease of signalling lipids at the plasma membrane. *Nat. Commun.* **2015,** *6:10056*, 1-10. doi: 10.1038/ncomms10056
2. Hoffmann, J.-E.; Plass, T.; Nikić, I.; Aramburu, I. V.; Koehler, C.; Gillandt, H.; Lemke, E. A.; Schultz, C., Highly Stable trans-Cyclooctene Amino Acids for Live-Cell Labeling. *Chem. Eur. J.* **2015,** *21*. doi: 10.1002/chem.201501647
3. Rusha, L.; Miller, S. C., Design and application of esterase-labile sulfonate protecting groups. *Chem. Commun.* **2011,** *47*, 2038-2040. doi: 10.1039/c0cc04796a
4. Wanzlick, H.-W., Bis(1,3-diphenylimidazolidinylidene-2). *Organic Syntheses* **1973,** *47*, 14-15. doi: 10.15227/orgsyn.047.0014
5. Wanzlick, H.-W.; Kleiner, H.-J., Nucleophile Carben-Chemie: Darstellung des Bis-[1.3-diphenyl-imidazolidinyliden-(2)]. *Angewandte Chemie* **1961,** *73*, 493. doi: 10.1002/ange.19610731408
